# Supplementary material for: Longitudinal qualitative study of living with neurogenic claudication
Source: BMJ Open. 2022 Sep 14;12(9):e060128. doi: 10.1136/bmjopen-2021-060128 (PMC9476140; doi:10.1136/bmjopen-2021-060128)
Supplement: Supplementary data [file bmjopen-2021-060128supp001.pdf]

Better Outcomes for Older People with Spinal Trouble (BOOST)

Semi-structured interview schedules

All three interview schedules will follow a semi-structured format. Interview content from previous interviews will inform the content and sequencing of questions in the following interviews.

First interview

We will refine the schedule for the first interview during the early phase of data collection, including the content and order of the interview questions. The content of the first interviews will be based around, but not limited to, the following set of questions.

1. Please tell me about yourself (household, work/hobbies/family).
2. Please tell me about your general health.
3. Taking a day that was a fairly normal day for you please tell me about what you do in the day, starting with when you get up.
4. Are there activities that you do regularly that you have not mentioned (prompt for hobbies, recreation, visits, holidays, social clubs, volunteering)
5. What does aging/getting older mean to you? Do you think this affects how active/involved you are?
6. Do you feel restricted in what you are able to do? If so, what are you unable to do that you would like to do? How long have you felt restricted?
    - Prompt for how much of the restriction is due to their back/leg pain.
7. How has this restriction affected different aspects of your life?
    - Prompt for physical, mental, and social aspects of their life.
8. Have you had to change the way you do things because of your back/leg pain?
    - Prompt for how they are adapting their physical environment, their behaviour, and their use of technology for mobility or communication.
9. Have you considered other ways you could change the way you do things to enable you to get round some of your restrictions?
    - Prompt for barriers to changing behaviour.
10. What have you done to try to manage the back/leg pain?
11. What exercise do you take/have you previously taken?
    - Prompt for why they take the exercise (physical/social enjoyment), does it help symptoms, are they restricted by symptoms.
    - Prompt for reasons for not exercising/barriers to exercise.

Griffiths F, *et al. BMJ Open* 2022; 12:e060128. doi: 10.1136/bmjopen-2021-060128

UNIVERSITY OF OXFORD

BOOST
Better Outcomes for Older people with Spinal Trouble

12. How do you feel about your ability to undertake activities/exercise?
13. What are your hopes/concerns for the physiotherapy that you will receive during the BOOST trial?
14. Do you think the BOOST programme will help with your back and leg problems?
15. The BOOST programme encourages people to be more active or do more walking. How do you feel about being asked to increase your activity and walking?

Second interview

The second interview schedule will be further developed after analysis of approximately 10 transcripts from the first round of interviews, and will be based around, but not limited to , the following set of questions:

1. Have there been any changes in your general health since the last interview?
2. How would you describe your activities now? How does this compare to 6 months ago?
3. How would you describe the problems with your back and legs now? How do these compare to 6 months ago?
4. Thinking again about the things that you would like to do, how does this compare to 6 months ago?
    - Prompt for what has changed, and in what way they changed.
5. Do you think that the physiotherapy you received in the BOOST study made any difference to your back and leg problems?
    - Prompt for whether there are change in the way they view their back/leg pain now; has this changed their habits?
6. What was your experience of the BOOST physiotherapy programme?
7. Has anything else made a difference to your back and leg problems?
8. Have your views on aging and activity changed since taking part in the study?

Third interview

The third interview schedule will be developed after analysis of approximately 10 transcripts from the second round of interviews. The interview schedule will encompass, and build on, the questions presented to participants in the second interview.
