# Supplementary material for: Longitudinal qualitative study of living with neurogenic claudication
Source: BMJ Open. 2022 Sep 14;12(9):e060128. doi: 10.1136/bmjopen-2021-060128 (PMC9476140; doi:10.1136/bmjopen-2021-060128)
Supplement: Supplementary data [file bmjopen-2021-060128supp002.pdf]

BMJ Publishing Group Limited (BMJ) disclaims all liability and responsibility arising from any reliance placed on this supplemental material which has been supplied by the author(s)

Supplementary file 2: Change framework

Participant ID	Living arrangements	Treatment arm Best Practice Advice (BPA) or BOOST programme	Participant-reported attendance	Desired treatment outcomes	Pain	6 months	12 months	Pain Trajectory	Baseline Mobility & ADL	6 months	12 months	Mobility & ADL Trajectory	Baseline Psycho impact	6 months	12 months	Psychological impact Trajectory	Baseline social & recreational participation	6 months	12 months	Social & recreational participation Trajectory	General health events	Co-morbid conditions	Coping strategies	Views on aging	Use of mobility aids	Feedback from others	Views on treatment/Trial	Were desired outcomes met?	Description of Turning points
02	Lives with spouse	BPA	Attended 2 BPA sessions	Get better (Symptom improvement)	Slight improvement in symptoms on stopping self-prescribed exercises<br>Loses balance after sustained walking<br>Pain on standing > 5 minutes	Occasional mild pains (no more severe pain)<br>Pain reduced to acceptable level, but did not fully resolve<br>Attributes to doing exercises gently than intensive before; release of trapped nerve<br>Balance slightly improved, still issue<br>Pain on standing > 5 minutes didnt change	Occasional, but no more severe pains<br>No change in pain (Pain reduced but did not fully resolve)<br>Not sure if due to stopping 1-2 exercises or adding other exercises to regime	Initial reduction in pain that was maintained.<br>Participant had reduced pain upon trial entry.	Mostly housebound; mobilises within house as much possible.<br>Manages walking upto 20 minutes maximum<br>If weather is good, manages 30 minutes, split into two or three 10 minute walks	Walking duration improved (30 minutes);<br>Balance improved	Mobilises around house & garden<br>Pain still affects walking<br>No change in Walking duration (remained 15-20 minutes)<br>(some seasonal effect- e.g. able to go out more in summer, in winter no place to sit down)<br>Not overly satisfied with walking- 6/10	Initial increase in mobility and back to baseline status	Frustrated depending on others for simple tasks<br>Depressed for not able to contribute to and attend rotary club events<br>Negative psychological impact of getting assistance from spouse at airports	Increased confidence that back pain wont reappear<br>Focuses more on safety while walking<br>Lacks courage to resume biking due to back not feeling strong enough for this activity<br>Fearful of symptoms returning	Participant feels envious of others who can walk faster<br>Avoids certain activities due to fear of pain	Negative impact remained throughout	Difficulty with walking/standing longer made going on holidays difficult<br>Unable to process ideas for book (due to difficulty with walking)<br>Avoids gardening, house maintainence, long travels and rotary club activities	No information	Avoids activities like gardening.<br>Limited ability to travel long-distances	Participation restrictions remained throughout	No information-Throat narrowing having a major social impact- On external feeding tube	Total 2. Hypertension; throat constriction causing swallowing issues	Adopts graded activity approach (breaks 30 minute walk into shorter walks); Pacing (sits down)<br>No new strategies adopted over time.	Current condition is mainly pain -related (90%) & 10% due to aging<br>Priorities being independant than aging<br>Accepts aging and content with overall health<br>Feels less capable than other people of same age	Does not like using aids because it makes them feel old.	No information available	Not sure if symptom improvement is due to release of trapped nerve or BOOST exercises<br>Gently doing exercises would have helped pain<br>BPA no different to previous physiotherapy participant had<br>Not 100% confident whether exercises helped or not (prev exp)<br>Wanted more information but found BPA insufficient in terms of number of sessions, content and followup.<br>Not 100% sure if improvements were due to BOOST or not<br>Sees no potential for further improvements from Physio and would try other treatments-massage & chiropractic<br>BOOST contributed to 20% in improvements<br>BOOST made participant psychologically better, than physical improvements<br>BOOST helped participant to selfmanage instead of opting for surgery.	NO.<br>No considerable pain relief	External feeding tube for throat constriction at T3<br>At T2 due to symptom improvement, no longer considers surgery for pain management
03	Lives alone; two children live closeby	BPA	Attended 3 BPA sessions	Resume physical and social activities as before	Worse night pain, builds up from day<br>Takes pain medication	Pain worsened over 5 months<br>Constant pain<br>Increased pain (particularly at night)<br>Takes more medication to sleep	Decompression laminectomy, since T2<br>Constant pain<br>Pain intensified<br>(following decompression surgery)<br>Remains on same pain medication, more dose & frequency	Progressive worsening of pain	Reduced walking distance & duration due to pain<br>Sits more when out	Pain inhibits mobility<br>Difficulty to do any ADL	Decompression laminectomy, since T2:<br>No change in Mobility and all activities (maintained)<br>Sits more often when out compared to previous year	Initial decrease in mobility that was maintained	No information	No information	Decompression laminectomy, since T2:<br>No impact on mental health	No impact on mental health	Impacted social life<br>Unable to mow the lawn<br>Avoids activities that involve physical activity, like going to city & around shops<br>Avoids social activities like shopping trips with friends<br>Attends weekly Pilates class (with modified exercises)<br>Stopped church voluntary café work	No change in participation restrictions	Decompression laminectomy, since T2:<br>No change in participation restrictions (Reduced voluntary work and attendance (church));<br>Stopped attending walking group<br>Reduced pace with Pilates class)	Participation restrictions remained throughout	Hip and leg pain-No info- Leg pain intensified after surgery	No comorbid issues reported.	Takes help from others: hired a person to maintain lawns and prune shrubs; daughter cleans stairs<br>After T1, Parks car away from destination, to integrate walking to shopping<br>Avoids doing stairs unnecessarily at home.<br>Modified social outings to reduce walking and standing	Aging is inevitable, but staying active helps staying young<br>NC is age related, accepts living with it<br>Health issues are part of aging<br>Activity is good for aging people	Not interested in trying a mobility aid as did not want to draw attention.	Other people noticed participant's limp while walking.<br>Family keen on considering other treatment options	Less optimistic of BOOST exercises, thinks surgery is final option<br>BOOST exercises were appropriate, but ineffective for pain<br>Exercises are better than nothing and good for physical health<br>BOOST has changed behaviour (increase in exercise level)<br>Pessimistic on prognosis- would worsen<br>BOOST would have been costly<br>BOOST gave quicker access to treatment	NO.<br>Reduced activity and participation.	Intensified pain after L4-5 decompression laminectomy at T3<br>Disappointed with outcome of surgery
04	Lives with spouse with a small back patio garden.<br>Previously moved from two-storey house, as no longer able to manage stairs; close contact with children	BOOST	Attended 11 of 12 group sessions.	Be pain free and return to building trade	Pain has increased since previous levels and constant 24/7.<br>Takes multiple pain meds	No reduction in pain<br>Constant pain and flare ups if he overdoes things<br>Limits amount of pain meds to avoid cognitive side effects (Zombie)	No reduction in pain (maintained)<br>Still in lot of pain<br>Constant pain with intermittent flare ups	Baseline Pain remained throughout	Decreased walking speed<br>Restricted mobility and balance issues<br>Mostly home based, few outside activities due to fear of falling<br>Relies on walking aid (crutch) in & out and family member during outings<br>Unable to do housework<br>Role reversal in house hold activities (spouse washing car, cutting grass)	Getting out more than before, inspite of pain<br>Walking upto 30 minutes, expanded walking environment<br>Improved transfers (in/out car), but not so significantly<br>Still some balance issues with walking<br>Still unable to do housework and gardening	Day is sedentary & home based<br>Confident mobilising inside home than outside<br>Struggles to do activities of daily living (dressing)<br>Avoids walking outside (seasonal effect-cold weather).<br>Mobilises inside and avoids walking in snow and ice	Initial increase in mobility and back to baseline status	Fear avoidance of activity outside-Fear of falling<br>Depressed, frustrated depending on others and feels older than actual age.<br>On anti-depressants<br>Unable to have intimate relationship with spouse<br>Feels worthless & burden to son<br>Fear of falling, leading to paralysis<br>Role reversal in house hold activities (spouse washing car, cutting grass)	Depressed, frustrated depending on others and feels older<br>Feels very depressing in winter<br>Accepts living with NC; would not return to previous level of function<br>Depressed because he expected improvements after BOOST	Depressed, frustrated depending on others and feels older than (maintained).<br>Frustrated as not active for actual age<br>Short tempered due to lack of sleep<br>Mood affected by cold weather (SAD)<br>Feels psychological impact of pain is less in summer<br>Winter has a negative impact on physical & mental health	Negative impact remained throughout	Goes to football match with son; shopping<br>Uses disabled seat; reliant on family to take to match due to concerns about falling<br>Unable to engage with grandchildren (coaching grandson football)	Shopping with spouse;<br>Foot ball outings halted (end of football season)<br>Avoids going out due to discomfort	Stopped going out for drink with son<br>Stopped shopping<br>Unable to sit and laugh and joke with people (socialise) due to pain<br>Avoids going out (fear of falling)<br>Misses involving with grandson's football sport	Progressive worsening.<br>Participant already had positive participation upon trial entry.	Complex multiple issues, Unclear health concern (NC or CS?)- diagnosed Cervical stenosis-Main concern CS	>5. Complex multi-morbid presentation of physical and mental health issues. Hypercholesterolemia, hypertension, stomach ulcer, irritable bowel, Barrett's oesophagus, urinary incontinence, and persistent headaches; Arthritis, cervical stenosis, Depression, OCD, suicidal thoughts	At T1, Moved from a two-storey house to the bungalow as unable to manage stairs.	Physical ability has nothing to do with age<br>Physical condition will worsen on aging<br>Living with a chronic pain condition makes participant feel older than actual age<br>Due to the condition, participant feels not as active for age<br>Being dependent on others make participant feel older for age.	Participant used crutches inside and outside home and accompanied by family members at outings.<br>Stopped using crutches by T3.	Spouse noted improvements in getting out of chair<br>Other group participants noted improvements in posture and movement.	Previous physiotherapy experience mixes: Hesitant doing anything that would cause pain & not expecting anything from BOOST<br>Loved BOOST sessions.<br>Throughly enjoyed the study<br>Found educational part useful.<br>Around T2, unsure if back pain has actually improved or has been overshadowed by neck pain<br>BOOST didn't change anything drastically in terms of improvements<br>Felt physical improvements did not lead to meaningful functional improvements<br>BOOST exercises were difficult to begin with-weight exercises were painful, took 2 sessions to get used to<br>BOOST exercises improved muscle tone<br>[illegible]	NO.<br>No reduction in pain.	Diagnosis of Cervical stenosis around T2, Main health concern at T2 & T3<br>Around T3, living circumstances impacted by financial difficulty with home heating.
24	Lives with spouse. Weekly contact with daughter	BOOST	Attended all 12 group sessions	Get better sleep	Distinct symptoms at day & night.<br>Predominantly occurs at night.<br>Night time burning pain<br>Uses gel or Paracetamol or a hot drink for pain relief	Reduced day time pain;<br>Night pain unchanged.	No change (further reduction) in day pain;<br>Night pain unchanged.	Initial reduction in day time pain was maintained.<br>Baseline night time Pain remained throughout	Restricted ability to stand or walk any length of time; crawls on stairs at home;<br>Difficulty accessing car, trains.<br>Reliant on spouse or public transport to go out<br>Winter makes participant less active with a feeling of tiredness all the time (SAD)	Increased walking (up to 2 hours), balance, and ability to negotiate stairs in upright position;<br>Confident using steps and slopes without fear of falling<br>Improved balance, example: Can carry objects while walking	[illegible]	[illegible]	[illegible]	[illegible]	[illegible]	[illegible]	[illegible]	[illegible]	[illegible]	[illegible]	[illegible]	[illegible]	[illegible]	[illegible]	[illegible]	[illegible]	[illegible]	[illegible]	[illegible]
30	Lives alone; Weekly contact with family	BOOST	Attended 10 group sessions	Be pain-free	Some improvement in pain in previous 2 weeks of T1 (attributes to pain medication & daily flexion exercises)<br>Pain generally present on walking; eases after ~1 hour	Reduction in pain, in terms of area and intensity	Further substantial reduction in pain (75% improvement)<br>Pain generally present on walking, eases as participant becomes mobile	Progressive improvement<br>Participant had reduced pain upon trial entry.	Walking has slightly improved in previous two weeks of T1.<br>Walking only short distances, even within the house, triggers leg pain<br>Not 100% happy with mobility<br>Stooped posture while walking<br>Difficulty to do grocery shopping<br>Mobility limited first thing in morning & returns to level that participant considers 'normal' over the day	Increased walking on a daily basis (from walking within short distances even within home)<br>Pleased with physical and social activity levels	[illegible]	[illegible]	[illegible]	[illegible]	[illegible]	[illegible]	[illegible]	[illegible]	[illegible]	[illegible]	[illegible]	[illegible]	[illegible]	[illegible]	[illegible]	[illegible]	[illegible]	[illegible]	[illegible]
[illegible]	[illegible]	[illegible]	[illegible]	[illegible]	[illegible]	[illegible]	[illegible]	[illegible]	[illegible]	[illegible]	[illegible]	[illegible]	[illegible]	[illegible]	[illegible]	[illegible]	[illegible]	[illegible]	[illegible]	[illegible]	[illegible]	[illegible]	[illegible]	[illegible]	[illegible]	[illegible]	[illegible]	[illegible]	[illegible]
