# Supplementary material for: Longitudinal qualitative study of living with neurogenic claudication
Source: BMJ Open. 2022 Sep 14;12(9):e060128. doi: 10.1136/bmjopen-2021-060128 (PMC9476140; doi:10.1136/bmjopen-2021-060128)
Supplement: Supplementary data [file bmjopen-2021-060128supp003.pdf]

**Supplementary file 3:** Patterns of change in biopsychosocial domains at T1, T2, and T3 (ordered by treatment arm and participant study code)

| | | Pain | | | Mobility and ADL | | | Psychological Impact | | | Social and recreational participation | | |
|---|---|---|---|---|---|---|---|---|---|---|---|---|---|
| Participant IDs | Treatment arm | Baseline | 6 months | 12 months | Baseline | 6 months | 12 months | Baseline | 6 months | 12 months | Baseline | 6 months | 12 months |
| 01 | BOOST | | | | | | | | | | | | |
| 04 | BOOST | | | | | | | | | | | | |
| 05 | BOOST | | | | | | | | | | | | |
| 06 | BOOST | | | | | | | | | | | | |
| 09 | BOOST | | | | | | | | | | | | |
| 11 | BOOST | | | | | | | | | | | | |
| 14 | BOOST | | | | | | | | | | | | |
| 24 | BOOST | | | | | | | | | | | | |
| 25 | BOOST | | | | | | | | | | | | |
| 26 | BOOST | | | | | | | | | | | | |
| 28 | BOOST | | | | | | | | | | | | |
| 30 | BOOST | | | | | | | | | | | | |
| 39 | BOOST | | | | | | | | | | | | |
| 41 | BOOST | | | | | | | | | | | | |
| 43 | BOOST | | | | | | | | | | | | |
| 57 | BOOST | | | | | | | | | | | | |
| 02 | BPA | | | | | | | | | | | | |
| 03 | BPA | | | | | | | | | | | | |
| 07 | BPA | | | | | | | | | | | | |
| 08 | BPA | | | | | | | | | | | | |
| 15 | BPA | | | | | | | | | | | | |
| 31 | BPA | | | | | | | | | | | | |
| 32 | BPA | | | | | | | | | | | | |
| 33 | BPA | | | | | | | | | | | | |
| 34 | BPA | | | | | | | | | | | | |
| 37 | BPA | | | | | | | | | | | | |
| 38 | BPA | | | | | | | | | | | | |
| 47 | BPA | | | | | | | | | | | | |
| 50 | BPA | | | | | | | | | | | | |
| 53 | BPA | | | | | | | | | | | | |

BOOST: BOOST programme; BPA: Best Practice Advice; ADL: Activities of Daily Living



Griffiths F, *et al. BMJ Open* 2022; 12:e060128. doi: 10.1136/bmjopen-2021-060128

**Legend**

| | |
|---|---|
| | When participants already had reduced pain/improved sleep/ improved mobility/ improved social and recreational participation/positive psychological impact at entry to trial or had improvements compared to a previous time point. |
| | Further reduction in pain, improved sleep/increase in mobility/ increase in social and recreational participation/positive psychological impact compared to a previous time point. |
| | Continued reduction in pain/improved sleep/increase in mobility/ increase in social and recreational participation/positive psychological impact compared to a previous time point. |
| | When participants already had increased pain/sleep disturbances/decreased mobility/ social and recreational participation/negative psychological impact at entry to trial or worsened status compared to a previous time point. |
| | Further increase in pain/sleep disturbances/decrease in mobility/decrease in social and recreational participation/negative psychological impact compared to a previous time point. |
| | Continued increase in pain/sleep disturbances/decrease in mobility/decrease in social and recreational participation/negative psychological impact compared to a previous time point. |
| | No change in pain/sleep/mobility/social and recreational participation/psychological impact compared to a previous time point. |
| | Lack of sufficient information from interviews. |
| | When pain, sleep, mobility, social and recreational participation, or psychological impact influenced by other musculoskeletal (MSK)/non-MSK issues or major health events. |



Griffiths F, *et al. BMJ Open* 2022; 12:e060128. doi: 10.1136/bmjopen-2021-060128
