# Supplementary material for: Longitudinal qualitative study of living with neurogenic claudication
Source: BMJ Open. 2022 Sep 14;12(9):e060128. doi: 10.1136/bmjopen-2021-060128 (PMC9476140; doi:10.1136/bmjopen-2021-060128)
Supplement: Author's manuscript [file bmjopen-2021-060128.draft_revisions.pdf]

BMJ Open is committed to open peer review. As part of this commitment we make the peer review history of every article we publish publicly available.

When an article is published we post the peer reviewers' comments and the authors' responses online. We also post the versions of the paper that were used during peer review. These are the versions that the peer review comments apply to.

The versions of the paper that follow are the versions that were submitted during the peer review process. They are not the versions of record or the final published versions. They should not be cited or distributed as the published version of this manuscript.

BMJ Open is an open access journal and the full, final, typeset and author-corrected version of record of the manuscript is available on our site with no access controls, subscription charges or pay-per-view fees (http://bmjopen.bmj.com).

If you have any questions on BMJ Open's open peer review process please email info.bmjopen@bmj.com

BMJ Open

Longitudinal qualitative study of living with neurogenic claudication

Journal:	*BMJ Open*
Manuscript ID	bmjopen-2021-060128
Article Type:	Original research
Date Submitted by the Author:	15-Dec-2021
Complete List of Authors:	Griffiths, Frances; University of Warwick, Warwick Medical School<br>Srikesavan, Cynthia; University of Oxford, Nuffield Department of Orthopaedics, Rheumatology, and Musculoskeletal Sciences<br>Ward, Lesley; Northumbria University, Department of Sport, Exercise & Rehabilitation<br>Boniface, Graham; University of Oxford, Nuffield Department of Orthopaedics, Rheumatology, and Musculoskeletal Sciences<br>Williamson, Esther; University of Oxford, Nuffield Department of Orthopaedics, Rheumatology and Musculoskeletal Sciences<br>Lamb, Sarah; University of Exeter, College of Medicine and Health; University of Oxford, Nuffield Department of Orthopaedics, Rheumatology and Musculoskeletal Sciences
Keywords:	Back pain < ORTHOPAEDIC & TRAUMA SURGERY, Spine < ORTHOPAEDIC & TRAUMA SURGERY, QUALITATIVE RESEARCH, Rehabilitation medicine < INTERNAL MEDICINE

SCHOLARONE™
Manuscripts

I, the Submitting Author has the right to grant and does grant on behalf of all authors of the Work (as defined in the below author licence), an exclusive licence and/or a non-exclusive licence for contributions from authors who are: i) UK Crown employees; ii) where BMJ has agreed a CC-BY licence shall apply, and/or iii) in accordance with the terms applicable for US Federal Government officers or employees acting as part of their official duties; on a worldwide, perpetual, irrevocable, royalty-free basis to BMJ Publishing Group Ltd ("BMJ") its licensees and where the relevant Journal is co-owned by BMJ to the co-owners of the Journal, to publish the Work in this journal and any other BMJ products and to exploit all rights, as set out in our licence.

The Submitting Author accepts and understands that any supply made under these terms is made by BMJ to the Submitting Author unless you are acting as an employee on behalf of your employer or a postgraduate student of an affiliated institution which is paying any applicable article publishing charge ("APC") for Open Access articles. Where the Submitting Author wishes to make the Work available on an Open Access basis (and intends to pay the relevant APC), the terms of reuse of such Open Access shall be governed by a Creative Commons licence – details of these licences and which Creative Commons licence will apply to this Work are set out in our licence referred to above.

Other than as permitted in any relevant BMJ Author's Self Archiving Policies, I confirm this Work has not been accepted for publication elsewhere, is not being considered for publication elsewhere and does not duplicate material already published. I confirm all authors consent to publication of this Work and authorise the granting of this licence.

For peer review only

Title page

Title of the article
Longitudinal qualitative study of living with neurogenic claudication

Authors
[1]Frances Griffiths, [2]Cynthia Srikesavan, [3]Lesley Ward, [4]Graham Boniface, [5]Esther Williamson, [6]Sarah E Lamb

Full name, Postal address and email of the Corresponding author
Dr Esther Williamson
Nuffield Department of Orthopaedics, Rheumatology and Musculoskeletal Sciences, University of Oxford, Windmill Road, Oxford OX3 7LD, United Kingdom.

Email: esther.williamson@ndorms.ox.ac.uk

Full name, department, institution, city and country of all co-authors
[1]Warwick Medical School, University of Warwick, Coventry, United Kingdom.
[2, 4, 5, 6]Nuffield Department of Orthopaedics, Rheumatology, and Musculoskeletal Sciences, University of Oxford, Windmill road, Oxford, United Kingdom.
[3]Department of Sport, Exercise & Rehabilitation, Northumbria University, United Kingdom.
[6]College of Medicine and Health, Institute for Health Research, University of Exeter, St Luke's Campus, Heavitree Road, Exeter, United Kingdom.

Word count, excluding title page, abstract, references, figures and tables: 5389

Strengths and limitations of this study

- In-depth interviews at three time points focus on participant's current experience; comparison across time was undertaken during analysis so was not reliant on participant interpretation of past-experience.
- Analysis summarises succinctly a large qualitative data set.
- Summarising data will have resulted in loss of nuance in our analysis.
- The results present one set of trajectory category membership for participants; some participants could be categorised differently.
- Our interest in successful aging shaped our path through analysis; a different interest is likely to result in different trajectory descriptions.

For peer review only - http://bmjopen.bmj.com/site/about/guidelines.xhtml

Abstract

Objectives
Neurogenic claudication (NC) causes pain and reduced mobility, particularly in older people and can negatively affect mental and social well-being, so limiting successful aging. This qualitative study explored how people with NC change over 12 months.

Design
A longitudinal qualitative study using semi-structured interviews.

Setting
Participants were recruited from a UK clinical trial of a physiotherapy intervention for NC.

Participants
Interviews were undertaken at baseline, one month after receiving any intervention and at 12 months. We analysed 30 sets of three interviews.

Results
Interview data was summarised for each time point into biopsychosocial domains: pain, mobility and activities of daily living, psychological impact and social and recreational participation. Through comparative analysis we explored participant trajectories over time.

Progressive improvement in at least one domain was experienced by 13 participants but there was variability in trajectories with early improvements that remained the same, transient changes and no change also commonly observed.

Eleven participants described co-present improvement trajectories in all domains. Three participants described co-present improvement in all domains except participation; one had never stopped their participation and two had unattainable expectations. Five participants described co-present improvement in one domain and deterioration in another and 14 participants described co-present no change in one domain and change in another.

There was evidence of interaction between domains e.g. improved mobility led to improved participation and for some participants, specific factors influenced change. Of the 15 participants who experienced improved participation, ten reported improvements in all other domains and five participants did not; for two, pain did not prevent participation, one used a walking aid and two had a positive psychological outlook.

Conclusion
The daily lived experiences of older adults with NC are variable and include interaction between biopsychosocial domains. Therapist understanding of these trajectories and their interactions may help to provide personalised therapy

Keywords: Neurogenic claudication; Longitudinal; Interviews; Trajectory

For peer review only

Main text

Background

Neurogenic claudication (NC) is caused by narrowing of the spinal canal (lumbar canal stenosis) most often due to osteoarthritis of the spine or spondylosis. It is frequently reported in adults aged above 60 years (1, 2). NC usually presents as pain and discomfort radiating from the back into the buttocks and legs in a unilateral or bilateral pattern. It is provoked by walking or prolonged standing and relieved by sitting or lumbar flexion (3). The pain often occurs intermittently. Other signs and symptoms may include weakness, altered sensations such as pins and needles and numbness, fatigue and gait changes (3). Pain in the lower back is usual but not a necessary diagnostic feature but regardless NC is considered a low back pain condition. NC is associated with decreased mobility and independence for older people, restriction of social activities, a negative emotional impact, and frailty (4, 5). The impact of back and leg pain consistent with NC on health status is more marked than for people with other patterns of back and leg pain; for older adults, compared to those who report back pain only or have leg pain which is not consistent with NC, those with NC report lower quality of life and are more likely to be frail, be less confident to walk ½ a mile and to report a decline in their mobility over the last year (1). The variation in symptom trajectory for people with NC is illustrated by a study of 146 people with NC followed up for 3 years (without surgical treatment), symptoms improved for about one third of patients, remained unchanged for half the patients and worsened in a few (6).

Ageing successfully is considered a key mechanism by which ageing populations can maximise their health and longevity and minimise their need to access health care systems (7). The concept of successful aging considers physical and mental health along with social and recreational engagement (7) and since its conception has been expanded to include living with multi-morbidities and chronic conditions such as NC (8-10). Our concern in this study is whether the symptoms of NC may lead to older people being unable to age successfully. We were particularly concerned to understand whether and how NC impacted on social and recreational participation and how this changed over time.

Qualitative evidence on living with back pain

The interaction physical and mental health and social and recreational engagement has been explored in qualitative studies of people living with low back pain and synthesised in a meta-ethnography (11). The interrelated themes identified were: impact of pain on self, use of coping strategies and impact of pain on engagement with others. The meta-ethnography concluded that chronic low back pain is complex, dynamic and multidimensional with those living with the pain experiencing distressing pain and loss with lowered self-worth, stigma, depression, premature aging, and fear of the future. A later cross-sectional qualitative study of people with NC, suggests that pain, physical ability and emotions are inter-related and at times inseparable (5). The meta-ethnography identified a lack of longitudinal studies. A subsequent longitudinal study interviewed eight people living with back pain annually over two years (12). Although pain and loss impacted on all areas of the participants lives, two different patterns of change over time were identified: participants who saw their pain a physical problem and a cause of loss and did not re-establish social and recreational activities, and those who experienced pain relief and were less enmeshed in the pain experience so able to look to the future (12).

For peer review only - http://bmjopen.bmj.com/site/about/guidelines.xhtml

Such longitudinal qualitative studies provide a depth of understanding to complement existing panel studies (13) and build on the idea of illness trajectory (14), biographical disruption (15) and understanding individuals as unique, complex and adaptive (16-18). Our study aims to provide this depth of understanding of people living with NC: how their NC impacts on their function and engagement and how this changes over time. It builds further on longitudinal qualitative studies in the health domain (19).

<u>The BOOST trial</u>

This qualitative study was embedded within the Better Outcomes for Older people with Spinal Trouble (BOOST) trial (20, 21), a multi-centre, randomised controlled trial with two treatment arms. It compared a 12-week group-based physical and psychological intervention (the BOOST programme) to individual best practice advice (BPA) for older adults (65+ years) with NC. The BOOST programme comprised group discussions that were underpinned by cognitive behavioural techniques, an individually tailored exercise and walking circuit, and home exercises. BPA comprised home exercises and self-management (1-3 appointments with a physiotherapist). Participants aged 65 years or over with the clinical presentation of NC symptoms were recruited from primary care practices of the National Health Service (NHS) in the United Kingdom (UK). People living in residential care or nursing homes, those registered blind and those with a terminal health condition or on a waiting list for surgery or unable to walk 3m without the support of another person or unable to provide informed signed consent were not included. Participants were randomised in a 2:1 ratio (BOOST programme: BPA). Full trial protocol details are reported elsewhere (21).

Methods

<u>Study design</u>

We used a longitudinal qualitative research design to understand the individual at the time of each interview (22) rather than seeking from the individuals their own narrative of change (23). We aimed to interview participants three times: at trial baseline (after randomisation but before the participant knew their treatment allocation) (T1); one month after any intervention (T2) and 12 months post-randomisation (T3). TI interviews were via face-to-face at participant's home, with T2 and T3 by telephone. Where participants had difficulty using the telephone, T2 and T3 interviews were undertaken face-to-face.

<u>Ethics approval</u>

Ethics approval was given by the London-Brent National Research Ethics Committee (REC number 16/LO/0349) on 03 March 2016.

<u>Participants and Recruitment</u>

All participants recruited to the BOOST trial (20, 21) were eligible for the interview study and consent to invite participants for interview was collected as part of trial procedures. We invited participants consecutively from each recruitment site over the 25-month trial recruitment period (August 2016 to August 2018), stratifying to ensure balance between range of age, gender, ethnicity, treatment allocation (ratio 1:1) and recruitment site location.

For peer review only - http://bmjopen.bmj.com/site/about/guidelines.xhtml

To ensure sufficient information power (24), we aimed for 30 participants retained at 12 months and so aimed to recruit approx. 60 participants to allow for 50% attrition.

Data collection

We obtained written consent at the start of the first interview and reaffirmed this verbally at follow-up interviews. We used semi-structured interview schedules (**Supplementary file 1**). We used the present tense when asking questions to encourage participants to focus on their current state (22). In T1 interviews, we explored the impact of NC on participant physical and psychosocial health including NC and their beliefs around exercise and ageing. In T2 and T3 interviews we explored similar issues along with their experiences of the BOOST trial interventions. Before T2 and T3 interviews we reviewed for each participant the data captured in their earlier interviews and made notes about the participant and their context. We used this knowledge in the interviews to rapidly establish rapport on the telephone and enable quick clarifications. For example:

> Participant: I go to my club.
>
> Interviewer: Is that the ladies club you mentioned last time we met?

All interviews were audio-recorded onto encrypted recorders and stored securely at University of Oxford. During transcription/summarising all personal identifiers were removed.

Data analysis

Throughout our analysis we compared data across time and between participants and drew on existing evidence-based concepts and definitions related to back pain and NC. We mapped the data into a framework matrix (25) then explored the matrix (26, 27) for change processes related to NC. This involved summarising patterns of change being experienced at each time point, trajectories of change between time points (27) co-presence of trajectories, interactions between trajectories and turning points. In our analysis of data at each time point we used an analysis approach we had previously developed (18) and applied (22). We focused on current experience and put to one side data where the participant was interpreting past-experience. This allowed us to compare what was current at each time point and to manage the volume of complex data (28). We describe each analysis step below. Analysis was conducted in Excel.

Step 1: Development of individual participant frameworks

We started analysis on complete interview sets (T1, T2 and T3). We selected sets where the participant provided rich data about the experience of NC. After selecting 30 sets (90 interviews) and undertaking initial analysis (see Step 2 below) we read and discussed our remaining data to check whether it was revealing new themes; we determined we had achieved data saturation(29). All T1 interviews were transcribed verbatim along with the first 10 T2 and 10 T3 interviews we analysed. The remaining interviews were analysed directly from audio recordings. To immerse ourselves in this large volume of data we initially wrote structured pen portraits(30) at each time point for ten data sets. This involved reading and rereading the data, clarifying our focus on current experience related to NC, summarising participant experiences and context with illustrative quotes, and developing a structure that worked across the 10 data sets. From these we developed and refined a coding template. We used this to thematically code all thirty interview sets, writing mini statements with illustrative quotes for each relevant code/sub code at each time point. The resulting 30 individual participant frameworks were used for subsequent analysis.

For peer review only - http://bmjopen.bmj.com/site/about/guidelines.xhtml

Step 2: Development of change framework

After team discussions, we summarised the coded data for each participant at each time point into the following biopsychosocial domains: pain, mobility and activities of daily living (ADL), psychological impact, social and recreational participation and sleep. For example, to summarise mobility status at T1, we collated and summarised data from codes: current mobility, activities reduced, activities stopped, activities increased, and sub-codes such as physical impact. Participants reported a range of symptoms including numbness, tingling and heaviness. However, the symptom that consistently predominated across all participant's accounts of their experience of NC was pain; we therefore summarised the experience of pain in the framework. We included data that conveyed the pattern of change each participant was experiencing at the time of each interview. For example, a gradual improvement or worsening as experienced day-to-day/week-to-week. We added to the change framework participant age, gender, ethnicity, living arrangement, group allocation in the trial, health events and co-morbid conditions. We summarised participant coping strategies, views on aging, use of mobility aids and feedback from others.

Step 3: Identification and categorisation of participant trajectories

To identify participant trajectories for each biopsychosocial domain, we compared the summaries in the change framework for what increased or emerged over time, what decreased or ceased over time and what remained consistent over time (26). We added a description of this trajectory to the change framework. In a new table we presented for each domain the colour coded trajectory for each participant. Each team member then independently, by eye, ordered the participants so those with the most similar combination of trajectories were adjacent. The team then compared their resulting tables, discussed similarities and differences, and agreed on one version of the table. Using this table, we then independently categorised the participants into groups based on similarity of trajectories and discussed and agreed categorisation.

Step 4: Exploring the nature of the trajectories

Identifying and categorising co-presence and interaction between the trajectories of the individual biopsychosocial domains

We scrutinised the change framework for each participant and compared participants to understand which trajectories were co-present at each time point and so may have interacted with each other. For example, a reduction in pain was accompanied by improvement in mobility or deterioration in psychological outlook was accompanied by reduced social or recreational participation. We returned to participant data to check whether the participant reported interaction between the trajectories. We grouped similar types of co-presence and explored how these related to our trajectory categories. Given our interest in successful aging, we particularly explored how social and recreational engagement changed along with other domains.

Identification of health-related turning points

We examined the data for health-related turning points mentioned by the participant; what the participants considered significant change in their health during the study timeframe.

Totality of change

From the whole change framework we examined factors that were not inseparable from trajectory category although are likely to be interrelated (5). Factors included whether they

For peer review only - http://bmjopen.bmj.com/site/about/guidelines.xhtml

lived alone, how they considered aging and use of mobility aids, and strategies for coping with NC in their daily lives. We identified what was similar and different between participants in the different trajectory categories.

Study team roles

SEL and EW (health service researchers and physiotherapists) led the BOOST programme of work including this study and the BOOST trial. FG (general medical practitioner, medical sociologist and qualitative researcher) led the qualitative study team that included LW (qualitative health researcher), CS (physiotherapist and a qualitative researcher) and GB (physiotherapy researcher). All contributed to qualitative study design, reading data, thematic and comparative analysis, synthesis, interpretation and reporting. LW undertook all interviews. LW and FG reviewed the interview schedules to improve the clarity and flow of the questions. CS worked with FG on analysis with GB providing independent review of coding/summaries. Any discrepancies were resolved through discussion. EW and SEL reviewed each step of the analysis and provided feedback to FG, LW, CS, and GB.

This study team were not involved in the delivery of BOOST interventions or outcome assessments in the trial. FG, LW, CS, GB remained blinded to the results of the BOOST trial until they had completed data coding and analysis.

Reporting

The Standards for Reporting Qualitative Research (SRQR) guidelines (31) were used to report the study. Quotations from interviews are labelled with participant number and interview T1, T2 or T3.

Results

The data

Participant attrition across the three time points was lower than expected, with 49 of the 60 participants (82%) providing three interviews. Reasons for withdrawal were: withdrawal from clinical trial (n=3), ill health/awaiting investigations (n=4), increased pain after receiving BOOST programme sessions (n=2), declined third interview (n=3), unable to contact (n=3) and withdrawn by researcher as participant postponed >6 months (n=2). Initial interviews averaged 70 minutes (range 44-93 minutes), with second and third interviews averaging 36 minutes and 32 minutes respectively.

Participant characteristics

There was little difference in terms of age (average 75 years), gender and ethnicity between trial, interview, and analysis cohorts and were similar in terms of measures related to NC. (**Table 1**).

For peer review only - http://bmjopen.bmj.com/site/about/guidelines.xhtml

Table 1: Description of BOOST trial participants, those interviewed and those included in the qualitative analysis presented in this paper. Values are N (%) or mean [SD] or median {IQR} as specified

Participant Characteristics	BOOST trial cohort<br>N (%)<br>Mean [SD]<br>Median {IQR}	Interview cohort<br>N (%)<br>Mean [SD]<br>Median {IQR}	Interview analysis cohort<br>N (%)<br>Mean [SD]<br>Median {IQR}
Total participants	435	59	30
Age (years at baseline)	74.9 [6.0]	75.5 [6.2]	75.1 [5.7]
Gender			
Female	246 (56.6)	31 (52.5)	14 (46.7)
Male	189 (43.4)	28 (47.5)	16 (53.3)
Ethnicity			
White British/White Other	400 (92)	52 (87)	26 (86.6)
Black British	12 (2.8)	3 (5)	2 (6.7)
Indian	9 (2.1)	3 (5)	2 (6.7)
Pakistani	6 (1.4)	1 (2)	0 (0)
Confidence walking long distance	5.6 [3.3]	5.1 [3.3]	4.7 [3.0]
Oswestry disability index	32.9 [13.9]	32.7 [13.5]	34.6 [14.5]
Tilburg frailty index	4.5 [2.6]	4.8 [2.8]	5.6 [2.8]
Exercise self-efficacy, median {IQR}	69 {53, 80}	69 {43, 86}	52. 5 {37, 81}
Fear avoidance beliefs	12.9 [5.9]	14.0 [6.3]	14.1 [6.4]
Six-minute walk test	255.4 [99.1]	249.3 [96.9]	227.7 [91.9]
Self-management rating scale	6.08 [1.80]	6.0 [1.7]	6.1 [1.7]
**Treatment allocation in the trial[1]**			
BOOST programme (BOOST)	292 (67.1)	32 (53)	16 (53.3)
Best practice advice (BPA)	143 (32.9)	27 (45)	14 (46.7)
**Complete sets of three interviews**		49	30

Abbreviations: BOOST: Better Outcomes for Older people with Spinal Trouble; SD: Standard Deviation: IQR: Interquartile Range

Scale range and direction

Confidence in ability to walk long distance (1 to 10, lower values worse)
Oswestry Disability Index V2.1a (0 to 100, higher values worse)
Tilburg Frailty Index (0 to 15, higher values worse)
Exercise self-efficacy scale (0 to 90, lower values worse)
Fear Avoidance Beliefs Questionnaire Physical Activity (0 to 24, higher values worse)
Six-minute walk test (distance covered walking for 6 minutes, lesser distance worse)
Self-management rating scale (0 to 10, lower values worse)

For peer review only - http://bmjopen.bmj.com/site/about/guidelines.xhtml

For peer review only

Analysis results

Analysis steps 1 to 3: participant trajectories

From summarising patterns of change from our individual participant frameworks (**See Supplementary file 2** for an example) into our change framework (**Supplementary file 3**) we identified patterns of change for each participant in each domain (**Supplementary file 4**). We then summarised the trajectory across the three timepoints (see **Table 2)**. We omitted sleep as a domain as although a question on sleep was asked in each interview many interviewees did not provide data.

For peer review only

Table 2: Participant trajectories in the biopsychosocial domains

For peer review only - http://bmjopen.bmj.com/site/about/guidelines.xhtml

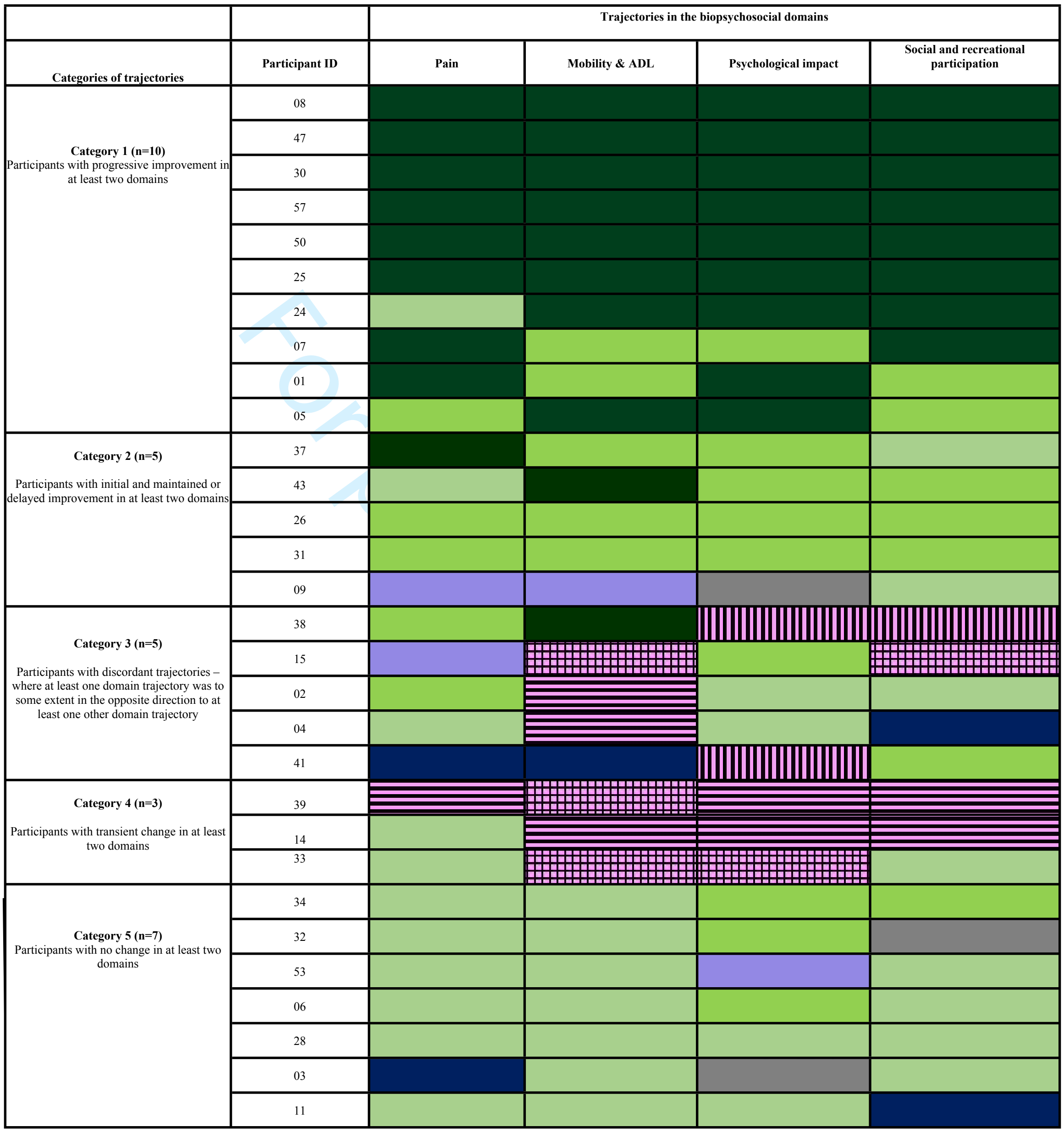

ADL: Activities of Daily Living

For peer review only - http://bmjopen.bmj.com/site/about/guidelines.xhtml

Table 2: Participant trajectories in the biopsychosocial domains

Categories of trajectories	Participant ID	Pain	Mobility & ADL	Psychological impact	Social and recreational participation
**Category 1 (n=10)** Participants with progressive improvement in at least two domains	08				
	47				
	30				
	57				
	50				
	25				
	24				
	07				
	01				
	05				
**Category 2 (n=5)** Participants with initial and maintained or delayed improvement in at least two domains	37				
	43				
	26				
	31				
	09				
**Category 3 (n=5)** Participants with discordant trajectories – where at least one domain trajectory was to some extent in the opposite direction to at least one other domain trajectory	38				
	15				
	02				
	04				
	41				
**Category 4 (n=3)** Participants with transient change in at least two domains	39				
	14				
	33				
**Category 5 (n=7)** Participants with no change in at least two domains	34				
	32				
	53				
	06				
	28				
	03				
	11				

Legends for trajectories

No change: No change in pain or in the decreased mobility decreased social and recreational participation or negative psychological impact experienced since before the onset of NC.

Progressive worsening: Progressive worsening of pain, decreased mobility, decreased social and recreational participation, or negative psychological impact (i.e. continued worsening after an earlier worsening).

Improvements remained same: Improvements experienced at T1 (compared to entry into trial) or at T2 (compared to T1) in pain, mobility, social and recreational participation, or psychological impact were maintained.

Progressive improvements: Progressive improvements in pain, mobility, social and recreational participation, or psychological impact (i.e. continued gain on an earlier improvement).

Transient change pattern 1: Initial improvements between T1 & T2 then reversed to T1 (Baseline) status at T3.

Transient change pattern 2: Initial improvement between T1 & T2 & then worsening between T2 & T3, to worse than T1

Transient change pattern 3: Initial worsening between T1 & T2 & then improving between T2 & T3

Delayed improvement: Pain, mobility, social and recreational participation, or psychological impact improved at T3 only.

Lack of sufficient information (at least 2 time points) to describe the change process.

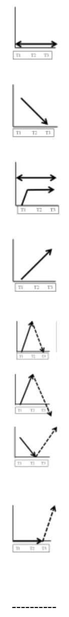

For peer review only - http://bmjopen.bmj.com/site/about/guidelines.xhtml

Progressive improvement in at least one domain was experienced by a total of 13 participants. Trajectories of no change, early improvements that remained the same and transient changes predominated. Of the 30 participants, 16 experienced no improvement in at least one domain. Improvement in pain was experienced by 16 participants. All 15 participants who experienced improved social and recreational participation also reported improvements in other domains (although for one the improvement in another domain was after initial worsening).

The trajectories in any one domain are not an indication of the amount or nature of change so the same trajectory might be experienced very differently. For example, participant 24 and participant 50 both showed progressive improvement in mobility, but one was able to walk much further than the other:

> *And as I said, I can go out and I can go off to different parts of the shops, or garden centres, we like looking round those.* (24, T3)

> *I am able to get up and walk without any aids around it* [home] *now* (50, T3).

In the domain of psychological impact, participant 04 and 02 continued from T1 to T3 with negative but different experiences. The first experienced frustration as he had to rely on his wife to do his self-care activities:

> *I can't get myself dressed, I can't do the things that I would normally do or anybody else would normally do for themselves. I need a hand; I need help with that, which I do get obviously, with [wife]. Again, that's frustrating because she's got things to do and I don't feel I can keep saying to her, oh can you just help me with this, can you help me with that?* (04, T3)

The second describes fear of damaging his back with certain activities:

> *But I don't do gardening like I used to do before. For the fear of damaging or hurting my back, and things like that. Because I tried that in the past, and once I start doing something then I get carried away, and the next two days then I have to suffer, so I try and avoid it* (02, T3).

In the domain of social and recreational participation, participants 07 and 24 experienced progressive improvements over time but with different reach. The first was prepared to extend her activities:

> *But wherever I go, if I go on holiday as well, I never say no, that I can't do (some)thing* (07 T3)

The second was positive about her ability to undertake small tasks that helped her feel she was participating equally with others. She described social outings with her friends:

> *I can carry a tray with drinks on now. I can take my turn, I can get in the queue, where it always used to be 'you get the table, I'll go* (24, T3).

When placing participants adjacent to other participants based on the similarity of trajectory, we were making choices about which trajectories to prioritise. Different choices resulted in a different ordering of participants. For example, we could place together participants with two or three domains with the same trajectory and not worry about the trajectory of the other domain(s), or we could place together participants with the same number of trajectories of a certain type, irrespective of which domains they were. However, participants tended to

For peer review only - http://bmjopen.bmj.com/site/about/guidelines.xhtml

remain towards one end or the other of the table. Table 2 presents one way of ordering the participants on which the team could agree.

Our visual impression was there were five main patterns of trajectories which we identified as our trajectory categories, although the boundaries of these are not clear cut. They were: category 1 (n=10) participants with progressive improvements in at least two domains; category 2 (n=5) paticipants with initial and maintained or delayed improvements in at least two domains; category 3 (n=5) participants with discordant trajectories – where at least one trajectory was to some extent in the opposite direction to at least one other trajectory (the opposite direction might be transient); category 4 (n=3) participants with transient change (in the same direction) in at least two domains; category 5 (n=7 participants with no change in at least two domains. We illustrate the trajectories for one participant at each time point with quotations from their data in **Table 3** and for one participant from each trajectory at T3 in **Table 4**.

For peer review only

Table 3: Illustrative quotes for one participant [Category 3] for all domains and time points

ID:04	Pain	Mobility and ADL	Psychological impact	Social and recreational participation
T1	*'And then, umm, there's pain if I get up, there's pain if I walk, there's pain sitting down, there's pain standing up, there's pain reaching out… it's a constant pain. 24/7 I'm in pain'*	*'I'm inactive, I can't do anything without being in pain…'*	*' So I started to get depressed then, because I couldn't... I can't do anything and I couldn't do anything and I, I just sort of thought, 'Well what's the point, what is the point of me being here when I can't do anything?' This is it, I'm just sitting around, I'm doing nothing, I'm no good …'*	*'Oh I love to see the grandkids but, again, I can't do anything with them, I can't do nothing with the grandkids because, again, the... My grandson's into football now and I feel there's a lot I could teach him with the, with the game and I can't.'*
T2	*'Not really, it's* [Pain] *there the majority of the time.'*	*'Well every day, every day. I mean we're either out shopping or that, I don't think there's a day goes by where I'm not doing some sort of walking.'*	*'I'm in a situation where I'm still depressed over it because obviously I'm not … I didn't expect a lot from it, I suppose in my mind I thought it might be, I might be having a lot more movements and things like that, which haven't worked out.'*	*'Socialising I don't do. I'm not a socialiser anyway. All them days … since the football finished and everything I'm not, I'm not one for going out and that anyway. As I said, sometimes it's not worth the effort to get ready and. Getting washed and changed and then getting out like that, so much is involved with it, sometimes it's not worth doing it'*
T3	*'Well I'm still in a lot of pain….*	*'I feel more confident when I'm in the house. I mean while the weather's like this I won't budge out at all, I won't venture out at all, there's no point….Obviously, when it's really severe, I can't get myself dressed, I can't do the things that I would normally do or anybody else would normally do for themselves'*	*'I mean, I keep bringing the mental side of it because I think everything that goes on does affect you mentally. It's very hard not to get away from a lot of things mentally'*	*'I said, my one grandchild is playing football himself now and I can't, there's no way I can go with him and help him or be involved in his, in coaching with him or teaching him anything or just having a kick around with him. I can't do that, so I feel I'm missing out on all that.'*
**Trajectory**	No change	Initial improvements between T1 & T2 then reversed to T1 (Baseline) status at T3	No change	Progressive worsening

For peer review only - http://bmjopen.bmj.com/site/about/guidelines.xhtml

Table 4: Illustrative quotes of participants from each trajectory category at T3

Participants from trajectory categories	Pain	Mobility and ADL	Psychological impact	Social and recreational participation
**Category 1** ID:08 White British Male, 70 years	*'But in terms of my back pain and legs, although I still have a little and last night it was particularly uncomfortable, most of the time it actually is fine, and I feel a lot better than I did last year.'*	*'If I go for a walk, for example, with my wife, I can probably do quite a long-ish walk, probably three to five miles, which I couldn't do 12 months ago.'*	*'But, for example, to give you a clue of how more confident I am, we are going away for five days on Sunday to [city] to do some sightseeing, some walking, and some photography. So I feel confident enough to be able to go and do that.'*	*'… we went down to* [Town] *for a week* [Holidays]*, back in November and the apartment we had was about a mile and half out of the town centre. So we walked that both ways, in and out, most days, and I coped with that quite well.'*
Category 2 ID:31 White British Female, 81 years	*'Oh it is better. It's under control, as much as I can get it under control, with the help of the Gabapentin, which, I know it's another drug to be on but if it works then.'*	*'I walk down the stairs and up the stairs, rather than use the [stair lift]. And I walk up and down the hall quite a bit, I do an exercise where I just walk up, turn around and come back, I do that about ten times. Particularly if it's a wet day and I can't get out.'*	*'Well, I'm walking better. Whereas before I was getting frightened to walk, because I was in so much pain.'*	*'Well, I tend to go in* [Gardening] *for a short while. Once my back starts hurting, I come back in and sit down. I don't overdo it generally.'*
**Category 3** ID:04 White British Male, 65 years	*"Well I'm still in a lot of pain, but not as much as I was last week. It's sort of calmed down a little bit now."*	*"Well, I don't cook for myself or anything like that. I mean, I can't mess around with things like that. Obviously, when it's really severe, I can't get myself dressed, I can't do the things that I would normally do or anybody else would normally do for themselves."*	*"I mean, I keep bringing the mental side of it because I think everything that goes on does affect you mentally. It's very hard not to get away from a lot of things mentally."*	*"Normally I miss out on a lot of things because of sitting somewhere for too long or something because this again causes discomfort. So rather than sit there feeling miserable I'd rather not go."*
**Category 4** ID:14 White British Female 77 years	*'At the moment I think I'm at a bad stage, because I've got so many things going on … I've got the back pain. I've got the neck pain. I've got the knee, which comes out of joint. So it's not a pretty sight really.'*	*'But I still make myself, when we've got a good day, not today like it is now, but if it's a good sunny day I will still try to go out and do about ten minutes, fifteen minutes' walk.'*	*'I was doing very well and very eager to get better or help myself somewhat … So I've got all these things* [other musculoskeletal issues] *going at once. Which has dragged me down a bit, there's no doubt about it, it's made me quite depressed.'*	*'It's obviously; it's something I'm not used to. I'm used to going everywhere and very, sort of, doing exercises and yoga classes and everything else. And it's getting worse, because it's stopped, it's stopping, everything is going wrong.'*
**Category 5** ID:11 White British Male 72 years	*Once I've got my legs, once I'm moving a bit, it does seem to ease off, I'm not so bad. But if I sit down, then once I get up again it's back to square one; you're still in pain and tingling.'*	*'So it's hard for me to put, you know, for me to actually sit still all day. In the last week, I've watched more telly than I've watched in 12 months. So you can tell, like, the situation. I've never watched so much, I've got square eyes.'*	*'Well at the moment I'm more anxious than ever with what I've got now. So I'm still a bit anxious, well even before this, before this I was still anxious. I was quite anxious really, you know, because I wanted to do things.'*	*'And so it's really, my lifestyle really, to me, it's gone to nil from what I - I'd be out doing everything sort of thing, nine o'clock, ten o'clock at night time gone by, til it got dark, pottering about, cutting the lawn, doing the lawn, or doing the neighbour's if they couldn't do it, elderly people I'd cut theirs for them. So it's, to say, like, you've come to, not nil as such, but you've really, it's a total cut off …'*

This categorisation was developed from one way of ordering the participants (see above). It was therefore expected that some participants could be in more than one category (for example participant 34 could be in category 2 or 5).

We chose to define category 3, discordant trajectories, as trajectories going in opposite directions (improving/worsening). Arguably, a no change trajectory alongside a transient change or an improving/worsening trajectory is also discordant. All but one of the 10 participants in categories 4 and 5 have this latter combination of trajectories, and four of the 15 participants in categories 1 and 2.

Of the 15 participants who experienced improvement in social and recreational participation, ten experienced improvements in all other domains and were in categories 1 and 2. The

For peer review only - http://bmjopen.bmj.com/site/about/guidelines.xhtml

remaining five participants (24, 43, 38, 41 and 34) did not experience improvement in all other domains and were from across all categories except category 4.

Analysis step 4: nature of the trajectories

Co-presence of domain trajectories and perceived interaction

We identified ten types of co-presence of the trajectories (**Table 5**). The most common presentation was Type 1 where positive trajectories were co-present for all domains. Our data suggests improvement in one domain often led to improvement in another and so on (see illustrative quotes Table 5). Type 2 (n=3) was similar except those improvements in pain, mobility and psychological were not co-present with improvements in social and recreational participation. The reasons for this were very different and specific to each individual: participant 09 was a 79-year-old farmer who was unable to get back to his heavy farm work, participant 37 was an 88-year-old who did not get back to going on group trips which involved walking as he did not want to hold up the others and 01 was an 81 year old who at baseline had already managed to return to Tai chi and her voluntary work and maintained this. All the participants of type 1 and 2 were in trajectory groups 1 and 2 (improvement trajectories) except participant 38 who was in trajectory group 3 as they had transient decline before improvement in two domains. This participant gained mobility and social and recreational participation through learning to effectively use a walking aid.

For peer review only - http://bmjopen.bmj.com/site/about/guidelines.xhtml

Table 5: Co-presence of trajectories (⇑ improving; ⇓ worsening; ⇔ no change) and illustrations of how they interact (or not)

Co-present trajectories of biopsychosocial domains	Participant ID	Participants quotes illustrating interaction between co-present trajectories	Trajectory Category (see table 2)
1) ⇑Pain, Mobility, Psychological impact, Participation	30, 31, 07,08, 25, 38, 05, 26, 57, 47, 50	*'When you first saw me, I was in a bit of pain, and everything was a struggle. But now, I take everything in my stride now ... I think at least 75% I feel better in myself* **[Pain]**… *Oh I'm quite satisfied* [with walking] *at the moment. As I say, a couple of miles is easy now, when I go out shopping or anything like that* **[Mobility & ADL]…***And then as I say, Wednesdays and Thursdays I go out to the club, have a drink, and socialise with my friends. And Saturday night I go out, get on the dance floor if I feel up to it, for half an hour or something like that* **[Participation]**…*But as far as, it* [Neurogenic claudication] *doesn't stop me doing anything I want to do'* **[Psychological impact]** (30, T3)	1 and 2<br>Improving
2) ⇑Pain, Mobility, Psychological impact ⇔ Participation	01, 09, 37	*'I really haven't had any problems with my back* (37, T2) **[Pain]**…*I don't think there is any problem with my general health…I think I am walking better than I was when I started this scheme…I suppose I have really not a lot of problem, I can walk the distances that I need to now, I don't want big distance… I think it* [Walking uphill] *is easier…it has been easier before when I first met you, if I walked uphill, I felt exhausted…now I don't* **[Mobility & ADL]**…*I design my life around my abilities* **[Psychological impact]**…*Not a lot, to be fair* [impact of condition on lifestyle]…*I keep myself involved that is my objective to keep myself occupied…To be fair, I think I work my physical life to the limitations I have got and being my age… I don't suppose I can say it* [General lifestyle] *altered other than doing exercises in the morning'* **[Participation]** (37, T3)	1 and 2<br>Improving
3) ⇔ Pain ⇑ Mobility, Psychological impact, Participation.	43, 24	*'No, just through the night* **[Pain]**… *I'm getting around fine…I just feel good* **[Mobility & ADL]**. *Makes me feel good, it gives me confidence. You see I can do it, and now I know I can do it* **[Psychological impact]**… *I can carry a tray with drinks on now. I can take my turn, I can get in the queue, where it always used to be 'you get the table, I'll go'* **[Participation]** (24, T3)	1 and 2<br>Improving
4) ⇓ Pain, Mobility, Psychological impact, Participation (except 41 where improved Participation gained by remained the same)	03, 11, 33, 41, 28	*'… once I've got my legs, once I'm moving a bit, it does seem to ease off, I'm not so bad. But if I sit down, then once I get up again it's back to square one; you're still in pain and tingling* **[Pain]**…. *'I'm really basically, not doing nil, but you know, walking up and down the garden and walking, not really doing to what I normally do. It's really halved* **[Mobility & ADL]**…*Well at the moment I'm more anxious than ever with what I've got now. So I'm still a bit anxious, well even before this, before this I was still anxious. I was quite anxious really, you know, because I wanted to do things* **[Psychological impact]**…… *And so it's really, my lifestyle really, to me, it's gone to nil from what I - I'd be out doing everything sort of thing, nine o'clock, ten o'clock at night time gone by, till it got dark, pottering about, cutting the lawn, doing the lawn, or doing the neighbour's if they couldn't do it, elderly people I'd cut theirs for them. So it's, to say, like, you've come to, not nil as such, but you've really, it's a total cut off'* **[Participation]** (11, T3)	5<br>No change
5) ⇔ Pain ⇓ Mobility, Participation (or not mentioned). ⇑ Psychological impact	53, 06,	*'Still I am in some difficulty and problem* **[Pain]**… *but….honestly I just carry on with my life* **[Psychological impact]**…. *…It* **[Pain]** *is not too bad at the moment….To be honest, I don't do anything* **[Mobility & ADL]** *at all…Some days…bad days which you know you just wonder what else you can do… Housework otherwise, I take my time. I don't try to push myself to you know everything I need to suppose one time...Little at a time* **[Mobility & ADL]**…*I don't worry about it I just try on and carry on with my life* **[Psychological impact]**…. *I do that* [Shopping] *not alone but always have somebody with me'* **[Participation]** (06, T3)	5<br>No change
6) ⇔ Pain ⇓ Mobility ⇑ Psychological impact, Participation	34	*'Could* **[Pain]** *be better. I can feel my sciatic nerve all the time* **[Pain]**… *…but it didn't really bother me, you know* **[Psychological impact]** …. *I can't walk as quick as I used to and I can't do steps…I am walking normally but the smaller steps what I used to do… My legs hurt when I start moving around…there are certain movements I just be careful with…but other than that, fine* **[Mobility & ADL]**, *I am doing my garden. My back doesn't like it very often…You know I come in, sit down, have a cup of tea and go back again. I go to my gardening club...Tuesday I see my friend as usual…put the world right and have a cup of coffee…today I had been to school….so listen to the children reading and now being given an award today* **[Participation]**……*You know you just have to get on with it, don't you? I can't go anywhere, I feel shattered, but you still have to do don't you? If you got to do something, you got to do it'* **[Psychological impact]** (34, T3)	5<br>No change

For peer review only

7) ⇑ Pain ⇓ Mobility, Psychological impact ⇔ Participation	02	*'But my back pain, the constant pain and the severe pain that I used to have is no longer there at the moment, so I'm happy to say that. However, it is not completely gone, and I get days, every now and again, when the pain comes back* ***[Pain]****.... What happens is then the pain starts, not while I'm walking but after I have done my walk and I sit down. And the more I walk, the more severe the pain is when I sit down* **[Mobility & ADL]***... 'It's not the ideal situation, because it makes me little bit home bound and I cannot travel as freely as I would like to, long distance and things like that* **[Participation]***...this summer I had high hopes of doing a lot of other things, to improve my back and things like that, but with this balloon sticking from my stomach I can't even go swimming or cycling... Cycling might be a bit too strenuous. Although I have been told that people do go swimming with this type of tube, but I don't want to take that chance'* **[Psychological impact]** (02, T3)	3 Discordant
8) ⇑Pain ⇓ Mobility ⇔ Participation ⇑ Psychological impact before T3 spinal fusion	15	*'I got no pain at all now…I am pain free in my back* **[Pain]***… …I got a walking frame that I can with difficulty walk around the house* **[Mobility & ADL]** *I say 'with difficulty' because at times my right leg just tend to buckle slightly, because the right leg is by far the worst one…it was by far the worst affected it does tend to buckle …and so either my wife or the carer when they come, they escort me, it is they don't have to do anything, they are there as a safety precaution* **[Mobility & ADL]***.... All in all, it's been a fairly traumatic sort of time…I keep myself in good spirits…well its happened what is the point of being miserable about it, I keep myself in good spirits and carry on'* **[Psychological impact]***…Not doing any physical activity, obviously my body is not getting tired….Obviously at the moment, is the rest and recuperation time…'* **[Participation]** (15, T3)	3 Discordant
9) Pain predominantly from other MSK problem. ⇓ Mobility, Psychological impact, Participation (or not mentioned)	04, 14, 39	*'At the moment I think I'm at a bad stage, because I've got so many things going on ... I've got the back pain. I've got the neck pain. I've got the knee, which comes out of joint. So it's not a pretty sight really* **[Pain]***.... I'm used to going everywhere and very, sort of, doing exercises and yoga classes and everything else. And it's getting worse, because it's stopped, it's stopping, everything is going wrong.... So I've got all these things going at once* **[Mobility & ADL and Participation]***. Which has dragged me down a bit, there's no doubt about it, it's made me quite depressed'* **[Psychological impact]** (14, T3)	4 Transient change
10) Pain predominantly from other MSK problem. ⇓Mobility, Participation (or not mentioned) ⇑Psychological impact	32	*'Terrible* **[Pain]** *at the moment…Such a lot of pain in my knee then I have got pain in my right buttock which is more like… it is the sciatic nerve is trapped and I can't sit for longer I have to keep putting the pillow under or keep lying down…Lying down flat makes my back worse...its* [Hip pain] *been on and off a couple of years…some days its* [Back pain] *been worse, I can't get washed and dressed… Its pain everywhere…there is nothing you can do till you get repaired* **[Psychological impact]***…I can't do any housework or anything because I can't stand without hanging on to my trolley… Next week I am starting meals on wheels…It is non-existent really* [General lifestyle] *I could do a few things but not now...it is walking* [reason] *I wouldn't want to go far because it gets too painful, I walk to the car'* **[Mobility & ADL and Participation]** (32, T3)	5 No change

ADL: Activities of Daily Living; MSK: Musculoskeletal

In the group with co-presence type 3, where there was no improvement in pain but improvement in all other domains, participant 43 used pain relief for the persisting pain enabling other domains to improve even though he was not keen on taking painkillers, and 24 experienced pain only at night. Both were in trajectory groups 1 or 2.

In type 4 (n=5) all domains were worsening. This was the second most common type of co-presentation and mirror image of that observed in Type 1. Worsening in one domain had a negative impact on the other domains. All participants were in trajectory group 5 (no change in at least 2 domains) except participants 33 and 41. The former experienced transient improvement in two domains but then worsened, so was in trajectory group 4. The latter was in trajectory group 3 where there was discordance. This participant had worsening pain and mobility which she had found depressing but by T3 she saw the pain, although severe, as manageable. The level of mobility required for her to maintain most of her

For peer review only - http://bmjopen.bmj.com/site/about/guidelines.xhtml

recreational and social interests was relatively limited and she could manage this through planning, but she had difficulty with activities involving walking in groups.

Type 5 (n=2) was similar to type 4 and both participants were in trajectory group 5 but there was improvement in psychological impact. Participant 53 had specifically addressed his depression that had resulted from the NC symptoms and 06 had adapted to the pain. However, in the timeframe of the study the reported improved psychological impact did not influence the other domains.

Participant 34 with co-presence type 6 where pain did not improve and mobility deteriorated (trajectory group 5) similarly maintained positive psychological outlook but was also able to maintain social and recreational engagement despite the pain and loss of mobility.

Participant 02 (co-presence type 7) and 15 (co-presence type 8) were both in trajectory group 3 where there was discordance. The former remained envious of those who could walk faster than he could which impacted negatively on his psychological wellbeing and would have liked to be able undertake long distance travel which was limited by his pain. Participant 15 had spinal surgery between T2 and T3 which raised his hopes of improvement although had not yet experienced improvement.

Co-presence types 9 and 10 were similar to types 4 and 5 respectively but the pain was not from NC but from other musculoskeletal problems.

We were particularly interested in those who still improved in social and recreational participation without improvement in all other domains. This was experienced by four participants for a variety of reasons highlighting those ongoing impairments across other domains does not necessarily impede improvement in participation. For 24 the pain was only at night, for 43 the pain could be controlled, and for 34 and 41 it was their positive psychological outlook.

Turning points

Thirteen participants did not mention a major change in their health. Twelve described major change in musculoskeletal problems: four described notable improvement or worsening of their pain and mobility, one had a fall that impacted wellbeing, four described worsening of joint problems (hip, knee, and neck), and four had back surgery during the data collection year. Four participants developed other major health issues (emergency abdominal surgery, Addison's disease, chronic obstructive airways disease, insertion external feeding tube). From the data for these latter participants, we were unable to detect any interaction between their trajectory and these health events.

Totality of change

There were many similarities between participants from across the different trajectories. The majority of participants lived with a partner with a total of 9 living alone. The majority of participants (from all categories) had a positive outlook on aging and wanted to be active, '*…Age is just a*

For peer review only - http://bmjopen.bmj.com/site/about/guidelines.xhtml

number, for me' (08, T1, 70 years). A few either had an acceptance of aging or had negative attitudes, *'You'd think I was 95 the way I'm talking to you'* (11, T2, 70 years). Most participants (from all categories) were using some form of mobility aids ranging from walking stick, wheeled walker and rollator, Nordic poles, Zimmer frame, and mobility scooters. A very few (all categories) mentioned they didn't want to become dependent on mobility aids or look older, *'I don't like them [laughs]. Vanity. I just suppose … It looks like you are an old man you know, or a disabled person'* (02, T1). In all categories, good weather/summer season enabled walking and outdoor activities and many reported limited activity during winter due to difficulties such as lack of confidence in walking when wet/icy and difficulty finding places to sit down. All participants reported a variety of self-made, convenient strategies for coping with their NC in at least one of their interviews. Participants predominantly used active, problem-focused strategies rather than passive ones such as avoiding painful activities. For example, they used solution-based strategies such as sitting/resting between walks or household chores, driving to local shops instead of walking, leaning on a shopping trolley to walk around, using a light-weight vacuum cleaner, and accepting help from family members with shopping and gardening. Participants who regularly (mostly daily, one 2-3 times a week) engaged in exercises specifically for their NC were found in all trajectory groups.

Discussion

For each person living with NC, we were able to identify the pattern of change at each of the three time points for each of the biopsychosocial domains of pain, mobility, psychological impact and social participation. By looking across these patterns we were able to identify trajectories over time for each domain. Participants in all trajectory categories had many attributes in common including attitudes to aging and in seeking strategies to enable themselves to function optimally.

Although there were participants where improvement occurred across all domains or there was no change across all domains, many participants reported discordance with change going in different directions in different domains, or there was no change in some domains alongside change in other domains. Published quantitative evidence has demonstrated within domain discordance: for mobility, discordance between walking capacity and actual walking (32, 33). With our qualitative data, we were able to illustrate interaction between domains, for example, pain affecting mobility, with negative psychological impact and social withdrawal and vice versa. This negative cycle might explain the within domain discordance for mobility. However, we were also able to illustrate how, for example, improvement in psychological impact or social and recreational interaction happened despite pain or mobility problems.

For ten out of fourteen participants reporting improvement in social and recreational participation, this occurred alongside improvement in all other domains. This is the best scenario and the aim of interventions for people with NC. Remaining participants reported dealing in some way specifically with their pain, mobility or psychological impact which still enabled them to improve social and recreational participation without

For peer review only - http://bmjopen.bmj.com/site/about/guidelines.xhtml

improvement in all domains. This suggests that it is possible to identify strategies to increase participation even if other domains are resistant to change. This is particularly important when considering the impact of pain. Pain is particularly problematic for this patient group and effective treatments for reducing the pain associated with NC are limited (34). For the participants where improvement in social and recreational participation was not achieved when it might be expected the reasons were: very high expectations of what for them counted as participation; did not want to cause a problem for others; there no loss of engagement to be regained. The other group warranting further consideration are those reporting worsening across all the domains and what can be done to break this perpetuating negative cycle.

Categorising and understanding change over time

Our study extends the limited current longitudinal qualitative evidence on living with back pain. We found a greater variety of trajectories than in a published study with just eight participants (see background) (12) as, in contrast to this study, we found examples of change in one domain despite no change in others as well as examples of reinforcement of change across domains. The earlier study collected data at longer time intervals (12 months over 2 years). This may have resulted in less detail about change between data points than in our study resulting in the perception of smoother trajectories. In our past qualitative study (22) which included 15 participants living with chronic back pain (excluding NC) interviewed three times in two years, we developed the following categories of participant's current experience of their back pain: past reminders (similar to fear avoidant behaviour), stuck and struggling, becalmed, and submerged. These categories describe the overall state of the participants rather than interactions that resulted in these states. Of the 15 participants, 12 changed category during the 2 years. Most of the participants started as stuck and struggling and moved to past reminders or becalmed, trajectories similar to our current study participants in categories 1 and 2. Two participants remained stuck and struggling/submerged, a similar trajectory to the participants at the bottom of our Table 2 (trajectories of no change/worsening). Our past study included cross-sectional data only from people living with diabetes (22) who were categorised in a similar way to those with back pain.

Trajectories have been identified in qualitative studies of people living with long-term conditions other than back pain where longitudinal data has been collected. In a study of 22 participants, data was collected 15 months post stroke and over the subsequent 4 months. Four different recovery trajectories were evident: (a) meaningful recovery, (b) cycles of recovery and decline, (c) ongoing disruption, (d) gradual, ongoing decline (35). The first (a) is similar to our progressive improvement trajectory, (b) may be similar to our discordant trajectories, (c) and (d) have some similarity to our category of no change; the difference may be due to the natural history of the NC where NC only worsens for a few (6). However, trajectories identified depend on the focus of analysis. A longitudinal interview study with 21 participants diagnosed with a chronic illness (e.g., diabetes, rheumatoid arthritis) focused on illness self-management (36). It was found participants go through the following phases: seeking effective self-management strategies, considering costs and benefits, creating routines and plans of action, and negotiating self-management that fits one's life. In our study we found all these strategies being used but our analysis focus was not self-management.

For peer review only - http://bmjopen.bmj.com/site/about/guidelines.xhtml

Categorising to predict outcome and complex interaction

We found many similarities between the participants in the 5 categories of trajectories and a lack of features that could be used to identify members of a particular trajectory which could be used to predict outcome. Many studies on back pain have analysed quantitative data using methods for categorising such as latent class analysis, seeking categories of patients that would allow matching of interventions to patient groups (37-39). Study participants can be categorised into clinically meaningful groups and category membership may correlate with outcome (40). However, interaction between category membership and intervention is difficult to demonstrate (38). A longitudinal study of spinal stenosis found factors predictive of subjective improvement, but none were modifiable (41). If improved social and recreational participation is our goal, our study would suggest that this is likely to be co-present with and interact with improvement across the domains of pain, mobility and psychological impact and not improve when there is no co-presence of improvement across the other domains. This interaction between domains is like that described in a cross-sectional interview study with people living with NC (5). Although our data suggests that for some participants improvement in one domain leads to improvement in another domain, this interaction is in the timeframe of hour-to-hour or day-to-day or week-to-week as occurs in complex systems (42, 43). This, what we have called co-presence, is different from the prediction of outcome over many months or years that is sought in the studies described above. However, our study also provides examples of participants where one factor did prevent or enable improvement, but this was a relatively small number of participants and the attributes varied. We suggest that seeking individual level factors that predict of outcome for people living with NC is unlikely to be helpful. However, we need to remain alert to external determinants of outcome. For example, in a US study, type of health insurance influenced rate of improvement for people with low back pain (44).

Strengths and limitations

We collected a relatively large qualitative data set with higher-than-expected participation rates in later interviews. This may have been because the repeat encounters generated a relationship of trust between researcher and participant (45). Although there are differences when interviews are conducted by telephone rather than face to face (46) our telephone interviews provided rich data for our analysis, perhaps helped by the first interview being face-to face

At each interview we focused on how the participant was at the time of the interview. However, in interviews people naturally tell stories and these stories include accounts of change that has occurred (47). What was current was sometimes difficult to disentangle and we may have interpreted later data differently given our understanding of the participant from earlier interviews (23).The data we included in the current analysis was from participants providing three interviews of rich data so they may differ from those in our study providing less data. As we proceeded through data analysis, we chose our path based on our interest in the complex nature of health, the lived experience of conditions such as NC and successful aging. Alternative data analysis paths through this data set are possible. Analysis could be tested further by interrogating the remaining unanalysed data. Reduction of data to mini statements loses richness and nuances in data but we retained the ability to go back directly to the framework and the data for fuller summaries and illustrative quotes. Data reduction enables comparison across the whole data set.

For peer review only - http://bmjopen.bmj.com/site/about/guidelines.xhtml

Although a longitudinal study, follow up was only for 12 months, a relatively short time. NC symptoms change over time. Some of our participants had already started to experience improvement in their symptoms at the start of the study. This is in line with findings of a cohort study of people with moderate NC(6).

Participants could be categorised in different ways and there was overlap between categories. This is a common finding when categorising people in both qualitative studies (22) and quantitative studies (37).

Implications for clinical practice

There may be benefit in those providing therapy for NC patients to identify the trajectory of their patient in order to tailor treatment. This requires careful listening as changes may be quite small so hard to discern. Although it is useful to categorise patients as we have done in this study, these categories are just useful labels to think with and guide discussions with patients. As therapists know, patient presentation can often seem messy. Having the categories of trajectory and how they potentially interact in mind may help tease out what is going on for people and identify where there is opportunity to reinforce positive change or modulate deterioration, but we need to beware of shoehorning people into categories as we could miss important changes in trajectory in one or more domains. Our study suggests therapists, while following clinical guidance for patients with NC, also need to individually tailor their interventions for patients to optimise participation in social and recreation activities that are fundamental to healthy aging.

For peer review only - http://bmjopen.bmj.com/site/about/guidelines.xhtml

References

1. Williamson E, Sanchez Santos MT, Morris A, Garrett A, Conway O, Boniface G, et al. The Prevalence of Back and Leg Pain and the Cross-sectional Association With Adverse Health Outcomes in Community Dwelling Older Adults in England. Spine (Phila Pa 1976). 2021;46(1):54-61.
2. Ishimoto Y, Yoshimura N, Muraki S, Yamada H, Nagata K, Hashizume H, et al. Prevalence of symptomatic lumbar spinal stenosis and its association with physical performance in a population-based cohort in Japan: the Wakayama Spine Study. Osteoarthritis Cartilage. 2012;20(10):1103-8.
3. Suri P RJ, Kalichman L, Katz JN. . Does this older adult with lower extremity pain have the clinical syndrome of lumbar spinal stenosis? JAMA. 2010;Dec 15;304(23):2628-36.
4. Lin SI, Lin RM, Huang LW. Disability in patients with degenerative lumbar spinal stenosis. Arch Phys Med Rehabil. 2006;87(9):1250-6.
5. Ammendolia C, Schneider M, Williams K, Zickmund S, Hamm M, Stuber K, et al. The physical and psychological impact of neurogenic claudication: the patients' perspectives. J Can Chiropr Assoc. 2017;61(1):18-31.
6. Wessberg P, Frennered K. Central lumbar spinal stenosis: natural history of non-surgical patients. Eur Spine J. 2017;26(10):2536-42.
7. Rowe JW, Kahn RL. Successful aging. Gerontologist. 1997;37(4):433-40.
8. Katz S CT. Critical perspectives on successful aging: Does it "appeal more than it illuminates"? The Gerontologist. 2015 Feb 1(1):26-33.
9. Foster L WA. Active and Successful Aging: A European Policy Perspective. The Gerontologist. 2014;Feb 1(1):83-90.
10. Martinson M BC. Successful aging and its discontents: A systematic review of the social gerontology literature. The gerontologist. 2015;Feb 1(1):58-69.
11. Snelgrove S LC. Living with chronic low back pain: a metasynthesis of qualitative research. Chronic illness. 2013;Dec: 9(4):283-301.
12. Snelgrove S ES, Liossi C. A longitudinal study of patients' experiences of chronic low back pain using interpretative phenomenological analysis: changes and consistencies. Psychology & health. 2013;Feb 1(2):121-38.
13. Kelley-Moore JA SJ, Kahana E, Kahana B. When do older adults become "disabled"? Social and health antecedents of perceived disability in a panel study of the oldest old. Journal of health and social behavior. 2006;Jun: 47(2):126-41.
14. Corbin J. Accompaniments of chronic illness: Changes in body, self, biography, and biographical time Res Sociol Health Care. 1987;6:249-81.
15. Bury M. Chronic illness as biographical disruption. Sociology of health & illness. 1982;4(2):167-82.
16. Anjum RL CS, Rocca E. Rethinking causality, complexity and evidence for the unique patient: a CauseHealth Resource for healthcare professionals and the clinical encounter. 2020.
17. Cilliers P. Difference, Identity and Complexity. In: Cilliers P, Preiser R, eds. Complexity, Difference and Identity Issues in Business Ethics. Dordrecht: Springer; 2010.
18. Griffiths F BJ, Byrne D, Crabtree BF, Dowrick C, Gunn J, Kokanovic R, Lamb S, Lindenmeyer A, Parchman M, Reis S. . Developing evidence for how to tailor medical interventions for the individual patient. Qualitative Health Research. 2010;Dec: 20(12):1629-41.

For peer review only - http://bmjopen.bmj.com/site/about/guidelines.xhtml

19. Weller S ER, Jamieson L, Davidson E Analysing large volumes of complex qualitative data; reflections from a group of international experts. 2019.
20. Ward L, Williamson E, Hansen Z, French DP, Boniface G, Rogers D, et al. Development and delivery of the BOOST (Better Outcomes for Older adults with Spinal Trouble) intervention for older adults with neurogenic claudication. Physiotherapy. 2019;105(2):262-74.
21. Williamson E, Ward L, Vadher K, Dutton SJ, Parker B, Petrou S, et al. Better Outcomes for Older people with Spinal Trouble (BOOST) Trial: a randomised controlled trial of a combined physical and psychological intervention for older adults with neurogenic claudication, a protocol. BMJ Open. 2018;8(10):e022205.
22. Griffiths FE, Lindenmeyer A, Borkan J, Donner Banzhoff N, Lamb S, Parchman M, et al. Case typologies, chronic illness and primary health care. J Eval Clin Pract. 2014;20(4):513-21.
23. Lewis J. Analysing qualitative longitudinal research in evaluations. Social Policy and Society. 2007;Oct: 6(4):545-56.
24. Malterud K, Siersma VD, Guassora AD. Sample Size in Qualitative Interview Studies: Guided by Information Power. Qual Health Res. 2016;26(13):1753-60.
25. Gale NK HG, Cameron E, Rashid S, Redwood S. . Using the framework method for the analysis of qualitative data in multi-disciplinary health research. BMC medical research methodology. 2013;Dec: 13(1):1-8.
26. Saldaña. Longitudinal qualitative research: Analyzing change through time: Rowman Altamira; 2003.
27. Grossoehme D, Lipstein E. Analyzing longitudinal qualitative data: the application of trajectory and recurrent cross-sectional approaches. BMC Res Notes. 2016;9:136.
28. Fadyl JK CA, Theadom A, McPherson KM, TBI Experiences Research Group, Wilkinson-Meyers L, Levack W, Starkey N, Kayes N, Feigin V, Harwood M. Optimising qualitative longitudinal analysis: insights from a study of traumatic brain injury recovery and adaptation. Nursing inquiry. 2017;Apr: 24(2).
29. Guest G, Bunce A, Johnson L. How Many Interviews Are Enough?: An Experiment with Data Saturation and Variability Field Methods. 2006;18(59).
30. Sheard L, Marsh C. How to analyse longitudinal data from multiple sources in qualitative health research: the pen portrait analytic technique. BMC medical research methodology. 2019;19(1):1-10.
31. O'Brien BC, Harris IB, Beckman TJ, Reed DA, Cook DA. Standards for reporting qualitative research: a synthesis of recommendations. Acad Med. 2014;89(9):1245-51.
32. Conway J, Tomkins CC, Haig AJ. Walking assessment in people with lumbar spinal stenosis: capacity, performance, and self-report measures. The spine journal : official journal of the North American Spine Society. 2011;11(9):816-23.
33. Smuck M, Muaremi A, Zheng P, Norden J, Sinha A, Hu R, et al. Objective measurement of function following lumbar spinal stenosis decompression reveals improved functional capacity with stagnant real-life physical activity. The spine journal : official journal of the North American Spine Society. 2018;18(1):15-21.

For peer review only - http://bmjopen.bmj.com/site/about/guidelines.xhtml

34. Bussières A, Cancelliere C, Ammendolia C, Comer CM, Al Zoubi F, Châtillon C-E, et al. Non-Surgical Interventions for Lumbar Spinal Stenosis Leading To Neurogenic Claudication: A Clinical Practice Guideline. The journal of pain. 2021.
35. Hawkins RJ, Jowett A, Godfrey M, Mellish K, Young J, Farrin A, et al. Poststroke Trajectories: The Process of Recovery Over the Longer Term Following Stroke. Glob Qual Nurs Res. 2017;4:2333393617730209.
36. Audulv Å, Asplund K, Norbergh K-G. The Integration of Chronic Illness Self-Management. Qual Health Res. 2012;22(3):332-45.
37. Meisingset I VO, Vøllestad NK, Robinson HS, Woodhouse A, Engebretsen KB, Glette M, Øverås CK, Nordstoga AL, Evensen KA, Skarpsno ES. . Novel approach towards musculoskeletal phenotypes. European Journal of Pain. 2020;May: 24(5):921-32.
38. Barons MJ GF, Parsons N, Alba A, Thorogood M, Medley GF, Lamb SE. . Matching patients to an intervention for back pain: classifying patients using a latent class approach. Journal of evaluation in clinical practice. 2014;Aug: 20(4):544-50.
39. Hill JC, Dunn KM, Lewis M, Mullis R, Main CJ, Foster NE, et al. A primary care back pain screening tool: identifying patient subgroups for initial treatment. Arthritis Rheum. 2008;59(5):632-41.
40. Hill JC, Whitehurst DG, Lewis M, Bryan S, Dunn KM, Foster NE, et al. Comparison of stratified primary care management for low back pain with current best practice (STarT Back): a randomised controlled trial. Lancet. 2011;378(9802):1560-71.
41. Matsudaira K, Hara N, Oka H, Kunogi J, Yamazaki T, Takeshita K, et al. Predictive Factors for Subjective Improvement in Lumbar Spinal Stenosis Patients with Nonsurgical Treatment: A 3-Year Prospective Cohort Study. PLoS One. 2016;11(2):e0148584.
42. Gorovitz S, MacIntyre A. Toward a theory of medical fallibility. Hastings Cent Rep. 1975;5(6):13-23.
43. Fox R. Medical Uncertainty Revisited. In: Bendelow G, Carpenter M, Vautier C, et al., eds. Gender, Health and Healing: The public / private divide London: Routledge2002.
44. Katzan IL TN, George SZ, Passek S, Frost F, Stilphen M. . The use of STarT back screening tool to predict functional disability outcomes in patients receiving physical therapy for low back pain. The Spine Journal. 2019;Apr 1(4):645-54.
45. Neale B. What is qualitative longitudinal research? : Bloomsbury Publishing; 2018.
46. Irvine A, Drew P, Sainsbury R. 'Am I not answering your questions properly?' Clarification, adequacy and responsiveness in semi-structured telephone and face-to-face interviews. Qualitative Research. 2013;13(1):87-106.
47. Algilani S, Ostlund-Lagerstrom L, Schoultz I, Brummer RJ, Kihlgren A. Increasing the qualitative understanding of optimal functionality in older adults: a focus group based study. BMC Geriatr. 2016;16:70.

Consent for publication
Not applicable

Competing interests
None

For peer review only - http://bmjopen.bmj.com/site/about/guidelines.xhtml

Funding

This research is funded by the NIHR Programme Grants for Applied Research (reference: PTC-RP-PG-0213-20002). Preparatory work for the programme of research was supported by the Collaboration for Leadership in Applied Health Research and Care Oxford at Oxford Health NHS Foundation Trust. SEL and EW received funding from the Collaboration for Leadership in Applied Health Research and Care Oxford at Oxford Health NHS Foundation Trust and are supported by the NIHR Biomedical Research Centre, Oxford.

Patient and public involvement (PPI)

A group of patient representatives were involved in developing the application for the BOOST study and one member was co-applicant. PPI representatives reviewed patient materials such as consent forms, participant information sheet and the interview schedules. We carried out some practice interviews with PPIs to test and refine the interview schedules prior to data collection. The interview findings were presented to the PPI representatives in a study meeting and were reviewed by the lead PPI representative.

Contributorship statement

Sarah E Lamb (SEL) and Esther Williamson (EW) are health service researchers and physiotherapists and led the BOOST programme of work including this study and the BOOST trial. Frances Griffiths (FG) is a general medical practitioner, medical sociologist and qualitative researcher and led the qualitative study team that included Lesley Ward, a qualitative health researcher, Cynthia Srikesavan (CS), a physiotherapist and a qualitative researcher, and Graham Boniface (GB), a physiotherapy researcher.

All contributed to the qualitative study design, reading data, thematic and comparative analysis, synthesis, interpretation and reporting. LW undertook all interviews. LW and FG reviewed the interview schedules to improve the clarity and flow of the questions. CS worked with FG on analysis with GB providing independent review of coding/summaries. Any discrepancies were resolved through discussion. EW and SEL reviewed each step of the analysis and provided feedback to FG, LW, CS, and GB.

For peer review only - http://bmjopen.bmj.com/site/about/guidelines.xhtml

For peer review only

Better Outcomes for Older People with Spinal Trouble (BOOST)

Semi-structured interview schedules

All three interview schedules will follow a semi-structured format. Interview content from previous interviews will inform the content and sequencing of questions in the following interviews.

For peer review only

First interview

We will refine the schedule for the first interview during the early phase of data collection, including the content and order of the interview questions. The content of the first interviews will be based around, but not limited to, the following set of questions.

1. Please tell me about yourself (household, work/hobbies/family).
2. Please tell me about your general health.
3. Taking a day that was a fairly normal day for you please tell me about what you do in the day, starting with when you get up.
4. Are there activities that you do regularly that you have not mentioned (prompt for hobbies, recreation, visits, holidays, social clubs, volunteering)
5. What does aging/getting older mean to you? Do you think this affects how active/involved you are?
6. Do you feel restricted in what you are able to do? If so, what are you unable to do that you would like to do? How long have you felt restricted?
    - Prompt for how much of the restriction is due to their back/leg pain.
7. How has this restriction affected different aspects of your life?
    - Prompt for physical, mental, and social aspects of their life.
8. Have you had to change the way you do things because of your back/leg pain?
    - Prompt for how they are adapting their physical environment, their behaviour, and their use of technology for mobility or communication.
9. Have you considered other ways you could change the way you do things to enable you to get round some of your restrictions?
    - Prompt for barriers to changing behaviour.
10. What have you done to try to manage the back/leg pain?
11. What exercise do you take/have you previously taken?
    - Prompt for why they take the exercise (physical/social enjoyment), does it help symptoms, are they restricted by symptoms.
    - Prompt for reasons for not exercising/barriers to exercise.

For peer review only - http://bmjopen.bmj.com/site/about/guidelines.xhtml

UNIVERSITY OF OXFORD

BOOST
Better Outcomes for Older people with Spinal Trouble

12. How do you feel about your ability to undertake activities/exercise?
13. What are your hopes/concerns for the physiotherapy that you will receive during the BOOST trial?
14. Do you think the BOOST programme will help with your back and leg problems?
15. The BOOST programme encourages people to be more active or do more walking. How do you feel about being asked to increase your activity and walking?

Second interview

The second interview schedule will be further developed after analysis of approximately 10 transcripts from the first round of interviews, and will be based around, but not limited to , the following set of questions:

1. Have there been any changes in your general health since the last interview?
2. How would you describe your activities now? How does this compare to 6 months ago?
3. How would you describe the problems with your back and legs now? How do these compare to 6 months ago?
4. Thinking again about the things that you would like to do, how does this compare to 6 months ago?
    - Prompt for what has changed, and in what way they changed.
5. Do you think that the physiotherapy you received in the BOOST study made any difference to your back and leg problems?
    - Prompt for whether there are change in the way they view their back/leg pain now; has this changed their habits?
6. What was your experience of the BOOST physiotherapy programme?
7. Has anything else made a difference to your back and leg problems?
8. Have your views on aging and activity changed since taking part in the study?

Third interview

The third interview schedule will be developed after analysis of approximately 10 transcripts from the second round of interviews. The interview schedule will encompass, and build on, the questions presented to participants in the second interview.

For peer review only

Participant ID	Living arrangements	Treatment arm<br>Best Practice Advice (BPA) or BOOST programme	Participant-reported attendance	Desired treatment outcomes	Pain	6 months	12 months	Pain Trajectory	Baseline Mobility & ADL	6 months	12 months	Mobility & ADL Trajectory	Baseline Psycho impact	6 months	12 months	Psychological impact Trajectory	Baseline social & recreational participation	6 months	12 months	Social & recreational participation Trajectory	General health events	Co-morbid conditions	Coping strategies	Views on aging	Use of mobility aids	Feedback from others	Views on treatment/Trial	Were desired outcomes met?	Description of Turning points
02	Lives with spouse	BPA	Attended 2 BPA sessions	Get better (Symptom improvement)	Slight improvement in symptoms on stopping self-prescribed exercises<br>Loses balance after sustained walking<br>Pain on standing > 5 minutes	Ocassional mild pains (no more severe pain)<br>Pain reduced to acceptable level, but did not fully resolve<br>Attributes to doing exercises gently than intensive before; release of trapped nerve<br>Balance slightly improved, still issue<br>Pain on standing > 5 minutes didnt change	Ocassional, but no more severe pains<br>No change in pain (Pain reduced but did not fully resolve)<br>Not sure if due to stopping 1-2 exercises or adding other exercises to regime	Initial reduction in pain that was maintained. Participant had reduced pain upon trial entry.	Mostly housebound; mobilises within house as much as possible.<br>Manages walking upto 20 minutes maximum<br>If weather is good, manages 30 minutes, split into two or three 10 minute walks	Walking duration improved (30 minutes);<br>Balance improved	Mobilises around house & garden<br>Pain still affects walking<br>No change in Walking duration (remained 15-20 minutes)<br>(some seasonal effect- e.g. able to go out more in summer, in winter no place to sit down)<br>Not overly satisfied with walking- 6/10	Initial increase in mobility and back to baseline status	Frustrated depending on others for simple tasks<br>Depressed for not able to contribute to and attend rotary club events<br>Negative psychological impact of getting assistance from spouse at airports	Increased confidence that back pain wont reappear<br>Focuses more on safety while walking<br>Lacks courage to resume biking due to back not feeling strong enough for this activity<br>Fearful of symptoms returning	Participant feels envious of others who can walk faster<br>Avoids certain activities due to fear of pain	Negative impact remained throughout	Difficulty with walking/standing longer made going on holidays difficult<br>Unable to process ideas for book (due to difficulty with walking)<br>Avoids gardening, house maintainence, long travels and rotary club activities	No information	Avoids activities like gardening.<br>Limited ability to travel long-distances	Participation restrictions remained throughout	No information-Throat narrowing having a major social impact- On external feeding tube	Total 2. Hypertension; throat constriction causing swallowing issues	Adopts graded activity approach (breaks 30 minute walk into shorter walks); Pacing (sits down)<br>No new strategies adopted over time.	Current condition is mainly pain -related (90%) & 10% due to aging<br>Priorities being independent than aging<br>Accepts aging and content with overall health<br>Feels less capable than other people of same age	Does not like using aids because it makes them feel old.	No information available	Not sure if symptom improvement is due to release of trapped nerve or BOOST exercises<br>Gently doing exercises would have helped pain<br>BPA no different to previous physiotherapy participant had<br>Not 100% confident whether exercises helped or not (prev exp)<br>Wanted more information but found BPA insufficient in terms of number of sessions, content and followup.<br>Not 100% sure if improvements were due to BOOST or not<br>Sees no potential for further improvements from Physio and would try other treatments-massage & chiropractic<br>BOOST contributed to 20% in improvements<br>BOOST made participant psychologically better, than physical improvements<br>BOOST helped participant to selfmanage instead of opting for surgery.	NO.<br>No considerable pain relief	External feeding tube for throat constriction at T3<br>At T2 due to symptom improvement, no longer considers surgery for pain management
03	Lives alone; two children live closeby	BPA	Attended 3 BPA sessions	Resume physical and social activities as before	Worse night pain, builds up from day<br>Takes pain medication	Pain worsened over 5 months<br>Constant pain<br>Increased pain (particularly at night)<br>Takes more medication to sleep	Decompression laminectomy, since T2<br>Constant pain<br>Pain intensified<br>(following decompression surgery)<br>Remains on same pain medication, more dose & frequency	Progressive worsening of pain	Reduced walking distance & duration due to pain<br>Sits more when out	Pain inhibits mobility<br>Difficulty to do any ADL	Decompression laminectomy, since T2:<br>No change in Mobility and all activities (maintained)<br>Sits more often when out compared to previous year	Initial decrease in mobility that was maintained	No information	No information	Decompression laminectomy, since T2:<br>No impact on mental health	No impact on mental health	Impacted social life<br>Unable to mow the lawn<br>Avoids activities that involve physical activity, like going to city & around shops<br>Avoids social activities like shopping trips with friends<br>Attends weekly Pilates class (with modified exercises)<br>Stopped church voluntary café work	No change in participation restrictions	Decompression laminectomy, since T2:<br>No change in participation restrictions (Reduced voluntary work and attendance (church);<br>Stopped attending walking group<br>Reduced pace with Pilates class)	Participation restrictions remained throughout	Hip and leg pain-No info- Leg pain intensified after surgery	No comorbid issues reported.	Takes help from others: hired a person to maintain lawns and prune shrubs; daughter cleans stairs<br>After T1, Parks car away from destination, to integrate walking to shopping<br>Avoids doing stairs unnecessarily at home.<br>Modified social outings to reduce walking and standing	Aging is inevitable, but staying active helps staying young<br>NC is age related, accepts living with it<br>Health issues are part of aging<br>Activity is good for aging people	Not interested in trying a mobility aid as did not want to draw attention.	Other people noticed participant's limp while walking.<br>Family keen on considering other treatment options	Less optimistic of BOOST exercises, thinks surgery is final option<br>BOOST exercises were appropriate, but ineffective for pain<br>Exercises are better than nothing and good for physical health<br>BOOST has changed behaviour (increase in exercise level)<br>Pessimistic on prognosis- would worsen<br>BOOST would have been costly<br>BOOST gave quicker access to treatment	NO.<br>Reduced activity and participation.	Intensified pain after L4-5 decompression laminectomy at T3<br>Disappointed with outcome of surgery
04	Lives with spouse with a small back patio garden.<br>Previously moved from two-storey house,<br>as no longer able to manage stairs; close contact with children	BOOST	Attended 11 of 12 group sessions.	Be pain free and return to building trade	Pain has increased since previous levels and constant 24/7.<br>Takes multiple pain meds	[illegible]	[illegible]	[illegible]	[illegible]	[illegible]	[illegible]	[illegible]	[illegible]	[illegible]	[illegible]	[illegible]	[illegible]	[illegible]	[illegible]	[illegible]	[illegible]	[illegible]	[illegible]	[illegible]	[illegible]	[illegible]	[illegible]	[illegible]	[illegible]
24	Lives with spouse. Weekly contact with daughter	BOOST	Attended all 12 group sessions	Get better sleep	Distinct symptoms at day & night.<br>Predominantly occurs at night.<br>Night time burning pain<br>Uses gel or Paracetamol or a hot drink for pain relief	[illegible]	[illegible]	[illegible]	[illegible]	[illegible]	[illegible]	[illegible]	[illegible]	[illegible]	[illegible]	[illegible]	[illegible]	[illegible]	[illegible]	[illegible]	[illegible]	[illegible]	[illegible]	[illegible]	[illegible]	[illegible]	[illegible]	[illegible]	[illegible]
30	Lives alone; Weekly contact with family	BOOST	Attended 10 group sessions	Be pain-free	Some improvement in pain in previous 2 weeks of T1 (attributes to pain medication & daily flexion exercises)<br>Pain generally present on waking, eases after ~1 hour	[illegible]	[illegible]	[illegible]	[illegible]	[illegible]	[illegible]	[illegible]	[illegible]	[illegible]	[illegible]	[illegible]	[illegible]	[illegible]	[illegible]	[illegible]	[illegible]	[illegible]	[illegible]	[illegible]	[illegible]	[illegible]	[illegible]	[illegible]	[illegible]
31	Lives alone; Frequent contact with family	BPA	Attended 3 BPA sessions	Be pain-free and walk better	Reduced pain with Gabapentin, 900mg/day<br>Balance issues	[illegible]	[illegible]	[illegible]	[illegible]	[illegible]	[illegible]	[illegible]	[illegible]	[illegible]	[illegible]	[illegible]	[illegible]	[illegible]	[illegible]	[illegible]	[illegible]	[illegible]	[illegible]	[illegible]	[illegible]	[illegible]	[illegible]	[illegible]	[illegible]
07	Lives with spouse; Close contact with multiple family members	BPA	Attended 3 BPA sessions	Found it difficult to target an improvement due to multimorbid issues.<br>Would like to walk more (2-3 times per week) and get stronger.<br>Needed information about NC.	Main symptom is pain and cramps. Morning symptoms (pain and cramps) has slightly improved over the past few months.	[illegible]	[illegible]	[illegible]	[illegible]	[illegible]	[illegible]	[illegible]	[illegible]	[illegible]	[illegible]	[illegible]	[illegible]	[illegible]	[illegible]	[illegible]	[illegible]	[illegible]	[illegible]	[illegible]	[illegible]	[illegible]	[illegible]	[illegible]	[illegible]
08	Lives with spouse	BPA	Attended 1 session	To sleep better	Pain improved from 8 months ago.<br>Takes Co-codamol	[illegible]	[illegible]	[illegible]	[illegible]	[illegible]	[illegible]	[illegible]	[illegible]	[illegible]	[illegible]	[illegible]	[illegible]	[illegible]	[illegible]	[illegible]	[illegible]	[illegible]	[illegible]	[illegible]	[illegible]	[illegible]	[illegible]	[illegible]	[illegible]
11	Lives with spouse & son in council flat	BOOST	Attended 2 of 12 group sessions (Mobility issues & Parking costs)	Doesn't expect 100% recovery.<br>Reduced pain and more mobility	Severe pain in morning, eases gradually after medication- normal level around late morning.<br>Takes Gabapentin, Tramadol, paracetamol & TENS	[illegible]	[illegible]	[illegible]	[illegible]	[illegible]	[illegible]	[illegible]	[illegible]	[illegible]	[illegible]	[illegible]	[illegible]	[illegible]	[illegible]	[illegible]	[illegible]	[illegible]	[illegible]	[illegible]	[illegible]	[illegible]	[illegible]	[illegible]	[illegible]
14	Lives with spouse<br>Has good social and family network	BOOST	Attended 11 of 12 group sessions	Being able to walk	Increased pain in hips, buttocks and low back<br>Not keen on meds, takes 1/2 dose of prescribed codeine due to concerns about addiction & sleepiness	[illegible]	[illegible]	[illegible]	[illegible]	[illegible]	[illegible]	[illegible]	[illegible]	[illegible]	[illegible]	[illegible]	[illegible]	[illegible]	[illegible]	[illegible]	[illegible]	[illegible]	[illegible]	[illegible]	[illegible]	[illegible]	[illegible]	[illegible]	[illegible]
01	Lives alone	BOOST	Attended all 12 group sessions	Pain relief	Pain is main concern<br>Pain from right hip to bottom of foot<br>Coping with pain	[illegible]	[illegible]	[illegible]	[illegible]	[illegible]	[illegible]	[illegible]	[illegible]	[illegible]	[illegible]	[illegible]	[illegible]	[illegible]	[illegible]	[illegible]	[illegible]	[illegible]	[illegible]	[illegible]	[illegible]	[illegible]	[illegible]	[illegible]	[illegible]
15	Lives with spouse	BPA	Discharged at 2nd session	Reassurance that exercises wont harm (Mental impact > Physical impact)	Constant pain<br>Pain on standing (within 1-2 mts)<br>Pain leads to numbness/pins needles in foot, with walking<br>Progressive leg weakness	[illegible]	[illegible]	[illegible]	[illegible]	[illegible]	[illegible]	[illegible]	[illegible]	[illegible]	[illegible]	[illegible]	[illegible]	[illegible]	[illegible]	[illegible]	[illegible]	[illegible]	[illegible]	[illegible]	[illegible]	[illegible]	[illegible]	[illegible]	[illegible]
25	Lives alone<br>adult children closeby<br>Supportive neighbours	BOOST	Attended 11 of 12 group sessions	To walk better	Constant, burning pain in right thigh<br>Stiffness on walking<br>Pain killers werent helping much, so takes Paracetamol<br>Wants to cut down Paracetamol	[illegible]	[illegible]	[illegible]	[illegible]	[illegible]	[illegible]	[illegible]	[illegible]	[illegible]	[illegible]	[illegible]	[illegible]	[illegible]	[illegible]	[illegible]	[illegible]	[illegible]	[illegible]	[illegible]	[illegible]	[illegible]	[illegible]	[illegible]	[illegible]
33	Lives with spouse	BPA	Attended 3 BPA sessions	To walk normally and able to do things without pain	Not much pain, but numbness in legs that makes walking difficult<br>Has good and bad days	[illegible]	[illegible]	[illegible]	[illegible]	[illegible]	[illegible]	[illegible]	[illegible]	[illegible]	[illegible]	[illegible]	[illegible]	[illegible]	[illegible]	[illegible]	[illegible]	[illegible]	[illegible]	[illegible]	[illegible]	[illegible]	[illegible]	[illegible]	[illegible]
38	Lives with spouse	BPA	Attended 3 BPA sessions	To get back to squash sport<br>There wouldn't be too many things that participant would do that are impossible to do now	Ocassional pain in buttocks while walking, that is manageable<br>Pain in feet and legs that makes walking difficult<br>Numbness in feet makes participant feel losing control and balance while walking	[illegible]	[illegible]	[illegible]	[illegible]	[illegible]	[illegible]	[illegible]	[illegible]	[illegible]	[illegible]	[illegible]	[illegible]	[illegible]	[illegible]	[illegible]	[illegible]	[illegible]	[illegible]	[illegible]	[illegible]	[illegible]	[illegible]	[illegible]	[illegible]
41	Lives with spouse	BOOST	Attended all 12 group sessions	To improve leg strength	No problem in the back<br>Pain, weakness & numbness from buttocks to feet<br>Buttock 'ache'<br>Numbness in legs 'always there'<br>Leg weakness & balance problems	[illegible]	[illegible]	[illegible]	[illegible]	[illegible]	[illegible]	[illegible]	[illegible]	[illegible]	[illegible]	[illegible]	[illegible]	[illegible]	[illegible]	[illegible]	[illegible]	[illegible]	[illegible]	[illegible]	[illegible]	[illegible]	[illegible]	[illegible]	[illegible]
05	Lives with spouse; weekly contact with adult children	BOOST	Attended 10 of 12 group sessions	To become pain free	Low back pain radiating to buttock and right leg<br>Pain is always there in varying degrees<br>No issues with balance or strength<br>Takes Paracetamol, only when very painful	[illegible]	[illegible]	[illegible]	[illegible]	[illegible]	[illegible]	[illegible]	[illegible]	[illegible]	[illegible]	[illegible]	[illegible]	[illegible]	[illegible]	[illegible]	[illegible]	[illegible]	[illegible]	[illegible]	[illegible]	[illegible]	[illegible]	[illegible]	[illegible]
09	Lives with spouse	BOOST	Attended 5 of 12 sessions<br>Had an accident, spouse's heart attack,<br>Hours to reach Oxford and back, unable<br>to walk 20 minutes of walking circuit	Less pain for the movement<br>To do hard jobs quicker<br>To get MRI done	Pain in right leg, all the time<br>Sore lower back within 300 yards of walking<br>Takes pain killers, if pain gets worse	[illegible]	[illegible]	[illegible]	[illegible]	[illegible]	[illegible]	[illegible]	[illegible]	[illegible]	[illegible]	[illegible]	[illegible]	[illegible]	[illegible]	[illegible]	[illegible]	[illegible]	[illegible]	[illegible]	[illegible]	[illegible]	[illegible]	[illegible]	[illegible]
26	Lives with extended family	BOOST	Attended 11 of 12 group sessions.<br>Missed one session because of holiday trip	To become independant by 50%<br>To improve general health	No pain. Main symptoms are stiffness and numbness in back, feet and ankles<br>Numbness and instability always there<br>Feelings of unsteadiness due to leg weakness<br>Heaviness and numbness in legs and feet after walking a certain distance<br>Takes Paracetamol for pain relief<br>Finds acupuncture helpful in pain relief, but doesnt go often due to treatment costs	[illegible]	[illegible]	[illegible]	[illegible]	[illegible]	[illegible]	[illegible]	[illegible]	[illegible]	[illegible]	[illegible]	[illegible]	[illegible]	[illegible]	[illegible]	[illegible]	[illegible]	[illegible]	[illegible]	[illegible]	[illegible]	[illegible]	[illegible]	[illegible]
28	Lives with spouse;<br>daughter & family in other half of house	BOOST	Attended 9 of 12 group sessions.<br>Missed sessions due to trip to Spain<br>Attending sessions was tiring bus journey (10 to 5),<br>but felt like day out and managed them.<br>Felt it was a real achievement.	Doesn't expect 100% recovery.<br>To be mobile* and improve feet and leg symptoms<br>*Mobile (able to go out without support and do stairs better, go abroad)	Constant back pain, that is manageable.<br>Pain and feelings of weakness in legs that worsened previous six months<br>Takes Paracetamol to manage pain<br>Pain & balance issues due to leg weakness while walking	[illegible]	[illegible]	[illegible]	[illegible]	[illegible]	[illegible]	[illegible]	[illegible]	[illegible]	[illegible]	[illegible]	[illegible]	[illegible]	[illegible]	[illegible]	[illegible]	[illegible]	[illegible]	[illegible]	[illegible]	[illegible]	[illegible]	[illegible]	[illegible]
37	Lives alone	BPA	No information available	Pain relief and being able to walk to shops	Impact of NC is very minimal<br>Has no back pain.<br>Discomfort and heaviness in legs while walking uphill, 300 yards or during ocassional calf tightness in the mornings	[illegible]	[illegible]	[illegible]	[illegible]	[illegible]	[illegible]	[illegible]	[illegible]	[illegible]	[illegible]	[illegible]	[illegible]	[illegible]	[illegible]	[illegible]	[illegible]	[illegible]	[illegible]	[illegible]	[illegible]	[illegible]	[illegible]	[illegible]	[illegible]
57	Lives with spouse; Visits daughter, locally (goes twice a day)	BOOST	No information available	To get rid of pain and walk better	Horrible Leg pain > back pain, making participant limp<br>Within 5-10 minutes, back pain radiates down and making to limp<br>No issues with balance<br>Takes Paracetamol only if needed	[illegible]	[illegible]	[illegible]	[illegible]	[illegible]	[illegible]	[illegible]	[illegible]	[illegible]	[illegible]	[illegible]	[illegible]	[illegible]	[illegible]	[illegible]	[illegible]	[illegible]	[illegible]	[illegible]	[illegible]	[illegible]	[illegible]	[illegible]	[illegible]
32	Lives with spouse	BPA	Attended one BPA session	Pain relief	Worse pain than before. Horrendous night pain in legs.<br>Due to mulitple MSK issues, it was difficult to establish NC pain alone.	[illegible]	[illegible]	[illegible]	[illegible]	[illegible]	[illegible]	[illegible]	[illegible]	[illegible]	[illegible]	[illegible]	[illegible]	[illegible]	[illegible]	[illegible]	[illegible]	[illegible]	[illegible]	[illegible]	[illegible]	[illegible]	[illegible]	[illegible]	[illegible]
34	Lives alone	BPA	No information available	To get rid of sciatic pain so as to sleep better.	Two pain presentations: Back & leg and Sciatic nerve pain<br>Sciatic nerve pain 24/7; Constant pain<br>Variable pain in mid-back<br>Tingling down the legs<br>Pain frequency more than before<br>Ocassional balance problems	[illegible]	[illegible]	[illegible]	[illegible]	[illegible]	[illegible]	[illegible]	[illegible]	[illegible]	[illegible]	[illegible]	[illegible]	[illegible]	[illegible]	[illegible]	[illegible]	[illegible]	[illegible]	[illegible]	[illegible]	[illegible]	[illegible]	[illegible]	[illegible]
39	Lives with spouse	BOOST	Attended 4 of 12 group sessions<br>Missed 2-3 sessions due to holidays	No heaviness in legs while walking<br>Improve mentally and physically	Feelings of heaviness in both legs & being cumbersome<br>Worse pain while pottering aroud house<br>Issues with balance while walking<br>Takes painkillers only if needed<br>Need to sit down for 10-15 minutes or have a pain killer as legs become cumbersome	[illegible]	[illegible]	[illegible]	[illegible]	[illegible]	[illegible]	[illegible]	[illegible]	[illegible]	[illegible]	[illegible]	[illegible]	[illegible]	[illegible]	[illegible]	[illegible]	[illegible]	[illegible]	[illegible]	[illegible]	[illegible]	[illegible]	[illegible]	[illegible]
43	Lives with spouse	BOOST	Did 10 of 12 BOOST sessions<br>Missed 2 sessions due to illness	To confidently be able to walk and to get back on mountain bike	Symptoms progressively worsened over past year<br>Pain below belt line<br>Stiffness during kneeling to standing<br>Pain, aching, or pulling in top of buttocks, thighs and calf muscle<br>Numbness and pain in left thigh, back of calf muscles and restricted feeling around left ankle<br>Takes Paracetamol daily, but not keen o taking medication.	[illegible]	[illegible]	[illegible]	[illegible]	[illegible]	[illegible]	[illegible]	[illegible]	[illegible]	[illegible]	[illegible]	[illegible]	[illegible]	[illegible]	[illegible]	[illegible]	[illegible]	[illegible]	[illegible]	[illegible]	[illegible]	[illegible]	[illegible]	[illegible]
47	Lives alone	BPA	Attended 2 BPA sessions	To be able to do long walks (up to 3 miles) that participant enjoys without the need to take breaks and sit	Intolerable & intermittent pain<br>Muscle spasm in lower back<br>Takes 2 Paracetamol morning & night everyday<br>Takes 500mg Ibubrufen for back, ocassionally	[illegible]	[illegible]	[illegible]	[illegible]	[illegible]	[illegible]	[illegible]	[illegible]	[illegible]	[illegible]	[illegible]	[illegible]	[illegible]	[illegible]	[illegible]	[illegible]	[illegible]	[illegible]	[illegible]	[illegible]	[illegible]	[illegible]	[illegible]	[illegible]
50	Lives with spouse and son	BPA	Attended 2 BPA sessions	To get rid of pain and able to exercise more<br>to strengthen the back muscles	Lot of pain down the legs<br>Severe pain on walking even shorter distance (10/10)	[illegible]	[illegible]	[illegible]	[illegible]	[illegible]	[illegible]	[illegible]	[illegible]	[illegible]	[illegible]	[illegible]	[illegible]	[illegible]	[illegible]	[illegible]	[illegible]	[illegible]	[illegible]	[illegible]	[illegible]	[illegible]	[illegible]	[illegible]	[illegible]
53	Lives with spouse;<br>adult children locally	BPA	No information available	To stop leg muscle wasting and to strengthen them	Pain in legs, hips and back<br>Muscle wasting in both legs<br>Tingling in both feet<br>Takes gabapentin x 3 times, but not helpful<br>Avoids pain meds during ocassions that are manageable	[illegible]	[illegible]	[illegible]	[illegible]	[illegible]	[illegible]	[illegible]	[illegible]	[illegible]	[illegible]	[illegible]	[illegible]	[illegible]	[illegible]	[illegible]	[illegible]	[illegible]	[illegible]	[illegible]	[illegible]	[illegible]	[illegible]	[illegible]	[illegible]
06	Lives alone	BOOST	Attended 9 sessions.<br>Participant missed one due to rain and two due to holidays in Jamaica.	Pain relief	Constant pain<br>Pain around the buttocks and back of both thighs.<br>Takes painkiller (Paracetamol) in severe pain or before tasks that aggravates pain	[illegible]	[illegible]	[illegible]	[illegible]	[illegible]	[illegible]	[illegible]	[illegible]	[illegible]	[illegible]	[illegible]	[illegible]	[illegible]	[illegible]	[illegible]	[illegible]	[illegible]	[illegible]	[illegible]	[illegible]	[illegible]	[illegible]	[illegible]	[illegible]

For peer review only

Supplementary file 4: Patterns of change in biopsychosocial domains at T1, T2, and T3 (ordered by treatment arm and participant study code)

		Pain			Mobility and ADL			Psychological Impact			Social and recreational participation		
Participant code	Treatment arm	Baseline	6 months	12 months	Baseline	6 months	12 months	Baseline	6 months	12 months	Baseline	6 months	12 months
BOQ01	BOOST												
BOQ04	BOOST												
BOQ05	BOOST												
BOQ06	BOOST												
BOQ09	BOOST												
BOQ11	BOOST												
BOQ14	BOOST												
BOQ24	BOOST												
BOQ25	BOOST												
BOQ26	BOOST												
BOQ28	BOOST												
BOQ30	BOOST												
BOQ39	BOOST												
BOQ41	BOOST												
BOQ43	BOOST												
BOQ57	BOOST												
BOQ02	BPA												
BOQ03	BPA												
BOQ07	BPA												
BOQ08	BPA												
BOQ15	BPA												
BOQ31	BPA												
BOQ32	BPA												
BOQ33	BPA												
BOQ34	BPA												
BOQ37	BPA												
BOQ38	BPA												
BOQ47	BPA												
BOQ50	BPA												
BOQ53	BPA												

BOOST: BOOST programme; BPA: Best Practice Advice; ADL: Activities of Daily Living

For peer review only - http://bmjopen.bmj.com/site/about/guidelines.xhtml

Legend

	When participants already had reduced pain/improved sleep/ improved mobility/ improved social and recreational participation/positive psychological impact at entry to trial or had improvements compared to a previous time point.
	Further reduction in pain, improved sleep/increase in mobility/ increase in social and recreational participation/positive psychological impact compared to a previous time point.
	Continued reduction in pain/improved sleep/increase in mobility/ increase in social and recreational participation/positive psychological impact compared to a previous time point.
	When participants already had increased pain/sleep disturbances/decreased mobility/ social and recreational participation/negative psychological impact at entry to trial or worsened status compared to a previous time point.
	Further increase in pain/sleep disturbances/decrease in mobility/decrease in social and recreational participation/negative psychological impact compared to a previous time point.
	Continued increase in pain/sleep disturbances/decrease in mobility/decrease in social and recreational participation/negative psychological impact compared to a previous time point.
	No change in pain/sleep/mobility/social and recreational participation/psychological impact compared to a previous time point.
	Lack of sufficient information from interviews.
	When pain, sleep, mobility, social and recreational participation, or psychological impact influenced by other musculoskeletal (MSK)/non-MSK issues or major health events.

For peer review only - http://bmjopen.bmj.com/site/about/guidelines.xhtml

Title: Longitudinal qualitative study of living with neurogenic claudication

Reporting checklist for qualitative study.

Based on the SRQR guidelines.

Instructions to authors

Complete this checklist by entering the page numbers from your manuscript where readers will find each of the items listed below.

Your article may not currently address all the items on the checklist. Please modify your text to include the missing information. If you are certain that an item does not apply, please write "n/a" and provide a short explanation.

Upload your completed checklist as an extra file when you submit to a journal.

In your methods section, say that you used the SRQRreporting guidelines, and cite them as:

O'Brien BC, Harris IB, Beckman TJ, Reed DA, Cook DA. Standards for reporting qualitative research: a synthesis of recommendations. Acad Med. 2014; 89(9):1245-1251.

		Reporting Item	Page Number
**Title**			
	#1	Concise description of the nature and topic of the study identifying the study as qualitative or indicating the approach (e.g. ethnography, grounded theory) or data collection methods (e.g. interview, focus group) is recommended	1
**Abstract**			
	#2	Summary of the key elements of the study using the abstract format of the intended publication; typically includes background, purpose, methods, results and conclusions	2
**Introduction**			
Problem formulation	#3	Description and significance of the problem / phenomenon studied: review of relevant theory and empirical work; problem statement	4, 5

For peer review only

Title: Longitudinal qualitative study of living with neurogenic claudication

Purpose or research question	#4	Purpose of the study and specific objectives or questions	5
**Methods**			
Qualitative approach and research paradigm	#5	Qualitative approach (e.g. ethnography, grounded theory, case study, phenomenolgy, narrative research) and guiding theory if appropriate; identifying the research paradigm (e.g. postpositivist, constructivist / interpretivist) is also recommended; rationale. The rationale should briefly discuss the justification for choosing that theory, approach, method or technique rather than other options available; the assumptions and limitations implicit in those choices and how those choices influence study conclusions and transferability. As appropriate the rationale for several items might be discussed together.	5
Researcher characteristics and reflexivity	#6	Researchers' characteristics that may influence the research, including personal attributes, qualifications / experience, relationship with participants, assumptions and / or presuppositions; potential or actual interaction between researchers' characteristics and the research questions, approach, methods, results and / or transferability	24
Context	#7	Setting / site and salient contextual factors; rationale	5, 6
Sampling strategy	#8	How and why research participants, documents, or events were selected; criteria for deciding when no further sampling was necessary (e.g. sampling saturation); rationale	5, 6
Ethical issues pertaining to human subjects	#9	Documentation of approval by an appropriate ethics review board and participant consent, or explanation for lack thereof; other confidentiality and data security issues	5
Data collection methods	#10	Types of data collected; details of data collection procedures including (as appropriate) start and stop dates of data collection and analysis, iterative process, triangulation of sources / methods, and modification of	6

For peer review only

Title: Longitudinal qualitative study of living with neurogenic claudication

		procedures in response to evolving study findings; rationale	
Data collection instruments and technologies	#11	Description of instruments (e.g. interview guides, questionnaires) and devices (e.g. audio recorders) used for data collection; if / how the instruments(s) changed over the course of the study	6
Units of study	#12	Number and relevant characteristics of participants, documents, or events included in the study; level of participation (could be reported in results)	Table 1<br>Page 9
Data processing	#13	Methods for processing data prior to and during analysis, including transcription, data entry, data management and security, verification of data integrity, data coding, and anonymisation / deidentification of excerpts	6
Data analysis	#14	Process by which inferences, themes, etc. were identified and developed, including the researchers involved in data analysis; usually references a specific paradigm or approach; rationale	6, 7
Techniques to enhance trustworthiness	#15	Techniques to enhance trustworthiness and credibility of data analysis (e.g. member checking, audit trail, triangulation); rationale	6, 7
**Results/findings**			
Syntheses and interpretation	#16	Main findings (e.g. interpretations, inferences, and themes); might include development of a theory or model, or integration with prior research or theory	8-21
Links to empirical data	#17	Evidence (e.g. quotes, field notes, text excerpts, photographs) to substantiate analytic findings	Pages 13, 15, 16, 19, 20<br>Tables 3, 4, 5<br>Supplementary file 2
**Discussion**			
Intergration with prior	#18	Short summary of main findings; explanation of how	21-24

For peer review only

Title: Longitudinal qualitative study of living with neurogenic claudication

work, implications, transferability and contribution(s) to the field		findings and conclusions connect to, support, elaborate on, or challenge conclusions of earlier scholarship; discussion of scope of application / generalizability; identification of unique contributions(s) to scholarship in a discipline or field	
Limitations	#19	Trustworthiness and limitations of findings	23-24
**Other**			
Conflicts of interest	#20	Potential sources of influence of perceived influence on study conduct and conclusions; how these were managed	24
Funding	#21	Sources of funding and other support; role of funders in data collection, interpretation and reporting	24-25

None The SRQR checklist is distributed with permission of Wolters Kluwer © 2014 by the Association of American Medical Colleges. This checklist can be completed online using https://www.goodreports.org/, a tool made by the EQUATOR Network in collaboration with Penelope.ai

For peer review only

BMJ Open

Longitudinal qualitative study of living with neurogenic claudication

Journal:	*BMJ Open*
Manuscript ID	bmjopen-2021-060128.R1
Article Type:	Original research
Date Submitted by the Author:	01-Jun-2022
Complete List of Authors:	Griffiths, Frances; University of Warwick, Warwick Medical School<br>Srikesavan, Cynthia; University of Oxford, Nuffield Department of Orthopaedics, Rheumatology, and Musculoskeletal Sciences<br>Ward, Lesley; Northumbria University, Department of Sport, Exercise & Rehabilitation<br>Boniface, Graham; University of Oxford, Nuffield Department of Orthopaedics, Rheumatology, and Musculoskeletal Sciences<br>Williamson, Esther; University of Oxford, Nuffield Department of Orthopaedics, Rheumatology and Musculoskeletal Sciences<br>Lamb, Sarah; University of Exeter, College of Medicine and Health; University of Oxford, Nuffield Department of Orthopaedics, Rheumatology and Musculoskeletal Sciences
**Primary Subject Heading**:	Rehabilitation medicine
Secondary Subject Heading:	Rehabilitation medicine
Keywords:	Back pain < ORTHOPAEDIC & TRAUMA SURGERY, Spine < ORTHOPAEDIC & TRAUMA SURGERY, QUALITATIVE RESEARCH, Rehabilitation medicine < INTERNAL MEDICINE

SCHOLARONE™
Manuscripts

I, the Submitting Author has the right to grant and does grant on behalf of all authors of the Work (as defined in the below author licence), an exclusive licence and/or a non-exclusive licence for contributions from authors who are: i) UK Crown employees; ii) where BMJ has agreed a CC-BY licence shall apply, and/or iii) in accordance with the terms applicable for US Federal Government officers or employees acting as part of their official duties; on a worldwide, perpetual, irrevocable, royalty-free basis to BMJ Publishing Group Ltd ("BMJ") its licensees and where the relevant Journal is co-owned by BMJ to the co-owners of the Journal, to publish the Work in this journal and any other BMJ products and to exploit all rights, as set out in our licence.

The Submitting Author accepts and understands that any supply made under these terms is made by BMJ to the Submitting Author unless you are acting as an employee on behalf of your employer or a postgraduate student of an affiliated institution which is paying any applicable article publishing charge ("APC") for Open Access articles. Where the Submitting Author wishes to make the Work available on an Open Access basis (and intends to pay the relevant APC), the terms of reuse of such Open Access shall be governed by a Creative Commons licence – details of these licences and which Creative Commons licence will apply to this Work are set out in our licence referred to above.

Other than as permitted in any relevant BMJ Author's Self Archiving Policies, I confirm this Work has not been accepted for publication elsewhere, is not being considered for publication elsewhere and does not duplicate material already published. I confirm all authors consent to publication of this Work and authorise the granting of this licence.

For peer review only

Title page

Title of the article
Longitudinal qualitative study of living with neurogenic claudication

Authors
[1]Frances Griffiths, [2]Cynthia Srikesavan, [3]Lesley Ward, [4]Graham Boniface, [5]Esther Williamson, [6]Sarah E Lamb

Full name, Postal address and email of the Corresponding author
Dr Esther Williamson
Nuffield Department of Orthopaedics, Rheumatology and Musculoskeletal Sciences, University of Oxford, Windmill Road, Oxford OX3 7LD, United Kingdom.

Email: esther.williamson@ndorms.ox.ac.uk

Full name, department, institution, city and country of all co-authors
[1]Warwick Medical School, University of Warwick, Coventry, United Kingdom.
[2, 4, 5, 6]Nuffield Department of Orthopaedics, Rheumatology, and Musculoskeletal Sciences, University of Oxford, Windmill road, Oxford, United Kingdom.
[3]Department of Sport, Exercise & Rehabilitation, Northumbria University, United Kingdom.
[6]College of Medicine and Health, Institute for Health Research, University of Exeter, St Luke's Campus, Heavitree Road, Exeter, United Kingdom.

Word count, excluding title page, abstract, references, figures and tables: 5389

Strengths and limitations of this study

- In-depth interviews at three time points focus on participant's current experience; comparison across time was undertaken during analysis so was not reliant on participant interpretation of past-experience.
- Analysis summarises succinctly a large qualitative data set.
- Summarising data will have resulted in loss of nuance in our analysis.
- The results present one set of trajectory category membership for participants; some participants could be categorised differently.
- Our interest in successful aging shaped our path through analysis; a different interest is likely to result in different trajectory descriptions.

For peer review only - http://bmjopen.bmj.com/site/about/guidelines.xhtml

Abstract

Objectives
Neurogenic claudication (NC) causes pain and reduced mobility, particularly in older people and can negatively affect mental and social well-being, so limiting successful aging. This qualitative study explored how people with NC change over 12 months.

Design
A longitudinal qualitative study using semi-structured interviews.

Setting
Participants were recruited from a UK clinical trial of a physiotherapy intervention for NC.

Participants
Interviews were undertaken at baseline, one month after receiving any intervention and at 12 months. We analysed 30 sets of three interviews.

Results
Interview data was summarised for each time point into biopsychosocial domains: pain, mobility and activities of daily living, psychological impact and social and recreational participation. Through comparative analysis we explored participant trajectories over time.

Progressive improvement in at least one domain was experienced by 13 participants but there was variability in trajectories with early improvements that remained the same, transient changes and no change also commonly observed.

Eleven participants described co-present improvement trajectories in all domains. Three participants described co-present improvement in all domains except participation; one had never stopped their participation and two had unattainable expectations. Five participants described co-present improvement in one domain and deterioration in another and 14 participants described co-present no change in one domain and change in another.

There was evidence of interaction between domains e.g. improved mobility led to improved participation and for some participants, specific factors influenced change. Of the 15 participants who experienced improved participation, ten reported improvements in all other domains and five participants did not; for two, pain did not prevent participation, one used a walking aid and two had a positive psychological outlook.

Conclusion
The daily lived experiences of older adults with NC are variable and include interaction between biopsychosocial domains. Therapist understanding of these trajectories and their interactions may help to provide personalised therapy

Keywords: Neurogenic claudication; Longitudinal; Interviews; Trajectory

For peer review only - http://bmjopen.bmj.com/site/about/guidelines.xhtml

Main text

Background

Neurogenic claudication (NC) is caused by narrowing of the spinal canal (lumbar canal stenosis) most often due to osteoarthritis of the spine or spondylosis. It is frequently reported in adults aged above 60 years (1, 2). NC usually presents as pain and discomfort radiating from the back into the buttocks and legs in a unilateral or bilateral pattern. It is provoked by walking or prolonged standing and relieved by sitting or lumbar flexion (3). The pain often occurs intermittently. Other signs and symptoms may include weakness, altered sensations such as pins and needles and numbness, fatigue and gait changes (3). Pain in the lower back is usual but not a necessary diagnostic feature but regardless NC is considered a low back pain condition. NC is associated with decreased mobility and independence for older people, restriction of social activities, a negative emotional impact, and frailty (4, 5). The impact of back and leg pain consistent with NC on health status is more marked than for people with other patterns of back and leg pain; for older adults, compared to those who report back pain only or have leg pain which is not consistent with NC, those with NC report lower quality of life and are more likely to be frail, be less confident to walk ½ a mile and to report a decline in their mobility over the last year (1). The variation in symptom trajectory for people with NC is illustrated by a study of 146 people with NC followed up for 3 years (without surgical treatment), symptoms improved for about one third of patients, remained unchanged for half the patients and worsened in a few (6).

Ageing successfully is considered a key mechanism by which ageing populations can maximise their health and longevity and minimise their need to access health care systems (7). The concept of successful aging considers physical and mental health along with social and recreational engagement (7) and since its conception has been expanded to include living with multi-morbidities and chronic conditions such as NC (8-10). Our concern in this study is whether the symptoms of NC may lead to older people being unable to age successfully. We were particularly concerned to understand whether and how NC impacted on social and recreational participation and how this changed over time.

<u>Qualitative evidence on living with back pain</u>

The interaction physical and mental health and social and recreational engagement has been explored in qualitative studies of people living with low back pain and synthesised in a meta-ethnography (11). The interrelated themes identified were: impact of pain on self, use of coping strategies and impact of pain on engagement with others. The meta-ethnography concluded that chronic low back pain is complex, dynamic and multidimensional with those living with the pain experiencing distressing pain and loss with lowered self-worth, stigma, depression, premature aging, and fear of the future. A later cross-sectional qualitative study of people with NC, suggests that pain, physical ability and emotions are inter-related and at times inseparable (5). The meta-ethnography identified a lack of longitudinal studies. A subsequent longitudinal study interviewed eight people living with back pain annually over two years (12). Although pain and loss impacted on all areas of the participants lives, two different patterns of change over time were identified: participants who saw their pain a physical problem and a cause of loss and did not re-establish social and recreational activities, and those who experienced pain relief and were less enmeshed in the pain experience so able to look to the future (12).

For peer review only - http://bmjopen.bmj.com/site/about/guidelines.xhtml

Such longitudinal qualitative studies provide a depth of understanding to complement existing panel studies (13) and build on the idea of illness trajectory (14), biographical disruption (15) and understanding individuals as unique, complex and adaptive (16-18). Our study aims to provide this depth of understanding of people living with NC: how their NC impacts on their function and engagement and how this changes over time. It builds further on longitudinal qualitative studies in the health domain (19).

The BOOST trial

This qualitative study was embedded within the Better Outcomes for Older people with Spinal Trouble (BOOST) trial (20, 21), a multi-centre, randomised controlled trial with two treatment arms. It compared a 12-week group-based physical and psychological intervention (the BOOST programme) to individual best practice advice (BPA) for older adults (65+ years) with NC. The BOOST programme comprised group discussions that were underpinned by cognitive behavioural techniques, an individually tailored exercise and walking circuit, and home exercises. BPA comprised home exercises and self-management (1-3 appointments with a physiotherapist). Participants aged 65 years or over with the clinical presentation of NC symptoms were recruited from primary care practices of the National Health Service (NHS) in the United Kingdom (UK). People living in residential care or nursing homes, those registered blind and those with a terminal health condition or on a waiting list for surgery or unable to walk 3m without the support of another person or unable to provide informed signed consent were not included. Participants were randomised in a 2:1 ratio (BOOST programme: BPA). Full trial protocol details are reported elsewhere (21).

Methods

Study design

We used a longitudinal qualitative research design to understand the individual at the time of each interview (22) rather than seeking from the individuals their own narrative of change (23). We aimed to interview participants three times: at trial baseline (after randomisation but before the participant knew their treatment allocation) (T1); one month after any intervention (T2) and 12 months post-randomisation (T3). TI interviews were via face-to-face at participant's home, with T2 and T3 by telephone. Where participants had difficulty using the telephone, T2 and T3 interviews were undertaken face-to-face.

Ethics approval

Ethics approval was given by the London-Brent National Research Ethics Committee (REC number 16/LO/0349) on 03 March 2016.

Participants and Recruitment

All participants recruited to the BOOST trial (20, 21) were eligible for the interview study and consent to invite participants for interview was collected as part of trial procedures. We invited participants consecutively from each recruitment site over the 25-month trial recruitment period (August 2016 to August 2018), stratifying to ensure balance between range of age, gender, ethnicity, treatment allocation (ratio 1:1) and recruitment site location.

For peer review only - http://bmjopen.bmj.com/site/about/guidelines.xhtml

To ensure sufficient information power (24), we aimed for 30 participants retained at 12 months and so aimed to recruit approx. 60 participants to allow for 50% attrition.

Data collection

We obtained written consent at the start of the first interview and reaffirmed this verbally at follow-up interviews. We used semi-structured interview schedules (**Supplementary file 1**). We used the present tense when asking questions to encourage participants to focus on their current state (22). In T1 interviews, we explored the impact of NC on participant physical and psychosocial health including NC and their beliefs around exercise and ageing. In T2 and T3 interviews we explored similar issues along with their experiences of the BOOST trial interventions. Before T2 and T3 interviews we reviewed for each participant the data captured in their earlier interviews and made notes about the participant and their context. We used this knowledge in the interviews to rapidly establish rapport on the telephone and enable quick clarifications. For example:

> Participant: I go to my club.
>
> Interviewer: Is that the ladies club you mentioned last time we met?

All interviews were audio-recorded onto encrypted recorders and stored securely at University of Oxford. During transcription/summarising all personal identifiers were removed.

Data analysis

Throughout our analysis we compared data across time and between participants and drew on existing evidence-based concepts and definitions related to back pain and NC. We mapped the data into a framework matrix (25) then explored the matrix (26, 27) for change processes related to NC. This involved summarising patterns of change being experienced at each time point, trajectories of change between time points (27) co-presence of trajectories, interactions between trajectories and turning points. In our analysis of data at each time point we used an analysis approach we had previously developed (18) and applied (22). We focused on current experience and put to one side data where the participant was interpreting past-experience. This allowed us to compare what was current at each time point and to manage the volume of complex data (28). We describe each analysis step below. Analysis was conducted in Excel.

Step 1: Development of individual participant frameworks

We started analysis on complete interview sets (T1, T2 and T3). We selected sets where the participant provided rich data about the experience of NC. After selecting 30 sets (90 interviews) and undertaking initial analysis (see Step 2 below) we read and discussed our remaining data to check whether it was revealing new themes; we determined we had achieved data saturation(29). All T1 interviews were transcribed verbatim along with the first 10 T2 and 10 T3 interviews we analysed. The remaining interviews were analysed directly from audio recordings. To immerse ourselves in this large volume of data we initially wrote structured pen portraits(30) at each time point for ten data sets. This involved reading and rereading the data, clarifying our focus on current experience related to NC, summarising participant experiences and context with illustrative quotes, and developing a structure that worked across the 10 data sets. From these we developed and refined a coding template. We used this to thematically code all thirty interview sets, writing mini statements with illustrative quotes for each relevant code/sub code at each time point. The resulting 30 individual participant frameworks were used for subsequent analysis.

For peer review only - http://bmjopen.bmj.com/site/about/guidelines.xhtml

Step 2: Development of change framework

After team discussions, we summarised the coded data for each participant at each time point into the following biopsychosocial domains: pain, mobility and activities of daily living (ADL), psychological impact, social and recreational participation and sleep. For example, to summarise mobility status at T1, we collated and summarised data from codes: current mobility, activities reduced, activities stopped, activities increased, and sub-codes such as physical impact. Participants reported a range of symptoms including numbness, tingling and heaviness. However, the symptom that consistently predominated across all participant's accounts of their experience of NC was pain; we therefore summarised the experience of pain in the framework. We included data that conveyed the pattern of change each participant was experiencing at the time of each interview. For example, a gradual improvement or worsening as experienced day-to-day/week-to-week. We added to the change framework participant age, gender, ethnicity, living arrangement, group allocation in the trial, health events and co-morbid conditions. We summarised participant coping strategies, views on aging, use of mobility aids and feedback from others.

Step 3: Identification and categorisation of participant trajectories

To identify participant trajectories for each biopsychosocial domain, we compared the summaries in the change framework for what increased or emerged over time, what decreased or ceased over time and what remained consistent over time (26). We added a description of this trajectory to the change framework. In a new table we presented for each domain the colour coded trajectory for each participant. Each team member then independently, by eye, ordered the participants so those with the most similar combination of trajectories were adjacent. The team then compared their resulting tables, discussed similarities and differences, and agreed on one version of the table. Using this table, we then independently categorised the participants into groups based on similarity of trajectories and discussed and agreed categorisation.

Step 4: Exploring the nature of the trajectories

Identifying and categorising co-presence and interaction between the trajectories of the individual biopsychosocial domains

We scrutinised the change framework for each participant and compared participants to understand which trajectories were co-present at each time point and so may have interacted with each other. For example, a reduction in pain was accompanied by improvement in mobility or deterioration in psychological outlook was accompanied by reduced social or recreational participation. We returned to participant data to check whether the participant reported interaction between the trajectories. We grouped similar types of co-presence and explored how these related to our trajectory categories. Given our interest in successful aging, we particularly explored how social and recreational engagement changed along with other domains.

Identification of health-related turning points

We examined the data for health-related turning points mentioned by the participant; what the participants considered significant change in their health during the study timeframe.

Totality of change

From the whole change framework we examined factors that were not inseparable from trajectory category although are likely to be interrelated (5). Factors included whether they

For peer review only - http://bmjopen.bmj.com/site/about/guidelines.xhtml

lived alone, how they considered aging and use of mobility aids, and strategies for coping with NC in their daily lives. We identified what was similar and different between participants in the different trajectory categories.

Study team roles

SEL and EW (health service researchers and physiotherapists) led the BOOST programme of work including this study and the BOOST trial. FG (general medical practitioner, medical sociologist and qualitative researcher) led the qualitative study team that included LW (qualitative health researcher), CS (physiotherapist and a qualitative researcher) and GB (physiotherapy researcher). All contributed to qualitative study design, reading data, thematic and comparative analysis, synthesis, interpretation and reporting. LW undertook all interviews. LW and FG reviewed the interview schedules to improve the clarity and flow of the questions. CS worked with FG on analysis with GB providing independent review of coding/summaries. Any discrepancies were resolved through discussion. EW and SEL reviewed each step of the analysis and provided feedback to FG, LW, CS, and GB.

This study team were not involved in the delivery of BOOST interventions or outcome assessments in the trial. FG, LW, CS, GB remained blinded to the results of the BOOST trial until they had completed data coding and analysis.

Reporting

The Standards for Reporting Qualitative Research (SRQR) guidelines (31) were used to report the study. Quotations from interviews are labelled with participant number and interview T1, T2 or T3.

Results

The data

Participant attrition across the three time points was lower than expected, with 49 of the 60 participants (82%) providing three interviews. Reasons for withdrawal were: withdrawal from clinical trial (n=3), ill health/awaiting investigations (n=4), increased pain after receiving BOOST programme sessions (n=2), declined third interview (n=3), unable to contact (n=3) and withdrawn by researcher as participant postponed >6 months (n=2). Initial interviews averaged 70 minutes (range 44-93 minutes), with second and third interviews averaging 36 minutes and 32 minutes respectively.

Participant characteristics

There was little difference in terms of age (average 75 years), gender and ethnicity between trial, interview, and analysis cohorts and were similar in terms of measures related to NC. (**Table 1**).

For peer review only - http://bmjopen.bmj.com/site/about/guidelines.xhtml

Table 1: Description of BOOST trial participants, those interviewed and those included in the qualitative analysis presented in this paper. Values are N (%) or mean [SD] or median {IQR} as specified

Participant Characteristics	BOOST trial cohort<br>N (%)<br>Mean [SD]<br>Median {IQR}	Interview cohort<br>N (%)<br>Mean [SD]<br>Median {IQR}	Interview analysis cohort<br>N (%)<br>Mean [SD]<br>Median {IQR}
Total participants	435	59	30
Age (years at baseline)	74.9 [6.0]	75.5 [6.2]	75.1 [5.7]
Gender			
Female	246 (56.6)	31 (52.5)	14 (46.7)
Male	189 (43.4)	28 (47.5)	16 (53.3)
Ethnicity			
White British/White Other	400 (92)	52 (87)	26 (86.6)
Black British	12 (2.8)	3 (5)	2 (6.7)
Indian	9 (2.1)	3 (5)	2 (6.7)
Pakistani	6 (1.4)	1 (2)	0 (0)
Confidence walking long distance	5.6 [3.3]	5.1 [3.3]	4.7 [3.0]
Oswestry disability index	32.9 [13.9]	32.7 [13.5]	34.6 [14.5]
Tilburg frailty index	4.5 [2.6]	4.8 [2.8]	5.6 [2.8]
Exercise self-efficacy, median {IQR}	69 {53, 80}	69 {43, 86}	52. 5 {37, 81}
Fear avoidance beliefs	12.9 [5.9]	14.0 [6.3]	14.1 [6.4]
Six-minute walk test	255.4 [99.1]	249.3 [96.9]	227.7 [91.9]
Self-management rating scale	6.08 [1.80]	6.0 [1.7]	6.1 [1.7]
**Treatment allocation in the trial[1]**			
BOOST programme (BOOST)	292 (67.1)	32 (53)	16 (53.3)
Best practice advice (BPA)	143 (32.9)	27 (45)	14 (46.7)
**Complete sets of three interviews**		49	30

Abbreviations: BOOST: Better Outcomes for Older people with Spinal Trouble; SD: Standard Deviation: IQR: Interquartile Range

Scale range and direction

Confidence in ability to walk long distance (1 to 10, lower values worse)
Oswestry Disability Index V2.1a (0 to 100, higher values worse)
Tilburg Frailty Index (0 to 15, higher values worse)
Exercise self-efficacy scale (0 to 90, lower values worse)
Fear Avoidance Beliefs Questionnaire Physical Activity (0 to 24, higher values worse)
Six-minute walk test (distance covered walking for 6 minutes, lesser distance worse)
Self-management rating scale (0 to 10, lower values worse)

For peer review only

Analysis results

Analysis steps 1 to 3: participant trajectories

From summarising patterns of change from our individual participant frameworks (**See Supplementary file 2** for an example) into our change framework (**Supplementary file 3**) we identified patterns of change for each participant in each domain (**Supplementary file 4**). We then summarised the trajectory across the three timepoints (see **Table 2)**. We omitted sleep as a domain as although a question on sleep was asked in each interview many interviewees did not provide data.

Table 2: Participant trajectories in the biopsychosocial domains

For peer review only

For peer review only - http://bmjopen.bmj.com/site/about/guidelines.xhtml

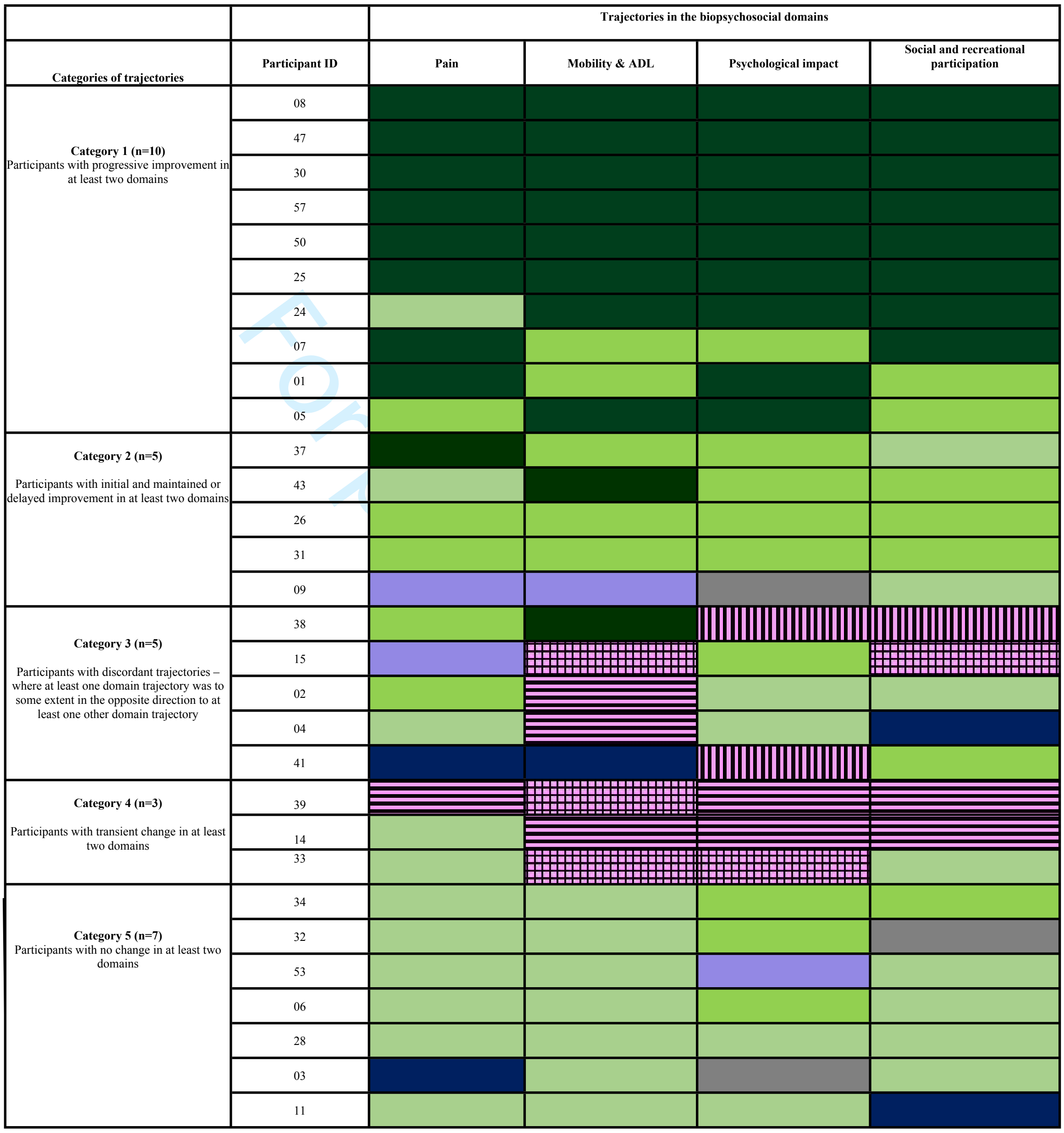

ADL: Activities of Daily Living

For peer review only - http://bmjopen.bmj.com/site/about/guidelines.xhtml

Table 2: Participant trajectories in the biopsychosocial domains

Categories of trajectories	Participant ID	Pain	Mobility & ADL	Psychological impact	Social and recreational participation
**Category 1 (n=10)** Participants with progressive improvement in at least two domains	08				
	47				
	30				
	57				
	50				
	25				
	24				
	07				
	01				
	05				
**Category 2 (n=5)** Participants with initial and maintained or delayed improvement in at least two domains	37				
	43				
	26				
	31				
	09				
**Category 3 (n=5)** Participants with discordant trajectories – where at least one domain trajectory was to some extent in the opposite direction to at least one other domain trajectory	38				
	15				
	02				
	04				
	41				
**Category 4 (n=3)** Participants with transient change in at least two domains	39				
	14				
	33				
**Category 5 (n=7)** Participants with no change in at least two domains	34				
	32				
	53				
	06				
	28				
	03				
	11				

Legends for trajectories

No change: No change in pain or in the decreased mobility decreased social and recreational participation or negative psychological impact experienced since before the onset of NC.

Progressive worsening: Progressive worsening of pain, decreased mobility, decreased social and recreational participation, or negative psychological impact (i.e. continued worsening after an earlier worsening).

Improvements remained same: Improvements experienced at T1 (compared to entry into trial) or at T2 (compared to T1) in pain, mobility, social and recreational participation, or psychological impact were maintained.

Progressive improvements: Progressive improvements in pain, mobility, social and recreational participation, or psychological impact (i.e. continued gain on an earlier improvement).

Transient change pattern 1: Initial improvements between T1 & T2 then reversed to T1 (Baseline) status at T3.

Transient change pattern 2: Initial improvement between T1 & T2 & then worsening between T2 & T3, to worse than T1

Transient change pattern 3: Initial worsening between T1 & T2 & then improving between T2 & T3

Delayed improvement: Pain, mobility, social and recreational participation, or psychological impact improved at T3 only.

Lack of sufficient information (at least 2 time points) to describe the change process.

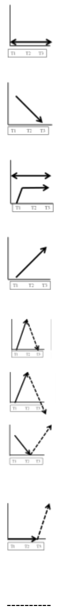

For peer review only - http://bmjopen.bmj.com/site/about/guidelines.xhtml

Progressive improvement in at least one domain was experienced by a total of 13 participants. Trajectories of no change, early improvements that remained the same and transient changes predominated. Of the 30 participants, 16 experienced no improvement in at least one domain. Improvement in pain was experienced by 16 participants. All 15 participants who experienced improved social and recreational participation also reported improvements in other domains (although for one the improvement in another domain was after initial worsening).

The trajectories in any one domain are not an indication of the amount or nature of change so the same trajectory might be experienced very differently. For example, participant 24 and participant 50 both showed progressive improvement in mobility, but one was able to walk much further than the other:

> *And as I said, I can go out and I can go off to different parts of the shops, or garden centres, we like looking round those.* (24, T3)

> *I am able to get up and walk without any aids around it* [home] *now* (50, T3).

In the domain of psychological impact, participant 04 and 02 continued from T1 to T3 with negative but different experiences. The first experienced frustration as he had to rely on his wife to do his self-care activities:

> *I can't get myself dressed, I can't do the things that I would normally do or anybody else would normally do for themselves. I need a hand; I need help with that, which I do get obviously, with [wife]. Again, that's frustrating because she's got things to do and I don't feel I can keep saying to her, oh can you just help me with this, can you help me with that?* (04, T3)

The second describes fear of damaging his back with certain activities:

> *But I don't do gardening like I used to do before. For the fear of damaging or hurting my back, and things like that. Because I tried that in the past, and once I start doing something then I get carried away, and the next two days then I have to suffer, so I try and avoid it* (02, T3).

In the domain of social and recreational participation, participants 07 and 24 experienced progressive improvements over time but with different reach. The first was prepared to extend her activities:

> *But wherever I go, if I go on holiday as well, I never say no, that I can't do (some)thing* (07 T3)

The second was positive about her ability to undertake small tasks that helped her feel she was participating equally with others. She described social outings with her friends:

> *I can carry a tray with drinks on now. I can take my turn, I can get in the queue, where it always used to be 'you get the table, I'll go* (24, T3).

When placing participants adjacent to other participants based on the similarity of trajectory, we were making choices about which trajectories to prioritise. Different choices resulted in a different ordering of participants. For example, we could place together participants with two or three domains with the same trajectory and not worry about the trajectory of the other domain(s), or we could place together participants with the same number of trajectories of a certain type, irrespective of which domains they were. However, participants tended to

For peer review only - http://bmjopen.bmj.com/site/about/guidelines.xhtml

remain towards one end or the other of the table. Table 2 presents one way of ordering the participants on which the team could agree.

Our visual impression was there were five main patterns of trajectories which we identified as our trajectory categories, although the boundaries of these are not clear cut. They were: category 1 (n=10) participants with progressive improvements in at least two domains; category 2 (n=5) paticipants with initial and maintained or delayed improvements in at least two domains; category 3 (n=5) participants with discordant trajectories – where at least one trajectory was to some extent in the opposite direction to at least one other trajectory (the opposite direction might be transient); category 4 (n=3) participants with transient change (in the same direction) in at least two domains; category 5 (n=7 participants with no change in at least two domains. We illustrate the trajectories for one participant at each time point with quotations from their data in **Table 3** and for one participant from each trajectory at T3 in **Table 4**.

For peer review only

For peer review only - http://bmjopen.bmj.com/site/about/guidelines.xhtml

Table 3: Illustrative quotes for one participant [Category 3] for all domains and time points

ID:04	**Pain**	**Mobility and ADL**	**Psychological impact**	**Social and recreational participation**
T1	*'And then, umm, there's pain if I get up, there's pain if I walk, there's pain sitting down, there's pain standing up, there's pain reaching out… it's a constant pain. 24/7 I'm in pain'*	*'I'm inactive, I can't do anything without being in pain…'*	*' So I started to get depressed then, because I couldn't... I can't do anything and I couldn't do anything and I, I just sort of thought, 'Well what's the point, what is the point of me being here when I can't do anything?' This is it, I'm just sitting around, I'm doing nothing, I'm no good …'*	*'Oh I love to see the grandkids but, again, I can't do anything with them, I can't do nothing with the grandkids because, again, the... My grandson's into football now and I feel there's a lot I could teach him with the, with the game and I can't.'*
T2	*'Not really, it's* [Pain] *there the majority of the time.'*	*'Well every day, every day. I mean we're either out shopping or that, I don't think there's a day goes by where I'm not doing some sort of walking.'*	*'I'm in a situation where I'm still depressed over it because obviously I'm not … I didn't expect a lot from it, I suppose in my mind I thought it might be, I might be having a lot more movements and things like that, which haven't worked out.'*	*'Socialising I don't do. I'm not a socialiser anyway. All them days … since the football finished and everything I'm not, I'm not one for going out and that anyway. As I said, sometimes it's not worth the effort to get ready and. Getting washed and changed and then getting out like that, so much is involved with it, sometimes it's not worth doing it'*
T3	*'Well I'm still in a lot of pain….*	*'I feel more confident when I'm in the house. I mean while the weather's like this I won't budge out at all, I won't venture out at all, there's no point….Obviously, when it's really severe, I can't get myself dressed, I can't do the things that I would normally do or anybody else would normally do for themselves'*	*'I mean, I keep bringing the mental side of it because I think everything that goes on does affect you mentally. It's very hard not to get away from a lot of things mentally'*	*'I said, my one grandchild is playing football himself now and I can't, there's no way I can go with him and help him or be involved in his, in coaching with him or teaching him anything or just having a kick around with him. I can't do that, so I feel I'm missing out on all that.'*
**Trajectory**	No change	Initial improvements between T1 & T2 then reversed to T1 (Baseline) status at T3	No change	Progressive worsening

For peer review only

For peer review only - http://bmjopen.bmj.com/site/about/guidelines.xhtml

Table 4: Illustrative quotes of participants from each trajectory category at T3

Participants from trajectory categories	Pain	Mobility and ADL	Psychological impact	Social and recreational participation
**Category 1** ID:08 White British Male, 70 years	*'But in terms of my back pain and legs, although I still have a little and last night it was particularly uncomfortable, most of the time it actually is fine, and I feel a lot better than I did last year.'*	*'If I go for a walk, for example, with my wife, I can probably do quite a long-ish walk, probably three to five miles, which I couldn't do 12 months ago.'*	*'But, for example, to give you a clue of how more confident I am, we are going away for five days on Sunday to [city] to do some sightseeing, some walking, and some photography. So I feel confident enough to be able to go and do that.'*	*'… we went down to* [Town] *for a week* [Holidays], *back in November and the apartment we had was about a mile and half out of the town centre. So we walked that both ways, in and out, most days, and I coped with that quite well.'*
Category 2 ID:31 White British Female, 81 years	*'Oh it is better. It's under control, as much as I can get it under control, with the help of the Gabapentin, which, I know it's another drug to be on but if it works then.'*	*'I walk down the stairs and up the stairs, rather than use the [stair lift]. And I walk up and down the hall quite a bit, I do an exercise where I just walk up, turn around and come back, I do that about ten times. Particularly if it's a wet day and I can't get out.'*	*'Well, I'm walking better. Whereas before I was getting frightened to walk, because I was in so much pain.'*	*'Well, I tend to go in* [Gardening] *for a short while. Once my back starts hurting, I come back in and sit down. I don't overdo it generally.'*
**Category 3** ID:04 White British Male, 65 years	*"Well I'm still in a lot of pain, but not as much as I was last week. It's sort of calmed down a little bit now."*	*"Well, I don't cook for myself or anything like that. I mean, I can't mess around with things like that. Obviously, when it's really severe, I can't get myself dressed, I can't do the things that I would normally do or anybody else would normally do for themselves."*	*"I mean, I keep bringing the mental side of it because I think everything that goes on does affect you mentally. It's very hard not to get away from a lot of things mentally."*	*"Normally I miss out on a lot of things because of sitting somewhere for too long or something because this again causes discomfort. So rather than sit there feeling miserable I'd rather not go."*
**Category 4** ID:14 White British Female 77 years	*'At the moment I think I'm at a bad stage, because I've got so many things going on ... I've got the back pain. I've got the neck pain. I've got the knee, which comes out of joint. So it's not a pretty sight really.'*	*'But I still make myself, when we've got a good day, not today like it is now, but if it's a good sunny day I will still try to go out and do about ten minutes, fifteen minutes' walk.'*	*'I was doing very well and very eager to get better or help myself somewhat … So I've got all these things* [other musculoskeletal issues] *going at once. Which has dragged me down a bit, there's no doubt about it, it's made me quite depressed.'*	*'It's obviously; it's something I'm not used to. I'm used to going everywhere and very, sort of, doing exercises and yoga classes and everything else. And it's getting worse, because it's stopped, it's stopping, everything is going wrong.'*
**Category 5** ID:11 White British Male 72 years	*Once I've got my legs, once I'm moving a bit, it does seem to ease off, I'm not so bad. But if I sit down, then once I get up again it's back to square one; you're still in pain and tingling.'*	*'So it's hard for me to put, you know, for me to actually sit still all day. In the last week, I've watched more telly than I've watched in 12 months. So you can tell, like, the situation. I've never watched so much, I've got square eyes.'*	*'Well at the moment I'm more anxious than ever with what I've got now. So I'm still a bit anxious, well even before this, before this I was still anxious. I was quite anxious really, you know, because I wanted to do things.'*	*'And so it's really, my lifestyle really, to me, it's gone to nil from what I - I'd be out doing everything sort of thing, nine o'clock, ten o'clock at night time gone by, til it got dark, pottering about, cutting the lawn, doing the lawn, or doing the neighbour's if they couldn't do it, elderly people I'd cut theirs for them. So it's, to say, like, you've come to, not nil as such, but you've really, it's a total cut off …'*

This categorisation was developed from one way of ordering the participants (see above). It was therefore expected that some participants could be in more than one category (for example participant 34 could be in category 2 or 5).

We chose to define category 3, discordant trajectories, as trajectories going in opposite directions (improving/worsening). Arguably, a no change trajectory alongside a transient change or an improving/worsening trajectory is also discordant. All but one of the 10 participants in categories 4 and 5 have this latter combination of trajectories, and four of the 15 participants in categories 1 and 2.

Of the 15 participants who experienced improvement in social and recreational participation, ten experienced improvements in all other domains and were in categories 1 and 2. The

For peer review only - http://bmjopen.bmj.com/site/about/guidelines.xhtml

remaining five participants (24, 43, 38, 41 and 34) did not experience improvement in all other domains and were from across all categories except category 4.

Analysis step 4: nature of the trajectories

Co-presence of domain trajectories and perceived interaction

We identified ten types of co-presence of the trajectories (**Table 5**). The most common presentation was Type 1 where positive trajectories were co-present for all domains. Our data suggests improvement in one domain often led to improvement in another and so on (see illustrative quotes Table 5). Type 2 (n=3) was similar except those improvements in pain, mobility and psychological were not co-present with improvements in social and recreational participation. The reasons for this were very different and specific to each individual: participant 09 was a 79-year-old farmer who was unable to get back to his heavy farm work, participant 37 was an 88-year-old who did not get back to going on group trips which involved walking as he did not want to hold up the others and 01 was an 81 year old who at baseline had already managed to return to Tai chi and her voluntary work and maintained this. All the participants of type 1 and 2 were in trajectory groups 1 and 2 (improvement trajectories) except participant 38 who was in trajectory group 3 as they had transient decline before improvement in two domains. This participant gained mobility and social and recreational participation through learning to effectively use a walking aid.

For peer review only - http://bmjopen.bmj.com/site/about/guidelines.xhtml

Table 5: Co-presence of trajectories (⇑ improving; ⇓ worsening; ⇔ no change) and illustrations of how they interact (or not)

Co-present trajectories of biopsychosocial domains	Participant ID	Participants quotes illustrating interaction between co-present trajectories	Trajectory Category (see table 2)
1) ⇑Pain, Mobility, Psychological impact, Participation	30, 31, 07,08, 25, 38, 05, 26, 57, 47, 50	*'When you first saw me, I was in a bit of pain, and everything was a struggle. But now, I take everything in my stride now ... I think at least 75% I feel better in myself* **[Pain]**… *Oh I'm quite satisfied* [with walking] *at the moment. As I say, a couple of miles is easy now, when I go out shopping or anything like that* **[Mobility & ADL]…***And then as I say, Wednesdays and Thursdays I go out to the club, have a drink, and socialise with my friends. And Saturday night I go out, get on the dance floor if I feel up to it, for half an hour or something like that* **[Participation]**…*But as far as, it* [Neurogenic claudication] *doesn't stop me doing anything I want to do'* **[Psychological impact]** (30, T3)	1 and 2<br>Improving
2) ⇑Pain, Mobility, Psychological impact ⇔ Participation	01, 09, 37	*'I really haven't had any problems with my back* (37, T2) **[Pain]**…*I don't think there is any problem with my general health…I think I am walking better than I was when I started this scheme…I suppose I have really not a lot of problem, I can walk the distances that I need to now, I don't want big distance… I think it* [Walking uphill] *is easier…it has been easier before when I first met you, if I walked uphill, I felt exhausted…now I don't* **[Mobility & ADL]**…*I design my life around my abilities* **[Psychological impact]**…*Not a lot, to be fair* [impact of condition on lifestyle]…*I keep myself involved that is my objective to keep myself occupied…To be fair, I think I work my physical life to the limitations I have got and being my age… I don't suppose I can say it* [General lifestyle] *altered other than doing exercises in the morning'* **[Participation]** (37, T3)	1 and 2<br>Improving
3) ⇔ Pain ⇑ Mobility, Psychological impact, Participation.	43, 24	*'No, just through the night* **[Pain]**… *I'm getting around fine…I just feel good* **[Mobility & ADL]**. *Makes me feel good, it gives me confidence. You see I can do it, and now I know I can do it* **[Psychological impact]**… *I can carry a tray with drinks on now. I can take my turn, I can get in the queue, where it always used to be 'you get the table, I'll go'* **[Participation]** (24, T3)	1 and 2<br>Improving
4) ⇓ Pain, Mobility, Psychological impact, Participation (except 41 where improved Participation gained by remained the same)	03, 11, 33, 41, 28	*'… once I've got my legs, once I'm moving a bit, it does seem to ease off, I'm not so bad. But if I sit down, then once I get up again it's back to square one; you're still in pain and tingling* **[Pain]**…. *'I'm really basically, not doing nil, but you know, walking up and down the garden and walking, not really doing to what I normally do. It's really halved* **[Mobility & ADL]**…*Well at the moment I'm more anxious than ever with what I've got now. So I'm still a bit anxious, well even before this, before this I was still anxious. I was quite anxious really, you know, because I wanted to do things* **[Psychological impact]**…… *And so it's really, my lifestyle really, to me, it's gone to nil from what I - I'd be out doing everything sort of thing, nine o'clock, ten o'clock at night time gone by, till it got dark, pottering about, cutting the lawn, doing the lawn, or doing the neighbour's if they couldn't do it, elderly people I'd cut theirs for them. So it's, to say, like, you've come to, not nil as such, but you've really, it's a total cut off'* **[Participation]** (11, T3)	5<br>No change
5) ⇔ Pain ⇓ Mobility, Participation (or not mentioned). ⇑ Psychological impact	53, 06,	*'Still I am in some difficulty and problem* **[Pain]**… *but….honestly I just carry on with my life* **[Psychological impact]**…. *…It* **[Pain]** *is not too bad at the moment….To be honest, I don't do anything* **[Mobility & ADL]** *at all…Some days…bad days which you know you just wonder what else you can do… Housework otherwise, I take my time. I don't try to push myself to you know everything I need to suppose one time...Little at a time* **[Mobility & ADL]**…*I don't worry about it I just try on and carry on with my life* **[Psychological impact]**…. *I do that* [Shopping] *not alone but always have somebody with me'* **[Participation]** (06, T3)	5<br>No change
6) ⇔ Pain ⇓ Mobility ⇑ Psychological impact, Participation	34	*'Could* **[Pain]** *be better. I can feel my sciatic nerve all the time* **[Pain]**… *…but it didn't really bother me, you know* **[Psychological impact]** …. *I can't walk as quick as I used to and I can't do steps…I am walking normally but the smaller steps what I used to do… My legs hurt when I start moving around…there are certain movements I just be careful with…but other than that, fine* **[Mobility & ADL]**, *I am doing my garden. My back doesn't like it very often…You know I come in, sit down, have a cup of tea and go back again. I go to my gardening club...Tuesday I see my friend as usual…put the world right and have a cup of coffee…today I had been to school….so listen to the children reading and now being given an award today* **[Participation]**……*You know you just have to get on with it, don't you? I can't go anywhere, I feel shattered, but you still have to do don't you? If you got to do something, you got to do it'* **[Psychological impact]** (34, T3)	5<br>No change

For peer review only - http://bmjopen.bmj.com/site/about/guidelines.xhtml

7) ⇑ Pain ⇓ Mobility, Psychological impact ⇔ Participation	02	*'But my back pain, the constant pain and the severe pain that I used to have is no longer there at the moment, so I'm happy to say that. However, it is not completely gone, and I get days, every now and again, when the pain comes back* ***[Pain]****.... What happens is then the pain starts, not while I'm walking but after I have done my walk and I sit down. And the more I walk, the more severe the pain is when I sit down* **[Mobility & ADL]**... *'It's not the ideal situation, because it makes me little bit home bound and I cannot travel as freely as I would like to, long distance and things like that* **[Participation]***...this summer I had high hopes of doing a lot of other things, to improve my back and things like that, but with this balloon sticking from my stomach I can't even go swimming or cycling... Cycling might be a bit too strenuous. Although I have been told that people do go swimming with this type of tube, but I don't want to take that chance'* **[Psychological impact]** (02, T3)	3<br>Discordant
8) ⇑Pain ⇓ Mobility ⇔ Participation<br>⇑ Psychological impact before T3 spinal fusion	15	*'I got no pain at all now…I am pain free in my back* **[Pain]***… …I got a walking frame that I can with difficulty walk around the house* **[Mobility & ADL]** *I say 'with difficulty' because at times my right leg just tend to buckle slightly, because the right leg is by far the worst one…it was by far the worst affected it does tend to buckle …and so either my wife or the carer when they come, they escort me, it is they don't have to do anything, they are there as a safety precaution* **[Mobility & ADL]***.... All in all, it's been a fairly traumatic sort of time…I keep myself in good spirits…well its happened what is the point of being miserable about it, I keep myself in good spirits and carry on'* **[Psychological impact]***...Not doing any physical activity, obviously my body is not getting tired....Obviously at the moment, is the rest and recuperation time…'* **[Participation]** (15, T3)	3<br>Discordant
9) Pain predominantly from other MSK problem.<br>⇓ Mobility, Psychological impact, Participation (or not mentioned)	04, 14, 39	*'At the moment I think I'm at a bad stage, because I've got so many things going on ... I've got the back pain. I've got the neck pain. I've got the knee, which comes out of joint. So it's not a pretty sight really* **[Pain]***.... I'm used to going everywhere and very, sort of, doing exercises and yoga classes and everything else. And it's getting worse, because it's stopped, it's stopping, everything is going wrong.... So I've got all these things going at once* **[Mobility & ADL and Participation]***. Which has dragged me down a bit, there's no doubt about it, it's made me quite depressed'* **[Psychological impact]** (14, T3)	4<br>Transient change
10) Pain predominantly from other MSK problem.<br>⇓Mobility, Participation (or not mentioned) ⇑Psychological impact	32	*'Terrible* **[Pain]** *at the moment…Such a lot of pain in my knee then I have got pain in my right buttock which is more like… it is the sciatic nerve is trapped and I can't sit for longer I have to keep putting the pillow under or keep lying down…Lying down flat makes my back worse...its* [Hip pain] *been on and off a couple of years…some days its* [Back pain] *been worse, I can't get washed and dressed… Its pain everywhere…there is nothing you can do till you get repaired* **[Psychological impact]***…I can't do any housework or anything because I can't stand without hanging on to my trolley… Next week I am starting meals on wheels…It is non-existent really* [General lifestyle] *I could do a few things but not now...it is walking* [reason] *I wouldn't want to go far because it gets too painful, I walk to the car'* **[Mobility & ADL and Participation]** (32, T3)	5<br>No change

ADL: Activities of Daily Living; MSK: Musculoskeletal

In the group with co-presence type 3, where there was no improvement in pain but improvement in all other domains, participant 43 used pain relief for the persisting pain enabling other domains to improve even though he was not keen on taking painkillers, and 24 experienced pain only at night. Both were in trajectory groups 1 or 2.

In type 4 (n=5) all domains were worsening. This was the second most common type of co-presentation and mirror image of that observed in Type 1. Worsening in one domain had a negative impact on the other domains. All participants were in trajectory group 5 (no change in at least 2 domains) except participants 33 and 41. The former experienced transient improvement in two domains but then worsened, so was in trajectory group 4. The latter was in trajectory group 3 where there was discordance. This participant had worsening pain and mobility which she had found depressing but by T3 she saw the pain, although severe, as manageable. The level of mobility required for her to maintain most of her

For peer review only - http://bmjopen.bmj.com/site/about/guidelines.xhtml

recreational and social interests was relatively limited and she could manage this through planning, but she had difficulty with activities involving walking in groups.

Type 5 (n=2) was similar to type 4 and both participants were in trajectory group 5 but there was improvement in psychological impact. Participant 53 had specifically addressed his depression that had resulted from the NC symptoms and 06 had adapted to the pain. However, in the timeframe of the study the reported improved psychological impact did not influence the other domains.

Participant 34 with co-presence type 6 where pain did not improve and mobility deteriorated (trajectory group 5) similarly maintained positive psychological outlook but was also able to maintain social and recreational engagement despite the pain and loss of mobility.

Participant 02 (co-presence type 7) and 15 (co-presence type 8) were both in trajectory group 3 where there was discordance. The former remained envious of those who could walk faster than he could which impacted negatively on his psychological wellbeing and would have liked to be able undertake long distance travel which was limited by his pain. Participant 15 had spinal surgery between T2 and T3 which raised his hopes of improvement although had not yet experienced improvement.

Co-presence types 9 and 10 were similar to types 4 and 5 respectively but the pain was not from NC but from other musculoskeletal problems.

We were particularly interested in those who still improved in social and recreational participation without improvement in all other domains. This was experienced by four participants for a variety of reasons highlighting those ongoing impairments across other domains does not necessarily impede improvement in participation. For 24 the pain was only at night, for 43 the pain could be controlled, and for 34 and 41 it was their positive psychological outlook.

Turning points

Thirteen participants did not mention a major change in their health. Twelve described major change in musculoskeletal problems: four described notable improvement or worsening of their pain and mobility, one had a fall that impacted wellbeing, four described worsening of joint problems (hip, knee, and neck), and four had back surgery during the data collection year. Four participants developed other major health issues (emergency abdominal surgery, Addison's disease, chronic obstructive airways disease, insertion external feeding tube). From the data for these latter participants, we were unable to detect any interaction between their trajectory and these health events.

Totality of change

There were many similarities between participants from across the different trajectories. The majority of participants lived with a partner with a total of 9 living alone. The majority of participants (from all categories) had a positive outlook on aging and wanted to be active, '*…Age is just a*

For peer review only - http://bmjopen.bmj.com/site/about/guidelines.xhtml

number, for me' (08, T1, 70 years). A few either had an acceptance of aging or had negative attitudes, *'You'd think I was 95 the way I'm talking to you'* (11, T2, 70 years). Most participants (from all categories) were using some form of mobility aids ranging from walking stick, wheeled walker and rollator, Nordic poles, Zimmer frame, and mobility scooters. A very few (all categories) mentioned they didn't want to become dependent on mobility aids or look older, *'I don't like them [laughs]. Vanity. I just suppose … It looks like you are an old man you know, or a disabled person'* (02, T1). In all categories, good weather/summer season enabled walking and outdoor activities and many reported limited activity during winter due to difficulties such as lack of confidence in walking when wet/icy and difficulty finding places to sit down. All participants reported a variety of self-made, convenient strategies for coping with their NC in at least one of their interviews. Participants predominantly used active, problem-focused strategies rather than passive ones such as avoiding painful activities. For example, they used solution-based strategies such as sitting/resting between walks or household chores, driving to local shops instead of walking, leaning on a shopping trolley to walk around, using a light-weight vacuum cleaner, and accepting help from family members with shopping and gardening. Participants who regularly (mostly daily, one 2-3 times a week) engaged in exercises specifically for their NC were found in all trajectory groups.

Discussion

For each person living with NC, we were able to identify the pattern of change at each of the three time points for each of the biopsychosocial domains of pain, mobility, psychological impact and social participation. By looking across these patterns we were able to identify trajectories over time for each domain. Participants in all trajectory categories had many attributes in common including attitudes to aging and in seeking strategies to enable themselves to function optimally.

Although there were participants where improvement occurred across all domains or there was no change across all domains, many participants reported discordance with change going in different directions in different domains, or there was no change in some domains alongside change in other domains. Published quantitative evidence has demonstrated within domain discordance: for mobility, discordance between walking capacity and actual walking (32, 33). With our qualitative data, we were able to illustrate interaction between domains, for example, pain affecting mobility, with negative psychological impact and social withdrawal and vice versa. This negative cycle might explain the within domain discordance for mobility. However, we were also able to illustrate how, for example, improvement in psychological impact or social and recreational interaction happened despite pain or mobility problems.

For ten out of fourteen participants reporting improvement in social and recreational participation, this occurred alongside improvement in all other domains. This is the best scenario and the aim of interventions for people with NC. Remaining participants reported dealing in some way specifically with their pain, mobility or psychological impact which still enabled them to improve social and recreational participation without

For peer review only - http://bmjopen.bmj.com/site/about/guidelines.xhtml

improvement in all domains. This suggests that it is possible to identify strategies to increase participation even if other domains are resistant to change. This is particularly important when considering the impact of pain. Pain is particularly problematic for this patient group and effective treatments for reducing the pain associated with NC are limited (34). For the participants where improvement in social and recreational participation was not achieved when it might be expected the reasons were: very high expectations of what for them counted as participation; did not want to cause a problem for others; there no loss of engagement to be regained. The other group warranting further consideration are those reporting worsening across all the domains and what can be done to break this perpetuating negative cycle.

Categorising and understanding change over time

Our study extends the limited current longitudinal qualitative evidence on living with back pain. We found a greater variety of trajectories than in a published study with just eight participants (see background) (12) as, in contrast to this study, we found examples of change in one domain despite no change in others as well as examples of reinforcement of change across domains. The earlier study collected data at longer time intervals (12 months over 2 years). This may have resulted in less detail about change between data points than in our study resulting in the perception of smoother trajectories. In our past qualitative study (22) which included 15 participants living with chronic back pain (excluding NC) interviewed three times in two years, we developed the following categories of participant's current experience of their back pain: past reminders (similar to fear avoidant behaviour), stuck and struggling, becalmed, and submerged. These categories describe the overall state of the participants rather than interactions that resulted in these states. Of the 15 participants, 12 changed category during the 2 years. Most of the participants started as stuck and struggling and moved to past reminders or becalmed, trajectories similar to our current study participants in categories 1 and 2. Two participants remained stuck and struggling/submerged, a similar trajectory to the participants at the bottom of our Table 2 (trajectories of no change/worsening). Our past study included cross-sectional data only from people living with diabetes (22) who were categorised in a similar way to those with back pain.

Trajectories have been identified in qualitative studies of people living with long-term conditions other than back pain where longitudinal data has been collected. In a study of 22 participants, data was collected 15 months post stroke and over the subsequent 4 months. Four different recovery trajectories were evident: (a) meaningful recovery, (b) cycles of recovery and decline, (c) ongoing disruption, (d) gradual, ongoing decline (35). The first (a) is similar to our progressive improvement trajectory, (b) may be similar to our discordant trajectories, (c) and (d) have some similarity to our category of no change; the difference may be due to the natural history of the NC where NC only worsens for a few (6). However, trajectories identified depend on the focus of analysis. A longitudinal interview study with 21 participants diagnosed with a chronic illness (e.g., diabetes, rheumatoid arthritis) focused on illness self-management (36). It was found participants go through the following phases: seeking effective self-management strategies, considering costs and benefits, creating routines and plans of action, and negotiating self-management that fits one's life. In our study we found all these strategies being used but our analysis focus was not self-management.

For peer review only - http://bmjopen.bmj.com/site/about/guidelines.xhtml

Categorising to predict outcome and complex interaction

We found many similarities between the participants in the 5 categories of trajectories and a lack of features that could be used to identify members of a particular trajectory which could be used to predict outcome. Many studies on back pain have analysed quantitative data using methods for categorising such as latent class analysis, seeking categories of patients that would allow matching of interventions to patient groups (37-39). Study participants can be categorised into clinically meaningful groups and category membership may correlate with outcome (40). However, interaction between category membership and intervention is difficult to demonstrate (38). A longitudinal study of spinal stenosis found factors predictive of subjective improvement, but none were modifiable (41). If improved social and recreational participation is our goal, our study would suggest that this is likely to be co-present with and interact with improvement across the domains of pain, mobility and psychological impact and not improve when there is no co-presence of improvement across the other domains. This interaction between domains is like that described in a cross-sectional interview study with people living with NC (5). Although our data suggests that for some participants improvement in one domain leads to improvement in another domain, this interaction is in the timeframe of hour-to-hour or day-to-day or week-to-week as occurs in complex systems (42, 43). This, what we have called co-presence, is different from the prediction of outcome over many months or years that is sought in the studies described above. However, our study also provides examples of participants where one factor did prevent or enable improvement, but this was a relatively small number of participants and the attributes varied. We suggest that seeking individual level factors that predict of outcome for people living with NC is unlikely to be helpful. However, we need to remain alert to external determinants of outcome. For example, in a US study, type of health insurance influenced rate of improvement for people with low back pain (44).

<u>Strengths and limitations</u>

We collected a relatively large qualitative data set with higher-than-expected participation rates in later interviews. This may have been because the repeat encounters generated a relationship of trust between researcher and participant (45). Although there are differences when interviews are conducted by telephone rather than face to face (46) our telephone interviews provided rich data for our analysis, perhaps helped by the first interview being face-to face

At each interview we focused on how the participant was at the time of the interview. However, in interviews people naturally tell stories and these stories include accounts of change that has occurred (47). What was current was sometimes difficult to disentangle and we may have interpreted later data differently given our understanding of the participant from earlier interviews (23).The data we included in the current analysis was from participants providing three interviews of rich data so they may differ from those in our study providing less data. As we proceeded through data analysis, we chose our path based on our interest in the complex nature of health, the lived experience of conditions such as NC and successful aging. Alternative data analysis paths through this data set are possible. Analysis could be tested further by interrogating the remaining unanalysed data. Reduction of data to mini statements loses richness and nuances in data but we retained the ability to go back directly to the framework and the data for fuller summaries and illustrative quotes. Data reduction enables comparison across the whole data set.

For peer review only - http://bmjopen.bmj.com/site/about/guidelines.xhtml

Although a longitudinal study, follow up was only for 12 months, a relatively short time. NC symptoms change over time. Some of our participants had already started to experience improvement in their symptoms at the start of the study. This is in line with findings of a cohort study of people with moderate NC(6).

Participants could be categorised in different ways and there was overlap between categories. This is a common finding when categorising people in both qualitative studies (22) and quantitative studies (37).

Implications for clinical practice

There may be benefit in those providing therapy for NC patients to identify the trajectory of their patient in order to tailor treatment. This requires careful listening as changes may be quite small so hard to discern. Although it is useful to categorise patients as we have done in this study, these categories are just useful labels to think with and guide discussions with patients. As therapists know, patient presentation can often seem messy. Having the categories of trajectory and how they potentially interact in mind may help tease out what is going on for people and identify where there is opportunity to reinforce positive change or modulate deterioration, but we need to beware of shoehorning people into categories as we could miss important changes in trajectory in one or more domains. Our study suggests therapists, while following clinical guidance for patients with NC, also need to individually tailor their interventions for patients to optimise participation in social and recreation activities that are fundamental to healthy aging.

For peer review only - http://bmjopen.bmj.com/site/about/guidelines.xhtml

References

1. Williamson E, Sanchez Santos MT, Morris A, Garrett A, Conway O, Boniface G, et al. The Prevalence of Back and Leg Pain and the Cross-sectional Association With Adverse Health Outcomes in Community Dwelling Older Adults in England. Spine (Phila Pa 1976). 2021;46(1):54-61.
2. Ishimoto Y, Yoshimura N, Muraki S, Yamada H, Nagata K, Hashizume H, et al. Prevalence of symptomatic lumbar spinal stenosis and its association with physical performance in a population-based cohort in Japan: the Wakayama Spine Study. Osteoarthritis Cartilage. 2012;20(10):1103-8.
3. Suri P RJ, Kalichman L, Katz JN. . Does this older adult with lower extremity pain have the clinical syndrome of lumbar spinal stenosis? JAMA. 2010;Dec 15;304(23):2628-36.
4. Lin SI, Lin RM, Huang LW. Disability in patients with degenerative lumbar spinal stenosis. Arch Phys Med Rehabil. 2006;87(9):1250-6.
5. Ammendolia C, Schneider M, Williams K, Zickmund S, Hamm M, Stuber K, et al. The physical and psychological impact of neurogenic claudication: the patients' perspectives. J Can Chiropr Assoc. 2017;61(1):18-31.
6. Wessberg P, Frennered K. Central lumbar spinal stenosis: natural history of non-surgical patients. Eur Spine J. 2017;26(10):2536-42.
7. Rowe JW, Kahn RL. Successful aging. Gerontologist. 1997;37(4):433-40.
8. Katz S CT. Critical perspectives on successful aging: Does it "appeal more than it illuminates"? The Gerontologist. 2015 Feb 1(1):26-33.
9. Foster L WA. Active and Successful Aging: A European Policy Perspective. The Gerontologist. 2014;Feb 1(1):83-90.
10. Martinson M BC. Successful aging and its discontents: A systematic review of the social gerontology literature. The gerontologist. 2015;Feb 1(1):58-69.
11. Snelgrove S LC. Living with chronic low back pain: a metasynthesis of qualitative research. Chronic illness. 2013;Dec: 9(4):283-301.
12. Snelgrove S ES, Liossi C. A longitudinal study of patients' experiences of chronic low back pain using interpretative phenomenological analysis: changes and consistencies. Psychology & health. 2013;Feb 1(2):121-38.
13. Kelley-Moore JA SJ, Kahana E, Kahana B. When do older adults become "disabled"? Social and health antecedents of perceived disability in a panel study of the oldest old. Journal of health and social behavior. 2006;Jun: 47(2):126-41.
14. Corbin J. Accompaniments of chronic illness: Changes in body, self, biography, and biographical time Res Sociol Health Care. 1987;6:249-81.
15. Bury M. Chronic illness as biographical disruption. Sociology of health & illness. 1982;4(2):167-82.
16. Anjum RL CS, Rocca E. Rethinking causality, complexity and evidence for the unique patient: a CauseHealth Resource for healthcare professionals and the clinical encounter. 2020.
17. Cilliers P. Difference, Identity and Complexity. In: Cilliers P, Preiser R, eds. Complexity, Difference and Identity Issues in Business Ethics. Dordrecht: Springer; 2010.
18. Griffiths F BJ, Byrne D, Crabtree BF, Dowrick C, Gunn J, Kokanovic R, Lamb S, Lindenmeyer A, Parchman M, Reis S. . Developing evidence for how to tailor medical interventions for the individual patient. Qualitative Health Research. 2010;Dec: 20(12):1629-41.

For peer review only - http://bmjopen.bmj.com/site/about/guidelines.xhtml

19. Weller S ER, Jamieson L, Davidson E Analysing large volumes of complex qualitative data; reflections from a group of international experts. 2019.
20. Ward L, Williamson E, Hansen Z, French DP, Boniface G, Rogers D, et al. Development and delivery of the BOOST (Better Outcomes for Older adults with Spinal Trouble) intervention for older adults with neurogenic claudication. Physiotherapy. 2019;105(2):262-74.
21. Williamson E, Ward L, Vadher K, Dutton SJ, Parker B, Petrou S, et al. Better Outcomes for Older people with Spinal Trouble (BOOST) Trial: a randomised controlled trial of a combined physical and psychological intervention for older adults with neurogenic claudication, a protocol. BMJ Open. 2018;8(10):e022205.
22. Griffiths FE, Lindenmeyer A, Borkan J, Donner Banzhoff N, Lamb S, Parchman M, et al. Case typologies, chronic illness and primary health care. J Eval Clin Pract. 2014;20(4):513-21.
23. Lewis J. Analysing qualitative longitudinal research in evaluations. Social Policy and Society. 2007;Oct: 6(4):545-56.
24. Malterud K, Siersma VD, Guassora AD. Sample Size in Qualitative Interview Studies: Guided by Information Power. Qual Health Res. 2016;26(13):1753-60.
25. Gale NK HG, Cameron E, Rashid S, Redwood S. . Using the framework method for the analysis of qualitative data in multi-disciplinary health research. BMC medical research methodology. 2013;Dec: 13(1):1-8.
26. Saldaña. Longitudinal qualitative research: Analyzing change through time: Rowman Altamira; 2003.
27. Grossoehme D, Lipstein E. Analyzing longitudinal qualitative data: the application of trajectory and recurrent cross-sectional approaches. BMC Res Notes. 2016;9:136.
28. Fadyl JK CA, Theadom A, McPherson KM, TBI Experiences Research Group, Wilkinson-Meyers L, Levack W, Starkey N, Kayes N, Feigin V, Harwood M. Optimising qualitative longitudinal analysis: insights from a study of traumatic brain injury recovery and adaptation. Nursing inquiry. 2017;Apr: 24(2).
29. Guest G, Bunce A, Johnson L. How Many Interviews Are Enough?: An Experiment with Data Saturation and Variability Field Methods. 2006;18(59).
30. Sheard L, Marsh C. How to analyse longitudinal data from multiple sources in qualitative health research: the pen portrait analytic technique. BMC medical research methodology. 2019;19(1):1-10.
31. O'Brien BC, Harris IB, Beckman TJ, Reed DA, Cook DA. Standards for reporting qualitative research: a synthesis of recommendations. Acad Med. 2014;89(9):1245-51.
32. Conway J, Tomkins CC, Haig AJ. Walking assessment in people with lumbar spinal stenosis: capacity, performance, and self-report measures. The spine journal : official journal of the North American Spine Society. 2011;11(9):816-23.
33. Smuck M, Muaremi A, Zheng P, Norden J, Sinha A, Hu R, et al. Objective measurement of function following lumbar spinal stenosis decompression reveals improved functional capacity with stagnant real-life physical activity. The spine journal : official journal of the North American Spine Society. 2018;18(1):15-21.

For peer review only - http://bmjopen.bmj.com/site/about/guidelines.xhtml

34. Bussières A, Cancelliere C, Ammendolia C, Comer CM, Al Zoubi F, Châtillon C-E, et al. Non-Surgical Interventions for Lumbar Spinal Stenosis Leading To Neurogenic Claudication: A Clinical Practice Guideline. The journal of pain. 2021.
35. Hawkins RJ, Jowett A, Godfrey M, Mellish K, Young J, Farrin A, et al. Poststroke Trajectories: The Process of Recovery Over the Longer Term Following Stroke. Glob Qual Nurs Res. 2017;4:2333393617730209.
36. Audulv Å, Asplund K, Norbergh K-G. The Integration of Chronic Illness Self-Management. Qual Health Res. 2012;22(3):332-45.
37. Meisingset I VO, Vøllestad NK, Robinson HS, Woodhouse A, Engebretsen KB, Glette M, Øverås CK, Nordstoga AL, Evensen KA, Skarpsno ES. . Novel approach towards musculoskeletal phenotypes. European Journal of Pain. 2020;May: 24(5):921-32.
38. Barons MJ GF, Parsons N, Alba A, Thorogood M, Medley GF, Lamb SE. . Matching patients to an intervention for back pain: classifying patients using a latent class approach. Journal of evaluation in clinical practice. 2014;Aug: 20(4):544-50.
39. Hill JC, Dunn KM, Lewis M, Mullis R, Main CJ, Foster NE, et al. A primary care back pain screening tool: identifying patient subgroups for initial treatment. Arthritis Rheum. 2008;59(5):632-41.
40. Hill JC, Whitehurst DG, Lewis M, Bryan S, Dunn KM, Foster NE, et al. Comparison of stratified primary care management for low back pain with current best practice (STarT Back): a randomised controlled trial. Lancet. 2011;378(9802):1560-71.
41. Matsudaira K, Hara N, Oka H, Kunogi J, Yamazaki T, Takeshita K, et al. Predictive Factors for Subjective Improvement in Lumbar Spinal Stenosis Patients with Nonsurgical Treatment: A 3-Year Prospective Cohort Study. PLoS One. 2016;11(2):e0148584.
42. Gorovitz S, MacIntyre A. Toward a theory of medical fallibility. Hastings Cent Rep. 1975;5(6):13-23.
43. Fox R. Medical Uncertainty Revisited. In: Bendelow G, Carpenter M, Vautier C, et al., eds. Gender, Health and Healing: The public / private divide London: Routledge2002.
44. Katzan IL TN, George SZ, Passek S, Frost F, Stilphen M. . The use of STarT back screening tool to predict functional disability outcomes in patients receiving physical therapy for low back pain. The Spine Journal. 2019;Apr 1(4):645-54.
45. Neale B. What is qualitative longitudinal research? : Bloomsbury Publishing; 2018.
46. Irvine A, Drew P, Sainsbury R. 'Am I not answering your questions properly?' Clarification, adequacy and responsiveness in semi-structured telephone and face-to-face interviews. Qualitative Research. 2013;13(1):87-106.
47. Algilani S, Ostlund-Lagerstrom L, Schoultz I, Brummer RJ, Kihlgren A. Increasing the qualitative understanding of optimal functionality in older adults: a focus group based study. BMC Geriatr. 2016;16:70.

Consent for publication
Not applicable

Competing interests
None

For peer review only - http://bmjopen.bmj.com/site/about/guidelines.xhtml

Funding
This research is funded by the NIHR Programme Grants for Applied Research (reference: PTC-RP-PG-0213-20002). Preparatory work for the programme of research was supported by the Collaboration for Leadership in Applied Health Research and Care Oxford at Oxford Health NHS Foundation Trust. SEL and EW received funding from the Collaboration for Leadership in Applied Health Research and Care Oxford at Oxford Health NHS Foundation Trust and are supported by the NIHR Biomedical Research Centre, Oxford.

Patient and public involvement (PPI)
A group of patient representatives were involved in developing the application for the BOOST study and one member was co-applicant. PPI representatives reviewed patient materials such as consent forms, participant information sheet and the interview schedules. We carried out some practice interviews with PPIs to test and refine the interview schedules prior to data collection. The interview findings were presented to the PPI representatives in a study meeting and were reviewed by the lead PPI representative.

Contributorship statement
Sarah E Lamb (SEL) and Esther Williamson (EW) are health service researchers and physiotherapists and led the BOOST programme of work including this study and the BOOST trial. Frances Griffiths (FG) is a general medical practitioner, medical sociologist and qualitative researcher and led the qualitative study team that included Lesley Ward, a qualitative health researcher, Cynthia Srikesavan (CS), a physiotherapist and a qualitative researcher, and Graham Boniface (GB), a physiotherapy researcher.

All contributed to the qualitative study design, reading data, thematic and comparative analysis, synthesis, interpretation and reporting. LW undertook all interviews. LW and FG reviewed the interview schedules to improve the clarity and flow of the questions. CS worked with FG on analysis with GB providing independent review of coding/summaries. Any discrepancies were resolved through discussion. EW and SEL reviewed each step of the analysis and provided feedback to FG, LW, CS, and GB.

For peer review only - http://bmjopen.bmj.com/site/about/guidelines.xhtml

Better Outcomes for Older People with Spinal Trouble (BOOST)

Semi-structured interview schedules

All three interview schedules will follow a semi-structured format. Interview content from previous interviews will inform the content and sequencing of questions in the following interviews.

For peer review only

First interview

We will refine the schedule for the first interview during the early phase of data collection, including the content and order of the interview questions. The content of the first interviews will be based around, but not limited to, the following set of questions.

1. Please tell me about yourself (household, work/hobbies/family).
2. Please tell me about your general health.
3. Taking a day that was a fairly normal day for you please tell me about what you do in the day, starting with when you get up.
4. Are there activities that you do regularly that you have not mentioned (prompt for hobbies, recreation, visits, holidays, social clubs, volunteering)
5. What does aging/getting older mean to you? Do you think this affects how active/involved you are?
6. Do you feel restricted in what you are able to do? If so, what are you unable to do that you would like to do? How long have you felt restricted?
   - Prompt for how much of the restriction is due to their back/leg pain.
7. How has this restriction affected different aspects of your life?
   - Prompt for physical, mental, and social aspects of their life.
8. Have you had to change the way you do things because of your back/leg pain?
   - Prompt for how they are adapting their physical environment, their behaviour, and their use of technology for mobility or communication.
9. Have you considered other ways you could change the way you do things to enable you to get round some of your restrictions?
   - Prompt for barriers to changing behaviour.
10. What have you done to try to manage the back/leg pain?
11. What exercise do you take/have you previously taken?
    - Prompt for why they take the exercise (physical/social enjoyment), does it help symptoms, are they restricted by symptoms.
    - Prompt for reasons for not exercising/barriers to exercise.

For peer review only - http://bmjopen.bmj.com/site/about/guidelines.xhtml

UNIVERSITY OF OXFORD

BOOST Better Outcomes for Older people with Spinal Trouble

12. How do you feel about your ability to undertake activities/exercise?
13. What are your hopes/concerns for the physiotherapy that you will receive during the BOOST trial?
14. Do you think the BOOST programme will help with your back and leg problems?
15. The BOOST programme encourages people to be more active or do more walking. How do you feel about being asked to increase your activity and walking?

Second interview

The second interview schedule will be further developed after analysis of approximately 10 transcripts from the first round of interviews, and will be based around, but not limited to , the following set of questions:

1. Have there been any changes in your general health since the last interview?
2. How would you describe your activities now? How does this compare to 6 months ago?
3. How would you describe the problems with your back and legs now? How do these compare to 6 months ago?
4. Thinking again about the things that you would like to do, how does this compare to 6 months ago?
    - Prompt for what has changed, and in what way they changed.
5. Do you think that the physiotherapy you received in the BOOST study made any difference to your back and leg problems?
    - Prompt for whether there are change in the way they view their back/leg pain now; has this changed their habits?
6. What was your experience of the BOOST physiotherapy programme?
7. Has anything else made a difference to your back and leg problems?
8. Have your views on aging and activity changed since taking part in the study?

Third interview

The third interview schedule will be developed after analysis of approximately 10 transcripts from the second round of interviews. The interview schedule will encompass, and build on, the questions presented to participants in the second interview.

For peer review only - http://bmjopen.bmj.com/site/about/guidelines.xhtml

Supplementary file 3: Change framework

Participant ID	Living arrangements
02	Lives with spouse
03	Lives alone; two children live closeby
04	Lives with spouse with a small back patio garden. Previously moved from two-storey house, as no longer able to manage stairs; close contact with children

For peer review only

**24**	Lives with spouse. Weekly contact with daughter
**30**	Lives alone; Weekly contact with family
**31**	Lives alone; Frequent contact with family
**07**	Lives with spouse; Close contact with multiple family members

For peer review only

**08**	Lives with spouse
**11**	Lives with spouse & son in council flat
**14**	Lives with spouse<br>Has good social and family network
**01**	Lives alone
**15**	Lives with spouse

For peer review only

**25**	Lives alone<br>adult children closeby<br>Supportive neighbours
**33**	Lives with spouse
**38**	Lives with spouse
**41**	Lives with spouse
**05**	Lives with spouse; weekly contact with adult children

For peer review only

**09**	Lives with spouse
**26**	Lives with extended family
**28**	Lives with spouse;<br>daughter & family in other half of house

For peer review only

**37**	Lives alone
**57**	Lives with spouse; Visits daughter, locally (goes twice a day)
**32**	Lives with spouse
**34**	Lives alone
**39**	Lives with spouse

For peer review only

**43**	Lives with spouse
**47**	Lives alone
**50**	Lives with spouse and son
**53**	Lives with spouse;<br>adult children locally

For peer review only

**06**	Lives alone
[redacted]	

For peer review only

For peer review only

Treatment arm<br><br>Best Practice Advice (BPA)<br>or<br>BOOST programme	Participant-reported attendance
BPA	Attended 2 BPA sessions
BPA	Attended 3 BPA sessions
BOOST	Attended 11 of 12 group sessions.

For peer review only

BOOST	Attended all 12 group sessions
BOOST	Attended 10 group sessions
BPA	Attended 3 BPA sessions
BPA	Attended 3 BPA sessions

For peer review only

BPA	Attended 1 session
BOOST	Attended 2 of 12 group sessions (Mobility issues & Parking costs)
BOOST	Attended 11 of 12 group sessions
BOOST	Attended all 12 group sessions
BPA	Discharged at 2nd session

For peer review only

BOOST	Attended 11 of 12 group sessions
BPA	Attended 3 BPA sessions
BPA	Attended 3 BPA sessions
BOOST	Attended all 12 group sessions
BOOST	Attended 10 of 12 group sessions

For peer review only

BOOST	Attended 5 of 12 sessions<br>Had an accident, spouse's heart attack,<br>Hours to reach Oxford and back, unable<br>to walk 20 minutes of walking circuit
BOOST	Attended 11 of 12 group sessions.<br>Missed one session because of holiday trip
BOOST	Attended 9 of<br>12 group sessions.<br>Missed sessions due to trip to Spain<br>Attending sessions was tiring bus journey (10 to 5),<br>but felt like day out and managed them.<br>Felt it was a real achievement.

For peer review only

BPA	No information available
BOOST	No information available
BPA	Attended one BPA session
BPA	No information available
BOOST	Attended 4 of<br>12 group sessions<br>Missed 2-3 sessions due to holidays

For peer review only

BOOST	Did 10 of 12 BOOST sessions<br>Missed 2 sessions due to illness
BPA	Attended 2 BPA sessions
BPA	Attended 2 BPA sessions
BPA	No information available

For peer review only

BOOST	Attended 9 sessions. Participant missed one due to rain and two due to holidays in Jamaica.

For peer review only

For peer review only

Desired treatment outcomes
Get better (Symptom improvement)
Resume physical and social activities as before
Be pain free and return to building trade

For peer review only

Get better sleep
Be pain-free
Be pain-free and walk better
Found it difficult to target an improvement due to multimorbid issues.<br>Would like to walk more (2-3 times per week) and get stronger.<br>Needed information about NC.

For peer review only

To sleep better
Doesn't expect 100% recovery. Reduced pain and more mobility
Being able to walk
Pain relief
Reassurance that exercises wont harm (Mental impact > Physical impact)

For peer review only

To walk better
To walk normally and able to do things without pain
To get back to squash sport<br>There wouldn't be too many things that participant would do that are impossible to do now
To improve leg strength
To become pain free

For peer review only

Less pain for the movement<br>To do hard jobs quicker<br>To get MRI done
To become independant by 50%<br>To improve general health
Doesn't expect 100% recovery.<br>To be mobile* and improve feet and leg symptoms<br>*Mobile (able to go out without support and do stairs better, go abroad)

For peer review only

Pain relief and being able to walk to shops
To get rid of pain and walk better
Pain relief
To get rid of sciatic pain so as to sleep better.
No heaviness in legs while walking<br>Improve mentally and physically

For peer review only

To confidently be able to walk and to get back on mountain bike
To be able to do long walks (up to 3 miles) that participant enjoys without the need to take breaks and sit
To get rid of pain and able to exercise more to strengthen the back muscles
To stop leg muscle wasting and to strengthen them

For peer review only

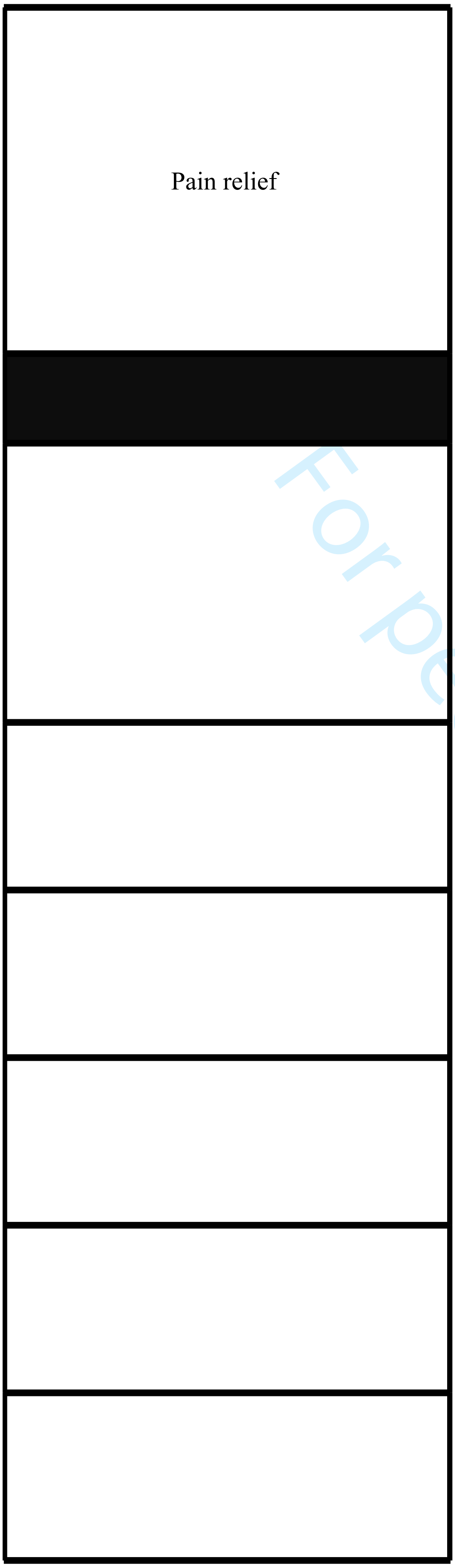

For peer review only

For peer review only

**Pain**
Slight improvement in symptoms on stopping self-prescribed exercises<br>Loses balance after sustained walking<br>Pain on standing > 5 minutes
Worse night pain, builds up from day<br>Takes pain medication
Pain has increased since previous levels and constant 24/7.<br>Takes multiple pain meds

For peer review only

Distinct symptoms at day & night.<br>Predominantly occurs at night.<br>Night time burning pain<br>Uses gel or Paracetamol or a hot drink for pain relief
Some improvement in pain in<br>previous 2 weeks of T1 (attributes to pain medication & daily flexion exercises)<br>Pain generally present on waking, eases after ~1 hour
Reduced pain with Gabapentin, 900mg/day<br>Balance issues
Main symptom is pain and cramps. Morning symptoms (pain and cramps) has slightly improved over the past few months.

For peer review only

Pain improved from 8 months ago.<br>Takes Co-codamol
Severe pain in morning, eases gradually after<br>medication- normal level around<br>late morning.<br>Takes Gabapentin, Tramadol, paracetamol & TENS
Increased pain in hips, buttocks and low back<br>Not keen on meds, takes 1/2 dose of prescribed codeine<br>due to concerns about addiction & sleepiness
Pain is main concern<br>Pain from right hip to bottom of foot<br>Coping with pain
Constant pain<br>Pain on standing (wiithin 1-2 mts)<br>Pain leads to numbness/pins<br>needles in foot, with walking<br>Progressive leg weakness

For peer review only

Constant, burning pain in right thigh<br>Stiffness on walking<br>Pain killers werent helping much, so takes Paracetamol<br>Wants to cut down Paracetamol
Not much pain, but numbness in legs that makes walking difficult<br>Has good and bad days
Ocassional pain in buttocks while walking, that is manageable<br>Pain in feet and legs that makes walking difficult<br>Numbness in feet makes participant feel losing control and balance while walking
No problem in the back<br>Pain, weakness & numbness from buttocks to feet<br>Buttock 'ache'<br>Numbness in legs 'always there'<br>Leg weakness & balance problems
Low back pain radiating to buttock and right leg<br>Pain is always there in varying degrees<br>No issues with balance or strength<br>Takes Paracetamol, only when very painful

For peer review only

Pain in right leg, all the time<br>Sore lower back within 300 yards of walking<br>Takes pain killers, if pain gets worse
No pain. Main symptoms are stiffness and numbness in back, feet and ankles<br>Numbness and instability always there<br>Feelings of unsteadiness due to leg weakness<br>Heaviness and numbness in legs and feet after walking a certain distance<br>Takes Paracetamol for pain relief<br>Finds acupuncture helpful in pain relief, but doesnt go often due to treatment costs
Constant back pain, that is manageable.<br>Pain and feelings of weakness in legs that worsened previous six months<br>Takes Paracetamol to manage pain<br>Pain & balance issues due to leg weakness while walking

For peer review only

Impact of NC is very minimal<br>Has no back pain.<br>Discomfort and heaviness in legs while walking<br>uphill, 300 yards or during ocassional calf tightness in the mornings
Horrible Leg pain > back pain, making participant limp<br>Within 5-10 minutes, back pain radiates down and making to limp<br>No issues with balance<br>Takes Paracetamol only if needed
Worse pain than before. Horrendous night pain in legs.<br>Due to mulitple MSK issues, it was difficult to establish NC pain alone.
Two pain presentations: Back & leg and Sciatic nerve pain<br>Sciatic nerve pain 24/7; Constant pain<br>Variable pain in mid-back<br>Tingling down the legs<br>Pain frequency more than before<br>Ocassional balance problems
Feelings of heaviness in both legs & being cumbersome<br>Worse pain while pottering aroud house<br>Issues with balance while walking<br>Takes painkillers only if needed<br>Need to sit down for 10-15 minutes or have a pain killer<br>as legs become cumbersome

For peer review only

Symptoms progressively worsened over past year<br>Pain below belt line<br>Stiffness during kneeling to standing<br>Pain, aching, or pulling in top of buttocks, thighs and calf muscle<br>Numbness and pain in left thigh, back of calf muscles and restricted feeling around left ankle<br>Takes Paracetamol daily, but not keen o taking medication.
Intolerable & intermittent pain<br>Muscle spasm in lower back<br>Takes 2 Paracetamol morning & night everyday<br>Takes 500mg Ibubrufen for back, ocassionally
Lot of pain down the legs<br>Severe pain on walking even shorter distance (10/10)
Pain in legs, hips and back<br>Muscle wasting in both legs<br>Tingling in both feet<br>Takes gabapentin x 3 times, but not helpful<br>Avoids pain meds during ocassions that are manageable

For peer review only

Constant pain
Pain around the buttocks and back of both thighs.
Takes painkiller (Paracetamol) in severe pain or before tasks that aggravates pain

For peer review only

For peer review only

**6 months**
Ocassional mild pains (no more severe pain)<br>Pain reduced to acceptable level, but did not fully resolve<br>Attributes to doing exercises gently than intensive before; release of<br>trapped nerve<br>Balance slightly improved, still issue<br>Pain on standing > 5 minutes didnt change
Pain worsened over 5 months<br>Constant pain<br>Increased pain (particularly at night)<br>Takes more medication to sleep
No reduction in pain<br>Constant pain and flare ups if he overdoes things<br>Limits amount of pain meds to avoid cognitive side effects<br>(Zombie)

For peer review only

Reduced day time pain;<br>Night pain unchanged.
Reduction in pain, in terms of area and intensity
Reduction in pain<br>Still on Gabapentin
Decrease in pain and morning cramps.<br>Intermittent symptoms occur.

For peer review only

Pain is halved.<br>Stopped taking pain meds
Same pattern as T1.<br>Pain severity eases over the day, as the pain medications take effect and enable mobilising<br>Takes same medications
Knee pain is main concern.<br>Knee pain triggered by BOOST sit to stand exercise<br>Ceased codeine (ineffective), and started morphine and amitriptyline to help improve mood
Back and leg pain is bit better, not bad as before & less frequent<br>Coping with pain, thinks need more improvement
Pain almost same,<br>participant manages now<br>Pain on standing-still the same<br>Not 'afraid anymore'

For peer review only

Feels better<br>Ocassional episodes of stiffness
Pain and heaviness in thighs makes<br>participant difficult to walk
Back condition is good<br>Balance is good<br>No further information
Pain worsened considerably<br>Constant pain, intensity varies by day to day activity<br>Lot more pain in sides and back of upper leg and buttocks<br>Also experiences low back ache (rather than pain)<br>Takes Paracetamol for pain
No pain in legs<br>Still gets some back pain, but keeps under control by exercises<br>Pain has completely gone now.

For peer review only

Back pain that needs 'more' pain killers<br>Legs 'ache a lot' and slows participant down<br>Balance is fine
Feels much better with leg a& back condition<br>Numbness and pain in legs is much better now<br>There are on and off days, but not bad as it used to be
Good and bad days<br>Lot of pain in legs & feet<br>No strength in legs<br>Lot of very sharp pain at night (unsure if it arthritis or NC)<br>Takes Paracetamol for pain

For peer review only

Impact of leg pain on walking and<br>general lifestyle is very minimal.<br>Leg discomfort (appears after 200 yards walking uphill)<br>has been better than T1.
Feeling 'marvellous'<br>Leg pain completely gone<br>(Disappeared)<br>Niggly back pain, but very minimal<br>& not constant anymore<br>Not taken Paracetamol few weeks
Not too bad.<br>Participant's main concern is the knee problem
Back & leg pain remains same<br>Sciatic pain remains same; Constant pain
Main problem: Pain comes instantly on standing up<br>Problem is more pain while standing<br>Heaviness in legs is not as bad as before while walking<br>Balance is good

For peer review only

Still has aches, cant say<br>pain has improved<br>Pain below belt line, while walking<br>Still takes pain killers (though not keen)
Much less sore and less stiff<br>Continues with Paracetamol<br>Rarely takes Ibubrufen
Symptoms are not too bad<br>Participant's pain medication changed a month before
No pain in back anymore<br>Muscle wasting in exponential way<br>Muscle wasting in right leg makes participant stumble often

For peer review only

Sciatica symptoms 'very painful' in last four months
Ongoing back pain all over but no new pain compared to T1
Dead feeling in part of both legs.
Still takes Paractemol for pain
Uses TENS, Voltarol gel or Tiger balm

For peer review only

For peer review only

12 months
Ocassional, but no more severe pains<br>No change in pain (Pain reduced but did notfully resolve)<br>Not sure if due to stopping 1-2 exercises or adding other exercises to regime
Decompression laminectomy, since T2<br>Constant pain<br>Pain intensified<br>(following decompression surgery)<br>Remains on same pain medication, more dose & frequency
No reduction in pain (maintained)<br>Still in lot of pain<br>Constant pain with intermittent flare ups

For peer review only

No change (further reduction) in day pain;<br>Night pain unchanged.
Further substantial reduction in pain<br>(75% improvement)<br>Pain generally present on waking, eases as participant becomes mobile
No change in pain (maintained)<br>Reduced morning stiffness<br>Still has balance issues that contributed to recent fall<br>Still on Gabapentin<br>Attributes pain reduction to Gabapentin
Stenosis pain have got lot better<br>Intermittent symptoms occur.

For peer review only

Emergency abdominal surgery six months ago<br>Symptoms have mostly resolved, except ocassional pains in nights<br>No longer uses pain medication<br>Back condition is more manageable now
Same pattern as T1.<br>Still on pain medication
Multi site pain<br>(back, neck and knee)
Feeling much better<br>Not bad as it used to be<br>Coping up with pain
Note: Spinal fusion surgery (just after Interview 2) for NC<br>No pain at all in the back (had succesful<br>surgery for NC)<br>Had Cauda equina syndrome & became double incontinent<br>Participant describes 'rest & recuperation' time

For peer review only

Before T3, had lumbar decompression L4, 5<br>Feeling great<br>Stopped taking painkillers<br>Still has stiffness and burning in legs
Still has numbness and pain
No pain, but has uncomfortable<br>tingling and numbness<br>When walking slower or standing for longer, feels falling over<br>and sits down
Back and leg condition escalated<br>Big jump forward in pain<br>On heavy dose of painkillers<br>Increased use of pain killers makes participant 'woolly'
Relieved of back pain<br>(disappeared after few weeks of BOOST programme)<br>Pleased that pain has completely gone now

For peer review only

Back surgery for CES, 7 weeks before T3 interview<br>Much better with pain<br>No pain in legs (previously severe), still has back ache.<br>Still takes pain killers for back ache
Feeling good<br>Back and leg condition is on and off<br>'Too much pain' while cooking is ocassional now
Condition same as in T1<br>Good and bad days<br>Thinks that balance and strength<br>has not noticeably improved<br>Horrendous pain in leg and foot<br>Awful pain in left side foot<br>Still takes Paracetamol for pain

For peer review only

Impact of leg pain on walking and<br>general lifestyle is very minimal<br>Able to walk uphill with less exhaustion
No more Leg pain (main problem)<br>Ocassional back pain,<br>but lot better<br>Limping while walking has reduced
Not that bad & some days are worse.<br>Has been experiencing pain everywhere.<br>Main concern is the hip and knee pain.
Back & leg pain could be better.<br>Sciatic pain is always there; Constant pain
Knee arthritis is getting worse; Suggested for bilateral replacements<br>Main concern: Aches & heaviness in legs<br>Legs affecting more than knees<br>Feels worse when pottering around house & need to sit down 5-10 mts<br>every half hour<br>Feels better when walking outside than pottering inside house.

For peer review only

No pain in thighs<br>Still has heaviness in legs<br>Major problem is pain above the hips<br>Takes painkillers for hip pain, only if needed.
Back pain has improved<br>No information on pain killers
Pain is much better and under control.<br>Manages and ignores pain well.<br>On new pain med for 6 months.
Pain in mid back subsided a bit<br>Consistent back pain is now intermittent<br>Right leg muscle wasting progresses<br>Pain in knees and hips

For peer review only

Back and leg condition is the same. There are good and bad days.<br>still in some difficulty with the back.<br>Pain is not too bad.
[redacted]

For peer review only

For peer review only

**Pain Trajectory**
Initial reduction in pain that was maintained. Participant had reduced pain upon trial entry.
Progressive worsening of pain
Baseline Pain remained throughout

For peer review only

Initial reduction in day time pain was maintained.<br>Baseline night time Pain remained throughout
Progressive improvement<br>Participant had reduced pain upon trial entry.
Baseline level of reduced pain remained throughout.<br>Participant had reduced pain upon trial entry.
Progressive improvement.<br>Participant had reduced pain upon trial entry.

For peer review only

Progressive improvement.<br>Participant had reduced pain upon trial entry.
Baseline Pain remained throughout
Increased baseline pain.<br>Pain from other MSK conditions dominates at T2, T3.
Progressive improvement
Baseline pain was maintained at T2, followed by improvement

For peer review only

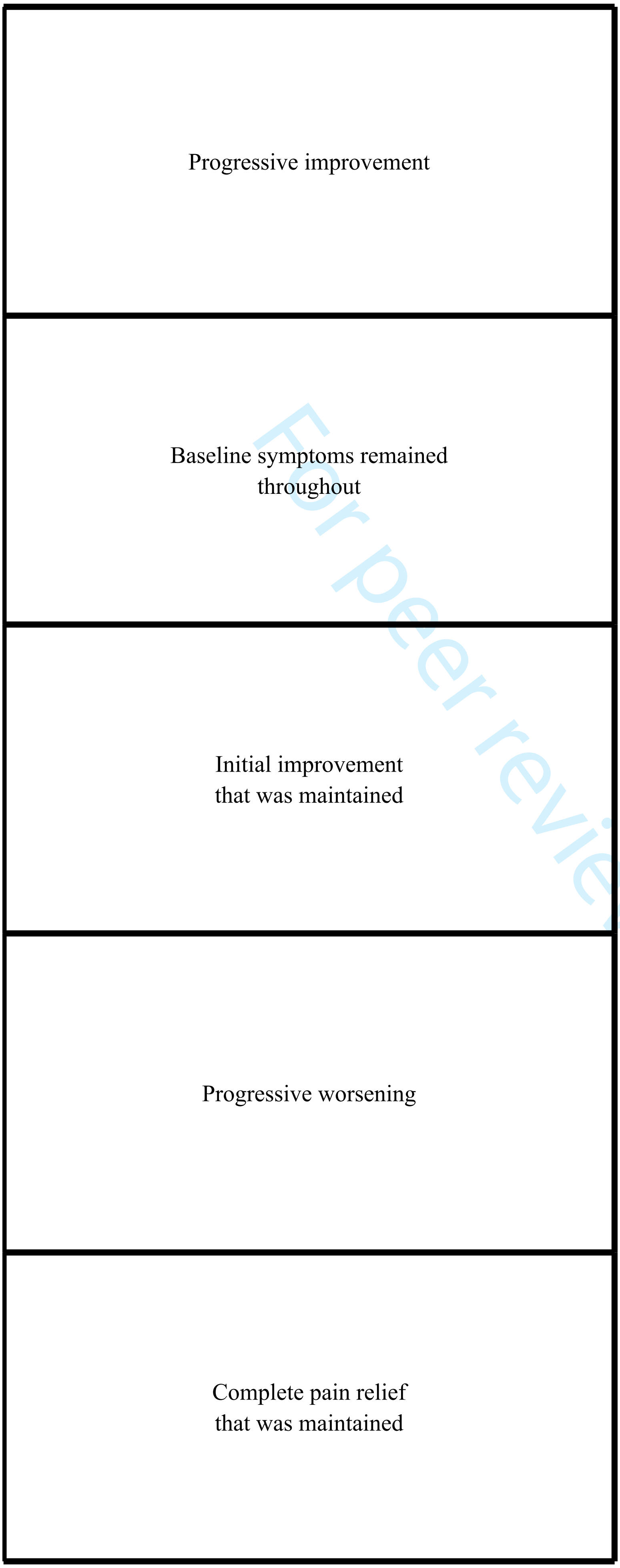

For peer review only

Baseline pain was maintained at T2, followed by improvement
Initial reduction in symptoms that was maintained
Baseline Pain and other symptoms remained throughout

For peer review only

Progressive improvement
Progressive improvement
Baseline Pain remained throughout
Baseline Pain remained throughout
Initial reduction in symptoms and back to baseline status

For peer review only

Baseline Pain and other symptoms remained throughout
Progressive improvement
Progressive improvement
Initial worsening of symptoms that was maintained

For peer review only

Baseline pain remained throughout

For peer review only

For peer review only

**Baseline<br>Mobility & ADL**
Mostly housebound; mobilises within house as much possible.<br>Manages walking upto 20 minutes maximum<br>If weather is good, manages 30 minutes, split into two or three 10 minute walks
Reduced walking distance &<br>duration due to pain<br>Sits more when out
Decreased walking speed<br>Restricted mobility and balance issues<br>Mostly home based, few outside activities due to fear of falling<br>Relies on walking aid (crutch) in & out and family member during outings<br>Unable to do housework<br>Role reversal in house hold activities (spouse washing car, cutting grass)

For peer review only

Restricted ability to stand or walk any length of time; crawls on stairs at home;<br>Difficulty accessing car, trains.<br>Reliant on spouse or public transport to go out<br>Winter makes participant less active with a feeling of tiredness all the time (SAD)
Walking has slightly improved in previous two weeks of T1.<br>Walking only short distances, even within the house, triggers leg pain<br>Not 100% happy with mobility<br>Stooped posture while walking<br>Difficulty to do grocery shopping<br>Mobility limited first thing in morning & returns to level that participant considers 'normal' over the day
Sedentary & Home based<br>Walking, Sitting, Standing are affected by pain<br>Active as much possible within house<br>Difficulty with stairs (due to compouding MSK issue)
Negotiating stairs, Mobilising out of bed takes around an hour. Pain interrupts walking within 10 minutes.

For peer review only

Activities limited by pain<br>Relates reduced mobility negatively impacting mental health
Mostly sedentary lifestyle, mainly limited to within ground floor at home and garden.<br>Mobility parallel to pain presentation- worse when he gets up and gradually increases<br>Difficult to mobilise even shorter distances outside, say 100 yards<br>As pain reduces, does functional tasks in garden<br>Difficulty negotiating stairs in home<br>Main form of mobility is driving & participant has no issues
Walking is affected very much<br>Could walk reduced distances only (double decker bus length)<br>Difficulty negotiating stairs<br>Dragging gait due to NC<br>Unable to stand for any length of time<br>Driving unaffected
Difficulty getting out of bed<br>Ocassionally uses stair lift<br>Negotiating stairs- uses hands/knees<br>& stair lift ocassionally<br>Goes out almost all days and be active<br>Takes bus for outings- walks upto 10 minutes<br>Difficulty walking uphill
Tendency to fall if tired<br>At risk of falls due to leg weakness<br>Unable to walk for some distance; has to stop frequently<br>Difficulty walking due to pain<br>Lost confidence in walking<br>Difficulty walking even shorter distances and feels exhausted<br>Driving is unaffected

For peer review only

Has good and bad days in terms of mobility<br>Tries to walk inside without aids<br>Uses Zimmer frame to go to toilet<br>Has no confidence to walk outside without atleast one stick<br>Uses walking stick outside<br>Uses taxi<br>Able to walk short distances, then gets pain<br>Walks as if a baby learning to walk<br>Stopped driving and taking the bus<br>Tries to use stairs to bedroom
Mobility depends on day<br>Able to walk to nearby church<br>Difficulty standing long at the sink<br>Difficulty with cooking standing long
Could walk half or more mile before pain comes on<br>Numbness in feet leads to losing control and balance while walking
No major problems with daily life<br>Pain not stopping doing any activity<br>No issues with mobility inside home<br>No difficulty going multiple staircases at home<br>Due to weakness & numbness, has balance problems<br>Holds bannisters going up/down to avoid falling<br>No issues with driving<br>spouse drops/picks for academic work<br>Slow walking due to leg weakness (main issue)<br>Uses stick very ocassionally<br>Balance problems while walking
Participant 90% pleased with the mobility level<br>Walks everyday to alottment area (20 mts)<br>Going upstairs can cause pain, but participant manages it<br>No issues with driving

For peer review only

Back problem restricts participant's walking<br>Uses handrails on stairs<br>Gets pain within walking 300 yards<br>Stopped walking too far<br>Reduced walking up hills due to shortness of breath<br>Driving is not too bad, but uncomfortable
Mixed messages about walking:<br>No issues walking inside home<br>Difficulty going upstairs, sometimes<br>Difficulty coming down stairs in morning<br>Uses handrails to come down<br>Tries to walk whenever possible, at own pace<br>Pins and needles comes at 5-10 minutes of walking<br>Goes out on alternate days<br>Tries to walk whenever possible<br>Unable to walk 20 minutes distance to son's place
Feels weaker and physical decline<br>Pain and balance problems due to weakness in legs<br>Comfortable indoors but still moves (no aid) with pain<br>Prefers to stay indoors, doesnt enjoy outside due to pain and walking problems<br>Not going out as used to (reduced shopping 2-3 times/week and city trips 2-3 times/year)<br>be due to pain<br>Afraid going out due to fear of falling, so has a support (stick)<br>Reduced ability to walk or go shopping<br>Cannot walk far without aid<br>Difficulty going upstairs; Comes down one step at a time<br>Increasingly difficult getting in out of car<br>Driving unaffected but has reduced<br>Stopped the regular morning walks for nearly a year

For peer review only

Impact of NC is very minimal<br>Very active inside home-cooking, cleaning, constant walking<br>No issues walking on flat surfaces or downhill<br>Discomfort and heaviness while walking uphill<br>Difficulty (exhausted) walking to local shops, uses car<br>No issues with standing
Leg pain makes participant limp within 5-10 minutes walking<br>Leg pain within 10-15 minutes standing<br>No issues with balance<br>No issues as long as no long distance walking<br>Dog walking up to the road every day
Stopped walking/biking outside since 2 years<br>Difficulty with house work (cleaning)<br>Difficulty getting in out of car<br>Manages trips to laundry room downstairs<br>(6-8 times with 2 loads)<br>Goes out everyday to shopping area using mobility scooter<br>(up to 5 min walking distance)<br>Could walk a small distance inside store<br>Feels exhausted all the time
Participant afraid to walk on own<br>Could walk only half way up the road, due to back pain & 24/7 sciatic pain<br>During less back pain & good weather, could walk up to 3/4 mile<br>Unable to walk longer or quicker<br>Uses taxi & online shopping
Leg symptoms made participant stop doing things<br>Leg symptoms affect day to day activities like shopping<br>Difficulty going up steep stairs at home<br>Not sure how far participant could walk while going for shopping<br>Needs to sit down 10-15 mts or use painkiller as legs become heavy & cumbersome<br>Has trouble with long walks<br>Issues with balance and tries to be cautious while walking

For peer review only

Busy retirement life
Feels restricted in most of things got to do, compared to 5 years
Difficulty doing stairs due to pain
Unable to walk any distance- too painful
Walks with stick-less painful

Participant goes to local shops and small markets
Unable to do long walks due to back problem and arthritis joints
Couldnt go far and unable to finish a self-created walking circuit around the village
Unable to stand for a while & talk to people; so need to find a seat
Rain doesnt stop participant's activity

Uses bannisters on walls, bar on bed, hand rails to mobilise around home
Has a walking stick to balance while walking outside

Back pain stopped participant from doing everything
Destroyed general lifestyle
Unable to continue any activity-Becomes lethargic mainly due to back problem
Amount of walking is quite restricted
Unable to push trolley around the store
Crawls or takes one step a time on steep staircase
Difficulty with carrying a tray or coffee upstairs
Holds on something to get off the bed
No issues as far participant doesnt go far and takes rest in between
Fairly confident walking with a stick for a distance
Multiple falls due to right leg weakness

For peer review only

Unable to walk as before (2-3 miles)
Unable to walk faster
Bending down makes participant have a short of breath
Stooped posture while walking due to neck problem

For peer review only

For peer review only

**6 months**
Walking duration improved (30 minutes);<br>Balance improved
Pain inhibits mobility<br>Difficulty to do any ADL
Getting out more than before, inspite of pain<br>Walking upto 30 minutes, expanded walking environment<br>Improved transfers (in/out car), but not so signficantly<br>Still some balance issues with walking<br>Still unable to do housework and gardening

For peer review only

Increased walking (up to 2 hours), balance, and ability to negotiate stairs in upright position;<br>Confident using steps and slopes without fear of falling<br>Improved balance, example: Can carry objects while walking
Increased walking on a daily basis (from walking within short distances even within home)<br>Pleased with physical and social activity levels
Walking better (less shuffling)<br>No issues with mobility during a holiday or air travel<br>Difficulty with stairs (due to compouding MSK issue)
Morning mobility improved. Able to easily mobilise in out of bed. Difficulty negotiating stairs due to hip/knee issues. Improved walking (half a mile easily) due to less pain.<br>Has to concentrate while walking due to balance issues.<br>Difficulty being active outdoors in winter with risk of unsteadiness

For peer review only

Feels physically better due to reduced pain
Walking is bit better- duration increased
Increased physical activity- engaging in stretching exercise for flexibility
Increased energy levels due to reduced pain, but not to the fullest

Mobility is the same as T1.
Recent two falls impacted confidence in walking
Balance and gait issues with walking
Participant limits the walking distance by parking closer
Restricted walking duration due to fear of falls
Has to be careful getting from bed due to balance issues pose falls risk

Keeps being mobile
Feels pain medication help in walking
Walking duration has increased (upto 30 minutes)
Walking pace has increased by using walking poles
Pleased with mobility levels
Does some form of walking and exercising most days
Driving is unaffected

Pleased with walking
Walking almost all days
Walking speed increased
Achieved a goal- walking down to second bus stop
Could walk approx 20 minutes & return
Negotiating stairs -same
Continues to use stair lift ocassionally
Didnt try walking uphill

Feels better with improved activity levels
Walks up to main road with stick
Pain still stopping the participant to be active
Feels in better place physically
Attributes physical improvements 100% to BOOST

For peer review only

Walking is much better<br>Doesn’t need a trolley or stick to walk around house<br>'Carries a stick when outside, uses it for uneven grounds or steps<br>Can walk almost normal<br>100% satisifed with mobility<br>Achieved 95% mobility but never will be able to reach 100%<br>Can go and get out of bus with one stick<br>Uses chairlift at nights when tired
Mobility depends on day<br>Back condition slightly better<br>Can walk more freely and walk longer (managed to walk quarter mile)<br>Able to stand bit longer at sink than before<br>Can do cooking and sits down to prepare meal
Back in real world'<br>Satisfied with more walking than before<br>Walking tremendously improved since T1<br>Unable to stand for long time and needs to sit down<br>Walks with rollator 4-5 miles without sitting down, 1.5 to 2 hours<br>Goes for walk everyday 45 minutes to 2.5 hours at a reasonable pace for upto 5 miles<br>Balance is good<br>Can get on the bus
Difficult going upstairs due to pain<br>Walking is slow (strolls)<br>Doesnt take a stick everyday
Mobility and activity levels are very good<br>Feeling better in general well being<br>Feels an improvement overall<br>No pain while walking<br>Happy with mobility levels

For peer review only

No much difference in condition compared with T1<br>Not doing much in the farm due to back pain<br>But still goes every day to do something<br>Struggling to walk without more pain killers<br>Drives slowly & takes longer time 2.5 hours
Posture has improved<br>Balance is bit better now<br>Walks more than before<br>Can walk 100 yard/ quarter mile now<br>Feels better with leg and back condition<br>Somedays can walk without stick
Comfortable mobilising inside home<br>No strength in legs; Walking is difficult<br>Current ability to walk is 'average'<br>Happy being indoors<br>Manages stairs (14 steps) at home<br>Afraid of falling when going outside, walks with a stick

For peer review only

Impact of leg pain on walking on<br>general lifestyle is very minimal<br>Could walk 200 yards uphill to local shops. Feelings of heaviness improved<br>Also likes to drive to shops<br>Feels walking has improved<br>Says cannot walk long distances due to aging
Feeling 'marvellous' with present condition<br>'Lot lot better' with walking<br>NC has no impact on day to day activities<br>Able to walk without pain starting 5 minutes<br>Able to continue walking without any<br>restrictions or looking for<br>places to sit down (as before)
Participant still manages trips to laundry room downstairs<br>(6-8 times with 2 loads)<br>Fatigue limits going out (3 times a week)<br>Still uses mobility scooter to move outside<br>Still has difficulty getting in out of car<br>Still uses trolley to walk in super markets<br>Managed to walk 1000 yards with 3-wheeled walker<br>Mobility limited by the knee problem<br>Because of other MSK pain conditions cropping up, it is difficult to<br>differentiate what is caused by the back and what isn't.
Still going out & doing things as before<br>No changes in activity levels<br>'Satisfied' with activity<br>Walking is not that bad<br>Sits down when pain on walking
Main problem: Pain comes instantly on standing up<br>Difficulty going up steep stairs at home; manages normal stairs<br>Mobile most of morning at home<br>Participant is walking more- walking better & longer<br>Doesn't feel the heaviness in legs immediately while walking<br>Describes walking 20 minutes twice a week easily<br>Has no concerns with walking

For peer review only

More active now<br>'Vast difference' in condition<br>Not sure if achieved physical improvements<br>Not 100% satisfied with walking compared to how he was before<br>More good days than bad<br>Covered 8000 steps one day and was far happier & amazed<br>Goes for long walks with dog up to one mile without any problem<br>Walking unaided
Participant's mobility is increased<br>Felt physically better- stamina, activity and breathing wise<br>Could walk up to 30-40 minutes, breaks, and return as well<br>Parks car further away from destination to walk more<br>Walks with more confidence<br>Can stand for longer without need to lean on something<br>Didnt need the hiking stick when walking to coffee shop (Major change since T1)<br>Confident to increase the walking even more
Exercises are helping to walk up down stairs at home<br>Able to walk for 5-6 minutes before getting tired<br>Able to walk further along street with walker<br>Uses wheeled walker with trolley seat<br>Able to go shopping/to town with spouse
Despite pain relief, activities remain restricted.<br>Main concern: Right leg weakness<br>Leg problem 'crucified' activities of daily living<br>Unable to stand very long (washing up at sink)<br>Uses stair lift more often<br>Finds it difficult to move around

For peer review only

Walking is same as before.<br>Walks everyday, if it is not raining<br>Walks, sometimes stops and slows down<br>For faraway places, either drives or takes a bus<br>Tries to walk faster, but right foot seems to be dragging.<br>Able to get on and off bus<br>Able to get up from chair 'reasonably well' inside home
[redacted]

For peer review only

For peer review only

**12 months**
Mobilises around house & garden<br>Pain still affects walking<br>No change in Walking duration (remained 15-20 minutes)<br>(some seasonal effect- e.g. able to go out more in summer, in winter no place to sit down)<br>Not overly satisfied with walking- 6/10
Decompression laminectomy, since T2:<br>No change in Mobility and all activities (maintained)<br>Sits more often when out compared to previous year
Day is sedentary & home based<br>Confident mobilising inside home than outside<br>Struggles to do activities of daily living (dressing)<br>Avoids walking outside (seasonal effect-cold weather).<br>Mobilises inside and avoids walking in snow and ice

For peer review only

Goes for walking everyday<br>Further increase in walking, balance,<br>and ability to negotiate inclines and walking<br>different places, negotiate stairs in upright position (100% improvement)<br>No impact on walking
Walks most days<br>Further increased walking -<br>2 miles at ease;<br>Very satisifed with mobility<br>Son & acquaintances noticed improvements in posture
Participant remains active within house<br>Walking better; attributes to improved pain<br>Able to walk up the road<br>Difficulty with stairs<br>(due to compounding MSK issue)<br>Would take bus, if weather is good
Morning mobility out of bed improved. Able to sit at the table in the mornings due to less pain.<br>Able to walk down stairs one at a time<br>Walking up stairs difficult at night times<br>Has to concentrate while walking due to balance issues.

For peer review only

Emergency abdominal surgery six months ago:<br>Activities restricted mainly due to recent emergency surgery and time of year (winter time)<br>Can do most activities except heavy lifting and more rigorous activities<br>Increased walking - upto one hour<br>Able to re-engage with longer walks with spouse- up to 3 to 5 miles, compared to T1<br>Able to walk for leisure, including local walks and holidays<br>Able to walk further up hills without stopping
Increasingly sedentary and home based- mobilise within ground floor only<br>Walking is about the same as in T2<br>Participant limits the walking distance by parking closer<br>Participant limited the walking duration<br>No issues with driving<br>Mobility impacted by legs and lung condition.
Typical day is 'pretty rotten'.<br>(due to multi site pain-knee, back, neck)<br>Limited in walking distance<br>Goes for walks for 10-15 minutes<br>Still able to drive
Walking is better<br>Pleased with walking<br>Goes out everyday<br>Negotiating stairs -same<br>Continues to use stair lift ocassionally<br>Walks to and fro bus stop (goal achieved in T2)<br>Continues to use bus
Note: Spinal fusion surgery just after Interview 2) for NC<br>Had Cauda equina syndrome & became double incontinent<br>Describes 'rest & recuperation' time for participant<br>Uses walking frame inside house & moves with difficulty<br>Right leg buckles & some one always escorts participant during walking<br>Attempts to stand independantly

For peer review only

Before T3, had lumbar decompression L4, 5<br>'Great improvements' in walking<br>Able to walk without stick, sometimes<br>Balance is not 'brilliant' but 'not bad'<br>Day to day activities are 80-90% better<br>Still not quick on stairs, uses stair lifts at nights when tired
Slight improvement in mobility since T1<br>'Very frustrated' as he couldn't walk much<br>Pain still limits walking<br>Walking is quite bad and cannot walk very far<br>Can walk up to 100 yards, gets numb and painful. Sits for 5-10 minutes<br>Mangaged to walk quarter mile (post office)- maximum sort of activity<br>Confidence with walking has affected to a certain degree
Satisifed and feels 100% better in mobility levels compared to T1<br>Walking more often (from 200 yards to 3-4 miles now)<br>Able to walk shorter distances without rollator<br>Able to walk distances with rollator<br>Feels unsafe over control on feet when walking slower or standing<br>Walks 1.5 hours of walking, most days
Sitting more during day<br>Continues to use stairs<br>Walking with a stick now<br>Lost 1 inch of height; walks with tight hunched posture<br>Movements are more focused on getting to a place<br>Standing has become more difficult
Participant walking a lot more<br>Walking a lot (30 minutes a day), most days<br>Back problem has no effect on lifestyle<br>Believes current lifestyle is fine and current activities are enough

For peer review only

Back surgery 7 weeks before T3 interview<br>Much better with mobility.<br>Can walk half a mile now.<br>Participant is pleased with walking<br>Can sit down and drive, pefectly again<br>Not doing much in the farm, only odd jobs<br>Shortness of breath (from recent surgery) interferes with walking
Mobility improved compared to T1<br>Walking is better<br>Can manage stairs at home (12 steps), uses handrails<br>Can walk shorter distances<br>After walking some time, feels weakness in legs and feet and stumbles<br>Walks slowly and takes time
Comfortable mobilising inside home<br>Balance is not too bad indoors because she is comfortable inside home<br>Happy being indoors<br>Manages stairs (14 steps) at home<br>Not walking much due to foot pain<br>Not 'terribly confident' with walking<br>Uses stick when going out<br>Doesnt feel 100% safe walking outside.<br>Unable to do 20-minute BOOST walking

For peer review only

Impact of leg pain on walking or general lifestyle is very minimal<br>Thinks walking better compared to T1<br>Doesnt walk much being lazy, uses car<br>Able to walk uphills to shops easier (before, he felt exhausted)<br>Doesn't walk long distances to keep life easy
Lifestyle & activities 'much better'<br>'Very satisfied' with walking<br>No more restrictions in walking<br>Now able to walk around shops with spouse<br>Limping while walking has reduced
Lifestyle no different than before back pain<br>'Non-existent lifestyle'<br>Unable to do many things due to hip<br>Still manages to do laundry<br>Mobility levels varies between days<br>Unable to sit longer on scooter trips to supermarket<br>Better mobilising with walker<br>Would walk up to car, and not too far
Continues to go out<br>Still doing the same activities<br>Could not walk quicker<br>Balance is okay
Thinks that legs are affecting more than knees<br>Going up stairs is getting worse, but tries to do them<br>Tries to be mobile within house-doing housework<br>Terrible difficulty getting up from chairs<br>Doing what is used to, but not as much used to<br>Feels worse at home just pottering & has to sit 5-10 mts every half hour<br>Could walk, but not longer distances<br>Thinks about the least distance to walk & plans the walking. Worse than T1.<br>Still drives, but not very often and has lost confidence

For peer review only

Tries to be active & doesnt sit too long<br>Walking has improved in terms of strength and balance, particularly the latter<br>Gives examples of activities: Driving almost six hours;<br>Walking up to 6 kms during two ocassions<br>Covers 1300-1400 steps on regular basis<br>Feels more active than before<br>Walking stick to lean on, during long walks with dog
No restrictions in indoor mobility or daily life activities<br>Walking has 'greatly improved'; Would achieve 7000 steps in and outside, most days.<br>Tries to increase the 1-mile circuit walking<br>Hasnt continued circuit walking because of wet weather and muddy conditions
Getting a lot better<br>Able to get up and walk without support in house<br>Mobility is lot better than it was.<br>Balance is still wobbly sometimes.<br>Lot more confident with walker because participant can sit down
General lifestyle hasnt changed much<br>Unable to say there were any physical improvements<br>Participant feels like stumbling while walking due to leg weakness, and needs stick all the time

For peer review only

Walking is same as before<br>Walks mainly for 'exercise' but participant does walk to shops if the day is good<br>Walking is about 20-30 minutes, but not a quick walking<br>No issues with driving<br>Participant feels that mobility and activities haven't worsened
[redacted]

For peer review only

For peer review only

**Mobility & ADL Trajectory**
Initial increase in mobility and back to baseline status
Initial decrease in mobility that was maintained
Initial increase in mobility and back to baseline status

For peer review only

Progressive improvement
Progressive improvement.<br>Participant already had improved mobility upon trial entry.
Initial increase in mobility that was maintained.
Initial increase in mobility that was maintained. Still has balance issues.

For peer review only

Progressive improvement
Baseline decrease in mobility that remained throughout
Initial increase in mobility and back to baseline status Impacted by other joint pain.
Initial increase in mobility that was maintained. Participant already had improved mobility upon trial entry.
Initial increase in mobility that worsened due to other condition

For peer review only

Progressive improvement
Slight increase in mobility that worsened
Progressive improvement
Progressive worsening.<br>Participant had improved mobility upon trial entry.
Progressive improvement.<br>Participant already had improved mobility upon trial entry.

For peer review only

Baseline decrease in mobility maintained at T2, followed by increase

Initial increase in mobility that was maintained

Baseline decreas in mobility remained throughout

For peer review only

Initial increase in mobility that was maintained. Participant already had improved mobility upon trial entry.
Progressive improvement
Baseline decrease in mobility remained throughout
Baseline decrease in mobility remained throughout
Initial inrease in mobility that worsened

For peer review only

Progressive improvement
Progressive improvement
Progressive improvement
Baseline decrease in mobility remained throughout

For peer review only

Baseline decrease in mobility remained throughout

For peer review only

For peer review only

Baseline<br>Psycho impact
Frustrated depending on others for simple tasks<br>Depressed for not able to contribute to and attend rotary club events<br>Negative psychological impact of getting assistance from spouse at airports
No information
Fear avoidance of activity outside-Fear of falling<br>Depressed, frustrated depending on others and<br>feels older than actual age.<br>On anti-depressants<br>Unable to have intimate relationship with spouse<br>Feels worthless & burden to son<br>Fear of falling, leading to paralysis<br>Role reversal in house hold activities (spouse washing car, cutting grass)

For peer review only

Lack of interest in dressing and housework<br>Doesnt feel lively due to sleep deprivation<br>Impatient when accessing public transport, as participant feels holding other passengers up
Lack of motivation to engage in social and house hold activities-gardening
Participant lost confidence in walking<br>Less inclined to do anything because of pain<br>Doesn’t like being dependant on others<br>Frightened that the walking is very much reduced
Worried about prognosis; Irritable; Frustrated and emphasises family support.

For peer review only

Slight lack of confidence while walking<br>due to recent fall.<br>More postive and motivated due to reduced pain.<br>Loss of activity towards depression and anxiety about future.
NC pain & mobility issues result in impaired mental health;<br>Worried 24/7; suicidal thoughts<br>Physical limitations are stressful<br>Unable to cope up with social isolation due to mobility issues<br>Anxiety due to reduced independance<br>Upset unable to do gardening as before (professional gardener)
Sometimes, feels sorry for with NC (depression)<br>Decline in general positive outlook (being a happy person) due to pain and reduced mobility<br>Apprehensive about going out regarding finding place to sit down<br>Angry being unable to do anything about her condition
Coping up with pain<br>Determined to be active<br>Confident using stick in the buses<br>Doesnt want to stop what she is doing
Participant fearful that exercise might cause harm<br>Frightened that may cause any damage to the back by pushing to limits<br>Backpain dominates the life<br>Prefers surgery for NC<br>Accepts the back problem is chronic and the need to self manage<br>Lack of confidence in walking due to pain<br>Distressed with the decreased abilities<br>Back problem affects participant's mood and becomes snappy<br>Horrible feeling to fight the pain<br>Frightened to cause any damage to the back, so paces between activities

For peer review only

Has no confidence to walk outside without stick<br>Annoyed because unable to help people at WI<br>Determined not to use wheelchair<br>Hopeful to overcome obstacles doing things like taking the bus
Accepted to live with back problem<br>Negative effect on mental health<br>Demotivating while engaging with others<br>Ankle issue is stressful and unable to cope up
Upset with symphatetic<br>smiles from others
Annoyed with aging spine<br>Becoming more consious of physical limitations<br>Participant says physical condition 'shocks'<br>Has to concentrate on walking due to balance issues<br>Depressed about the leg muscle weakness<br>Accepts that the back problem wont change
Fairly active lifestyle with walking and driving<br>Wont let the pain impact the mobility<br>Feels lucky that some people may become immobile with NC<br>Participant is more worried by NC, than BP and heart conditions<br>Participant's worry is whether the NC is going to get worse and would lead to stop doing things

For peer review only

Afraid that walking more would lead to pain
Confident walking on own with stick<br>Gets upset when people see with walking stick<br>Has fear of falling<br>Participant feels to take help to be more active<br>Participant worried about depending on others during holidays<br>Anxious about leg, back and ankle problems<br>Depressed when people relates health problems to aging<br>Determined to walk, inspite of difficulty and be active
Afraid of going out because of fear of falling and prefers having a stick<br>Doesnt feel capable any more<br>Fear of falling while walking upstairs or downhill<br>Prefers to stay indoors<br>Annoyed of inability to do things as before<br>Determined to be active in spite of pain<br>Says not given up and willing to try to improve the condition<br>Not depressed with her loss of general flexibility<br>Lost confidence in going out in car and meeting friends<br>Moans about her back condition everyday<br>Not giving up and willing to self-help<br>Cannot stop thinking if she might end up in wheelchair

For peer review only

Impact of NC is very minimal<br>Cautious walking downstairs due to balance issues, holds handrail<br>Gets depressed, ocassionally<br>Participant is worried if the condition worsens<br>Confident in the ability to self manage and adapt an easy life
Accepted to live with back problem<br>Depends on colleagues to lift heavy items<br>Pain annoys the participant making to put off activities like car washing
Fed up with difficulties in walking<br>Aware of doing things within limits<br>Describes as independant person<br>Participant pushes to do cleaning tasks at home
Fed up with constant pain<br>Determined attitude to manage pain & be mobile<br>Feels sitting around is unhealthy, mentally and physically<br>Would continue to adapt & do things<br>Mentally attached with the garden
Mentally down<br>Worried about how she is going to end up<br>Feels cumbersome due to leg symptoms<br>Prefers to be self-reliant & refuses to ask help

For peer review only

Frustrated with difficulty moving around & restricitions in activities<br>Frightened about getting to stage of sitting down and doing nothing.<br>Keen on being active, though it hurts.<br>Depressed about worsening of symptoms<br>Developed depression and anxiety<br>Anxious about causing accident while driving<br>Frustrated because of the longer time it takes to complete an activity<br>More thoughtful before doing a task<br>Doesnt want to take meds for depression
Much worried' about back problem<br>Back problem dominates mind<br>Accepted progression of NC<br>Emphasises importance of self-management<br>Cautious about snow due to fear of falling
Depends on others<br>Irritated when unable to do something wanted to do<br>Not able to do anything leads to depression<br>Strained relation with spouse<br>Puts a brave face to make things lighter.<br>Has to change many things to make life lighter.<br>Participant feels independance has gone completely.<br>Back problem has stopped the life as participant has to plan everything
Back problem impacts mentally than physically<br>Participant dissatisfied with ability to do activities of daily living (due to lethargy)<br>Scared of pain and so, cautious in doing things<br>Doesnt believe to be physically cured, instead would accept living with the condition<br>Feels horrible seeing others able to do things whilst participant cannot<br>Back problem has impacted mentally that participant is unable to go for social outings<br>Back problem has impacted mentally that participant has to distract on hobbies<br>Frustrated with lack of timely help and treatment options<br>Feels mentally active at 75 years<br>Tries to self-motivate to try and do things

For peer review only

Determined and focused on self managing pain
Believes in the consequences of overloading the body
Distracts from pain by doing things that are enjoyable
Doesnt worry about pain and lives in own terms
Describes as 'handyman'

For peer review only

For peer review only

6 months
Increased confidence that back pain wont reappear<br>Focuses more on safety while walking<br>Lacks courage to resume biking due to back not feeling strong enough for this activity<br>Fearful of symptoms returning
No information
Depressed, frustrated depending on others and feels older<br>Feels very depressing in winter<br>Accepts living with NC; would not return to previous level of function<br>Depressed because he expected improvements after BOOST

For peer review only

Increased sense of well being, confidence to use steps/slopes;<br>Increased ability makes participant feel 'useful'.<br>Depressed as unable to do housework (does not have energy due to sleep deprivation)
More active than before<br>Walking on a daily basis;Improved mood; Improved motivation to go out and walk.<br>Proactively tries to increase the amount of walking<br>Adopts a positive mind-set to overcome times when experiencing pain.
Less fearful of walking<br>(due to reduced pain)<br>Feels able to self-manage NC<br>Learning to cope; and less anxiety<br>Determined to maintain independence
Determined to maintain function and mobility.

For peer review only

Mentally improved<br>Improved confidence in walking<br>Improved physical condition led to improved mental health<br>Improved mood<br>Improved ability to cope up<br>Feels positive due to significant improvement
Recent two falls impacted<br>confidence in walking
Ceased codeine (ineffective), and started morphine and amitriptyline<br>Improvement in depression<br>Feels taking control of life<br>Came into terms with restricted mobility, which enabled to make the most of remaining ability<br>Support from friends, family help cope emotionally
Coping up with pain<br>Determined to go out/active
Reassured that exercise will not cause harm-Biggest benefit<br>Participant's fear factor is taken away<br>'Can do more' as participant is clarifed<br>Mental health towards NC is much better<br>Copes up with pain & active than before<br>Attributes mental improvements 100% to BOOST

For peer review only

On top of world' due to improvements in walking<br>Coping better and with confidence<br>Participant feels independent as now can go on bus on own<br>Realises life is worth living and back to normal now<br>Doesn't sit around and feel sorry for ownself
Participant is coping well<br>Feels alright if he doesnt think about the back problem<br>Manages to cope with circumstances<br>Participant is content but thinks can do more<br>Worried about mental capacity to<br>cope with events/life situations
Has no confidence to walk without rollator<br>Feels guilty about people smiling and opening doors<br>Satisfied with more walking
Tries to be active and not a couch potato<br>Bit dpressed about how the back problem would progress<br>Worry stops participant from booking events that participant<br>would love to attend<br>In terms of general life, feels worse due to hot weather
No information available

For peer review only

No information available
Confidence in walking 6-7<br>out of 10
Participant prefers to make life easy as possible<br>example: Declines going out for meals<br>Still aftraid of fall when outside<br>Not terribly confident with the walking (fear of falling)<br>Confident using stick otuside<br>Doesn’t want to give up<br>Tries hard to get on with life<br>Accepts the condition, not depressed or upset<br>Participant mentally hopeful with using BOOST for self-help<br>Participant feels not missing any activities

For peer review only

Compared to T1, participant now more confident in walking short distances<br>Not embarrased with mobility levels<br>Would avoid Probus tour activities that involves lot of walking, not to embarrass others
Feeling 'marvellous' with present condition<br>No more annoyed with the walking needing to sit down.
Doesnt like to give up (household chores)
Participant manages by carrying out whatever possible<br>Continues to do activities -what and when possible<br>Just gets around because participant has to<br>Needs to cope with her back pain because nothing can be done.
Describes that mental impact has got better<br>Pain while standing makes participant mentally not to get up<br>Particiapnt self-reminds to keep going with exercises as per physio's advice<br>Describes mental impact has got slightly better<br>Confidence while walking is not good due to impaired eyesight

For peer review only

Participant's confidence has increased, ex. deliberately walks across car park than parking closer<br>Participant feels improved mentally (though not sure of physical improvements)<br>Has come to terms with the back condition & takes effort to manage<br>More satisfied now because of understanding of the condition participant was 6-12 months before<br>Confidence in walking has increased<br>Seeing more good days in terms of depression
NC is now physically and mentally less problem<br>Feels NC is redeemable through exercise and self-management<br>Participant feels rehabilitation helped to achieve greater outcomes
Has got used to pain.<br>Learning to tolerate and manage pain.<br>Learnt to cope than before.<br>Lot more confident with walker because he can sit down<br>Tries to be positive<br>Does get depressed somedays, sitting and crying.<br>Some days are good and some bad-in terms of motivation.
Participant frightened about the leg problem<br>Worried about the other side leg & arm strength as well<br>Concerned about the future

For peer review only

Feels 'uncomfortable' to live with pain
Feels no one can help with the pain.

For peer review only

For peer review only

12 months
Participant feels envious of others who can walk faster<br>Avoids certain activities due to fear of pain
Decompression laminectomy, since T2:<br>No impact on mental health
Depressed, frustrated depending on others and feels older than (maintained).<br>Frustrated as not active for actual age<br>Short tempered due to lack of sleep<br>Mood affected by cold weather (SAD)<br>Feels psychological impact of pain is less in summer<br>Winter has a negative impact on physical & mental health

For peer review only

Increased confidence to try new things;<br>Negotiates stairs; inclined surfaces<br>Increased independance;<br>Determined to maintain mobility gains
Positive & determined attitude to cope up with condition<br>Increased confidence in walking after successfully walking longer during a recent holiday (Turning point in mind set overcoming NC related mobility issues).<br>Paces walking
Less fearful of walking (due to reduced pain);<br>Tends to be carful due to balance issues.
Overcautious while walking; Determined to walk

For peer review only

Emergency abdominal surgery six months ago:<br>Views mental health as driver of physical improvement<br>Improved confidence in walking over challenged terrains<br>Improved confidence in walking after being assured that previous fall was not back related<br>Felt motivated and pushed to walk further during a recent holiday<br>With recent surgery, feels positive to lead a life he wants to live<br>Feels more potential for improvement and well being
Learns to adjust to limitations<br>Finds NC & lack of physical ability distressing<br>Substantial distress & anxiety (back condition) compounded by lung condition<br>Annoyed being dependant on others<br>Fear avoidant behaviour of walking more or exercising more<br>Recent two falls impact on confidence with walking
Physical issues led to deterioration in mental health (multiple MSK issues- esp knee pain, recent fall)<br>Depressed mood<br>Upset with physical inability from compounding MSK issues<br>Fear avoidance behaviour -hesitates going out due to recent fall
Participant determined to do whatever possible to do<br>Pain would not stop the activities<br>Thinks exercise improves mood<br>Participant feels coping better after doing exercises
Note: Spinal fusion surgery (just after Interview 2) for NC<br>Had Cauda equina syndrome & became double incontinent<br>Has a ‘Positive’ outlook & in good spirits<br>Accepts that the current state is out of control & being miserable won’t change anything.

For peer review only

Before T3, had lumbar decompression L4, 5<br>Amazed with progress<br>Believes improvements are from the support from family<br>Feels life worth living<br>Accepts the condition will never be 100%<br>Realised the need to self-manage
Very frustrated' with walking<br>Confidence in walking affected to a degree<br>Motivation is not great<br>Not depressed<br>Has more insight on back problem
Compared to T1, now totally independent<br>with rollator going shopping or using bus<br>Confident in walking with rollator
Participant assumes that the condition is manageable<br>Condition is always in participant's mind, but wont let it grab<br>Surgery is now a 'trivial matter' compared to the condition<br>Participant feels no problems mentally, amidst physical limitations
Back problem wont stop participant from activities<br>Participant realises that more can be done<br>'Very confident' about getting out and walking

For peer review only

No information available
Does not depend on family to do activities<br>Particiapant tries not to let the symptoms have an impact<br>Depends on family for long distance travels
Not terribly confident with the walking (due to foot pain)<br>Not feeling 100% safe walking outside<br>Annoyed with the foot problem<br>Participant doesnt want to give up<br>Doesnt want to end up in wheel chair<br>Feels mentally better because of doing the trial and exercises

For peer review only

Would avoid Probus tour activities that involves lot of walking, not to embarrass others
Very satisifed' with improvements in walking<br>Wasnt going to Gym before. BOOST made the participant go.
Main concern is hip and knee pain<br>Down for hip surgery<br>Hoping to walk more after surgery &<br>believes surgery would sort the knee problem also
Participant feels not moving around would be fatal<br>Says 'Won't let' the leg pain stop the activities<br>Says, 'I won't be beaten. You just have to keep moving<br>Determined to keep moving<br>Pushes on & manages condition
Participant has lost bit of confidence in crossing roads<br>Has to push to meet people and do things<br>Feels that your whole confidence goes when you lose the ability to get around<br>Pain makes participant mentally tired<br>Pain gets participant down, so cant be spontaneous

For peer review only

Seeing more good days in terms of motivation to do activities<br>Still has good and bad days with low mood. But tries to be active and not to waste a day.<br>Confidence in doing things remains same since T2<br>Participant frightened that the condition would worsen and end up in wheel chair. So tries to be active.
General attitude to life has become 'very good' due to exercises<br>Feels mentally positive<br>Suggests it is important to know your limitations<br>Suggests it is important to be active
Coping better.<br>Manages and ignores pain well.<br>Has begun to enjoy life practically.<br>Has more positive outlook<br>Acknowledges to live with back problem.
Participant feels improved mentally<br>Participant would push to be more active<br>Emphasises importance of addressing depression linked with NC

For peer review only

Doesn’t worry about anything and carries on<br>Takes time and tries not to do everything in one go<br>Participant takes care and doesn’t do silly things. Example: Like skiing in bad weather

For peer review only

For peer review only

Psychological impact Trajectory
Negative impact remained throughout
No impact on mental health
Negative impact remained throughout

For peer review only

Progressive positive impact
Progressive positive impact
Positive impact that was maintained
Positive impact that was maintained

For peer review only

Progressive positive impact
Negative impact that remained throughout
Initial positive impact and back to baseline status<br>Impacted by other joint pain.
Iniital positive impact<br>followed by improvement.<br>Participant already had positive psychological impact<br>upon trial entry.
Initial positive impact that<br>was maintained

For peer review only

Progressive positive impact

Initial positive impact
that worsened

Initial negative impact
followed by positive
impact

Initial negative impact
followed by positive
impact

Progressive positive impact.
Participant already had positive psychological impact
upon trial entry.

For peer review only

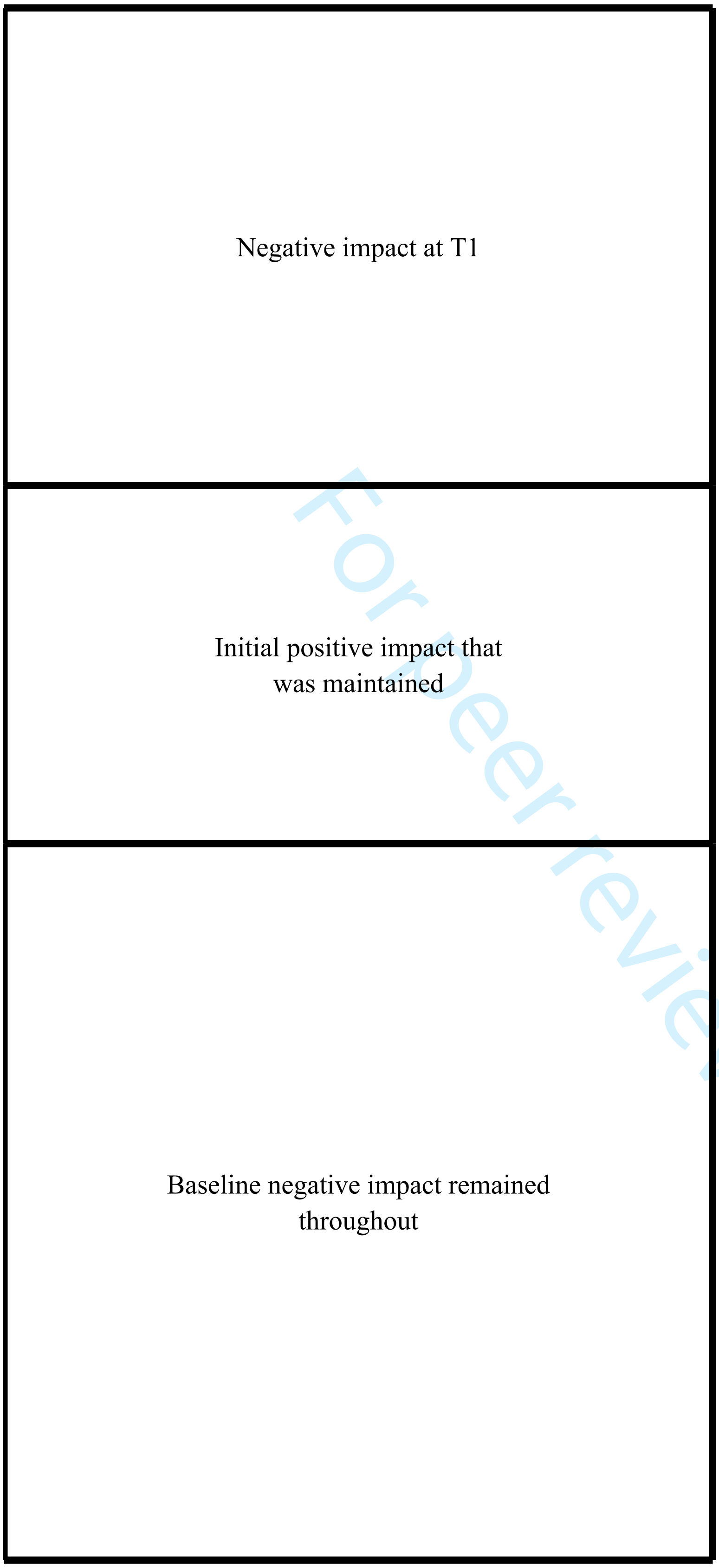

For peer review only

Initial positive impact that was maintained
Progressive positive impact
Baseline positive impact remained throughout. (Participant already had positive psychological impact upon trial entry.)
Baseline Positive psychological impact remained throughout. (Participant already had positive psychological impact upon trial entry.)
Initial positive impact but went back to baseline status

For peer review only

For peer review only - http://bmjopen.bmj.com/site/about/guidelines.xhtml

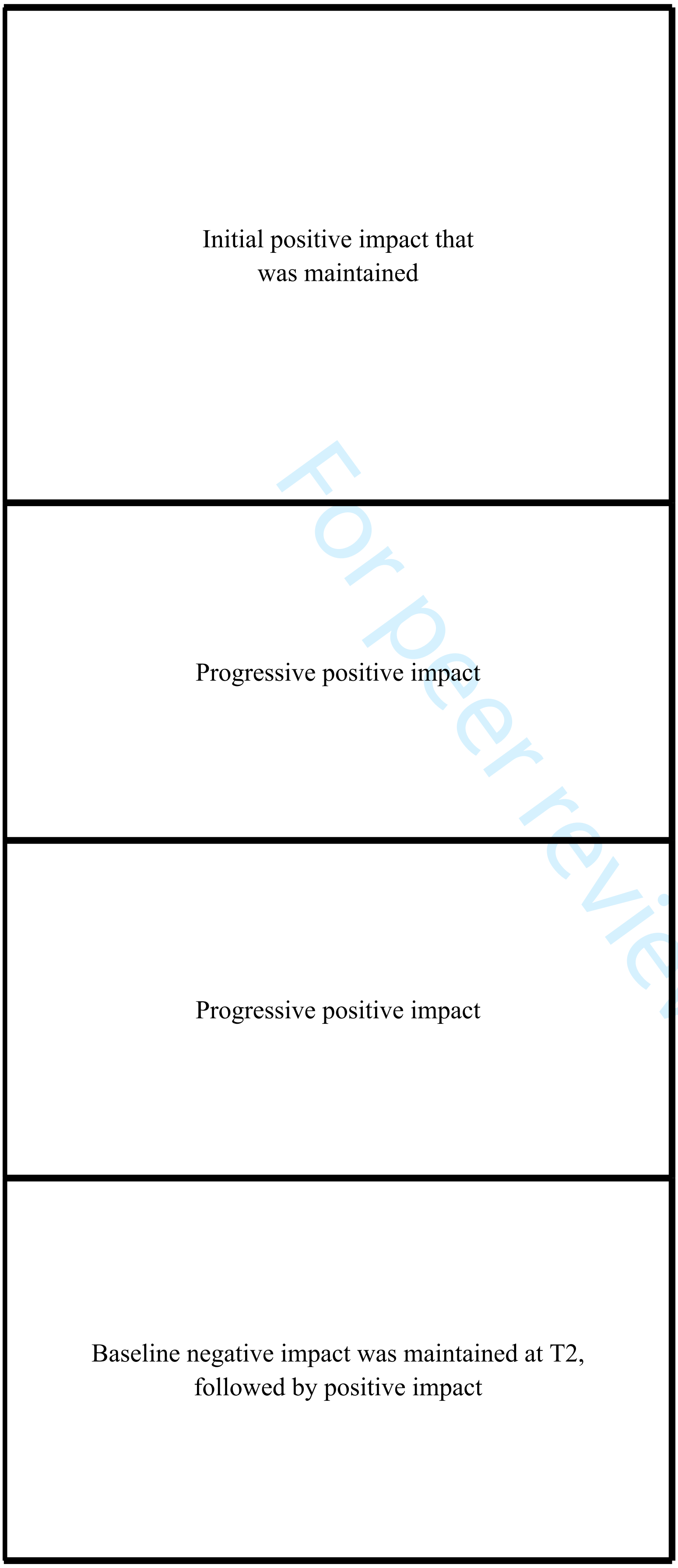

For peer review only

Baseline Positive psychological impact remained throughout (Participant already had positive psychological impact upon trial entry.)

For peer review only

For peer review only

Baseline<br>social & recreational participation
Difficulty with walking/standing longer made going on holidays difficult<br>Unable to process ideas for book (due to difficulty with walking)<br>Avoids gardening, house maintainence, long travels and rotary club activities
Impacted social life<br>Unable to mow the lawn<br>Avoids activities that involve physical activity, like going to city & around shops<br>Avoids social activities like shopping trips with friends<br>Attends weekly Pilates class (with modified exercises)<br>Stopped church voluntary café work
Goes to football match with son; shopping<br>Uses disabled seat; reliant on family to take to match due to concerns about falling<br>Unable to engage with grandchildren (coaching grandson football)

For peer review only

Weekly grocery shopping with spouse.<br>Cooks a family dinner on a Sunday.<br>Ocassional meet-ups with friends in town, reliant on spouse or public transport for travel.<br>Discouraged by others driving car or climbing up furniture to do cleaning
Socially active individual<br>But lack of motivation has,<br>Reduced shopping visits &<br>misses interacting with people<br>Difficulty with grocery shopping and garden maintainence<br>Unable to dance in social club<br>Plans to recommence swimming
Participant goes to local pensioners club every week<br>Balance issues prevents participant from dancing in club<br>Stopped visiting friend down the road<br>Stopped walking to local bus stop<br>Uses taxi for shopping<br>Less time spent in gardening
Unable to dance.

For peer review only

For peer review only - http://bmjopen.bmj.com/site/about/guidelines.xhtml

Ocassional photography field trips<br>Weekly photography class at local school<br>Goes shopping
Gardening activities reduced (being a prof gardener)<br>Unable to walk along canal<br>Unable to do the usual activities like plumbing, fitting, odd jobs<br>Enjoys car boot outings, sitting in the car
Condition has 'Taken participant's social life'<br>Mobility limits social participation<br>Unable to engage in social outings with friends<br>Unable to walk further during holidays<br>No longer able to go walking in countryside with spouse<br>Does some of her social activities (needle work) in sitting
Loves going out<br>Does usual social activities<br>Goes to weekly Taichi class & voluntary work at church<br>Difficulty with household activities<br>clothes line, pull trolley uphill<br>doing curtains, lift heavy items<br>Unable to help friend<br>Reduced the gardening hours
Pain limits almost all activities<br>Slowed down with gardening and being active with grandson<br>Reduced going out to avoid pain

For peer review only

Participant stopped driving<br>Mental obstacles in taking the bus<br>Depends on friends for outings<br>Difficulty with gardening as unable to kneel (hoping to do some gardening)<br>Unable to dance<br>Unable to clip tables at WI
Pain affects holidays when needed to walk<br>Prefers not to be active in social activities as he cant cope with presssures anymore<br>Reduced yearly holiday trips as spouse worries he cant cope up at airports
Unable to play squash
No variety in activity'<br>Spends time in academic libraries<br>Does embroidery/ cross-stitch<br>One day voluntary week<br>Attends events/meetings<br>Main impact: Physical condition interferes with the ability to do field trips<br>Unable to keep up with spouse's pace while walking
Fairly active lifestyle<br>Meets friends every week<br>Does lot of painting and drawing<br>Works on alottment area<br>Enjoys growing tomatoes in alottment area<br>(Does lot of digging and lifting)<br>3-4 hours, 5-6 days per week

For peer review only

5-6 hours work in the farm usually, but unable to do much now.<br>Sold all the ewes because struggling to lamb them due to hip and back problem<br>Unable to turn the sheep and do their feet
Difficulty with walking does not stop holidays<br>Happy 'inside out' going to ladies club every week<br>Does simple exercises for legs and arms during club visits<br>Goes on trips with ladies club<br>Not able to cook standing, so sits on chair<br>Not able to go anywhere on own<br>Takes her own time and often gets pain during holiday trips<br>Unable to do yoga due to joint stiffness
Difficulty walking in the long garden<br>Does not drive as before<br>Not going out much in car or meet friends<br>Reduced the evening outings<br>Cannot party as before<br>Difficulty going to airport that stops the holidays abroad<br>Feels unsafe walking along beaches during holidays (fear of fall due to leg weakness)

For peer review only

Impact of NC is very minimal<br>No longer ablet to go for Probus walking trips due to heaviness/discomfort in legs
Active life: Dog walk daily, TESCO weekly, Meals twice a week<br>Pain is restricting lots of activities<br>NC restricts gardening<br>Difficulty standing in queques in airports<br>Stopped taking dog for extended walks<br>Stopped going to pub uphill due to pain<br>Stopped house decorating due to balance issues<br>Finds a seat to sit down in airports
Stopped going on holidays abroad<br>Hires mobility scooters & hotels with walkin showers during holidays
Socially active (garden, Zumba, Silverline, voluntary work at school listening to children reading, friends)<br>Spends 2 hours viewing the estate & gardening activities<br>Manages garden activities on sitting/taking help/pacing<br>Takes rest in between Zumba class<br>Finds ways to manage back problem during holidays (taxi)
Participant feels not to be bothered (ex. Stopping going for Christmas sale and meeting friend)

For peer review only

Feels restricted in most of things got to do<br>Unable to walk the dog across the fields<br>Unable to use the mountain bike<br>Cannot work on machinery and vehicles as before<br>Unable to climb ladders anymore
Difficulty with outdoor life & gardening<br>Takes breaks during housework<br>Unable to go long walks with friends<br>Unable to stand for a while & talk to people; so need to find a seat
Condition affected participant's whole enjoyment of going out<br>Ability to cook meals has gone<br>Condition strained the relationship with spouse
Back problem destroyed family life and general lifestyle<br>Condition left participant unable to take part in social outings<br>Involves in fixing model trains & coin collection hobbies

For peer review only

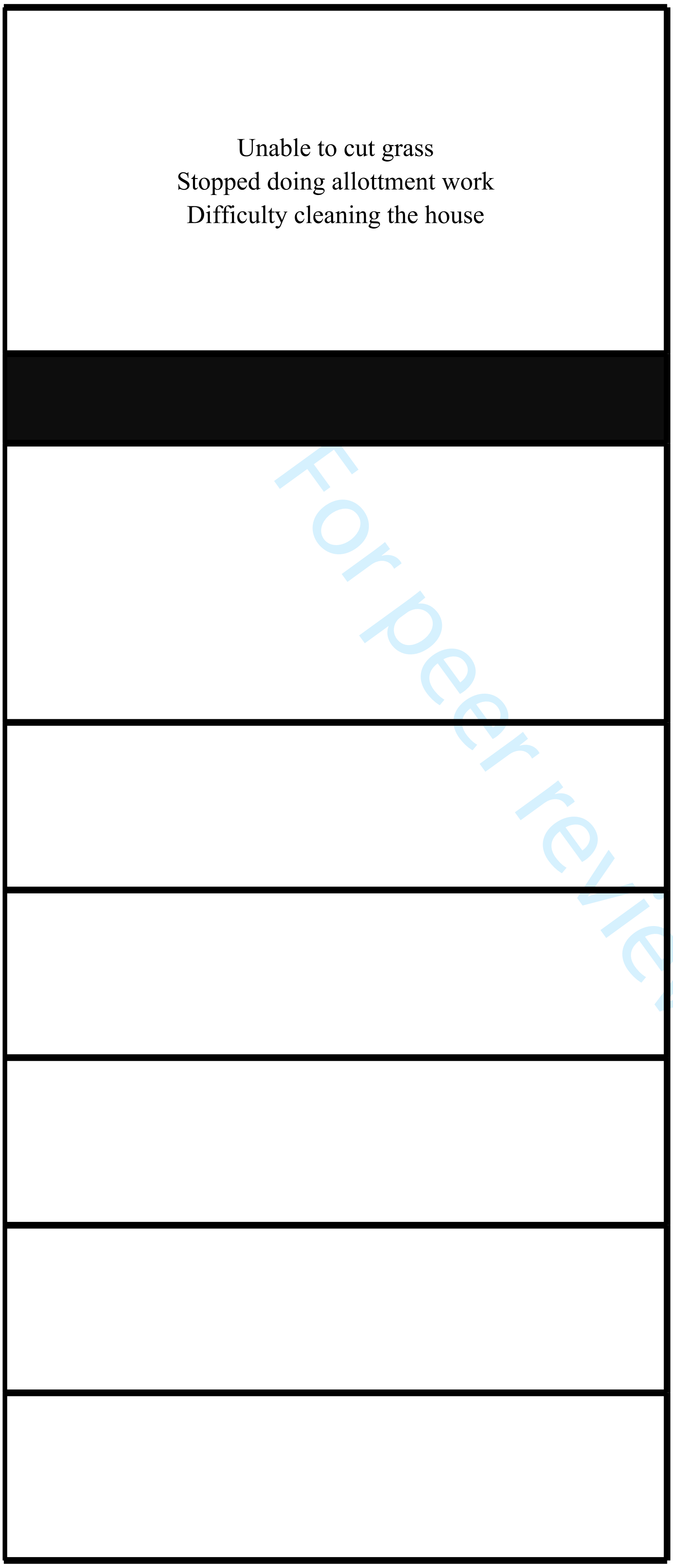

For peer review only

For peer review only

6 months
No information
No change in participation restrictions
Shopping with spouse;<br>Foot ball outings halted (end of football season)<br>Avoids going out due to<br>discomfort

For peer review only

Cooks a Sunday dinner for family. Meets up with friends for lunch in town.<br>Prevented by others to take turns at getting drinks, carrying tray
Pleased with socially active levels<br>Resumed dancing in local club; Increased ability to mow lawn (one day)<br>Resumed grocery shopping<br>Plans to recommence swimming
Increased ability to walk to friend down the road, resumed shopping<br>independantly, attend pensioners club and dance;<br>Reduced ability to do gardening
Participant is able to take the dog for a walk.<br>Able to do more housework.<br>Able to do more gardening but paces them<br>Able to involve more in family outings and holidays.<br>Unable to carry shopping bags.

For peer review only

Doing lot of indoor photography
Regular daily outings driving to shopping<br>Regularly attends car boot sales, uses sticks<br>Parks closer to destinations<br>Unable to do gardening activities that involve heavy lifting or bending<br>Carries out light gardening activities
Goes weekly art class<br>Regularly walks 20-30 minutes with spouse and friends<br>Outdoor access has increased with blue badge parking
Not doing any new activities since T1<br>Things are more or less the same since T1<br>Reduced gardening due to cold weather
Feels better with improved activity levels<br>Lot more active than used to be<br>Walks to main road with grandson<br>Pain still stopping participant to be active (go tourist places)<br>Participant does little more each day by pacing approach<br>Attributes physical improvements 100% to BOOST

For peer review only

Uses bus with confidence- relates to been given seceond life<br>Manages bit of gardening<br>Attends communion without stick<br>Unable to move tables and chairs at WI
Difficulty with walking during<br>recent holiday trip, managed by taking taxi
Doing all usual activities except squash<br>Struggles with cooking and washing due to losing control on balance
Walking is slow (e.g. strolls) and if participant was with a group doing things (archaeological field trips), it is difficult to keep up.<br>Participant makes travel plans ahead
Did a lot of walking in recent holiday<br>Does a lot of digging, bending & lifting<br>Built a chicken coop with 8 chickens

For peer review only

Not doing much in the farm<br>More pain during walking and bending activities
Doing same usual activities<br>Not tried any new activities<br>Lifestyle remains same<br>Goes to local club; does simple exercises<br>Can stand and cook now<br>Not doing any gardening because it involves bending activities
Declines going out for meals to keep life easy<br>Feels she is not missing any activities<br>Went to Spain recently & managed by sitting down frequently<br>Believes she can travel abroad with stick

For peer review only

Impact of leg pain on walking on general lifestyle is very minimal<br>Would avoid Probus tour activities that involves lot of walking, not to embarrass others
Feeling 'marvellous' with present condition<br>Doing same usual activities, but with no pain<br>Pleased with ability to walk dog around block<br>Able to walk the dog round the block 'quite easily'<br>Hoping to do some gardening
No information available
Still doing things as before
Participant walked well during recent holidays

For peer review only

Participant resumed using mountain bike (one of treatment targets)<br>Day to day activities is lot better-So able to do more gardening<br>Would consider voluntary work if walking improves further<br>Decorated the kitchen (since T1)<br>Able to do more cooking than before, leaning on kitchen cabinet<br>Has grandson and grand daughter twice a week<br>More active now<br>More satisfied now because of understanding the condition it was months before<br>Goes for long walks with dog up to one mile without any problem<br>Built (since T1) a store on own for winter wood and generator<br>Enthusiasm is low (participant not sure if due to health issues
Tries to do some gardening within own abilities<br>Can stand and talk to somebody without thinking to sit or lean on something
Tries to do some gardening by pacing
No information available.

For peer review only

Told by doctors not to do housework/mow the lawns, but try and do something<br>Mowing the lawn was 'very difficult' to mow the lawn.Participant ignored pain and took breaks to finish.<br>Participant's holiday was good. The only problem was sitting for hours on the plane can be uncomfortable.<br>Asked for special services (wheel chair) while checking in
[redacted]

For peer review only

12 months
Avoids activities like gardening.<br>Limited ability to travel long-distances
Decompression laminectomy, since T2:<br>No change in participation restrictions (Reduced voluntary work and attendance (church);<br>Stopped attending walking group<br>Reduced pace with Pilates class)
Stopped going out for drink with son<br>Stopped shopping<br>Unable to sit and laugh and joke with people (socialise) due to pain<br>Avoids going out (fear of falling)<br>Misses involving with grandson's football sport

For peer review only

Increased independance to go shopping, social outings, carry laundry, access train stations<br>Able to take turns during social outings (getting coffee, carrying tray to table)<br>Others giving more independance<br>Walking to different places (shops, gardens) improved participation<br>Cooks a Sunday dinner for family. Meets up with friends for lunch in town.
Further increased ability to mow lawn (one session) and dance longer.<br>Uses stairs when lifts are out<br>Intends to resume swimming soon<br>Increased walking distance on a recent holiday
No change. Increased ability to walk to friend down the road.<br>Reduced dancing at pensioners club<br>Uses taxi for shopping<br>Reduced ability to garden (maintained)
Participant is still taking the dog out<br>Began swimming at a local gym<br>Manages walking during holidays<br>No further information

For peer review only

Emergency abdominal surgery six months ago:<br>Activities restricted mainly due to recent emergency surgery and time of year (winter time)<br>Continues to go for shopping<br>Helps spouse in household activities<br>Able to re-engage with longer walks with spouse- up to 3 to 5 miles, compared to T1<br>Able to walk for leisure, including local walks and holidays
Overall activity impacted by leg and lung condition. Activity has reduced almost 50% since T1.<br>Generally less active during winter<br>Stopped most of the usual activities<br>Stopped gardening for a while<br>Attends an occasional car boot sale; mainly staying seated in the car<br>Less active due to leg & lung condition, winter weather<br>No problem with driving. Drives spouse to do shopping once a week
Level of activity redued due to compounding MSK issues<br>Takes help from spouse in house work
Doing same usual activities before T1
Note: Spinal fusion surgery (just after Interview 2) for NC<br>Had Cauda equina syndrome & became double incontinent<br>Describes as 'rest & recuperation' time<br>Mobility within house with walking frame<br>Goes for little walks once or twice a day with spouse

For peer review only

Before T3, had lumbar decompression L4, 5<br>Participant 'Amazed' with the progress made<br>Able to do gardening, walk without stick at times
Managed recent holiday with short walks<br>with lot of rest in between. Had no problem getting in to the airport
Compared to T1, now totally independent<br>with rollator going shopping or using bus<br>Unable to go to foot ball matches due to difficulty negotiating stands and crowd
Main impact: Restricitons on archealogical site visits (main pleasure)<br>Walking problems are not impacting social activities
Goes to art classes<br>Not much work on the allotment (winter time).<br>Activities are relatively constant since T1

For peer review only

Back surgery 7 weeks before T3 interview<br>Not doing much in the farm<br>General lifestyle is much better<br>compared to T1
Cooks for everyone<br>Goes to ladies club every week<br>Goes to daughter in law's place every week<br>Used wheelchair throughout flight and airport during a recent holiday trip
Doesn’t mind going on holidays<br>Avoids going to big shops (tiredness & leg pain)<br>Foot pain stopping participant to go out with friends for tea

For peer review only

Impact of leg pain on walking on general lifestyle is very minimal<br>Would avoid Probus tour activities that involves lot of walking<br>Cannot swim as before
Lifestyle & activities 'much better'<br>Now able to walk around shops with spouse<br>Now able to walk dog around block & fields (Notable improvement)<br>Going to gym
No information available
Still doing things as before (gardening, meets friends, school voluntary work)<br>Some Zumba exercises have become difficult, sits before continuing further
Participant has to take effort to meet people and do things.<br>Participant thinks about walking ability and feeling old when walking with family

For peer review only

Still able to walk the dog<br>Still has grandson and grand daughter over twice a week<br>Still uses the mountain bike<br>Managed to decorate the lounge, stairs, dining and bedrooms
Goes out with friends<br>Goes to WI once a month<br>Aiming to go on holidays (on hold due to Addison's)
Able to take daughter's childern to and fro school (80 miles a day)<br>Can walk down the garden with stroller<br>Got involved in mind programme (memory club)-sing along and mental games.
General lifestyle hasnt changed much since T1.<br>Continues hobbies such as fixing trains, boats & watches

For peer review only

Able to do day-to-day activities (cook meals, do laundry) without depending on others.<br>Goes shopping with family<br>Mobility and activities haven't worsened

For peer review only

For peer review only

Social & recreational participation Trajectory
Participation restrictions remained throughout
Participation restrictions remained throughout
Progressive worsening. Participant already had positive participation upon trial entry.

For peer review only

Progressive improvement.<br>Participant already had positive participation upon trial entry.
Progressive improvement
Initial increase in participation that was maintained
Progressive improvement

For peer review only

Progressive improvement.
Participant already had positive participation
upon trial entry.

Baseline participation restrictions that got worsened at T3

Initial increase in participation and back to
baseline status. Impacted by other joint pain

Baseline level of positive participation
remained throughout. Participant already had positive
participation
upon trial entry.

Initial increase in participation that
worsened due to other condition

For peer review only

Progressive improvement

Participation restrictions remained throughout

Initial restriction followed
by improvement

Baseline level of positive participation
that remained throughout. Participant already had positive participation
upon trial entry.

Baseline level of positive participation
that remained throughout. Participant already had positive participation
upon trial entry.

For peer review only

Participation restrictions remained throughout
Baseline level of positive participation that remained throughout. Participant already had positive participation upon trial entry.
Participation restrictions remained throughout

For peer review only

Participation restrictions remained throughout. However, the impact of leg pain is minimal across time.
Progressive improvement
No information available
Baseline level of positive participation that remained throughout. Participant already had positive participation upon trial entry.
Initial increase in participation and back to baseline status.

For peer review only

Initial increase in participation that was maintained
Progressive improvement
Progressive improvement
Participation restrictions remained same at T3<br>No data at T2

For peer review only

Baseline participation restrictions remained throughout

For peer review only

For peer review only

General health events
No information-Throat narrowing having a major social impact-On external feeding tube
Hip and leg pain-No info- Leg pain intensified after surgery
Complex multiple issues, Unclear health concern (NC or CS?)-diagnosed Cervical stenosis-Main concern CS

For peer review only

Fine-Unchanged-Unchanged
Pretty good (but main concern is NC)-Improved 50-60% due to increased mobility (Pleased with physical and social activity)-Great
Not too bad-Pain & mobility issues are OA related- Been careful with mobility after recent fall
Deterioration in last 2 years-Reasonably healthy-Knee pain dominates other issues (Stenosis has got better)

For peer review only

Quite good-Unchanged (leg condition is central hub affecting many areas of life)-Major emergency surgery for abdominal condiiton
Not too bad (got anxiety, NC is major concern)- Feels well (monthly B12 Inj)- Not good (lung condition is major concern)
No concern- Knee pain aggravated by sit to stand BOOST exercise (main concern) -Bad stage (multiple MSK issues + recent fall, deterioration in mental health, main concern knee pain requiring surgery)
NC pain is main concern- No major concern-Doing well
Main concern-back problem Not good (morbidly obese, History of previous 2 falls) Arthritis in other joints increased due to bending activites with moving house- to get the right leg stronger & normal bowel/bladder function

For peer review only

Happy life-<br>Blood test for kidney problem-<br>Feeling great (Had L4 L5 decompression surgery)
Main concerns:<br>Back & ankle problem<br>- Back & ankle problem<br>-Back problem
Main concerns:<br>Leg condition-<br>No information-<br>No information
Totally healthy-General health is okay-<br>Back problem is main concern
Relatively good health-Fine-<br>Not too bad<br>T1: Main concern is stenosis pain

For peer review only

Main concern: Back, shoulder, and hip pain
Main concerns:<br>Legs, back and ankles-Some<br>days chronically fatigued-<br>Lack of vitality and fatigue related<br>to back problem
Leg weakness is main concern-Good<br>& bad days, no other health or family problems-<br>Chronic foot pain is main concern

For peer review only

Participant quite happy with the general health except the difficulty with walking to shops & cataract- No information available-Balance problem & exhaustion is main concern
Main concern: To get rid of leg & back pain- No information- No information
Main concern: Knee & hand pain- Knee problem rather than the back- Waiting for hip surgery
Main concern: Back & gut-No information -No information.
Main concern: Legs, whole body (mentally & physically)-No information- Aches & heaviness in legs

For peer review only

Main concern: Pretty good-Pulmonary blockage and 2 days hospital stay-No information
Main concern: Back problem-No information-No information
Main concern: Back problem due to depression-No information-No information
Main concern: Ocassional episodes of depression-Right leg weakness causing participant stumble often-

For peer review only

Good-Same as T1-Thinks had an improvement

For peer review only

For peer review only

Co-morbid conditions
Total 2. Hypertension; throat constriction causing swallowing issues
No comorbid issues reported.
>5. Complex multi-morbid presentation of physical and mental health issues. Hypercholesterolemia, hypertension, stomach ulcer, irritable bowel, Barrett's oesophagus, urinary incontinence, and persistent headache; Arthritis, cervical stenosis, Depression, OCD, suicidal thoughts

For peer review only

Total 5. Epilepsy; hiatus hernia; seasonal affective disorder (SAD); Scoliosis; Knee OA
Total 3. Asthma; Hypertension; Hip OA
Total 2. RA; Knee OA
>5. Stomach issues (including ulcers); Bell 's palsy (with associated neck spasms); type 2 diabetes (3-4 years); laryngospasm; otosclerosis causing deafness in right ear; aneurysm (actively monitored). Multi-site chronic pain (30+ years; hands, knees [arthritis??], lower back); plantar fasciitis (20+ years); fractured vertebrae (T12, 5 years ago); bilateral trochanteric bursitis;

For peer review only

Total 5. Barrett’s oesophagus (5+ years); on cusp of high cholesterol; obese (Body Mass Index 31); digestive issues.<br>Historic back surgery.
>5. Multiple, historic physical morbidities, and recent mental health issues<br>associated with NC-related pain and mobility issues<br>Historic hip replacement resulting in use of two arm sticks to walk; historic back injury;<br>chronic osteoarthritis; recent prostate issues; urge incontinence;<br>(~ 8 medications daily).
Total 2. Chronic pain from a partial left knee replacement 7 years previously.<br>Acute neck pain (<2 months), impacting sleep
None noted
>5. Previous history of heart attack and kidney shut down, and a<br>blood clot on the heart, and septicaemia.<br>Minor balance problems due to congenital partial deafness<br>History of hip arthritis and replacements<br>Arthritis in shoulders, knees, back

For peer review only

Total 4. Hiatus hernia,<br>Heart murmur,<br>heart valve problem,<br>Bilateral knee replacements
Total 4. Type 2 diabetes<br>Previous bilateral shoulder replacements<br>Ankle injury in teen years<br>Ankle arthritis
Total 5. Vision issues; hypertension,<br>high cholesterol; slight asthma.<br>Had heart valve replacement<br>and single bypass
Total 2. Bowel problem<br>Weakness in right ankle
Total 4. High BP; Deafness;<br>Previous fall; Arthritis spine

For peer review only

Total 3. Parkinsons; Memory problems<br>Previous hip replacement
>5. Atrial fibrillation; hypertension;<br>high cholesterol; numbness in hands; Plantar fascitis;<br>Historic sciatic nerve
>5. Chest pain, Hypertension,<br>Chest pain, Blood pressure,<br>Glaucoma, Visibly swollen joints in hands<br>Foot arthritis<br>Historic hip replacement

For peer review only

Total 4. Cataracts; Indigestion;<br>Heart triple bypass; knee injury
Total 3. BP, Cholesterol; Previous<br>history of Frozen shoulder
> 5. Bronchial asthma & MSK issues include whiplash, causing<br>pain and mobility issues;<br>foot pain, impacting mobility; knee pain (two knee<br>replacements);<br>hand pain; Coccyx injury?
Total 2.<br>Ashtma & Cervical spondylosis.
Total 5. Drooping eyelids; Diverticulosis;<br>Claustrophobia; Bilateral hip replacements; Arthritis fingers

For peer review only

Total 3. Type 2 diabetes; hypertension; heart aneurysm
Total 4.<br>Addison's disease (well managed)<br>Asthma (Reasonably controlled)<br>Osteoporosis<br>Arthritis in other joints
Total 3.<br>Diabetes; Meniere disease;<br>Osteoarthritis of hips, knees and feet.
> 5.<br>COPD; Depression;hypothyroid; hand OA (joint disfigurement);<br>recent bladder and bowel incontinence. Poor appetite.

For peer review only

Hypertension, cholesterol, and ‘prostate’
Arthritis in hip and shoulder

[redacted]

For peer review only

For peer review only

Coping strategies
Adopts graded activity approach (breaks 30 minute walk into shorter walks); Pacing (sits down)<br>No new strategies adopted over time.
Takes help from others: hired a person to maintain lawns and prune shrubs; daughter cleans stairs<br>After T1, Parks car away from destination, to integrate walking to shopping<br>Avoids doing stairs unnecssarily at home.<br>Modified social outings to reduce walking and standing
At T1, Moved from a two-storey house to the bungalow as unable to manage stairs.

For peer review only

After T1, Adapts ADLs (climibing stairs) & house hold chores (carrying laundry basket) to build as exercise.
Participant uses various coping strategies in T1, T2, T3.<br>Plans shopping trips; Shops in smaller volumes; Uses rucksack instead of shopping bags; Uses Activity pacing
From T2, pacing, exercise, and environmental adaptations<br>Plans the day<br>Avoids too much activity<br>Breaks tasks into smaller parts<br>Adopts pacing<br>Uses flexion exercises<br>Does environmental adaptations (perennial plants)
Activity pacing<br>Gardening for short period; and keeps things at waist-height to avoid bending.<br>No new strategies adopted after T2.

For peer review only

No information
Has someone to help in gardening<br>Uses downstairs bathroom<br>Learned to adjust to physical limitations and pain<br>Uses garden fork for balance; table to pot plants in garden<br>Parks closer to destinations
At T1, Adopts pacing and resting while doing housework<br>Reluctant to modify home, use walking aids<br>Adopts pacing and resting while doing housework<br>Plans social outings -to access seating, close parking<br>Sometimes overdoes things and ends up in pain the next day<br><br>After T1, continues pacing and resting<br>Uses walking poles for increased pace<br>Nordic sticks for posture and even weight distribution<br>Wears a girdle; Uses Fitbit reminder to be mobile
From T1, Participant has been using various strategies:<br>Plans the outings based on bus routes<br>Takes help from son to cut garden grass<br>Takes help from someone to lay lawns<br>Uses stair lift for independance<br>Avoids activities (lifting heavy items) that cause pain
Participant has been using various strategies at T1<br>At T1, Plans to move to new home to avoid using stairs<br>Modified the big garden so it is easy to manage<br>Uses hosepipe instead of water can<br>Takes frequent stops to avoid pain<br>Lumbar support and headrest in car<br>At T2, reports pacing, pausing and doing things within pain limits<br>Takes help from spouse (work as a team)

For peer review only

Adaptations since T1<br>From T1, Depends on people and gadgets
At T1, Takes help from others during family gatherings<br>Uses orthopaedic beds<br>Stool to use in kitchen<br>Takes taxi during holiday trips<br>After T1, Participant is careful about doing things and takes rest if needed
At T1, sits down while numbness develops while walking to avoid a fall<br>After T1, uses lawn mower to lean; sits in garden<br>Stooped posture while walking<br>Looks for disability space
At T1, Does online shopping to avoid markets<br>Spouse drops and picks up<br>Spouse does most of housework<br>Supports on bannisters to avoid falling<br>Paces to do OWN things<br><br>After T1, Avoids doing too much of activity<br>Plans travel ahead
While digging in garden, uses left leg to put pressure<br>Avoids activities that causes pain (twisting or overstretching)

For peer review only

At T1, Neighbour takes care of sheep<br>Uses vehicle to go uphill<br>Doesnt walk more to avoid pain<br>Drives tractor instead of walking<br>Uses machine to do sheep's feet and takes help from spouse<br><br>After T1, Takes help from someone to take care of sheep<br>Breaks down tasks<br>Takes help from daughter with loading work
At T1, Tries to keep holidays easy by not taking long walks<br>Uses chair to sit down during walking<br>Takes help from friends<br>After T1, Avoids activities that cause pain<br>Uses wheelchair throughout flight<br>Does activities at own pace<br>Depends on family on long travels
At T1, Stops and sits down to manage pain while walking<br>After T2, Participant keeps life as easy as possible (avoid meal outs)

For peer review only

At T1, Has become socially selective
Avoids social walks that includes hills
Uses car instead of walking
Uses supermarket trolley
After T1, Takes long strides to manage the walking problem
Keeps life easy as possible
Participant plans life around own abilities

Depends on colleagues to lift heavy items
Finds a seat to sit in airports
Tends not to walk far
Tries to be careful lifting heavy items
Opts for car wash

At T1, Uses catalogue shopping
Uses push on trolley while shopping
After T1, Takes help from others
Doesnt overdo activities to avoid pain
Uses grabber aid to pick things from floor
Plans Meals on wheels to avoid cooking

Note: Participant actively adopts a variety of coping strategies throughout
Takes a taxi during bad weather, or trips with multiple changes in buses.
Online shopping to avoid shopping trips
Quick meals to avoid standing & cooking
Paces during gardening activities
Takes help from others
Avoids bending or stooping activities

At T1, Stooped posture during pain while walking
Spouse does most housework

After T1, Stops and rests during walking
Uses hypnotherapy app for motivation
Takes long strides to help pain

For peer review only

At T1, role reversal -hoovering while spouse takes dog out<br>Parks closer to destination to avoid walking back<br>Hops rotavator instead of walking forward and backward (to avoid too much walking)<br>After T1, Uses a mobile app to track physical activity<br>Learnt to break up tasks<br>Learnt about symptoms of NC<br>Uses wheelie bins to lean on while doing BOOST exercises<br>Plans activities to make them easy as they can (example, moving some old Belfast style sinks in the garden)
Participant does online shopping<br>Takes breaks during housework<br>Uses light weight vacuum<br>Plans walking distance & seats to sit<br>Gets help with gardening<br>Doesnt overdo things; Takes breaks
Participant uses walker with seat to stop and sit<br>Participant paces while gardening<br>Takes help from son in gardening
Grandson helps with hobby (fixing trains)<br>Sits on swing to collect ball from dog<br>Uses pedal exerciser to deal with leg weakness

For peer review only

Participant doesnt do any strenous activity<br>Distracts from pain by doing something he enjoys.<br>Always uses a comfortable posture and adapts sitting.<br>Goes shopping with family to avoid carrying<br>Uses a back support while driving<br>Takes time and tries not to do everything in one go

For peer review only

For peer review only

**Views on aging**
Current condition is mainly pain -related (90%) & 10% due to aging<br>Priortises being independant than aging<br>Accepts aging and content with overall health<br>Feels less capable than other people of same age
Aging is inevitable, but staying active helps staying young<br>NC is age related, accepts living with it<br>Health issues are part of aging<br>Activity is good for aging people
Physical ability has nothing to do with age<br>Physical condition will worsen on aging<br>Living with a chronic pain condition makes participant feel older than actual age<br>Due to the condition, participant feels not as active for age<br>Being dependant on others make participant feel older for age.

For peer review only

Feels younger in mind but keeps reminding that it is not so.<br>Public signage referring to older portrays them as physically disabled.
Positve attitude to aging<br>Participant considers active than peers
Physical decline cannot be reversed with aging.<br>Increased dependance on others<br>Pacing is part of aging<br>Participant thinks doing better than other younger people<br>Views NC as age related<br>Would stay active as long as possible
MSK issues and deterioration are due to aging;<br>Attitudes have a role in dealing with a health condition.<br>Aging related to health decline and impact on physical activity.

For peer review only

Age is just a number.
Positive outlook keeps young
Determined to engage in hobbies and activity regardles of aging

Participant feels 'Aging slows you down'
Feels capable of getting stronger at this age
Mobility issues makes participant feel older
Physical decline is mismatch bet physical ability and mental ability

Participant doesn’t want to be defined by age
Relates reduced ability as life style related rather to aging
Has a proactive attitude to take care of health while aging
Positive attitude keeps you younger

Participant proud of age
Feels lucky to have reached 80 & move around
Pleased with activity levels
Thinks more active
Activity restrictions are due to pain, not aging

Aging has less impact than the health condition on slowing down

For peer review only

Feels like 21 mentally<br>Accepts slowing down with aging<br>Back condition is related to aging<br>Participant feels still able to do everything
Unable to cope up with pressures with aging<br>Relates reduced ability to run social events to aging<br>Feels still young<br>Feels not bad for own age<br>Sees people worse or better of same age<br>Steps back from roles in charity boards
Doesn’t feel 76 years old<br>Doesn’t feel unhealthy<br>Participant feels to get back to squash<br>Participant fine with activity levels for own age<br>Feels guilty about people smiling and opening doors<br>Doesn’t consider disabled<br>Feels fitter
Considers healthy for age<br>Slow walking pace compared to others<br>Does not accept aging as reason for the deterioration<br>Not overly consious of aging process<br>Expects to live upto 90 years (comes from healthy family)
Aware of health problems with aging<br>Self help may not be helpful for older people<br>Participant views aging as natural process but determined not to be influenced by it<br>OA knee is common in older people than NC<br>Participant thinks the current activity levels are good for own age

For peer review only

Older people work less
Participant feels the condition is not severe enough for surgery
Not keen on surgery at this age
Participant feels there is slowing down than before
Feels activity levels are right for own age

Some say participant looks like 60 year old
Accepts physical decline as part of aging
Believes in what can/cannot be done at old age
Feels not active compared to others of same age
Health issues limits things though active for age
Thinks 72 is not old age
Thinks health condition is age related and would want to try non surgical options

Accepts aging
Relates loss of physical ability to aging
Doesn’t want surgery at this age
Tends to be extra careful crossing roads due to age
Happy with current level of activity (going out for meals is not important anymore)
Feels lucky compared to friends of same age who are not doing well

For peer review only

Participant feels younger than own age<br>Others tell participant looks younger and active<br>Feels active & lucky compared to neighbour with stooped posture<br>Relates aging to physical limitations, difficulty going upstairs, balance problems, walking long distances<br>Sees no reason why physical health cannot be improved at this age
Participant doesnt want to use walking aids as it makes look older<br>Relates physical decline to aging<br>Participant admits getting older<br>Others dont believe participant's age<br>Feels younger
Participant believes 'Aging slows you down'<br>At old age, 2 minutes is a long duration of time<br>Older people must exercise<br>Believes surgery alone can sort the problem<br>Not keen on surgery due to age
Participant doesnt feel older<br>Feels doing well in mobility & activity for own age<br>Feels mentally younger<br>Aging slows you down<br>Gets upset (soul destroying) when told about a health condition 'its your age'
Participant feels not older mentally<br>Getting older physically due to leg symptoms<br>Participant feels not to be bothered at this age<br>Feels it takes time to become self-motivated at this age

For peer review only

Participant feels younger
Anxious about how longer would live
Going abroad for holidays is not a good idea at old age
Associates rollator to aging and doesnt want to use it
Describes enjoying own company at this age
Afraid of sitting around as getting older
Aging modifies one's physical and mental aspects
Participant confident about improving physical ability at this age
Considers aging as a reason for limitations but not an excuse
Being less active reduces general level of fitness
Relates tiredness after sternous work to aging
Frustrated due to inability to move around like used to, because of aging

Aging modifies one's physical and mental aspects
Participant confident about improving physical ability
Considers aging as a reason for limitations but not an excuse
Being less active reduces general level of fitness

Participant expected to be more healthy for own age, coming from healthy family line
Never thought he got to the stage with walker.

Participant doesnt like getting older
Participant considers the health condition seperate from aging
Describes back problem can occur at any age

For peer review only

People tell participant looks younger for the age.
Participant feels to still be active and improve physical activity
Feels fortunate to do things, watch programmes and cook meals which are impossible for some other people.

For peer review only

For peer review only

Use of mobility aids
Does not like using aids because it makes them feel old.
Not interested in trying a mobility aid as did not want to draw attention.
Participant used crutches inside and outside home and accompanied by family members at outings.<br>Stopped using crutches by T3.

For peer review only

Uses walking sticks for longer distances (around garden centres)
Uses a folding stick on holidays of uneven terrain as a precautionary measure.<br>Does not want to depend on walking stick.<br>Uses stick to increase pace or to walk long distances.
Unable to use aids (walking stick) due to RA in wrists
Uses walking poles.

For peer review only

Not using any aids
Participant uses walking stick for short distances.<br>Uses two arm sticks for longer distances.<br>Not willing to try walking frame.<br>At T3, spouse annoyed as participant repeatedly puts at a higher risk of fall when walking due to not using the most appropriate mobility aid.<br>This has led to a behaviour change, with Participant using arm sticks more often when going out.
Reluctant to use- vanity, reduce independance<br>doesnt like forced socialisation<br>Prefers to use shooting stick or trolley for balance
Uses walking stick<br>Ocassionally uses stair lift at home
Uses walking stick when going outside<br><br>Since T2, had CE and used walking frame within house

For peer review only

Tries to walk inside without aids<br>Uses Zimmer frame to go to toilet<br>Uses walking stick outside
Not using any aids as he is too proud to use
Uses wheeled walking aid with seat (rollator) to sit down when numbness develops rather than for walking
Uses walking sticks<br>ocassionally- Progressed with walking with a stick by T3<br>Uses telescopic poles during field trips<br>Uses boots on rougher sites to stabilise weak right ankle
No information available

For peer review only

Not using any aid
Uses a foldable stick for balance<br>Uses wheelchair at airports
Does not use walking stick at home<br>Uses stick outside and on holidays

For peer review only

Likes to be independent.<br>But carries a stick for safety reasons,<br>during snow.
Not using walking aid<br>Didnt want to use-makes<br>look older
Uses mobility scooter for shopping<br>Also has a wheel chair & 3-walker
Doesnt use any walking aid.
Avoids using walking stick.<br>But carries a folded stick while holidays to Spain.

For peer review only

Participant walks without any aids;<br>Would take a stick if needed to walk more.<br>Would not use rollator as it makes look older
Always uses walking stick outside.
Uses a wheeled walker with a seat to<br>sit whenever wants to.
Always uses walking stick outside<br>Uses stair lift at home

For peer review only

Advised by doctor not to use stick because of shoulder problems
[redacted]

For peer review only

For peer review only

Feedback from others
No information available
Other people noticed participant's limp while walking.<br>Family keen on considering other treatment options
Spouse noted improvments in getting out of chair<br>Other group participants noted improvements in posture and movement.

For peer review only

Other people (spouse and friends) allowed participant to be independant again.<br>Others perceive participant activeand more physically able for own age.
Positive feedback from son and acquaintances on imprvoed posture and walking<br>People who havent seen for a long time noticed a deterioration in walking<br>People think participant is younger for own age
Positive feedback from daughters &<br>BOOST research clinician on improved walking speed
No information available

For peer review only

T2: Participant's spouse noted improvments in anxiety, stress and restlessness at night<br>Spouse encourages participant to be physically active.<br>T3: spouse noticed improvements in walking ;<br>Spouse encourages participant to be physically active.<br>BOOST research clinician mentioned improvements by 1/3rd since baseline
Participant does not like to use arm sticks in public.<br>At T3, spouse annoyed as participant repeatedly at a higher risk of fall when walking due to not using the most appropriate mobility aid.<br>This has led to a behaviour change, with Participant using arm sticks more often when going out.
T2: Participant's spouse noted improvement in overall well being<br>Support from friends and family helped participant cope emotionally<br>T2 & T3: spouse proactive keeping participant engaged in activities and outings
Participant's family has not really described any improvements.
No information available

For peer review only

T2-Everybody says participant is back to old self now<br>Positive feedback regarding negotiating stairs<br>Positive feedback that participant is walking and back to normal gardening,<br>looking better<br>T3-Participant's daughter, son and church members noted improvements in walking
At T2, spouse thinks he wont be able to manage in airports.<br>At T3, Participant's spouse is thinking<br>is walking is getting worse.
T2- Every body tells participant looks well<br>T3,- Participant's spouse thinks that participant's walking was getting better
No information available
BOOST assesors told he considerably<br>improved in 6-min walk test<br>Participant also noted improvements

For peer review only

No information available
At T3, participants family noticed improvements in back condition
No information available

For peer review only

A physio mentioned that participant's back muscles are getting stronger<br>People tell that participant is much younger and more active for actual age
At T3, participant's spouse noticed improvements in walking
No information available
No information available
No information available

For peer review only

At T1, Participant's spouse noticed that participant is getting depressed<br>At T2, Participant's spouse noted improvements in participant's activities and his mood.<br>At T3, BOOST research clinician noted improvements in 6-minute walk test
No information available.
Participant's spouse thinks that participant has learnt to cope with the condition better than before.
Participant's son suggested not to use pedal exerciser

For peer review only

No information available.

For peer review only

For peer review only

Views on treatment/Trial

Not sure if symptom improvement is due to release of trapped nerve or BOOST exercises
Gently doing exercises would have helped pain
BPA no different to previous physiotherapy participant had
Not 100% confident whether exercises helped or not (prev exp)
Wanted more information but found BPA insufficient in terms of number of sessions, content and followup.
Not 100% sure if improvements were due to BOOST or not
Sees no potential for further improvements from Physio and would try other treatments-massage & chiropractic
BOOST contributed to 20% in improvements
BOOST made participant psychologically better, than physical improvements
BOOST helped participant to selfmanage instead of opting for surgery

Less optimistic of BOOST exercises, thinks surgery is final option
BOOST exercises were appropriate, but ineffective for pain
Exercises are bettter than nothing and good for physical health
BOOST has changed behaviour (increase in exercise level)
Pessimistic on prognosis- would worsen
BOOST would have been costly
BOOST gave quicker access to treatment

Previous physiotherapy experience means: Hesistant doing anything that would cause pain & not expecting anything from BOOST
Loved BOOST sessions.
Throughly enjoyed the study
Found educational part useful.
Around T2, unsure if back pain has actually improved or has been overshadowed by neck pain
BOOST didn't change anything drastically in terms of improvements
Felt physical improvements did not lead to meaningful functional improvements
BOOST exercises were difficult to begin with-weight exercises were painful, took 2 sessions to get used to
BOOST exercises improved muscle tone
Attributes to BOOST info: Physical activity is not related to damage; falling to paralysis
Uses coping strategies learned from BOOST to manage flare-ups
Not expecting much from BOOST because of previous physiotherapy

BOOST trial as a potential treatment option
Group sessions were relaxing,
Nice feeling - small groups were like 1-to-1
Anticipated initial discomforts with exercise & muscles becoming flexible over time
Participant feels no improvement in sleeping
Enjoyed using weights -Favourite part
New insights on exercise to maintain physical condition
New insights on integrating exercises to activities of daily living
Peer interaction was valuable -'I wasnt the only one'
Shared experiences with group - reg. uselessness, depressed mood, feelings etc.
BOOST Physiotherapists were patient and encouraging
Would recommend BOOST to others
BOOST programme helped a lot
BOOST exercises easy to adhere to as they were transferable to the home environment, without the need for

Participant liked the structure of sessions
Found educational content informative
BOOST exercises were more effective than walking alone, as they target specific muscles for functional improvement
Experienced Delayed Onset Muscle Soreness (DOMS) often upto 48 hours
BOOST exercises improved muscle tone
Participant links improved leg strength to improved walking and posture
Participant links improved well being to improved walking and posture
Participant links improved mobility to exercise and improved posture
Expected exercises to be difficult to begin with and then easier
Group peer engagement improved understanding of NC
Learnt to pace BOOST exercises
BOOST gave advice on body positioning - to relieve pain
Peer discussion gave ideas for symptom management
Pleased with BOOST and would recommend to others

Pleased with BPA sessions
Would like to have more sessions
Exercises were appropriate
BOOST info booklet was aide memoire
Flexion exercises are effective in reducing pain; Floor exercises were not helpful
Confident that exercises are helpful, but unsure if it would be a permanent solution to NC

Participant attributes mobility gains to her persevering with exercise
BOOST appointments were not helpful over what participant was doing before
Felt BOOST booklet provided limited information on NC; If not strong willed, BPA is waste of time.
Trial was limited in its scope, did not provide enough opportunities to exercise.
Attributes improvements partly to BOOST exercise and natural healing process.
Found interviews helpful.

For peer review only

Participant sees BOOST trial as a beginning of long term lifestyle change.<br>BOOST trial had an impact on biopsychosocial aspects of the back condition<br>Felt that coping strategies to tackle anxiety and depression was not addressed well<br>Questioned the lack of feedback and suggested a follow up appointment would be helpful<br>Valued self-management instructions given by BOOST research physiotherapist<br>Views BOOST research appointments as physiotherapy consultancy sessions<br>Considered feedback from research clinician beneficial for overall improvement than exercises from BOOST physiotherapist<br>Attributes improvements to BOOST exercise and treatment
Generally positive, but has concerns on effectiveness.<br>T2, Not sure if it has helped the symptoms<br>Parking issues to be considered in trial design
Participant views BOOST as first targeted treatment<br>Hopeful that BOOST would maintain mobility<br>Found group interaction and peer support effective > exercise<br>Participant was upset about not receiving MRI<br>Thought BOOST would address other MSK issues (knee and neck pain)
Participant enjoyed 'everything' of BOOST.<br>Glad to be part of BOOST<br>Would like more BOOST sessions<br>Recommends BOOST to others
Participant's main concern is to be reassured tha exercise wont cause damage<br>Suggests partners should also be part of trials - interviews<br>Considers BOOST exercises were good enough without causing any fear<br>BPA sessions gave opportunities to talk about the fears participant had<br>Participant didnt get the incentive of having feedback from BPA sessions<br>Main issue: To rely on self-motivation<br>At T2, attributes physical & mental improvements 100% to BOOST<br>BOOST changed participant's mental outlook and behaviour<br>Enjoyed being part of BOOST

Exercises were helpful and very good<br>Education content was interesting and gave opportunities to clarify<br>Information booklet was useful and a motivator to exercise<br>BOOST programme has given participant will to live, the boost to live<br>BOOST sessions were motivating
Participant disappointed with BPA<br>Feels Physiotherapy is a disappointment because they tell you what to do but they don't do it with you.<br>Participant says that BOOST would have benefitted<br>Thought BPA would have been effective if physiotherapist spent more time showing the exercises
Felt 3 BPA sessions were enough.<br>BOOST booklet was informative.
Felt discussions helpful<br>Finds exercises in isolation as boring<br>The key message from discussions was "keeping up the regularity of activity" and "there is no escape from that";<br>and think of other activities (exercise type things)<br>that participant would enjoy more than the exercises.
BOOST education on exercises motivates participant to keep up with exercise<br>BOOST programme was very professional<br>Key messages were importance of being mobile and exercising<br>BOOST allowed conversations with other members<br>BOOST folder is aide memoire<br>Self explanatory with pictures and text<br>Education on slacked muscles to cause pain; toned muscles to alleviate pain<br>BOOST introduced participant to another level of activity that participant wouldnt have done without the trial

For peer review only

Participant priortises getting exercises to ease the pain than getting more information

Main reason to join was to get MRI done and get Physiotherapy or surgery as needed

BOOST did not give any new info than what was received from the osteopath

Exercises werent doing any good.

Resisted knee strengthening is painful to do.

Participant didnt find BOOST walking sessions helpful, as participant cant walk that long

BOOST exercises affected participant's hips

Knee strenghening exercises were causing soreness

Flexibility exercises were relieving the leg aches before the surgery. Participant didnt get such pain after surgery

Booklet information and education sessions were useful.

Felt his osteopath gave more information than BOOST booklet

Participant feels BOOST trial didnt help because it couldnt as it was treating a completely different thing

Glad to have got the MRI scan done

Participant enjoyed doing BOOST sessions, meeting people and doing activities with them

Exercises were relevant for the condition

Felt exercises improved the posture and relieved the back compression

Felt exercises improved the strength and balance

Felt exercises relieved the pain and stiffness; helped the walking

BOOST sessions helpedparticipant's improve the back condition

'Liked' listening to others

Good to have BOOST programme for older people

Felt BOOST programme gave the ability to plan life

Was motivated seeing others doing more

Participant not keen on surgery and hence decided to participate in trial

Wants to find more information about condition

No concerns about study

Very pleased with group sessions

Therapists were helpful and supportive

Sessions put participant in another level of activity

Nice to meet other patients with same condition

Found group sessions helpful- particularly on medication very helpful

Group sessions reminded the importance of doing exercises

Felt sessions were repetitive and thought she could have done some at home

Gave good info on medication and importance of exercise

All exercises were easy to do;

BOOST exercises helped participant's body from stiffening up

Thinks that BOOST exercises partially helped the strength; Not a major difference

BOOSTexercises made participant feel better 'mentally' than physically

Would continue the exercises as it would be horrendous if stopped

Managed traveling to BOOST sessions by bus

Participant feels mentally better because of doing the trial and exercises

Glad to be part of trial

For peer review only

Participant joined the trial to improve mobility levels<br>Has no concerns<br>Number of BPA sessions were not enough<br>Expected more from study<br>Considered BOOST visits as study visits focused more on paper work than patient interest<br>A non-study appointment would have been more focussed<br>Felt the need for recognition of problems that came from BOOST exercises (tennis elbow)<br>Didnt receive any Balance exercises from BOOST physio
Doesnt expect major changes in the condition, except able to walk dog more and do some gardening<br>Doesnt like having MRI<br>Attributes pain relief to BOOST exercises, sessions and home exercises<br>Group discussion, circuit exercises, walking were fine<br>Found the whole BOOST programme helpful, than a single specific exercise<br>Exercises were more helpful than discussion part<br>Discussion was to improve general knowledge<br>BOOST booklet as reminder for the order, reps of exercises<br>Would recommend BOOST to others<br>The BOOST Physiotherapy sessions were ‘great’.
Thinks BOOST exercises were beneficial<br>'Quite satisfied' with BPA<br>Received adequate info from BOOST<br>Feels more PT sessions wouldnt make any difference
Had initial improvements that later ceased<br>Participant felt that BPA didnt help the sciatic problem, because exercise aggravated the pain (forward bending)<br>Perceives no benefit from BOOST exercises<br>Feels BOOST exercises havent really done anything<br>Upset that BPA didnt work<br>Agrees that cost may be a barrier to go to exercise classes or hospital appointments.
Participant thinks BOOST exercises loosened the body<br>Felt flexion exercise eased the pain slightly;<br>Balance exercise were good for balance; Hip exercise for strength<br>Felt BOOST exercise didnt help heaviness in legs<br>BOOST gave her more food for thought about walking or exercising in pain;<br>BOOST helped participant get on mentally<br>Found BOOST fine and easy<br>Didnt know choosing best answer for some questions in questionnaires<br>Pleased with BOOST study

For peer review

2 reasons to participate: Personal benefit, Help others
BOOST gave little bit more than the physiotherapy that participant had before
BOOST physiotherapists were good, patient and understanding
Understood about NC symptoms from BOOST study
Participant had to accept living with NC
BOOST discussions focused on pain, dealing with pain, exercises to relieve pain
BOOST sessions gave opportunities for conversations & discuss ideas and thoughts
Learnt to break longer tasks to manageable chunks
Information on releiving pain (leaning on kitchen) increased the confidence
Appreciates help received from BOOST
Good and enthusiastic BOOST physios made participant stay with the programme
BOOST physios' attitude helped participant improve walking
BOOST exercises increased participant's confidence
Exercises were good to begin with, found some with weights difficult to do
Would recommend BOOST to others

1-to-1 BPA sessions were win-win situation for the participant
Considers exercises advice as brilliant
Felt BOOST exercises very helpful and god-send
Exercises improved participant's general attitude & made a big difference to participant's back and life
Has a sense of pride being part of trial
With the trial, felt walking as more achievable

Participant felt BOOST exercises worked well in terms of movement & strength
Got supportive information
BPA was not too bad
Would recommend BOOST to others
Was happy with the way participant was treated
BPA was tailored to participant needs.

Participant has 'no faith' in physiotherapy
Doesnt expect physiotherapy to work & when in pain,
Physiotherapy would aggravate the condition
Finds acupuncture to be effective
Joined BOOST to help self and others
Hopeful that BOOST would help others
At T2, 'totally disappointed' with BPA
Participant feels hasnt received anything from BOOST
Not called for followup sessions
Felt BOOST was not for the leg problem
Participant feels BOOST gave nothing and vice versa

For peer review only

Participant hoping to achieve 'something' from BOOST treatments, as long as it doesnt cause any discomfort.
Not expecting a pain free body by participating. Would wait to see how things get started and takes place
Felt BOOST was something that was always done.
Felt BOOST programme would not help the back condition get better but would help keep going.
Participant felt BOOST programme was excellent.
Participant found BOOST ‘very helpful’ and interesting, with home exercises to do.
There were things/activities that participant has never tried before and felt benefitted taking part in the trial

For peer review only

For peer review only

Were desired outcomes met?
NO.<br>No considerable pain relief
NO.<br>Reduced activity and participation.
NO.<br>No reduction in pain.

For peer review only

NO.<br>Still had issues with sleeping due to pain.<br>Sleeping did not improve at all.
YES.<br>Substantial reduction in pain (75%)
YES.<br>Reduced pain & Improved walking
YES<br>Able to walk more

For peer review only

YES<br>Slept better
NO<br>Pain did not reduce;<br>Mobility did not increase
NO<br>Walking did not improve
YES<br>Pain relief achieved
YES<br>Reassurance received<br>(exercise x harm)

For peer review only

YES<br>Walking improved
NO<br>Symptoms did not reduce;<br>Slight improvement in Mobility
NO<br>Didn't get back to Squash
NO<br>Leg strength not improved
YES<br>Pain relief achieved

For peer review only

Yes
Participant was glad that MRI was done

YES
Independance improved

NO
Mobility & symptoms did not improve

For peer review only

YES<br>Progressive improvements in symptoms<br>Able to walk uphills shops with less exhaustion than before
YES<br>Reduced pain & improved walking
NO<br>Pain did not reduce
NO<br>Pain did not reduce<br>Sleep did not improve
NO<br>Symptoms did not reduce.

For peer review only

YES<br>Walking improved; Resumed biking
YES<br>Able to move in out<br>covering 7000 steps (3.5 miles)
YES<br>Pain relief achieved
NO<br>Muscle wasting progressed

For peer review only

NO
Pain relief not acheived

For peer review only

For peer review only

**Description of Turning points**
External feeding tube for throat constriction at T3<br>At T2 due to symptom improvement, no longer considers surgery for pain management
Intensified pain after L4-5 decompression laminectomy at T3<br>Disappointed with outcome of surgery
Diagnosis of Cervical stenosis around T2, Main health concern at T2 & T3<br>Around T3, living circumstances impacted by financial difficulty with home heating

For peer review only

100% improvement in ability to negotiate stairs at T3 (from crawling at T1)
50-60% improvement in mobility around T2.<br>75% reduction in pain around T3.
None noted.
None noted.

For peer review only

Major emergency surgery for abdominal condiiton bet T2-T3<br>At T3, Abdominal condition is major concern for participant	
Two falls since T1 interview	
At T3, Multisite pain, es. Knee pain has primary impact on general health	
None noted.	
Just after Interview 2, had successful spinal fusion surgery for NC<br>Had Cauda equina that left participant with double incontinence<br>Moved to compact bungalow, due to mobility issues with stairs previously<br>Participant has positive outlook & in good spirits	

For peer review only

Major improvement T2: Able to walk to shops and daughters place since 4 years<br>Between T2 & T3: L4 L5 decompression surgery	
None noted.	
None noted.	
From ocassional use of walking stick to continuous usage becauuse of leg weakness at T3	
None noted.	

For peer review only

Had an emergency back surgery for Cauda Equina Syndrome few weeks before T3.<br>Since leaving hospital, had shortness of breath	
None noted.	
None noted.	

For peer review only

None noted.	
None noted.	
Around T3, Participant was waiting for hip surgery.	
None noted.	
Looking forward for knee surgery after T3	

For peer review only

None noted.	
Around T2, Participant feels physically better-Breathing, Stamina and Activity wise<br>Around T3, Ups and downs health wise caused by Addison's disease,<br>post-viral vertigo and frequent chest infections<br>(all unconnected to the back problem)	
Around T1, Back problem has a lot of effect on Quality of life, ability to do things & relation with spouse. NC pain 10/10	
At T2, participant thinks COPD interacts with leg condition making exercising difficult	

For peer review only

None noted.	

For peer review only

For peer review only

For peer review only

For peer review only

For peer review only

For peer review only

For peer review only

For peer review only

For peer review only

[redacted]	[redacted]

For peer review only

For peer review only

For peer review only

For peer review only

For peer review only

For peer review only

For peer review only

For peer review only

For peer review only

For peer review only

For peer review only

For peer review only

For peer review only

For peer review only

For peer review only

For peer review only

For peer review only

For peer review only

For peer review only

For peer review only

For peer review only

For peer review only

For peer review only

For peer review only

For peer review only

For peer review only

For peer review only

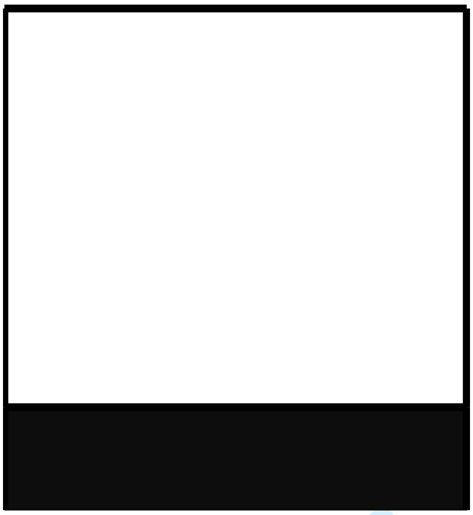

For peer review only

Supplementary file 4: Patterns of change in biopsychosocial domains at T1, T2, and T3 (ordered by treatment arm and participant study code)

			Pain			Mobility and ADL			Psychological Impact			Social and recreational participation		
**Participant code**	**Ethnicity, Gender, and Age**	**Treatment arm**	**Baseline**	**6 months**	**12 months**	**Baseline**	**6 months**	**12 months**	**Baseline**	**6 months**	**12 months**	**Baseline**	**6 months**	**12 months**
BOQ01	Black British female, 81 years	BOOST												
BOQ04	White British male, 65 years	BOOST												
BOQ05	White British male, 72 years	BOOST												
BOQ06	Black British male, 70 years	BOOST												
BOQ09	White British male, 79 years	BOOST												
BOQ11	White British male, 72 years	BOOST												
BOQ14	White British female, 77 years	BOOST												
BOQ24	White British female, 78 years	BOOST												
BOQ25	White British female, 88 years	BOOST												
BOQ26	Indian female, 72 years	BOOST												
BOQ28	White other female, 76 years	BOOST												
BOQ30	White British male, 80 years	BOOST												
BOQ39	White British female, 76 years	BOOST												
BOQ41	White British female, 71 years	BOOST												
BOQ43	White British male, 68 years	BOOST												
BOQ57	White British female, 76 years	BOOST												
BOQ02	Indian male, 70 years	BPA												
BOQ03	White British female, 79 years	BPA												
BOQ07	White British female, 72 years	BPA												
BOQ08	White British male, 70 years	BPA												
BOQ15	White British male, 65 years	BPA												
BOQ31	White British female, 81 years	BPA												
BOQ32	White British female, 77 years	BPA												
BOQ33	White British male, 72 years	BPA												
BOQ34	White British female, 78 years	BPA												
BOQ37	White British male, 88 years	BPA												
BOQ38	White British male, 76 years	BPA												
BOQ47	White British Female, 74 years	BPA												
BOQ50	White British male, 70 years	BPA												
BOQ53	White British male, 74 years	BPA												

BOOST: BOOST programme; BPA: Best Practice Advice; ADL: Activities of Daily Living

For peer review only - http://bmjopen.bmj.com/site/about/guidelines.xhtml

Legend

	When participants already had reduced pain/improved sleep/ improved mobility/ improved social and recreational participation/positive psychological impact at entry to trial or had improvements compared to a previous time point.
	Further reduction in pain, improved sleep/increase in mobility/ increase in social and recreational participation/positive psychological impact compared to a previous time point.
	Continued reduction in pain/improved sleep/increase in mobility/ increase in social and recreational participation/positive psychological impact compared to a previous time point.
	When participants already had increased pain/sleep disturbances/decreased mobility/ social and recreational participation/negative psychological impact at entry to trial or worsened status compared to a previous time point.
	Further increase in pain/sleep disturbances/decrease in mobility/decrease in social and recreational participation/negative psychological impact compared to a previous time point.
	Continued increase in pain/sleep disturbances/decrease in mobility/decrease in social and recreational participation/negative psychological impact compared to a previous time point.
	No change in pain/sleep/mobility/social and recreational participation/psychological impact compared to a previous time point.
	Lack of sufficient information from interviews.
	When pain, sleep, mobility, social and recreational participation, or psychological impact influenced by other musculoskeletal (MSK)/non-MSK issues or major health events.

For peer review only

For peer review only - http://bmjopen.bmj.com/site/about/guidelines.xhtml

Title: Longitudinal qualitative study of living with neurogenic claudication

Reporting checklist for qualitative study.

Based on the SRQR guidelines.

Instructions to authors

Complete this checklist by entering the page numbers from your manuscript where readers will find each of the items listed below.

Your article may not currently address all the items on the checklist. Please modify your text to include the missing information. If you are certain that an item does not apply, please write "n/a" and provide a short explanation.

Upload your completed checklist as an extra file when you submit to a journal.

In your methods section, say that you used the SRQRreporting guidelines, and cite them as:

O'Brien BC, Harris IB, Beckman TJ, Reed DA, Cook DA. Standards for reporting qualitative research: a synthesis of recommendations. Acad Med. 2014; 89(9):1245-1251.

		Reporting Item	Page Number
**Title**			
	#1	Concise description of the nature and topic of the study identifying the study as qualitative or indicating the approach (e.g. ethnography, grounded theory) or data collection methods (e.g. interview, focus group) is recommended	1
**Abstract**			
	#2	Summary of the key elements of the study using the abstract format of the intended publication; typically includes background, purpose, methods, results and conclusions	2
**Introduction**			
Problem formulation	#3	Description and significance of the problem / phenomenon studied: review of relevant theory and empirical work; problem statement	3, 4

For peer review only

Title: Longitudinal qualitative study of living with neurogenic claudication

Purpose or research question	#4	Purpose of the study and specific objectives or questions	4
**Methods**			
Qualitative approach and research paradigm	#5	Qualitative approach (e.g. ethnography, grounded theory, case study, phenomenolgy, narrative research) and guiding theory if appropriate; identifying the research paradigm (e.g. postpositivist, constructivist / interpretivist) is also recommended; rationale. The rationale should briefly discuss the justification for choosing that theory, approach, method or technique rather than other options available; the assumptions and limitations implicit in those choices and how those choices influence study conclusions and transferability. As appropriate the rationale for several items might be discussed together.	4
Researcher characteristics and reflexivity	#6	Researchers' characteristics that may influence the research, including personal attributes, qualifications / experience, relationship with participants, assumptions and / or presuppositions; potential or actual interaction between researchers' characteristics and the research questions, approach, methods, results and / or transferability	7
Context	#7	Setting / site and salient contextual factors; rationale	4. 5
Sampling strategy	#8	How and why research participants, documents, or events were selected; criteria for deciding when no further sampling was necessary (e.g. sampling saturation); rationale	4. 5
Ethical issues pertaining to human subjects	#9	Documentation of approval by an appropriate ethics review board and participant consent, or explanation for lack thereof; other confidentiality and data security issues	4
Data collection methods	#10	Types of data collected; details of data collection procedures including (as appropriate) start and stop dates of data collection and analysis, iterative process,	5

For peer review only

Title: Longitudinal qualitative study of living with neurogenic claudication

		triangulation of sources / methods, and modification of procedures in response to evolving study findings; rationale	
Data collection instruments and technologies	#11	Description of instruments (e.g. interview guides, questionnaires) and devices (e.g. audio recorders) used for data collection; if / how the instruments(s) changed over the course of the study	5
Units of study	#12	Number and relevant characteristics of participants, documents, or events included in the study; level of participation (could be reported in results)	Table 1<br>Pages 7, 8
Data processing	#13	Methods for processing data prior to and during analysis, including transcription, data entry, data management and security, verification of data integrity, data coding, and anonymisation / deidentification of excerpts	5
Data analysis	#14	Process by which inferences, themes, etc. were identified and developed, including the researchers involved in data analysis; usually references a specific paradigm or approach; rationale	Pages 5-7
Techniques to enhance trustworthiness	#15	Techniques to enhance trustworthiness and credibility of data analysis (e.g. member checking, audit trail, triangulation); rationale	7
**Results/findings**			
Syntheses and interpretation	#16	Main findings (e.g. interpretations, inferences, and themes); might include development of a theory or model, or integration with prior research or theory	Pages 7-20
Links to empirical data	#17	Evidence (e.g. quotes, field notes, text excerpts, photographs) to substantiate analytic findings	Pages 7-20<br>Tables 3, 4, 5<br>Supplementary files 2, 3
**Discussion**			
Intergration with prior	#18	Short summary of main findings; explanation of how	Pages 20-23

For peer review only

Title: Longitudinal qualitative study of living with neurogenic claudication

work, implications, transferability and contribution(s) to the field		findings and conclusions connect to, support, elaborate on, or challenge conclusions of earlier scholarship; discussion of scope of application / generalizability; identification of unique contributions(s) to scholarship in a discipline or field	
Limitations	#19	Trustworthiness and limitations of findings	22-23
**Other**			
Conflicts of interest	#20	Potential sources of influence of perceived influence on study conduct and conclusions; how these were managed	26
Funding	#21	Sources of funding and other support; role of funders in data collection, interpretation and reporting	27

None The SRQR checklist is distributed with permission of Wolters Kluwer © 2014 by the Association of American Medical Colleges. This checklist can be completed online using https://www.goodreports.org/, a tool made by the EQUATOR Network in collaboration with Penelope.ai

For peer review only

BMJ Open

Longitudinal qualitative study of living with neurogenic claudication

Journal:	*BMJ Open*
Manuscript ID	bmjopen-2021-060128.R2
Article Type:	Original research
Date Submitted by the Author:	29-Jul-2022
Complete List of Authors:	Griffiths, Frances; University of Warwick, Warwick Medical School<br>Srikesavan, Cynthia; University of Oxford, Nuffield Department of Orthopaedics, Rheumatology, and Musculoskeletal Sciences<br>Ward, Lesley; Northumbria University, Department of Sport, Exercise & Rehabilitation<br>Boniface, Graham; University of Oxford, Nuffield Department of Orthopaedics, Rheumatology, and Musculoskeletal Sciences<br>Williamson, Esther; University of Oxford, Nuffield Department of Orthopaedics, Rheumatology and Musculoskeletal Sciences<br>Lamb, Sarah; University of Exeter, College of Medicine and Health; University of Oxford, Nuffield Department of Orthopaedics, Rheumatology and Musculoskeletal Sciences
**Primary Subject Heading**:	Rehabilitation medicine
Secondary Subject Heading:	Rehabilitation medicine
Keywords:	Back pain < ORTHOPAEDIC & TRAUMA SURGERY, Spine < ORTHOPAEDIC & TRAUMA SURGERY, QUALITATIVE RESEARCH, Rehabilitation medicine < INTERNAL MEDICINE

SCHOLARONE™
Manuscripts

I, the Submitting Author has the right to grant and does grant on behalf of all authors of the Work (as defined in the below author licence), an exclusive licence and/or a non-exclusive licence for contributions from authors who are: i) UK Crown employees; ii) where BMJ has agreed a CC-BY licence shall apply, and/or iii) in accordance with the terms applicable for US Federal Government officers or employees acting as part of their official duties; on a worldwide, perpetual, irrevocable, royalty-free basis to BMJ Publishing Group Ltd ("BMJ") its licensees and where the relevant Journal is co-owned by BMJ to the co-owners of the Journal, to publish the Work in this journal and any other BMJ products and to exploit all rights, as set out in our licence.

The Submitting Author accepts and understands that any supply made under these terms is made by BMJ to the Submitting Author unless you are acting as an employee on behalf of your employer or a postgraduate student of an affiliated institution which is paying any applicable article publishing charge ("APC") for Open Access articles. Where the Submitting Author wishes to make the Work available on an Open Access basis (and intends to pay the relevant APC), the terms of reuse of such Open Access shall be governed by a Creative Commons licence – details of these licences and which Creative Commons licence will apply to this Work are set out in our licence referred to above.

Other than as permitted in any relevant BMJ Author's Self Archiving Policies, I confirm this Work has not been accepted for publication elsewhere, is not being considered for publication elsewhere and does not duplicate material already published. I confirm all authors consent to publication of this Work and authorise the granting of this licence.

For peer review only

Title page

Title of the article
Longitudinal qualitative study of living with neurogenic claudication

Authors
[1]Frances Griffiths, [2]Cynthia Srikesavan, [3]Lesley Ward, [4]Graham Boniface, [5]Esther Williamson, [6]Sarah E Lamb

Full name, Postal address and email of the Corresponding author
Dr Esther Williamson
Nuffield Department of Orthopaedics, Rheumatology and Musculoskeletal Sciences, University of Oxford, Windmill Road, Oxford OX3 7LD, United Kingdom.

Email: esther.williamson@ndorms.ox.ac.uk

Full name, department, institution, city and country of all co-authors
[1]Warwick Medical School, University of Warwick, Coventry, United Kingdom.
[2, 4, 5, 6]Nuffield Department of Orthopaedics, Rheumatology, and Musculoskeletal Sciences, University of Oxford, Windmill road, Oxford, United Kingdom.
[3]Department of Sport, Exercise & Rehabilitation, Northumbria University, United Kingdom.
[6]College of Medicine and Health, Institute for Health Research, University of Exeter, St Luke's Campus, Heavitree Road, Exeter, United Kingdom.

Word count, excluding title page, abstract, references, figures and tables: 5389

For peer review only

Abstract

Objectives

Neurogenic claudication (NC) causes pain and reduced mobility, particularly in older people and can negatively affect mental and social well-being, so limiting successful aging. This qualitative study explored how people with NC change over 12 months.

Design

A longitudinal qualitative study using semi-structured interviews.

Setting

Participants were recruited from a UK clinical trial of a physiotherapy intervention for NC.

Participants

Interviews were undertaken at baseline, one month after receiving any intervention and at 12 months. We analysed 30 sets of three interviews.

Results

Interview data was summarised for each time point into biopsychosocial domains: pain, mobility and activities of daily living, psychological impact and social and recreational participation. Through comparative analysis we explored participant trajectories over time.

Progressive improvement in at least one domain was experienced by 13 participants but there was variability in trajectories with early improvements that remained the same, transient changes and no change also commonly observed.

Eleven participants described co-present improvement trajectories in all domains. Three participants described co-present improvement in all domains except participation; one had never stopped their participation and two had unattainable expectations. Five participants described co-present improvement in one domain and deterioration in another and 14 participants described co-present no change in one domain and change in another.

There was evidence of interaction between domains e.g. improved mobility led to improved participation and for some participants, specific factors influenced change. Of the 15 participants who experienced improved participation, ten reported improvements in all other domains and five participants did not; for two, pain did not prevent participation, one used a walking aid and two had a positive psychological outlook.

Conclusion

The daily lived experiences of older adults with NC are variable and include interaction between biopsychosocial domains. Therapist understanding of these trajectories and their interactions may help to provide personalised therapy

Keywords: Neurogenic claudication; Longitudinal; Interviews; Trajectory

For peer review only

Strengths and limitations of this study

- In-depth interviews at three time points focus on participant's current experience; comparison across time was undertaken during analysis so was not reliant on participant interpretation of past-experience.
- Analysis summarises succinctly a large qualitative data set.
- Summarising data will have resulted in loss of nuance in our analysis.
- The results present one set of trajectory category membership for participants; some participants could be categorised differently.
- Our interest in successful aging shaped our path through analysis; a different interest is likely to result in different trajectory descriptions.

For peer review only

For peer review only - http://bmjopen.bmj.com/site/about/guidelines.xhtml

Main text

Background

Neurogenic claudication (NC) is caused by narrowing of the spinal canal (lumbar canal stenosis) most often due to osteoarthritis of the spine or spondylosis. It is frequently reported in adults aged above 60 years (1, 2). NC usually presents as pain and discomfort radiating from the back into the buttocks and legs in a unilateral or bilateral pattern. It is provoked by walking or prolonged standing and relieved by sitting or lumbar flexion (3). The pain often occurs intermittently. Other signs and symptoms may include weakness, altered sensations such as pins and needles and numbness, fatigue and gait changes (3). Pain in the lower back is usual but not a necessary diagnostic feature but regardless NC is considered a low back pain condition. NC is associated with decreased mobility and independence for older people, restriction of social activities, a negative emotional impact, and frailty (4, 5). The impact of back and leg pain consistent with NC on health status is more marked than for people with other patterns of back and leg pain; for older adults, compared to those who report back pain only or have leg pain which is not consistent with NC, those with NC report lower quality of life and are more likely to be frail, be less confident to walk ½ a mile and to report a decline in their mobility over the last year (1). The variation in symptom trajectory for people with NC is illustrated by a study of 146 people with NC followed up for 3 years (without surgical treatment), symptoms improved for about one third of patients, remained unchanged for half the patients and worsened in a few (6).

Ageing successfully is considered a key mechanism by which ageing populations can maximise their health and longevity and minimise their need to access health care systems (7). The concept of successful aging considers physical and mental health along with social and recreational engagement (7) and since its conception has been expanded to include living with multi-morbidities and chronic conditions such as NC (8-10). Our concern in this study is whether the symptoms of NC may lead to older people being unable to age successfully. We were particularly concerned to understand whether and how NC impacted on social and recreational participation and how this changed over time.

<u>Qualitative evidence on living with back pain</u>

The interaction physical and mental health and social and recreational engagement has been explored in qualitative studies of people living with low back pain and synthesised in a meta-ethnography (11). The interrelated themes identified were: impact of pain on self, use of coping strategies and impact of pain on engagement with others. The meta-ethnography concluded that chronic low back pain is complex, dynamic and multidimensional with those living with the pain experiencing distressing pain and loss with lowered self-worth, stigma, depression, premature aging, and fear of the future. A later cross-sectional qualitative study of people with NC, suggests that pain, physical ability and emotions are inter-related and at times inseparable (5). The meta-ethnography identified a lack of longitudinal studies. A subsequent longitudinal study interviewed eight people living with back pain annually over two years (12). Although pain and loss impacted on all areas of the participants lives, two different patterns of change over time were identified: participants who saw their pain a physical problem and a cause of loss and did not re-establish social and recreational activities, and those who experienced pain relief and were less enmeshed in the pain experience so able to look to the future (12).

For peer review only - http://bmjopen.bmj.com/site/about/guidelines.xhtml

Such longitudinal qualitative studies provide a depth of understanding to complement existing panel studies (13) and build on the idea of illness trajectory (14), biographical disruption (15) and understanding individuals as unique, complex and adaptive (16-18). Our study aims to provide this depth of understanding of people living with NC: how their NC impacts on their function and engagement and how this changes over time. It builds further on longitudinal qualitative studies in the health domain (19).

The BOOST trial

This qualitative study was embedded within the Better Outcomes for Older people with Spinal Trouble (BOOST) trial (20, 21), a multi-centre, randomised controlled trial with two treatment arms. It compared a 12-week group-based physical and psychological intervention (the BOOST programme) to individual best practice advice (BPA) for older adults (65+ years) with NC. The BOOST programme comprised group discussions that were underpinned by cognitive behavioural techniques, an individually tailored exercise and walking circuit, and home exercises. BPA comprised home exercises and self-management (1-3 appointments with a physiotherapist). Participants aged 65 years or over with the clinical presentation of NC symptoms were recruited from primary care practices of the National Health Service (NHS) in the United Kingdom (UK). People living in residential care or nursing homes, those registered blind and those with a terminal health condition or on a waiting list for surgery or unable to walk 3m without the support of another person or unable to provide informed signed consent were not included. Participants were randomised in a 2:1 ratio (BOOST programme: BPA). Full trial protocol details are reported elsewhere (21).

Methods

Study design

We used a longitudinal qualitative research design to understand the individual at the time of each interview (22) rather than seeking from the individuals their own narrative of change (23). We aimed to interview participants three times: at trial baseline (after randomisation but before the participant knew their treatment allocation) (T1); one month after any intervention (T2) and 12 months post-randomisation (T3). TI interviews were via face-to-face at participant's home, with T2 and T3 by telephone. Where participants had difficulty using the telephone, T2 and T3 interviews were undertaken face-to-face.

Ethics approval

Ethics approval was given by the London-Brent National Research Ethics Committee (REC number 16/LO/0349) on 03 March 2016.

Participants and Recruitment

All participants recruited to the BOOST trial (20, 21) were eligible for the interview study and consent to invite participants for interview was collected as part of trial procedures. We invited participants consecutively from each recruitment site over the 25-month trial recruitment period (August 2016 to August 2018), stratifying to ensure balance between range of age, gender, ethnicity, treatment allocation (ratio 1:1) and recruitment site location. To ensure sufficient information power (24), we aimed for 30 participants retained at 12 months and so aimed to recruit approx. 60 participants to allow for 50% attrition.

For peer review only - http://bmjopen.bmj.com/site/about/guidelines.xhtml

Data collection

We obtained written consent at the start of the first interview and reaffirmed this verbally at follow-up interviews. We used semi-structured interview schedules (**Supplementary file 1**). We used the present tense when asking questions to encourage participants to focus on their current state (22). In T1 interviews, we explored the impact of NC on participant physical and psychosocial health including NC and their beliefs around exercise and ageing. In T2 and T3 interviews we explored similar issues along with their experiences of the BOOST trial interventions. Before T2 and T3 interviews we reviewed for each participant the data captured in their earlier interviews and made notes about the participant and their context. We used this knowledge in the interviews to rapidly establish rapport on the telephone and enable quick clarifications. For example:

> Participant: I go to my club.
>
> Interviewer: Is that the ladies club you mentioned last time we met?

All interviews were audio-recorded onto encrypted recorders and stored securely at University of Oxford. During transcription/summarising all personal identifiers were removed.

Patient and public involvement (PPI)

A group of patient representatives were involved in developing the application for the BOOST study and one member was co-applicant. PPI representatives reviewed patient materials such as consent forms, participant information sheet and the interview schedules. We carried out some practice interviews with PPIs to test and refine the interview schedules prior to data collection. The interview findings were presented to the PPI representatives in a study meeting and were reviewed by the lead PPI representative.

Data analysis

Throughout our analysis we compared data across time and between participants and drew on existing evidence-based concepts and definitions related to back pain and NC. We mapped the data into a framework matrix (25) then explored the matrix (26, 27) for change processes related to NC. This involved summarising patterns of change being experienced at each time point, trajectories of change between time points (27) co-presence of trajectories, interactions between trajectories and turning points. In our analysis of data at each time point we used an analysis approach we had previously developed (18) and applied (22). We focused on current experience and put to one side data where the participant was interpreting past-experience. This allowed us to compare what was current at each time point and to manage the volume of complex data (28). We describe each analysis step below. Analysis was conducted in Excel.

Step 1: Development of individual participant frameworks

We started analysis on complete interview sets (T1, T2 and T3). We selected sets where the participant provided rich data about the experience of NC. After selecting 30 sets (90 interviews) and undertaking initial analysis (see Step 2 below) we read and discussed our remaining data to check whether it was revealing new themes; we determined we had achieved data saturation(29). All T1 interviews were transcribed verbatim along with the first 10 T2 and 10 T3 interviews we analysed. The remaining interviews were analysed directly from audio recordings. To immerse ourselves in this large volume of data we initially wrote structured pen portraits(30) at each time point for ten data sets. This involved reading and

For peer review only - http://bmjopen.bmj.com/site/about/guidelines.xhtml

rereading the data, clarifying our focus on current experience related to NC, summarising participant experiences and context with illustrative quotes, and developing a structure that worked across the 10 data sets. From these we developed and refined a coding template. We used this to thematically code all thirty interview sets, writing mini statements with illustrative quotes for each relevant code/sub code at each time point. The resulting 30 individual participant frameworks were used for subsequent analysis.

Step 2: Development of change framework

After team discussions, we summarised the coded data for each participant at each time point into the following biopsychosocial domains: pain, mobility and activities of daily living (ADL), psychological impact, social and recreational participation and sleep. For example, to summarise mobility status at T1, we collated and summarised data from codes: current mobility, activities reduced, activities stopped, activities increased, and sub-codes such as physical impact. Participants reported a range of symptoms including numbness, tingling and heaviness. However, the symptom that consistently predominated across all participants' accounts of their experience of NC was pain; we therefore summarised the experience of pain in the framework. We included data that conveyed the pattern of change each participant was experiencing at the time of each interview. For example, a gradual improvement or worsening as experienced day-to-day/week-to-week. We added to the change framework participant age, gender, ethnicity, living arrangement, group allocation in the trial, health events and co-morbid conditions. We summarised participant coping strategies, views on aging, use of mobility aids and feedback from others.

Step 3: Identification and categorisation of participant trajectories

To identify participant trajectories for each biopsychosocial domain, we compared the summaries in the change framework for what increased or emerged over time, what decreased or ceased over time and what remained consistent over time (26). We added a description of this trajectory to the change framework. In a new table we presented for each domain the colour coded trajectory for each participant. Each team member then independently, by eye, ordered the participants so those with the most similar combination of trajectories were adjacent. The team then compared their resulting tables, discussed similarities and differences, and agreed on one version of the table. Using this table, we then independently categorised the participants into groups based on similarity of trajectories and discussed and agreed categorisation.

Step 4: Exploring the nature of the trajectories

Identifying and categorising co-presence and interaction between the trajectories of the individual biopsychosocial domains

We scrutinised the change framework for each participant and compared participants to understand which trajectories were co-present at each time point and so may have interacted with each other. For example, a reduction in pain was accompanied by improvement in mobility or deterioration in psychological outlook was accompanied by reduced social or recreational participation. We returned to participant data to check whether the participant reported interaction between the trajectories. We grouped similar types of co-presence and explored how these related to our trajectory categories. Given our interest in successful aging, we particularly explored how social and recreational engagement changed along with other domains.

Identification of health-related turning points

For peer review only - http://bmjopen.bmj.com/site/about/guidelines.xhtml

We examined the data for health-related turning points mentioned by the participant; what the participants considered significant change in their health during the study timeframe.

Totality of change

From the whole change framework we examined factors that were not inseparable from trajectory category although are likely to be interrelated (5). Factors included whether they lived alone, how they considered aging and use of mobility aids, and strategies for coping with NC in their daily lives. We identified what was similar and different between participants in the different trajectory categories.

Study team roles

SEL and EW (health service researchers and physiotherapists) led the BOOST programme of work including this study and the BOOST trial. FG (general medical practitioner, medical sociologist and qualitative researcher) led the qualitative study team that included LW (qualitative health researcher), CS (physiotherapist and a qualitative researcher) and GB (physiotherapy researcher). All contributed to qualitative study design, reading data, thematic and comparative analysis, synthesis, interpretation and reporting. LW undertook all interviews. LW and FG reviewed the interview schedules to improve the clarity and flow of the questions. CS worked with FG on analysis with GB providing independent review of coding/summaries. Any discrepancies were resolved through discussion. EW and SEL reviewed each step of the analysis and provided feedback to FG, LW, CS, and GB.

This study team were not involved in the delivery of BOOST interventions or outcome assessments in the trial. FG, LW, CS, GB remained blinded to the results of the BOOST trial until they had completed data coding and analysis.

Reporting

The Standards for Reporting Qualitative Research (SRQR) guidelines (31) were used to report the study. Quotations from interviews are labelled with participant number and interview T1, T2 or T3.

Results

The data

Participant attrition across the three time points was lower than expected, with 49 of the 60 participants (82%) providing three interviews. Reasons for withdrawal were: withdrawal from clinical trial (n=3), ill health/awaiting investigations (n=4), increased pain after receiving BOOST programme sessions (n=2), declined third interview (n=3), unable to contact (n=3) and withdrawn by researcher as participant postponed >6 months (n=2). Initial interviews averaged 70 minutes (range 44-93 minutes), with second and third interviews averaging 36 minutes and 32 minutes respectively.

Participant characteristics

There was little difference in terms of age (average 75 years), gender and ethnicity between trial, interview, and analysis cohorts and were similar in terms of measures related to NC. (**Table 1**).

For peer review only - http://bmjopen.bmj.com/site/about/guidelines.xhtml

Table 1: Description of BOOST trial participants, those interviewed and those included in the qualitative analysis presented in this paper. Values are N (%) or mean [SD] or median {IQR} as specified

Participant Characteristics	BOOST trial cohort<br>N (%)<br>Mean [SD]<br>Median {IQR}	Interview cohort<br>N (%)<br>Mean [SD]<br>Median {IQR}	Interview analysis cohort<br>N (%)<br>Mean [SD]<br>Median {IQR}
Total participants	435	59	30
Age (years at baseline)	74.9 [6.0]	75.5 [6.2]	75.1 [5.7]
Gender			
Female	246 (56.6)	31 (52.5)	14 (46.7)
Male	189 (43.4)	28 (47.5)	16 (53.3)
Ethnicity			
White British/White Other	400 (92)	52 (87)	26 (86.6)
Black British	12 (2.8)	3 (5)	2 (6.7)
Indian	9 (2.1)	3 (5)	2 (6.7)
Pakistani	6 (1.4)	1 (2)	0 (0)
Confidence walking long distance	5.6 [3.3]	5.1 [3.3]	4.7 [3.0]
Oswestry disability index	32.9 [13.9]	32.7 [13.5]	34.6 [14.5]
Tilburg frailty index	4.5 [2.6]	4.8 [2.8]	5.6 [2.8]
Exercise self-efficacy, median {IQR}	69 {53, 80}	69 {43, 86}	52. 5 {37, 81}
Fear avoidance beliefs	12.9 [5.9]	14.0 [6.3]	14.1 [6.4]
Six-minute walk test	255.4 [99.1]	249.3 [96.9]	227.7 [91.9]
Self-management rating scale	6.08 [1.80]	6.0 [1.7]	6.1 [1.7]
**Treatment allocation in the trial[1]**			
BOOST programme (BOOST)	292 (67.1)	32 (53)	16 (53.3)
Best practice advice (BPA)	143 (32.9)	27 (45)	14 (46.7)
**Complete sets of three interviews**		49	30

Abbreviations: BOOST: Better Outcomes for Older people with Spinal Trouble; SD: Standard Deviation: IQR: Interquartile Range

Scale range and direction

Confidence in ability to walk long distance (1 to 10, lower values worse)

Oswestry Disability Index V2.1a (0 to 100, higher values worse)

Tilburg Frailty Index (0 to 15, higher values worse)

Exercise self-efficacy scale (0 to 90, lower values worse)

Fear Avoidance Beliefs Questionnaire Physical Activity (0 to 24, higher values worse)

Six-minute walk test (distance covered walking for 6 minutes, lesser distance worse)

Self-management rating scale (0 to 10, lower values worse)

<u>Analysis results</u>

For peer review only - http://bmjopen.bmj.com/site/about/guidelines.xhtml

Analysis steps 1 to 3: participant trajectories

From summarising patterns of change from our individual participant frameworks (**Example available on request**) into our change framework (**Supplementary file 2**) we identified patterns of change for each participant in each domain (**Supplementary file 3**). We then summarised the trajectory across the three time points (see **Table 2)**. We omitted sleep as a domain as although a question on sleep was asked in each interview many interviewees did not provide data.

Table 2: Participant trajectories in the biopsychosocial domains

For peer review only

For peer review only - http://bmjopen.bmj.com/site/about/guidelines.xhtml

Table 2: Participant trajectories in the biopsychosocial domains

Categories of trajectories	Participant ID	Pain	Mobility & ADL	Psychological impact	Social and recreational participation
**Category 1 (n=10)** Participants with progressive improvement in at least two domains	08				
	47				
	30				
	57				
	50				
	25				
	24				
	07				
	01				
	05				
**Category 2 (n=5)** Participants with initial and maintained or delayed improvement in at least two domains	37				
	43				
	26				
	31				
	09				
**Category 3 (n=5)** Participants with discordant trajectories – where at least one domain trajectory was to some extent in the opposite direction to at least one other domain trajectory	38				
	15				
	02				
	04				
	41				
**Category 4 (n=3)** Participants with transient change in at least two domains	39				
	14				
	33				
**Category 5 (n=7)** Participants with no change in at least two domains	34				
	32				
	53				
	06				
	28				
	03				
	11				

Legends for trajectories

No change: No change in pain or in the decreased mobility decreased social and recreational participation or negative psychological impact experienced since before the onset of NC.

Progressive worsening: Progressive worsening of pain, decreased mobility, decreased social and recreational participation, or negative psychological impact (i.e. continued worsening after an earlier worsening).

Improvements remained same: Improvements experienced at T1 (compared to entry into trial) or at T2 (compared to T1) in pain, mobility, social and recreational participation, or psychological impact were maintained.

Progressive improvements: Progressive improvements in pain, mobility, social and recreational participation, or psychological impact (i.e. continued gain on an earlier improvement).

Transient change pattern 1: Initial improvements between T1 & T2 then reversed to T1 (Baseline) status at T3.

Transient change pattern 2: Initial improvement between T1 & T2 & then worsening between T2 & T3, to worse than T1

Transient change pattern 3: Initial worsening between T1 & T2 & then improving between T2 & T3

Delayed improvement: Pain, mobility, social and recreational participation, or psychological impact improved at T3 only.

Lack of sufficient information (at least 2 time points) to describe the change process.

For peer review only

For peer review only - http://bmjopen.bmj.com/site/about/guidelines.xhtml

Progressive improvement in at least one domain was experienced by a total of 13 participants. Trajectories of no change, early improvements that remained the same and transient changes predominated. Of the 30 participants, 16 experienced no improvement in at least one domain. Improvement in pain was experienced by 16 participants. All 15 participants who experienced improved social and recreational participation also reported improvements in other domains (although for one the improvement in another domain was after initial worsening).

The trajectories in any one domain are not an indication of the amount or nature of change so the same trajectory might be experienced very differently. For example, participant 24 and participant 50 both showed progressive improvement in mobility, but one was able to walk much further than the other:

> *And as I said, I can go out and I can go off to different parts of the shops, or garden centres, we like looking round those.* (24, T3)

> *I am able to get up and walk without any aids around it* [home] *now* (50, T3).

In the domain of psychological impact, participant 04 and 02 continued from T1 to T3 with negative but different experiences. The first experienced frustration as he had to rely on his wife to do his self-care activities:

> *I can't get myself dressed, I can't do the things that I would normally do or anybody else would normally do for themselves. I need a hand; I need help with that, which I do get obviously, with [wife]. Again, that's frustrating because she's got things to do and I don't feel I can keep saying to her, oh can you just help me with this, can you help me with that?* (04, T3)

The second describes fear of damaging his back with certain activities:

> *But I don't do gardening like I used to do before. For the fear of damaging or hurting my back, and things like that. Because I tried that in the past, and once I start doing something then I get carried away, and the next two days then I have to suffer, so I try and avoid it* (02, T3).

In the domain of social and recreational participation, participants 07 and 24 experienced progressive improvements over time but with different reach. The first was prepared to extend her activities:

> *But wherever I go, if I go on holiday as well, I never say no, that I can't do (some)thing* (07 T3)

The second was positive about her ability to undertake small tasks that helped her feel she was participating equally with others. She described social outings with her friends:

> *I can carry a tray with drinks on now. I can take my turn, I can get in the queue, where it always used to be 'you get the table, I'll go* (24, T3).

When placing participants adjacent to other participants based on the similarity of trajectory, we were making choices about which trajectories to prioritise. Different choices resulted in a different ordering of participants. For example, we could place together participants with two or three domains with the same trajectory and not worry about the trajectory of the other domain(s), or we could place together participants with the same number of trajectories of a certain type, irrespective of which domains they were. However, participants tended to

For peer review only - http://bmjopen.bmj.com/site/about/guidelines.xhtml

remain towards one end or the other of the table. Table 2 presents one way of ordering the participants on which the team could agree.

Our visual impression was there were five main patterns of trajectories which we identified as our trajectory categories, although the boundaries of these are not clear cut. They were: category 1 (n=10) participants with progressive improvements in at least two domains; category 2 (n=5) paticipants with initial and maintained or delayed improvements in at least two domains; category 3 (n=5) participants with discordant trajectories – where at least one trajectory was to some extent in the opposite direction to at least one other trajectory (the opposite direction might be transient); category 4 (n=3) participants with transient change (in the same direction) in at least two domains; category 5 (n=7 participants with no change in at least two domains. We illustrate the trajectories for one participant at each time point with quotations from their data in **Table 3** and for one participant from each trajectory at T3 in **Table 4**.

For peer review only

For peer review only - http://bmjopen.bmj.com/site/about/guidelines.xhtml

Table 3: Illustrative quotes for one participant [Category 3] for all domains and time points

ID: 04	**Pain**	**Mobility and ADL**	**Psychological impact**	**Social and recreational participation**
T1	*'And then, umm, there's pain if I get up, there's pain if I walk, there's pain sitting down, there's pain standing up, there's pain reaching out… it's a constant pain. 24/7 I'm in pain'*	*'I'm inactive, I can't do anything without being in pain…'*	*' So I started to get depressed then, because I couldn't... I can't do anything and I couldn't do anything and I, I just sort of thought, 'Well what's the point, what is the point of me being here when I can't do anything?' This is it, I'm just sitting around, I'm doing nothing, I'm no good …'*	*'Oh I love to see the grandkids but, again, I can't do anything with them, I can't do nothing with the grandkids because, again, the... My grandson's into football now and I feel there's a lot I could teach him with the, with the game and I can't.'*
T2	*'Not really, it's* [Pain] *there the majority of the time.'*	*'Well every day, every day. I mean we're either out shopping or that, I don't think there's a day goes by where I'm not doing some sort of walking.'*	*'I'm in a situation where I'm still depressed over it because obviously I'm not … I didn't expect a lot from it, I suppose in my mind I thought it might be, I might be having a lot more movements and things like that, which haven't worked out.'*	*'Socialising I don't do. I'm not a socialiser anyway. All them days … since the football finished and everything I'm not, I'm not one for going out and that anyway. As I said, sometimes it's not worth the effort to get ready and. Getting washed and changed and then getting out like that, so much is involved with it, sometimes it's not worth doing it'*
T3	*'Well I'm still in a lot of pain….*	*'I feel more confident when I'm in the house. I mean while the weather's like this I won't budge out at all, I won't venture out at all, there's no point….Obviously, when it's really severe, I can't get myself dressed, I can't do the things that I would normally do or anybody else would normally do for themselves'*	*'I mean, I keep bringing the mental side of it because I think everything that goes on does affect you mentally. It's very hard not to get away from a lot of things mentally'*	*'I said, my one grandchild is playing football himself now and I can't, there's no way I can go with him and help him or be involved in his, in coaching with him or teaching him anything or just having a kick around with him. I can't do that, so I feel I'm missing out on all that.'*
**Trajectory**	No change	Initial improvements between T1 & T2 then reversed to T1 (Baseline) status at T3	No change	Progressive worsening

For peer review only - http://bmjopen.bmj.com/site/about/guidelines.xhtml

Table 4: Illustrative quotes of participants from each trajectory category at T3

Participants from trajectory categories	Pain	Mobility and ADL	Psychological impact	Social and recreational participation
**Category 1**<br>ID: 08	'But in terms of my back pain and legs, although I still have a little and last night it was particularly uncomfortable, most of the time it actually is fine, and I feel a lot better than I did last year.'	'If I go for a walk, for example, with my wife, I can probably do quite a long-ish walk, probably three to five miles, which I couldn't do 12 months ago.'	'But, for example, to give you a clue of how more confident I am, we are going away for five days on Sunday to [city] to do some sightseeing, some walking, and some photography. So I feel confident enough to be able to go and do that.'	'... we went down to [Town] for a week [Holidays], back in November and the apartment we had was about a mile and half out of the town centre. So we walked that both ways, in and out, most days, and I coped with that quite well.'
Category 2<br>ID: 31	'Oh it is better. It's under control, as much as I can get it under control, with the help of the Gabapentin, which, I know it's another drug to be on but if it works then.'	'I walk down the stairs and up the stairs, rather than use the [stair lift]. And I walk up and down the hall quite a bit, I do an exercise where I just walk up, turn around and come back, I do that about ten times. Particularly if it's a wet day and I can't get out.'	'Well, I'm walking better. Whereas before I was getting frightened to walk, because I was in so much pain.'	'Well, I tend to go in [Gardening] for a short while. Once my back starts hurting, I come back in and sit down. I don't overdo it generally.'
**Category 3**<br>ID: 04	"Well I'm still in a lot of pain, but not as much as I was last week. It's sort of calmed down a little bit now."	"Well, I don't cook for myself or anything like that. I mean, I can't mess around with things like that. Obviously, when it's really severe, I can't get myself dressed, I can't do the things that I would normally do or anybody else would normally do for themselves."	"I mean, I keep bringing the mental side of it because I think everything that goes on does affect you mentally. It's very hard not to get away from a lot of things mentally."	"Normally I miss out on a lot of things because of sitting somewhere for too long or something because this again causes discomfort. So rather than sit there feeling miserable I'd rather not go."
**Category 4**<br>ID: 14	'At the moment I think I'm at a bad stage, because I've got so many things going on ... I've got the back pain. I've got the neck pain. I've got the knee, which comes out of joint. So it's not a pretty sight really.'	'But I still make myself, when we've got a good day, not today like it is now, but if it's a good sunny day I will still try to go out and do about ten minutes, fifteen minutes' walk.'	'I was doing very well and very eager to get better or help myself somewhat ... So I've got all these things [other musculoskeletal issues] going at once. Which has dragged me down a bit, there's no doubt about it, it's made me quite depressed.'	'It's obviously; it's something I'm not used to. I'm used to going everywhere and very, sort of, doing exercises and yoga classes and everything else. And it's getting worse, because it's stopped, it's stopping, everything is going wrong.'
**Category 5**<br>ID: 11	Once I've got my legs, once I'm moving a bit, it does seem to ease off, I'm not so bad. But if I sit down, then once I get up again it's back to square one; you're still in pain and tingling.'	'So it's hard for me to put, you know, for me to actually sit still all day. In the last week, I've watched more telly than I've watched in 12 months. So you can tell, like, the situation. I've never watched so much, I've got square eyes.'	'Well at the moment I'm more anxious than ever with what I've got now. So I'm still a bit anxious, well even before this, before this I was still anxious. I was quite anxious really, you know, because I wanted to do things.'	'And so it's really, my lifestyle really, to me, it's gone to nil from what I - I'd be out doing everything sort of thing, nine o'clock, ten o'clock at night time gone by, til it got dark, pottering about, cutting the lawn, doing the lawn, or doing the neighbour's if they couldn't do it, elderly people I'd cut theirs for them. So it's, to say, like, you've come to, not nil as such, but you've really, it's a total cut off ...'

This categorisation was developed from one way of ordering the participants (see above). It was therefore expected that some participants could be in more than one category (for example participant 34 could be in category 2 or 5).

We chose to define category 3, discordant trajectories, as trajectories going in opposite directions (improving/worsening). Arguably, a no change trajectory alongside a transient change or an improving/worsening trajectory is also discordant. All but one of the 10

participants in categories 4 and 5 have this latter combination of trajectories, and four of the 15 participants in categories 1 and 2.

Of the 15 participants who experienced improvement in social and recreational participation, ten experienced improvements in all other domains and were in categories 1 and 2. The remaining five participants (24, 43, 38, 41 and 34) did not experience improvement in all other domains and were from across all categories except category 4.

Analysis step 4: nature of the trajectories

Co-presence of domain trajectories and perceived interaction

We identified ten types of co-presence of the trajectories (**Table 5**). The most common presentation was Type 1 where positive trajectories were co-present for all domains. Our data suggests improvement in one domain often led to improvement in another and so on (see illustrative quotes Table 5). Type 2 (n=3) was similar except those improvements in pain, mobility and psychological were not co-present with improvements in social and recreational participation. The reasons for this were very different and specific to each individual: participant 09 was a 79-year-old farmer who was unable to get back to his heavy farm work, participant 37 was an 88-year-old who did not get back to going on group trips which involved walking as he did not want to hold up the others and 01 was an 81 year old who at baseline had already managed to return to Tai chi and her voluntary work and maintained this. All the participants of type 1 and 2 were in trajectory groups 1 and 2 (improvement trajectories) except participant 38 who was in trajectory group 3 as they had transient decline before improvement in two domains. This participant gained mobility and social and recreational participation through learning to effectively use a walking aid.

For peer review only

For peer review only - http://bmjopen.bmj.com/site/about/guidelines.xhtml

Table 5: Co-presence of trajectories (⇑ improving; ⇓ worsening; ⇔ no change) and illustrations of how they interact (or not)

Co-present trajectories of biopsychosocial domains	Participant ID	Participants quotes illustrating interaction between co-present trajectories	Trajectory Category (see table 2)
1) ⇑ Pain, Mobility, Psychological impact, Participation	30, 31, 07,08, 25, 38, 05, 26, 57, 47, 50	*'When you first saw me, I was in a bit of pain, and everything was a struggle. But now, I take everything in my stride now ... I think at least 75% I feel better in myself* **[Pain]**… *Oh I'm quite satisfied* [with walking] *at the moment. As I say, a couple of miles is easy now, when I go out shopping or anything like that* **[Mobility & ADL]…***And then as I say, Wednesdays and Thursdays I go out to the club, have a drink, and socialise with my friends. And Saturday night I go out, get on the dance floor if I feel up to it, for half an hour or something like that* **[Participation]***…But as far as, it* [Neurogenic claudication] *doesn't stop me doing anything I want to do'* **[Psychological impact]** (30, T3)	1 and 2<br>Improving
2) ⇑ Pain, Mobility, Psychological impact ⇔ Participation	01, 09, 37	*'I really haven't had any problems with my back* (37, T2) **[Pain]***…I don't think there is any problem with my general health…I think I am walking better than I was when I started this scheme…I suppose I have really not a lot of problem, I can walk the distances that I need to now, I don't want big distance… I think it* [Walking uphill] *is easier…it has been easier before when I first met you, if I walked uphill, I felt exhausted…now I don't* **[Mobility & ADL]***…I design my life around my abilities* **[Psychological impact]***…Not a lot, to be fair* [impact of condition on lifestyle]*…I keep myself involved that is my objective to keep myself occupied…To be fair, I think I work my physical life to the limitations I have got and being my age… I don't suppose I can say it* [General lifestyle] *altered other than doing exercises in the morning'* **[Participation]** (37, T3)	1 and 2<br>Improving
3) ⇔ Pain ⇑ Mobility, Psychological impact, Participation.	43, 24	*'No, just through the night* **[Pain]***… I'm getting around fine…I just feel good* **[Mobility & ADL]**. *Makes me feel good, it gives me confidence. You see I can do it, and now I know I can do it* **[Psychological impact]***… I can carry a tray with drinks on now. I can take my turn, I can get in the queue, where it always used to be 'you get the table, I'll go'* **[Participation]** (24, T3)	1 and 2<br>Improving
4) ⇓ Pain, Mobility, Psychological impact, Participation (except 41 where improved Participation gained by remained the same)	03, 11, 33, 41, 28	*'… once I've got my legs, once I'm moving a bit, it does seem to ease off, I'm not so bad. But if I sit down, then once I get up again it's back to square one; you're still in pain and tingling* **[Pain]***…. 'I'm really basically, not doing nil, but you know, walking up and down the garden and walking, not really doing to what I normally do. It's really halved* **[Mobility & ADL]***…Well at the moment I'm more anxious than ever with what I've got now. So I'm still a bit anxious, well even before this, before this I was still anxious. I was quite anxious really, you know, because I wanted to do things* **[Psychological impact]***…… And so it's really, my lifestyle really, to me, it's gone to nil from what I - I'd be out doing everything sort of thing, nine o'clock, ten o'clock at night time gone by, till it got dark, pottering about, cutting the lawn, doing the lawn, or doing the neighbour's if they couldn't do it, elderly people I'd cut theirs for them. So it's, to say, like, you've come to, not nil as such, but you've really, it's a total cut off'* **[Participation]** (11, T3)	5<br>No change
5) ⇔ Pain ⇓ Mobility, Participation (or not mentioned). ⇑ Psychological impact	53, 06,	*'Still I am in some difficulty and problem* **[Pain]***… but….honestly I just carry on with my life* **[Psychological impact]***…. …It* **[Pain]** *is not too bad at the moment….To be honest, I don't do anything* **[Mobility & ADL]** *at all…Some days…bad days which you know you just wonder what else you can do… Housework otherwise, I take my time. I don't try to push myself to you know everything I need to suppose one time...Little at a time* **[Mobility & ADL]***…I don't worry about it I just try on and carry on with my life* **[Psychological impact]***…. I do that* [Shopping] *not alone but always have somebody with me'* **[Participation]** (06, T3)	5<br>No change
6) ⇔ Pain ⇓ Mobility ⇑ Psychological impact, Participation	34	*'Could* **[Pain]** *be better. I can feel my sciatic nerve all the time* **[Pain]***… …but it didn't really bother me, you know* **[Psychological impact]** *…. I can't walk as quick as I used to and I can't do steps…I am walking normally but the smaller steps what I used to do… My legs hurt when I start moving around…there are certain movements I just be careful with…but other than that, fine* **[Mobility & ADL]**, *I am doing my garden. My back doesn't like it very often…You know I come in, sit down, have a cup of tea and go back again. I go to my gardening club...Tuesday I see my friend as usual…put the world right and have a cup of coffee…today I had been to school….so listen to the children reading and now being given an award today* **[Participation]***……You know you just have to get on with it, don't you? I can't go anywhere, I feel shattered, but you still have to do don't you? If you got to do something, you got to do it'* **[Psychological impact]** (34, T3)	5<br>No change

For peer review only

7) ⇑ Pain ⇓ Mobility, Psychological impact ⇔ Participation	02	*'But my back pain, the constant pain and the severe pain that I used to have is no longer there at the moment, so I'm happy to say that. However, it is not completely gone, and I get days, every now and again, when the pain comes back* ***[Pain]****.... What happens is then the pain starts, not while I'm walking but after I have done my walk and I sit down. And the more I walk, the more severe the pain is when I sit down* **[Mobility & ADL]**... *'It's not the ideal situation, because it makes me little bit home bound and I cannot travel as freely as I would like to, long distance and things like that* **[Participation]**...*this summer I had high hopes of doing a lot of other things, to improve my back and things like that, but with this balloon sticking from my stomach I can't even go swimming or cycling... Cycling might be a bit too strenuous. Although I have been told that people do go swimming with this type of tube, but I don't want to take that chance'* **[Psychological impact]** (02, T3)	3<br>Discordant
8) ⇑Pain ⇓ Mobility ⇔ Participation<br>⇑ Psychological impact before T3 spinal fusion	15	*'I got no pain at all now…I am pain free in my back* **[Pain]**… …*I got a walking frame that I can with difficulty walk around the house* **[Mobility & ADL]** *I say 'with difficulty' because at times my right leg just tend to buckle slightly, because the right leg is by far the worst one…it was by far the worst affected it does tend to buckle …and so either my wife or the carer when they come, they escort me, it is they don't have to do anything, they are there as a safety precaution* **[Mobility & ADL]**.... *All in all, it's been a fairly traumatic sort of time…I keep myself in good spirits…well its happened what is the point of being miserable about it, I keep myself in good spirits and carry on'* **[Psychological impact]**...*Not doing any physical activity, obviously my body is not getting tired….Obviously at the moment, is the rest and recuperation time…'* **[Participation]** (15, T3)	3<br>Discordant
9) Pain predominantly from other MSK problem.<br>⇓ Mobility, Psychological impact, Participation (or not mentioned)	04, 14, 39	*'At the moment I think I'm at a bad stage, because I've got so many things going on ... I've got the back pain. I've got the neck pain. I've got the knee, which comes out of joint. So it's not a pretty sight really* **[Pain]**.... *I'm used to going everywhere and very, sort of, doing exercises and yoga classes and everything else. And it's getting worse, because it's stopped, it's stopping, everything is going wrong…. So I've got all these things going at once* **[Mobility & ADL and Participation]**. *Which has dragged me down a bit, there's no doubt about it, it's made me quite depressed'* **[Psychological impact]** (14, T3)	4<br>Transient change
10) Pain predominantly from other MSK problem.<br>⇓Mobility, Participation (or not mentioned) ⇑Psychological impact	32	*'Terrible* **[Pain]** *at the moment…Such a lot of pain in my knee then I have got pain in my right buttock which is more like… it is the sciatic nerve is trapped and I can't sit for longer I have to keep putting the pillow under or keep lying down…Lying down flat makes my back worse...its* [Hip pain] *been on and off a couple of years…some days its* [Back pain] *been worse, I can't get washed and dressed… Its pain everywhere…there is nothing you can do till you get repaired* **[Psychological impact]**...*I can't do any housework or anything because I can't stand without hanging on to my trolley… Next week I am starting meals on wheels…It is non-existent really* [General lifestyle] *I could do a few things but not now...it is walking* [reason] *I wouldn't want to go far because it gets too painful, I walk to the car'* **[Mobility & ADL and Participation]** (32, T3)	5<br>No change

ADL: Activities of Daily Living; MSK: Musculoskeletal

In the group with co-presence type 3, where there was no improvement in pain but improvement in all other domains, participant 43 used pain relief for the persisting pain enabling other domains to improve even though he was not keen on taking painkillers, and 24 experienced pain only at night. Both were in trajectory groups 1 or 2.

In type 4 (n=5) all domains were worsening. This was the second most common type of co-presentation and mirror image of that observed in Type 1. Worsening in one domain had a negative impact on the other domains. All participants were in trajectory group 5 (no change in at least 2 domains) except participants 33 and 41. The former experienced transient improvement in two domains but then worsened, so was in trajectory group 4. The latter was in trajectory group 3 where there was discordance. This participant had worsening pain and mobility which she had found depressing but by T3 she saw the pain, although severe, as manageable. The level of mobility required for her to maintain most of her

For peer review only - http://bmjopen.bmj.com/site/about/guidelines.xhtml

recreational and social interests was relatively limited and she could manage this through planning, but she had difficulty with activities involving walking in groups.

Type 5 (n=2) was similar to type 4 and both participants were in trajectory group 5 but there was improvement in psychological impact. Participant 53 had specifically addressed his depression that had resulted from the NC symptoms and 06 had adapted to the pain. However, in the timeframe of the study the reported improved psychological impact did not influence the other domains.

Participant 34 with co-presence type 6 where pain did not improve and mobility deteriorated (trajectory group 5) similarly maintained positive psychological outlook but was also able to maintain social and recreational engagement despite the pain and loss of mobility.

Participant 02 (co-presence type 7) and 15 (co-presence type 8) were both in trajectory group 3 where there was discordance. The former remained envious of those who could walk faster than he could which impacted negatively on his psychological wellbeing and would have liked to be able undertake long distance travel which was limited by his pain. Participant 15 had spinal surgery between T2 and T3 which raised his hopes of improvement although had not yet experienced improvement.

Co-presence types 9 and 10 were similar to types 4 and 5 respectively but the pain was not from NC but from other musculoskeletal problems.

We were particularly interested in those who still improved in social and recreational participation without improvement in all other domains. This was experienced by four participants for a variety of reasons highlighting those ongoing impairments across other domains does not necessarily impede improvement in participation. For 24 the pain was only at night, for 43 the pain could be controlled, and for 34 and 41 it was their positive psychological outlook.

Turning points

Thirteen participants did not mention a major change in their health. Twelve described major change in musculoskeletal problems: four described notable improvement or worsening of their pain and mobility, one had a fall that impacted wellbeing, four described worsening of joint problems (hip, knee, and neck), and four had back surgery during the data collection year. Four participants developed other major health issues (emergency abdominal surgery, Addison's disease, chronic obstructive airways disease, insertion external feeding tube). From the data for these latter participants, we were unable to detect any interaction between their trajectory and these health events.

Totality of change

There were many similarities between participants from across the different trajectories. The majority of participants lived with a partner with a total of 9 living alone. The majority of participants (from all categories) had a positive outlook on aging and wanted to be active, '*...Age is just a*

number, for me' (08, T1, 70 years). A few either had an acceptance of aging or had negative attitudes, *'You'd think I was 95 the way I'm talking to you'* (11, T2, 70 years). Most participants (from all categories) were using some form of mobility aids ranging from walking stick, wheeled walker and rollator, Nordic poles, Zimmer frame, and mobility scooters. A very few (all categories) mentioned they didn't want to become dependent on mobility aids or look older, *'I don't like them [laughs]. Vanity. I just suppose … It looks like you are an old man you know, or a disabled person'* (02, T1). In all categories, good weather/summer season enabled walking and outdoor activities and many reported limited activity during winter due to difficulties such as lack of confidence in walking when wet/icy and difficulty finding places to sit down. All participants reported a variety of self-made, convenient strategies for coping with their NC in at least one of their interviews. Participants predominantly used active, problem-focused strategies rather than passive ones such as avoiding painful activities. For example, they used solution-based strategies such as sitting/resting between walks or household chores, driving to local shops instead of walking, leaning on a shopping trolley to walk around, using a light-weight vacuum cleaner, and accepting help from family members with shopping and gardening. Participants who regularly (mostly daily, one 2-3 times a week) engaged in exercises specifically for their NC were found in all trajectory groups.

For peer review only

Discussion

For each person living with NC, we were able to identify the pattern of change at each of the three time points for each of the biopsychosocial domains of pain, mobility, psychological impact and social participation. By looking across these patterns we were able to identify trajectories over time for each domain. Participants in all trajectory categories had many attributes in common including attitudes to aging and in seeking strategies to enable themselves to function optimally.

Although there were participants where improvement occurred across all domains or there was no change across all domains, many participants reported discordance with change going in different directions in different domains, or there was no change in some domains alongside change in other domains. Published quantitative evidence has demonstrated within domain discordance: for mobility, discordance between walking capacity and actual walking (32, 33). With our qualitative data, we were able to illustrate interaction between domains, for example, pain affecting mobility, with negative psychological impact and social withdrawal and vice versa. This negative cycle might explain the within domain discordance for mobility. However, we were also able to illustrate how, for example, improvement in psychological impact or social and recreational interaction happened despite pain or mobility problems.

For ten out of fourteen participants reporting improvement in social and recreational participation, this occurred alongside improvement in all other domains. This is the best scenario and the aim of interventions for people with NC. Remaining participants reported dealing in some way specifically with their pain, mobility or psychological impact which still enabled them to improve social and recreational participation without

For peer review only - http://bmjopen.bmj.com/site/about/guidelines.xhtml

improvement in all domains. This suggests that it is possible to identify strategies to increase participation even if other domains are resistant to change. This is particularly important when considering the impact of pain. Pain is particularly problematic for this patient group and effective treatments for reducing the pain associated with NC are limited (34). For the participants where improvement in social and recreational participation was not achieved when it might be expected the reasons were: very high expectations of what for them counted as participation; did not want to cause a problem for others; there no loss of engagement to be regained. The other group warranting further consideration are those reporting worsening across all the domains and what can be done to break this perpetuating negative cycle.

Categorising and understanding change over time

Our study extends the limited current longitudinal qualitative evidence on living with back pain. We found a greater variety of trajectories than in a published study with just eight participants (see background) (12) as, in contrast to this study, we found examples of change in one domain despite no change in others as well as examples of reinforcement of change across domains. The earlier study collected data at longer time intervals (12 months over 2 years). This may have resulted in less detail about change between data points than in our study resulting in the perception of smoother trajectories. In our past qualitative study (22) which included 15 participants living with chronic back pain (excluding NC) interviewed three times in two years, we developed the following categories of participant's current experience of their back pain: past reminders (similar to fear avoidant behaviour), stuck and struggling, becalmed, and submerged. These categories describe the overall state of the participants rather than interactions that resulted in these states. Of the 15 participants, 12 changed category during the 2 years. Most of the participants started as stuck and struggling and moved to past reminders or becalmed, trajectories similar to our current study participants in categories 1 and 2. Two participants remained stuck and struggling/submerged, a similar trajectory to the participants at the bottom of our Table 2 (trajectories of no change/worsening). Our past study included cross-sectional data only from people living with diabetes (22) who were categorised in a similar way to those with back pain.

Trajectories have been identified in qualitative studies of people living with long-term conditions other than back pain where longitudinal data has been collected. In a study of 22 participants, data was collected 15 months post stroke and over the subsequent 4 months. Four different recovery trajectories were evident: (a) meaningful recovery, (b) cycles of recovery and decline, (c) ongoing disruption, (d) gradual, ongoing decline (35). The first (a) is similar to our progressive improvement trajectory, (b) may be similar to our discordant trajectories, (c) and (d) have some similarity to our category of no change; the difference may be due to the natural history of the NC where NC only worsens for a few (6). However, trajectories identified depend on the focus of analysis. A longitudinal interview study with 21 participants diagnosed with a chronic illness (e.g., diabetes, rheumatoid arthritis) focused on illness self-management (36). It was found participants go through the following phases: seeking effective self-management strategies, considering costs and benefits, creating routines and plans of action, and negotiating self-management that fits one's life. In our study we found all these strategies being used but our analysis focus was not self-management.

For peer review only - http://bmjopen.bmj.com/site/about/guidelines.xhtml

Categorising to predict outcome and complex interaction

We found many similarities between the participants in the 5 categories of trajectories and a lack of features that could be used to identify members of a particular trajectory which could be used to predict outcome. Many studies on back pain have analysed quantitative data using methods for categorising such as latent class analysis, seeking categories of patients that would allow matching of interventions to patient groups (37-39). Study participants can be categorised into clinically meaningful groups and category membership may correlate with outcome (40). However, interaction between category membership and intervention is difficult to demonstrate (38). A longitudinal study of spinal stenosis found factors predictive of subjective improvement, but none were modifiable (41). If improved social and recreational participation is our goal, our study would suggest that this is likely to be co-present with and interact with improvement across the domains of pain, mobility and psychological impact and not improve when there is no co-presence of improvement across the other domains. This interaction between domains is like that described in a cross-sectional interview study with people living with NC (5). Although our data suggests that for some participants improvement in one domain leads to improvement in another domain, this interaction is in the timeframe of hour-to-hour or day-to-day or week-to-week as occurs in complex systems (42, 43). This, what we have called co-presence, is different from the prediction of outcome over many months or years that is sought in the studies described above. However, our study also provides examples of participants where one factor did prevent or enable improvement, but this was a relatively small number of participants and the attributes varied. We suggest that seeking individual level factors that predict of outcome for people living with NC is unlikely to be helpful. However, we need to remain alert to external determinants of outcome. For example, in a US study, type of health insurance influenced rate of improvement for people with low back pain (44).

Strengths and limitations

We collected a relatively large qualitative data set with higher-than-expected participation rates in later interviews. This may have been because the repeat encounters generated a relationship of trust between researcher and participant (45). Although there are differences when interviews are conducted by telephone rather than face to face (46) our telephone interviews provided rich data for our analysis, perhaps helped by the first interview being face-to face

At each interview we focused on how the participant was at the time of the interview. However, in interviews people naturally tell stories and these stories include accounts of change that has occurred (47). What was current was sometimes difficult to disentangle and we may have interpreted later data differently given our understanding of the participant from earlier interviews (23).The data we included in the current analysis was from participants providing three interviews of rich data so they may differ from those in our study providing less data. As we proceeded through data analysis, we chose our path based on our interest in the complex nature of health, the lived experience of conditions such as NC and successful aging. Alternative data analysis paths through this data set are possible. Analysis could be tested further by interrogating the remaining unanalysed data. Reduction of data to mini statements loses richness and nuances in data but we retained the ability to go back directly to the framework and the data for fuller summaries and illustrative quotes. Data reduction enables comparison across the whole data set.

For peer review only - http://bmjopen.bmj.com/site/about/guidelines.xhtml

Although a longitudinal study, follow up was only for 12 months, a relatively short time. NC symptoms change over time. Some of our participants had already started to experience improvement in their symptoms at the start of the study. This is in line with findings of a cohort study of people with moderate NC(6).

Participants could be categorised in different ways and there was overlap between categories. This is a common finding when categorising people in both qualitative studies (22) and quantitative studies (37).

<u>Implications for clinical practice</u>
There may be benefit in those providing therapy for NC patients to identify the trajectory of their patient in order to tailor treatment. This requires careful listening as changes may be quite small so hard to discern. Although it is useful to categorise patients as we have done in this study, these categories are just useful labels to think with and guide discussions with patients. As therapists know, patient presentation can often seem messy. Having the categories of trajectory and how they potentially interact in mind may help tease out what is going on for people and identify where there is opportunity to reinforce positive change or modulate deterioration, but we need to beware of shoehorning people into categories as we could miss important changes in trajectory in one or more domains. Our study suggests therapists, while following clinical guidance for patients with NC, also need to individually tailor their interventions for patients to optimise participation in social and recreation activities that are fundamental to healthy aging.

For peer review only - http://bmjopen.bmj.com/site/about/guidelines.xhtml

For peer review only

References

1. Williamson E, Sanchez Santos MT, Morris A, Garrett A, Conway O, Boniface G, et al. The Prevalence of Back and Leg Pain and the Cross-sectional Association With Adverse Health Outcomes in Community Dwelling Older Adults in England. Spine (Phila Pa 1976). 2021;46(1):54-61.
2. Ishimoto Y, Yoshimura N, Muraki S, Yamada H, Nagata K, Hashizume H, et al. Prevalence of symptomatic lumbar spinal stenosis and its association with physical performance in a population-based cohort in Japan: the Wakayama Spine Study. Osteoarthritis Cartilage. 2012;20(10):1103-8.
3. Suri P RJ, Kalichman L, Katz JN. . Does this older adult with lower extremity pain have the clinical syndrome of lumbar spinal stenosis? JAMA. 2010;Dec 15;304(23):2628-36.
4. Lin SI, Lin RM, Huang LW. Disability in patients with degenerative lumbar spinal stenosis. Arch Phys Med Rehabil. 2006;87(9):1250-6.
5. Ammendolia C, Schneider M, Williams K, Zickmund S, Hamm M, Stuber K, et al. The physical and psychological impact of neurogenic claudication: the patients' perspectives. J Can Chiropr Assoc. 2017;61(1):18-31.
6. Wessberg P, Frennered K. Central lumbar spinal stenosis: natural history of non-surgical patients. Eur Spine J. 2017;26(10):2536-42.
7. Rowe JW, Kahn RL. Successful aging. Gerontologist. 1997;37(4):433-40.
8. Katz S CT. Critical perspectives on successful aging: Does it "appeal more than it illuminates"? The Gerontologist. 2015 Feb 1(1):26-33.
9. Foster L WA. Active and Successful Aging: A European Policy Perspective. The Gerontologist. 2014;Feb 1(1):83-90.
10. Martinson M BC. Successful aging and its discontents: A systematic review of the social gerontology literature. The gerontologist. 2015;Feb 1(1):58-69.
11. Snelgrove S LC. Living with chronic low back pain: a metasynthesis of qualitative research. Chronic illness. 2013;Dec: 9(4):283-301.
12. Snelgrove S ES, Liossi C. A longitudinal study of patients' experiences of chronic low back pain using interpretative phenomenological analysis: changes and consistencies. Psychology & health. 2013;Feb 1(2):121-38.
13. Kelley-Moore JA SJ, Kahana E, Kahana B. When do older adults become "disabled"? Social and health antecedents of perceived disability in a panel study of the oldest old. Journal of health and social behavior. 2006;Jun: 47(2):126-41.
14. Corbin J. Accompaniments of chronic illness: Changes in body, self, biography, and biographical time Res Sociol Health Care. 1987;6:249-81.
15. Bury M. Chronic illness as biographical disruption. Sociology of health & illness. 1982;4(2):167-82.
16. Anjum RL CS, Rocca E. Rethinking causality, complexity and evidence for the unique patient: a CauseHealth Resource for healthcare professionals and the clinical encounter. 2020.
17. Cilliers P. Difference, Identity and Complexity. In: Cilliers P, Preiser R, eds. Complexity, Difference and Identity Issues in Business Ethics. Dordrecht: Springer; 2010.
18. Griffiths F BJ, Byrne D, Crabtree BF, Dowrick C, Gunn J, Kokanovic R, Lamb S, Lindenmeyer A, Parchman M, Reis S. . Developing evidence for how to tailor medical interventions for the individual patient. Qualitative Health Research. 2010;Dec: 20(12):1629-41.

For peer review only - http://bmjopen.bmj.com/site/about/guidelines.xhtml

19. Weller S ER, Jamieson L, Davidson E Analysing large volumes of complex qualitative data; reflections from a group of international experts. 2019.
20. Ward L, Williamson E, Hansen Z, French DP, Boniface G, Rogers D, et al. Development and delivery of the BOOST (Better Outcomes for Older adults with Spinal Trouble) intervention for older adults with neurogenic claudication. Physiotherapy. 2019;105(2):262-74.
21. Williamson E, Ward L, Vadher K, Dutton SJ, Parker B, Petrou S, et al. Better Outcomes for Older people with Spinal Trouble (BOOST) Trial: a randomised controlled trial of a combined physical and psychological intervention for older adults with neurogenic claudication, a protocol. BMJ Open. 2018;8(10):e022205.
22. Griffiths FE, Lindenmeyer A, Borkan J, Donner Banzhoff N, Lamb S, Parchman M, et al. Case typologies, chronic illness and primary health care. J Eval Clin Pract. 2014;20(4):513-21.
23. Lewis J. Analysing qualitative longitudinal research in evaluations. Social Policy and Society. 2007;Oct: 6(4):545-56.
24. Malterud K, Siersma VD, Guassora AD. Sample Size in Qualitative Interview Studies: Guided by Information Power. Qual Health Res. 2016;26(13):1753-60.
25. Gale NK HG, Cameron E, Rashid S, Redwood S. . Using the framework method for the analysis of qualitative data in multi-disciplinary health research. BMC medical research methodology. 2013;Dec: 13(1):1-8.
26. Saldaña. Longitudinal qualitative research: Analyzing change through time: Rowman Altamira; 2003.
27. Grossoehme D, Lipstein E. Analyzing longitudinal qualitative data: the application of trajectory and recurrent cross-sectional approaches. BMC Res Notes. 2016;9:136.
28. Fadyl JK CA, Theadom A, McPherson KM, TBI Experiences Research Group, Wilkinson-Meyers L, Levack W, Starkey N, Kayes N, Feigin V, Harwood M. Optimising qualitative longitudinal analysis: insights from a study of traumatic brain injury recovery and adaptation. Nursing inquiry. 2017;Apr: 24(2).
29. Guest G, Bunce A, Johnson L. How Many Interviews Are Enough?: An Experiment with Data Saturation and Variability Field Methods. 2006;18(59).
30. Sheard L, Marsh C. How to analyse longitudinal data from multiple sources in qualitative health research: the pen portrait analytic technique. BMC medical research methodology. 2019;19(1):1-10.
31. O'Brien BC, Harris IB, Beckman TJ, Reed DA, Cook DA. Standards for reporting qualitative research: a synthesis of recommendations. Acad Med. 2014;89(9):1245-51.
32. Conway J, Tomkins CC, Haig AJ. Walking assessment in people with lumbar spinal stenosis: capacity, performance, and self-report measures. The spine journal : official journal of the North American Spine Society. 2011;11(9):816-23.
33. Smuck M, Muaremi A, Zheng P, Norden J, Sinha A, Hu R, et al. Objective measurement of function following lumbar spinal stenosis decompression reveals improved functional capacity with stagnant real-life physical activity. The spine journal : official journal of the North American Spine Society. 2018;18(1):15-21.

For peer review only - http://bmjopen.bmj.com/site/about/guidelines.xhtml

34. Bussières A, Cancelliere C, Ammendolia C, Comer CM, Al Zoubi F, Châtillon C-E, et al. Non-Surgical Interventions for Lumbar Spinal Stenosis Leading To Neurogenic Claudication: A Clinical Practice Guideline. The journal of pain. 2021.
35. Hawkins RJ, Jowett A, Godfrey M, Mellish K, Young J, Farrin A, et al. Poststroke Trajectories: The Process of Recovery Over the Longer Term Following Stroke. Glob Qual Nurs Res. 2017;4:2333393617730209.
36. Audulv Å, Asplund K, Norbergh K-G. The Integration of Chronic Illness Self-Management. Qual Health Res. 2012;22(3):332-45.
37. Meisingset I VO, Vøllestad NK, Robinson HS, Woodhouse A, Engebretsen KB, Glette M, Øverås CK, Nordstoga AL, Evensen KA, Skarpsno ES. . Novel approach towards musculoskeletal phenotypes. European Journal of Pain. 2020;May: 24(5):921-32.
38. Barons MJ GF, Parsons N, Alba A, Thorogood M, Medley GF, Lamb SE. . Matching patients to an intervention for back pain: classifying patients using a latent class approach. Journal of evaluation in clinical practice. 2014;Aug: 20(4):544-50.
39. Hill JC, Dunn KM, Lewis M, Mullis R, Main CJ, Foster NE, et al. A primary care back pain screening tool: identifying patient subgroups for initial treatment. Arthritis Rheum. 2008;59(5):632-41.
40. Hill JC, Whitehurst DG, Lewis M, Bryan S, Dunn KM, Foster NE, et al. Comparison of stratified primary care management for low back pain with current best practice (STarT Back): a randomised controlled trial. Lancet. 2011;378(9802):1560-71.
41. Matsudaira K, Hara N, Oka H, Kunogi J, Yamazaki T, Takeshita K, et al. Predictive Factors for Subjective Improvement in Lumbar Spinal Stenosis Patients with Nonsurgical Treatment: A 3-Year Prospective Cohort Study. PLoS One. 2016;11(2):e0148584.
42. Gorovitz S, MacIntyre A. Toward a theory of medical fallibility. Hastings Cent Rep. 1975;5(6):13-23.
43. Fox R. Medical Uncertainty Revisited. In: Bendelow G, Carpenter M, Vautier C, et al., eds. Gender, Health and Healing: The public / private divide London: Routledge2002.
44. Katzan IL TN, George SZ, Passek S, Frost F, Stilphen M. . The use of STarT back screening tool to predict functional disability outcomes in patients receiving physical therapy for low back pain. The Spine Journal. 2019;Apr 1(4):645-54.
45. Neale B. What is qualitative longitudinal research? : Bloomsbury Publishing; 2018.
46. Irvine A, Drew P, Sainsbury R. 'Am I not answering your questions properly?' Clarification, adequacy and responsiveness in semi-structured telephone and face-to-face interviews. Qualitative Research. 2013;13(1):87-106.
47. Algilani S, Ostlund-Lagerstrom L, Schoultz I, Brummer RJ, Kihlgren A. Increasing the qualitative understanding of optimal functionality in older adults: a focus group based study. BMC Geriatr. 2016;16:70.

Consent for publication
Not applicable

Data availability statement
All data relevant to the study are included in the article or uploaded as supplementary information.

For peer review only - http://bmjopen.bmj.com/site/about/guidelines.xhtml

Competing interests
None

Funding
This research is funded by the NIHR Programme Grants for Applied Research (reference: PTC-RP-PG-0213-20002). Preparatory work for the programme of research was supported by the Collaboration for Leadership in Applied Health Research and Care Oxford at Oxford Health NHS Foundation Trust. SEL and EW received funding from the Collaboration for Leadership in Applied Health Research and Care Oxford at Oxford Health NHS Foundation Trust and are supported by the NIHR Biomedical Research Centre, Oxford.

Contributorship statement
Sarah E Lamb (SEL) and Esther Williamson (EW) are health service researchers and physiotherapists and led the BOOST programme of work including this study and the BOOST trial. Frances Griffiths (FG) is a general medical practitioner, medical sociologist and qualitative researcher and led the qualitative study team that included Lesley Ward, a qualitative health researcher, Cynthia Srikesavan (CS), a physiotherapist and a qualitative researcher, and Graham Boniface (GB), a physiotherapy researcher.

All contributed to the qualitative study design, reading data, thematic and comparative analysis, synthesis, interpretation and reporting. LW undertook all interviews. LW and FG reviewed the interview schedules to improve the clarity and flow of the questions. CS worked with FG on analysis with GB providing independent review of coding/summaries. Any discrepancies were resolved through discussion. EW and SEL reviewed each step of the analysis and provided feedback to FG, LW, CS, and GB.

Ethics approval statement

Ethics approval was given by the London-Brent National Research Ethics Committee (REC number 16/LO/0349) on 03 March 2016.

For peer review only - http://bmjopen.bmj.com/site/about/guidelines.xhtml

Better Outcomes for Older People with Spinal Trouble (BOOST)

Semi-structured interview schedules

All three interview schedules will follow a semi-structured format. Interview content from previous interviews will inform the content and sequencing of questions in the following interviews.

First interview

We will refine the schedule for the first interview during the early phase of data collection, including the content and order of the interview questions. The content of the first interviews will be based around, but not limited to, the following set of questions.

1. Please tell me about yourself (household, work/hobbies/family).
2. Please tell me about your general health.
3. Taking a day that was a fairly normal day for you please tell me about what you do in the day, starting with when you get up.
4. Are there activities that you do regularly that you have not mentioned (prompt for hobbies, recreation, visits, holidays, social clubs, volunteering)
5. What does aging/getting older mean to you? Do you think this affects how active/involved you are?
6. Do you feel restricted in what you are able to do? If so, what are you unable to do that you would like to do? How long have you felt restricted?
    - Prompt for how much of the restriction is due to their back/leg pain.
7. How has this restriction affected different aspects of your life?
    - Prompt for physical, mental, and social aspects of their life.
8. Have you had to change the way you do things because of your back/leg pain?
    - Prompt for how they are adapting their physical environment, their behaviour, and their use of technology for mobility or communication.
9. Have you considered other ways you could change the way you do things to enable you to get round some of your restrictions?
    - Prompt for barriers to changing behaviour.
10. What have you done to try to manage the back/leg pain?
11. What exercise do you take/have you previously taken?
    - Prompt for why they take the exercise (physical/social enjoyment), does it help symptoms, are they restricted by symptoms.
    - Prompt for reasons for not exercising/barriers to exercise.

For peer review only

12. How do you feel about your ability to undertake activities/exercise?
13. What are your hopes/concerns for the physiotherapy that you will receive during the BOOST trial?
14. Do you think the BOOST programme will help with your back and leg problems?
15. The BOOST programme encourages people to be more active or do more walking. How do you feel about being asked to increase your activity and walking?

Second interview

The second interview schedule will be further developed after analysis of approximately 10 transcripts from the first round of interviews, and will be based around, but not limited to , the following set of questions:

1. Have there been any changes in your general health since the last interview?
2. How would you describe your activities now? How does this compare to 6 months ago?
3. How would you describe the problems with your back and legs now? How do these compare to 6 months ago?
4. Thinking again about the things that you would like to do, how does this compare to 6 months ago?
    - Prompt for what has changed, and in what way they changed.
5. Do you think that the physiotherapy you received in the BOOST study made any difference to your back and leg problems?
    - Prompt for whether there are change in the way they view their back/leg pain now; has this changed their habits?
6. What was your experience of the BOOST physiotherapy programme?
7. Has anything else made a difference to your back and leg problems?
8. Have your views on aging and activity changed since taking part in the study?

For peer review only

Third interview

The third interview schedule will be developed after analysis of approximately 10 transcripts from the second round of interviews. The interview schedule will encompass, and build on, the questions presented to participants in the second interview.

For peer review only - http://bmjopen.bmj.com/site/about/guidelines.xhtml

Supplementary file 2: Change framework

Participant ID	Living arrangements	Treatment arm Best Practice Advice (BPA) or BOOST programme	Participant-reported attendance	Desired treatment outcomes	Pain	6 months	12 months	Pain Trajectory	Baseline Mobility & ADL	6 months	12 months	Mobility & ADL Trajectory	Baseline Psycho impact	6 months	12 months	Psychological impact Trajectory	Baseline social & recreational participation	6 months	12 months	Social & recreational participation Trajectory	General health events	Co-morbid conditions	Coping strategies	Views on aging	Use of mobility aids	Feedback from others	Views on treatment/Trial	Were desired outcomes met?	Description of Turning points
02	Lives with spouse	BPA	Attended 2 BPA sessions	Get better (Symptom improvement)	Slight improvement in symptoms on stopping self-prescribed exercises<br>Loses balance after sustained walking<br>Pain on standing > 5 minutes	Ocassional mild pains (no more severe pain)<br>Pain reduced to acceptable level, but did not fully resolve<br>Attributes to doing exercises gently than intensive before; release of trapped nerve<br>Balance slightly improved, still issue<br>Pain on standing > 5 minutes didnt change	Ocassional, but no more severe pains<br>No change in pain (Pain reduced but did notfully resolve)<br>Not sure if due to stopping 1-2 exercises or adding other exercises to regime	Initial reduction in pain that was maintained.<br>Participant had reduced pain upon trial entry.	Mostly housebound; mobilises within house as much possible.<br>Manages walking upto 20 minutes maximum<br>If weather is good, manages 30 minutes, split into two or three 10 minute walks	Walking duration improved (30 minutes);<br>Balance improved	Mobilises around house & garden<br>Pain still affects walking<br>No change in Walking duration (remained 15-20 minutes)<br>(some seasonal effect- e.g. able to go out more in summer, in winter no place to sit down)<br>Not overly satisfied with walking- 6/10	Initial increase in mobility and back to baseline status	Frustrated depending on others for simple tasks<br>Depressed for not able to contribute to and attend rotary club events<br>Negative psychological impact of getting assistance from spouse at airports	Increased confidence that back pain wont reappear<br>Focuses more on safety while walking<br>Lacks courage to resume biking due to back not feeling strong enough for this activity<br>Fearful of symptoms returning	Participant feels envious of others who can walk faster<br>Avoids certain activities due to fear of pain	Negative impact remained throughout	Difficulty with walking/standing longer made going on holidays difficult<br>Unable to process ideas for book (due to difficulty with walking)<br>Avoids gardening, house maintainence, long travels and rotary club activities	No information	Avoids activities like gardening.<br>Limited ability to travel long-distances	Participation restrictions remained throughout	No information-Throat narrowing having a major social impact- On external feeding tube	Total 2. Hypertension; throat constriction causing swallowing issues	Adopts graded activity approach (breaks 30 minute walk into shorter walks); Pacing (sits down)<br>No new strategies adopted over time.	Current condition is mainly pain -related (90%) & 10% due to aging<br>Priortises being independant than aging<br>Accepts aging and content with overall health<br>Feels less capable than other people of same age	Does not like using aids because it makes them feel old.	No information available	Not sure if symptom improvement is due to release of trapped nerve or BOOST exercises<br>Gently doing exercises would have helped pain<br>BPA no different to previous physiotherapy participant had<br>Not 100% confident whether exercises helped or not (prev exp)<br>Wanted more information but found BPA insufficient in terms of number of sessions, content and followup.<br>Not 100% sure if improvements were due to BOOST or not<br>Sees no potential for further improvements from Physio and would try other treatments-massage & chiropractic<br>BOOST contributed to 20% in improvements<br>BOOST made participant psychologically better, than physical improvements<br>BOOST helped participant to selfmanage instead of opting for surgery.	NO.<br>No considerable pain relief	External feeding tube for throat constriction at T3<br>At T2 due to symptom improvement, no longer considers surgery for pain management
03	Lives alone; two children live closeby	BPA	Attended 3 BPA sessions	Resume physical and social activities as before	Worse night pain, builds up from day<br>Takes pain medication	Pain worsened over 5 months<br>Constant pain<br>Increased pain (particularly at night)<br>Takes more medication to sleep	Decompression laminectomy, since T2<br>Constant pain<br>Pain intensified<br>(following decompression surgery)<br>Remains on same pain medication, more dose & frequency	Progressive worsening of pain	Reduced walking distance & duration due to pain<br>Sits more when out	Pain inhibits mobility<br>Difficulty to do any ADL	Decompression laminectomy, since T2:<br>No change in Mobility and all activities (maintained)<br>Sits more often when out compared to previous year	Initial decrease in mobility that was maintained	No information	No information	Decompression laminectomy, since T2:<br>No impact on mental health	No impact on mental health	Impacted social life<br>Unable to mow the lawn<br>Avoids activities that involve physical activity, like going to city & around shops<br>Avoids social activities like shopping trips with friends<br>Attends weekly Pilates class (with modified exercises)<br>Stopped church voluntary café work	No change in participation restrictions	Decompression laminectomy, since T2:<br>No change in participation restrictions (Reduced voluntary work and attendance (church);<br>Stopped attending walking group<br>Reduced pace with Pilates class)	Participation restrictions remained throughout	Hip and leg pain-No info- Leg pain intensified after surgery	No comorbid issues reported.	Takes help from others: hired a person to maintain lawns and prune shrubs; daughter cleans stairs<br>After T1, Parks car away from destination, to integrate walking to shopping<br>Avoids doing stairs unnecessarily at home.<br>Modified social outings to reduce walking and standing	Aging is inevitable, but staying active helps staying young<br>NC is age related, accepts living with it<br>Health issues are part of aging<br>Activity is good for aging people	Not interested in trying a mobility aid as did not want to draw attention.	Other people noticed participant's limp while walking.<br>Family keen on considering other treatment options	Less optimistic of BOOST exercises, thinks surgery is final option<br>BOOST exercises were appropriate, but ineffective for pain<br>Exercises are better than nothing and good for physical health<br>BOOST has changed behaviour (increase in exercise level)<br>Pessimistic on prognosis- would worsen<br>BOOST would have been costly<br>BOOST gave quicker access to treatment	NO.<br>Reduced activity and participation.	Intensified pain after L4-5 decompression laminectomy at T3<br>Disappointed with outcome of surgery
04	Lives with spouse with a small back patio garden. Previously moved from two-storey house, as no longer able to manage stairs; close contact with children	BOOST	Attended 11 of 12 group sessions.	Be pain free and return to building trade	Pain has increased since previous levels and constant 24/7.<br>Takes multiple pain meds	No reduction in pain<br>Constant pain and flare ups if he overdoes things<br>Limits amount of pain meds to avoid cognitive side effects (Zombie)	No reduction in pain (maintained)<br>Still in lot of pain<br>Constant pain with intermittent flare ups	Baseline Pain remained throughout	[illegible]	[illegible]	[illegible]	[illegible]	[illegible]	[illegible]	[illegible]	[illegible]	[illegible]	[illegible]	[illegible]	[illegible]	[illegible]	[illegible]	[illegible]	[illegible]	[illegible]	[illegible]	[illegible]	NO.	[illegible]
24	Lives with spouse. Weekly contact with daughter	BOOST	Attended all 12 group sessions	Get better sleep	Distinct symptoms at day & night.<br>Predominantly occurs at night.<br>Night time burning pain<br>Uses gel or Paracetamol or a hot drink for pain relief	Reduced day time pain;<br>Night pain unchanged.	No change (further reduction) in day pain;<br>Night pain unchanged.	Initial reduction in day time pain was maintained.<br>Baseline night time Pain remained throughout	[illegible]	[illegible]	[illegible]	[illegible]	[illegible]	[illegible]	[illegible]	[illegible]	[illegible]	[illegible]	[illegible]	[illegible]	[illegible]	[illegible]	[illegible]	[illegible]	[illegible]	[illegible]	[illegible]	[illegible]	[illegible]
[illegible]	[illegible]	[illegible]	[illegible]	[illegible]	[illegible]	[illegible]	[illegible]	[illegible]	[illegible]	[illegible]	[illegible]	[illegible]	[illegible]	[illegible]	[illegible]	[illegible]	[illegible]	[illegible]	[illegible]	[illegible]	[illegible]	[illegible]	[illegible]	[illegible]	[illegible]	[illegible]	[illegible]	[illegible]	[illegible]

For peer review only

Supplementary file 3: Patterns of change in biopsychosocial domains at T1, T2, and T3 (ordered by treatment arm and participant study code)

		Pain			Mobility and ADL			Psychological Impact			Social and recreational participation		
**Participant IDs**	**Treatment arm**	**Baseline**	**6 months**	**12 months**	**Baseline**	**6 months**	**12 months**	**Baseline**	**6 months**	**12 months**	**Baseline**	**6 months**	**12 months**
01	BOOST												
04	BOOST												
05	BOOST												
06	BOOST												
09	BOOST												
11	BOOST												
14	BOOST												
24	BOOST												
25	BOOST												
26	BOOST												
28	BOOST												
30	BOOST												
39	BOOST												
41	BOOST												
43	BOOST												
57	BOOST												
02	BPA												
03	BPA												
07	BPA												
08	BPA												
15	BPA												
31	BPA												
32	BPA												
33	BPA												
34	BPA												
37	BPA												
38	BPA												
47	BPA												
50	BPA												
53	BPA												

BOOST: BOOST programme; BPA: Best Practice Advice; ADL: Activities of Daily Living

Legend

	When participants already had reduced pain/improved sleep/ improved mobility/ improved social and recreational participation/positive psychological impact at entry to trial or had improvements compared to a previous time point.
	Further reduction in pain, improved sleep/increase in mobility/ increase in social and recreational participation/positive psychological impact compared to a previous time point.
	Continued reduction in pain/improved sleep/increase in mobility/ increase in social and recreational participation/positive psychological impact compared to a previous time point.
	When participants already had increased pain/sleep disturbances/decreased mobility/ social and recreational participation/negative psychological impact at entry to trial or worsened status compared to a previous time point.
	Further increase in pain/sleep disturbances/decrease in mobility/decrease in social and recreational participation/negative psychological impact compared to a previous time point.
	Continued increase in pain/sleep disturbances/decrease in mobility/decrease in social and recreational participation/negative psychological impact compared to a previous time point.
	No change in pain/sleep/mobility/social and recreational participation/psychological impact compared to a previous time point.
	Lack of sufficient information from interviews.
	When pain, sleep, mobility, social and recreational participation, or psychological impact influenced by other musculoskeletal (MSK)/non-MSK issues or major health events.

For peer review only

For peer review only - http://bmjopen.bmj.com/site/about/guidelines.xhtml

Title: Longitudinal qualitative study of living with neurogenic claudication

Reporting checklist for qualitative study.

Based on the SRQR guidelines.

Instructions to authors

Complete this checklist by entering the page numbers from your manuscript where readers will find each of the items listed below.

Your article may not currently address all the items on the checklist. Please modify your text to include the missing information. If you are certain that an item does not apply, please write "n/a" and provide a short explanation.

Upload your completed checklist as an extra file when you submit to a journal.

In your methods section, say that you used the SRQRreporting guidelines, and cite them as:

O'Brien BC, Harris IB, Beckman TJ, Reed DA, Cook DA. Standards for reporting qualitative research: a synthesis of recommendations. Acad Med. 2014; 89(9):1245-1251.

		Reporting Item	Page Number
**Title**			
	#1	Concise description of the nature and topic of the study identifying the study as qualitative or indicating the approach (e.g. ethnography, grounded theory) or data collection methods (e.g. interview, focus group) is recommended	1
**Abstract**			
	#2	Summary of the key elements of the study using the abstract format of the intended publication; typically includes background, purpose, methods, results and conclusions	2
**Introduction**			
Problem formulation	#3	Description and significance of the problem / phenomenon studied: review of relevant theory and empirical work; problem statement	4, 5

For peer review only

Title: Longitudinal qualitative study of living with neurogenic claudication

Purpose or research question	#4	Purpose of the study and specific objectives or questions	5
**Methods**			
Qualitative approach and research paradigm	#5	Qualitative approach (e.g. ethnography, grounded theory, case study, phenomenolgy, narrative research) and guiding theory if appropriate; identifying the research paradigm (e.g. postpositivist, constructivist / interpretivist) is also recommended; rationale. The rationale should briefly discuss the justification for choosing that theory, approach, method or technique rather than other options available; the assumptions and limitations implicit in those choices and how those choices influence study conclusions and transferability. As appropriate the rationale for several items might be discussed together.	5
Researcher characteristics and reflexivity	#6	Researchers' characteristics that may influence the research, including personal attributes, qualifications / experience, relationship with participants, assumptions and / or presuppositions; potential or actual interaction between researchers' characteristics and the research questions, approach, methods, results and / or transferability	8
Context	#7	Setting / site and salient contextual factors; rationale	5, 6
Sampling strategy	#8	How and why research participants, documents, or events were selected; criteria for deciding when no further sampling was necessary (e.g. sampling saturation); rationale	5, 6
Ethical issues pertaining to human subjects	#9	Documentation of approval by an appropriate ethics review board and participant consent, or explanation for lack thereof; other confidentiality and data security issues	5
Data collection methods	#10	Types of data collected; details of data collection procedures including (as appropriate) start and stop dates of data collection and analysis, iterative process,	6

For peer review only

Title: Longitudinal qualitative study of living with neurogenic claudication

		triangulation of sources / methods, and modification of procedures in response to evolving study findings; rationale	
Data collection instruments and technologies	#11	Description of instruments (e.g. interview guides, questionnaires) and devices (e.g. audio recorders) used for data collection; if / how the instruments(s) changed over the course of the study	6
Units of study	#12	Number and relevant characteristics of participants, documents, or events included in the study; level of participation (could be reported in results)	Table 1<br>Pages 8, 9
Data processing	#13	Methods for processing data prior to and during analysis, including transcription, data entry, data management and security, verification of data integrity, data coding, and anonymisation / deidentification of excerpts	6
Data analysis	#14	Process by which inferences, themes, etc. were identified and developed, including the researchers involved in data analysis; usually references a specific paradigm or approach; rationale	Pages 6-8
Techniques to enhance trustworthiness	#15	Techniques to enhance trustworthiness and credibility of data analysis (e.g. member checking, audit trail, triangulation); rationale	8
**Results/findings**			
Syntheses and interpretation	#16	Main findings (e.g. interpretations, inferences, and themes); might include development of a theory or model, or integration with prior research or theory	Pages 8-20
Links to empirical data	#17	Evidence (e.g. quotes, field notes, text excerpts, photographs) to substantiate analytic findings	Pages 8-20<br><br>Tables 2, 3, 4, 5<br><br>Supplementary files 2, 3
**Discussion**			

For peer review only

Title: Longitudinal qualitative study of living with neurogenic claudication

Intergration with prior work, implications, transferability and contribution(s) to the field	#18	Short summary of main findings; explanation of how findings and conclusions connect to, support, elaborate on, or challenge conclusions of earlier scholarship; discussion of scope of application / generalizability; identification of unique contributions(s) to scholarship in a discipline or field	Pages 20-23
Limitations	#19	Trustworthiness and limitations of findings	22, 23
**Other**			
Conflicts of interest	#20	Potential sources of influence of perceived influence on study conduct and conclusions; how these were managed	26
Funding	#21	Sources of funding and other support; role of funders in data collection, interpretation and reporting	27

None The SRQR checklist is distributed with permission of Wolters Kluwer © 2014 by the Association of American Medical Colleges. This checklist can be completed online using https://www.goodreports.org/, a tool made by the EQUATOR Network in collaboration with Penelope.ai

For peer review only
